# Structural Organization and Dynamics of Homodimeric Cytohesin Family Arf GTPase Exchange Factors in Solution and on Membranes

**DOI:** 10.1016/j.str.2019.09.007

**Published:** 2019-12-03

**Authors:** Sanchaita Das, Andrew W. Malaby, Agata Nawrotek, Wenhua Zhang, Mahel Zeghouf, Sarah Maslen, Mark Skehel, Srinivas Chakravarthy, Thomas C. Irving, Osman Bilsel, Jacqueline Cherfils, David G. Lambright

**Affiliations:** 1 Program in Molecular Medicine, University of Massachusetts Medical School, Worcester, MA 01605, USA; 2 Department of Biochemistry and Molecular Pharmacology, University of Massachusetts Medical School, Worcester, MA 01655, USA; 3 The Biophysics Collaborative Access Team (BioCAT), Department of Biological Sciences, Illinois Institute of Technology, Chicago, IL 60616, USA; 4 CNRS and Ecole Normale Supérieure Paris-Saclay, 94235 Cachan, France; 5 MRC Laboratory of Molecular Biology, Cambridge CB2 0QH, UK

**Keywords:** GEF, SEC, SAXS, NS, EM, DLS, HDX, MS, autoinhibition, structure

## Abstract

Membrane dynamic processes require Arf GTPase activation by guanine nucleotide exchange factors (GEFs) with a Sec7 domain. Cytohesin family Arf GEFs function in signaling and cell migration through Arf GTPase activation on the plasma membrane and endosomes. In this study, the structural organization of two cytohesins (Grp1 and ARNO) was investigated in solution by size exclusion-small angle X-ray scattering and negative stain-electron microscopy and on membranes by dynamic light scattering, hydrogen-deuterium exchange-mass spectrometry and guanosine diphosphate (GDP)/guanosine triphosphate (GTP) exchange assays. The results suggest that cytohesins form elongated dimers with a central coiled coil and membrane-binding pleckstrin-homology (PH) domains at opposite ends. The dimers display significant conformational heterogeneity, with a preference for compact to intermediate conformations. Phosphoinositide-dependent membrane recruitment is mediated by one PH domain at a time and alters the conformational dynamics to prime allosteric activation by Arf-GTP. A structural model for membrane targeting and allosteric activation of full-length cytohesin dimers is discussed.

## Highlights

• Structural organization of cytohesins investigated by SEC-SAXS, NS-EM, DLS, and HDX-MS
• Cytohesin dimers have an elongated and conformationally dynamic quaternary structure
• Cytohesin dimers bind membranes using one PH domain at a time
• Membrane binding perturbs the structural organization to prime allosteric activation

## Graphical Abstract

**Figure:**
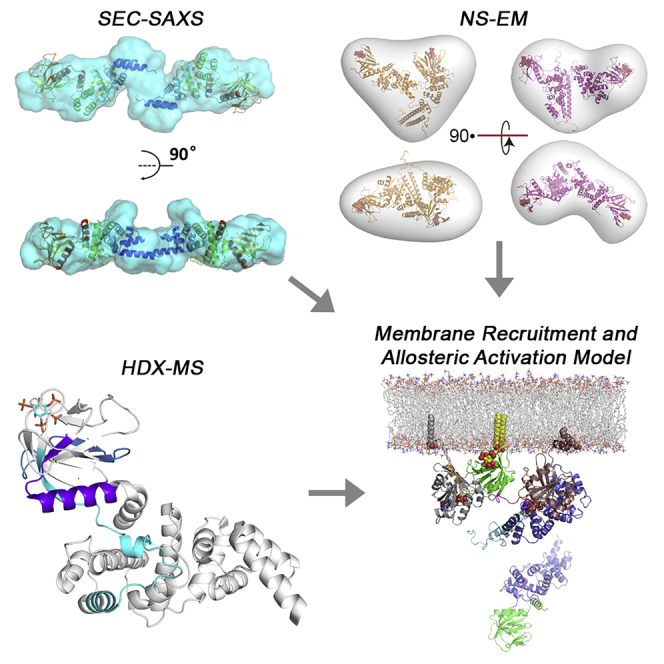

## Teaser

Arf GEFs activate Arf GTPases to control membrane dynamics. Das et al. investigate the structural organization of cytohesin dimers in solution and on membranes. The dimers adopt elongated, dynamic conformations and use one PH domain at a time to bind membranes, which perturbs the structural organization to prime allosteric activation.

## Introduction

Arf GTPases play fundamental roles in vesicle biogenesis and membrane dynamics (Donaldson and Honda, 2005, Donaldson and Jackson, 2011, Hashimoto et al., 2004, Muralidharan-Chari et al., 2009, Nie et al., 2003, Sztul et al., 2019). Activation is controlled by guanine nucleotide exchange factors (GEFs) containing a Sec7 domain, which catalyzes conversion from the inactive guanosine diphosphate (GDP)-bound state to the active guanosine triphosphate (GTP)-bound conformation (Casanova, 2007, Chardin et al., 1996, Cherfils et al., 1998). Additional domains mediate membrane recruitment through interactions with phosphoinositides, anionic phospholipids, and proteins including active Arf or Arl GTPases (Cherfils and Zeghouf, 2013, DiNitto and Lambright, 2006, Lemmon, 2004, Nawrotek et al., 2016). Membrane recruitment of Arf GTPases is mediated by a myristoylated N-terminal amphipathic helix and is required for activation by GEFs (Franco et al., 1995, Goldberg, 1998, Liu et al., 2009, Liu et al., 2010, Pasqualato et al., 2001, Pasqualato et al., 2002, Randazzo et al., 1995).

Cytohesins comprise a metazoan Arf GEF family with four mammalian paralogs (Grp1, ARNO, and cytohesins-1/4) that function in receptor signaling, endocytic trafficking, and cell adhesion/migration (Chardin et al., 1996, Fuss et al., 2006, Hafner et al., 2006, Hickman et al., 2018, Ito et al., 2018, Kolanus et al., 1996, Li et al., 2012, Mohanan et al., 2018, Ogasawara et al., 2000, Rafiq et al., 2017). All cytohesins share a common architecture comprised of a heptad repeat coiled coil (CC) domain, the Sec7 domain, and a pleckstrin-homology (PH) domain. The PH domain binds phosphatidyl inositol 3,4,5-trisphosphate (PIP_3_) and/or phosphatidyl inositol 4,5-bisphosphate (PIP_2_) (Chardin et al., 1996, Kavran et al., 1998, Klarlund et al., 1997), with affinities, specificities and spatiotemporal distributions dependent on splice variation in the phosphoinositide-binding pocket (Cronin et al., 2004, Klarlund et al., 2000, Ratcliffe et al., 2018). Two autoinhibitory elements, the Sec7-PH linker and C-terminal helix/polybasic region (CtH/PBR), strongly suppress GEF activity by occluding the active site in the Sec7 domain (DiNitto et al., 2007). Mutations in either autoinhibitory element increase GEF activity and truncation of the PBR suffices to render cytohesins constitutively active *in vitro* (DiNitto et al., 2007), albeit with reduced membrane targeting capacity (Nagel et al., 1998). Binding of membrane-associated Arf6-GTP to an allosteric site centered on the PH domain enhances membrane recruitment and relieves autoinhibition by sequestering the CtH/PBR in a groove at the Arf6-GTP/PH interface (Cohen et al., 2007, DiNitto et al., 2007, Malaby et al., 2013, Stalder et al., 2011). The CC domain is implicated in homodimerization (Chardin et al., 1996, Klarlund et al., 2001), heterodimerization with other proteins (DiNitto et al., 2010, Mansour et al., 2002), and intramolecular interactions with the Sec7-PH core (Hiester and Santy, 2013).

Atomic resolution studies have delineated structural bases for phosphoinositide recognition by the PH domain (Cronin et al., 2004, Ferguson et al., 2000, Lietzke et al., 2000), Arf substrate activation by the Sec7 domain (Renault et al., 2003), autoinhibition of the Sec7 active site (DiNitto et al., 2007), and interaction of Arf6-GTP with a linker-PH-CtH/PBR allosteric site fragment (Malaby et al., 2013). The structural organization and conformational dynamics of the monomeric autoregulatory core of Grp1, alone or artificially tethered to Arf6, was further investigated by size exclusion chromatography-small angle X-ray scattering (SEC-SAXS) in combination with single-particle negative stain-electron microscopy (NS-EM) (Malaby et al., 2018). These studies provided evidence for multiple conformations arising from flexibility of hinge residues at the N/C termini of the PH domain in the autoinhibited state as well as flexibility of the Sec7-PH linker in the allosterically activated complex. Hinge flexibility, which can be approximated by a mixture of the two conformers observed in the crystal structure of autoinhibited Grp1 (DiNitto et al., 2007), allows the Sec7 and PH domains to adopt alternative dispositions with distinct accessibility of the allosteric site. Sec7-PH linker flexibility is necessary to expose the active site and may further enhance membrane proximity of the Sec7 through partially ordered conformations in which the last five linker residues are docked in a groove at the Arf-GTP/PH interface as observed in the allosteric site complex (Malaby et al., 2013). However, our understanding of the structure and dynamics of full-length cytohesins and how dimerization mediated by the CC domain affects membrane interactions and Arf activation remains fragmentary.

**Figure 1:**
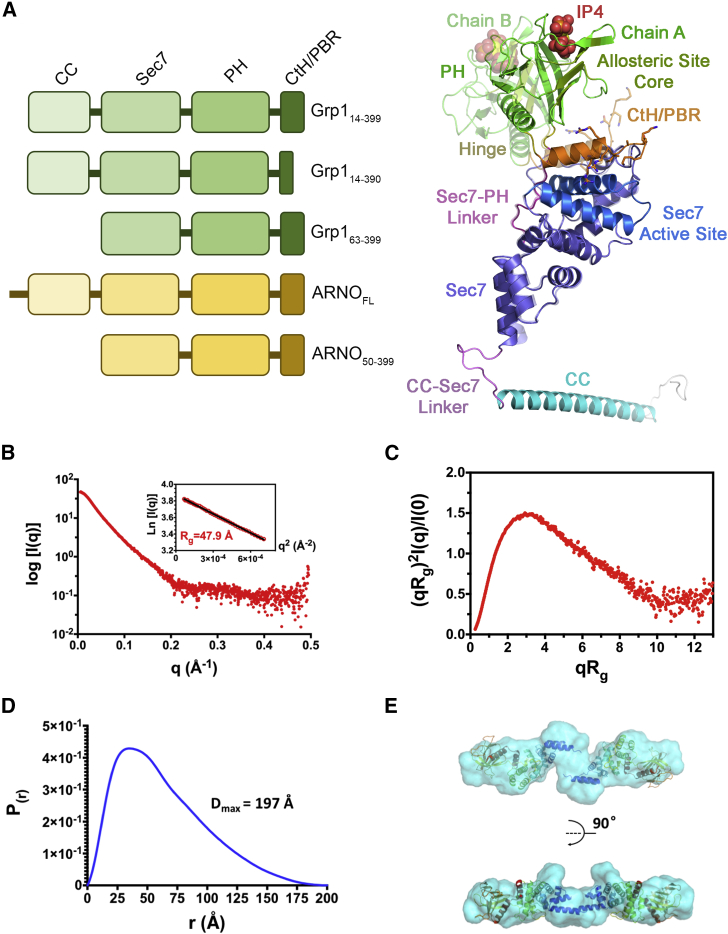
Cytohesin Architecture, Constructs and SAXS Analyses of Full-Length ARNO (A) Cytohesin constructs used in this study and hypothetical model highlighting relevant structural features. The model is based on chain A from the crystal structure of autoinhibited Grp1_63-399_ with missing regions modeled as described in the STAR Methods. Chain B is rendered as a transparent overlay after alignment of the Sec7 domains. PBR residues are depicted as sticks and the lipid head group as spheres. (B) SAXS profile of ARNO. The insert shows the Guinier plot (R_g_ × q_max_ = 1.22). (C) Dimensionless Kratky plot. The maximum is slightly shifted with respect to a fully globular protein but less than for Grp1. (D) P(r) plot giving an estimated D_max_ of 197 Å. (E) Fit of autoinhibited Grp1 structure in a representative envelope calculated by GASBOR and DAMMIN with 2-fold symmetry imposed. Additional envelopes are shown in Figure S1F.

To gain insight into the structural organization and conformational dynamics of the homodimers, cytohesins with and without the CC domain or PBR (Figure 1A) were investigated in solution using SEC-SAXS and NS-EM and in the presence of membranes using hydrogen-deuterium exchange-mass spectrometry (HDX-MS) and biochemical analyses. The results suggest an elongated, although dynamic, structural organization, with the CC domain at the center and PH domains at opposite ends. Membrane binding involves one PH domain at a time and alters the conformational dynamics within the Sec7-PH core. Our observations support the first complete model for the structural and dynamic organization of full-length cytohesins in solution and on membranes. Functional implications for autoregulation and membrane recruitment are discussed.

## Results

### Solution Structures of Full-Length ARNO and Grp1 Depict Elongated Dimers

To gain insight into the structural organization of full-length cytohesins, we analyzed the solution structure of ARNO and Grp1, which share 80% identity, using SEC-SAXS. First, we analyzed the structure of full-length ARNO (ARNO_FL_), in the absence of phosphoinositide head groups (statistics in Table S1). The SAXS profile, Kratky plot, and P(r) distribution are shown in Figures 1B–1D. ARNO_FL_ is a dimer in solution, with an estimated radius of gyration (R_G_) of 47.9 Å and a D_max_ of 197 Å. The shifted maximum of the Kratky plot (Figure 1C), the shoulder of the P(r) (Figure 1D), and *ab initio* envelopes calculated with DAMMIN and GASBOR with 2-fold symmetry imposed indicate an elongated shape (Figures 1E and S1A). By comparison, ARNO_50-400_, a construct that lacks the CC domain, has an R_G_ of 27.5 Å and a D_max_ of 98 Å, which is consistent with a monomeric structure and confirms that the CC domain drives dimerization (SAXS profile with Guinier plot, Kratky plot, and distance distribution function in Figures S1B–S1D, statistics in Table S1). *Ab initio* envelopes for this monomeric construct give a good fit with the crystal structure of autoinhibited Grp1 (DiNitto et al., 2007) (Figures S1E and S1F), indicating that the Sec7 and PH domains of ARNO likely adopt an autoinhibited conformation in solution similar to Grp1. Fitting the autoinhibited Sec7-PH tandem of Grp1 (DiNitto et al., 2007) into the SAXS envelopes of ARNO_FL_ leaves an unoccupied volume in the middle, which is predicted to correspond to the CC domain dimer (Figure 1E). These observations suggest that the CC domain is located at the center of the dimer in close proximity to the Sec7 domain, and that the PH domain is located at the extremities of the elongated structure where it makes no contact with the CC domain.

**Figure 2:**
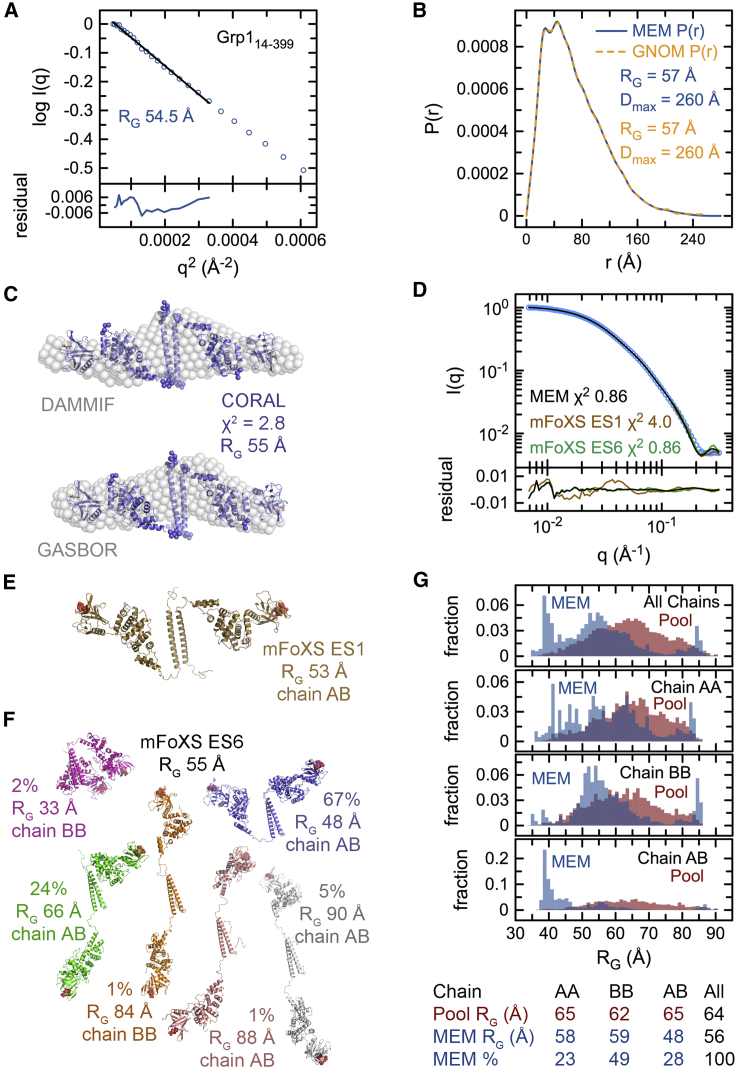
SAXS Analyses of Autoinhibited Grp1 Dimers (A) Guinier plot and fit for Grp1_14-399_. (B) P(r) distributions calculated with GNOM and MEM. (C) *Ab initio* envelopes calculated with DAMMIF or GASBOR and aligned with the rigid body CORAL model for the antiparallel CC dimer. (D) Comparison of the experimental SAXS profile with calculated profiles for the best-fitting single model (ES1) and multi-model (ES6) MultiFoXS ensembles as well as the all model MEM distribution for the antiparallel CC dimer. (E) Best-fitting single-state MultiFoXS model (ES1) for the antiparallel CC dimer. (F) Models for the best-fitting MultiFoXS ensemble (ES6) for the antiparallel CC dimer with percentages and R_G_ values. The overall R_G_ for the ensemble was calculated as the fraction-weighted mean of the individual R_G_ values. (G) Fraction-weighted histograms of R_G_ values for the MEM distribution and pool for the antiparallel CC dimer. Fraction-weighted mean R_G_ values and percentages are tabulated below.

Next, we analyzed the solution structure of the diglycine variant of autoinhibited Grp1_14-399_, a construct that includes the CC domain, in complex with the head group of PIP_3_ (Figure S2A). Sedimentation equilibrium experiments indicate that this construct is dimeric in the low micromolar concentration range (DiNitto et al., 2007, DiNitto et al., 2010). In the SEC-SAXS experiment, the peak concentration is ∼80 μM and the buffer-subtracted SAXS profiles over the main peak are characterized by a uniform R_G_. Minor peaks before and after the main peak may represent a higher-order oligomer and monomer, respectively. Singular value decomposition (SVD) of the SAXS profiles from the main peak and a post-peak buffer region revealed two significant components from which a high-quality protein scattering profile was reconstructed by Guinier-optimized linear combination (SVD-LC) as described previously (Malaby et al., 2015). Guinier analysis of the low q region yielded an R_G_ of 54.5 Å (Figure 2A; Table S2), which is approximately twice the value of 28 Å for monomeric Grp1_63-399_, which lacks the CC domain (Malaby et al., 2018). Nearly indistinguishable P(r) distributions calculated by two different algorithms (Figure 2B) provide slightly larger, and likely more accurate, estimates of R_G_ (57 Å), with tails extending to D_max_ ∼260 Å. Molecular weight (MW) estimates are near the calculated value for a dimer (Table S2), with the exception of methods prone to overestimation for non-globular geometries. The shifted maximum in a dimensionless Kratky plot (Figure S4A) indicates that dimeric Grp1_14-399_ has a more elongated structure than monomeric Grp1_63-399_. Thus, the scattering profile of Grp1_14-399_ is consistent with an elongated dimer.

The larger R_G_ and D_max_ for liganded Grp1_14-399_ compared with unliganded ARNO_FL_ suggests that head group binding to the PH domain influences the overall tertiary/quaternary structural organization and may be related to changes in H-D exchange rates accompanying membrane-association of ARNO_FL_ described below. It is unlikely that the differences are due to structural variation among cytohesins because R_G_ and D_max_ are similar for the liganded forms of ARNO_2-400_ and Grp1_14-399_ (compare Figures 2A and S3B). Scattering from the head group as well as differences related to splice variants, experimental conditions, and/or details of data processing/analysis may also contribute. Apart from these differences, our analysis of ARNO and Grp1 supports a conserved quaternary architecture for cytohesin family homodimers. We therefore used either Grp1 or ARNO for subsequent structural and biochemical analyses depending on other considerations. Given the availability of structural information for the autoinhibited core, Grp1 was a logical choice for more detailed structural modeling of SEC-SAXS and NS-EM data, whereas ARNO is thought to form the most stable homodimers and was used for GEF assays, and dynamic light scattering (DLS) and HDX-MS experiments.

### Modeling of Autoinhibited Grp1 Conformational Dynamics Using SEC-SAXS

To obtain further insight into the conformational dynamics of autoinhibited full-length cytohesins, we carried out *ab initio* and structure-based modeling of the SAXS profile for phosphoinositide-bound Grp1_14-399_. Averaged/filtered *ab initio* bead envelopes calculated without imposed symmetry using two different algorithms (DAMMIF and GASBOR) have similar elongated shapes with pseudo 2-fold symmetry consistent with the expected dyad symmetry of the CC domain (Figure 2C). More detailed information was provided by rigid body and ensemble analyses based on the crystal structure of the autoinhibited Sec7-PH core (Grp1_63-399_) connected by flexible linkers to a canonical CC model. Since the SAXS profile for Grp1_63-399_ is more accurately represented by a combination of two hinge conformations (corresponding to chains A and B from the crystal structure; see also Figure 1A) than either alone (Malaby et al., 2018) and since the topology of the CC is not known, all combinations of hinge conformers (hereafter denoted AA, BB, and AB for the dimer) were analyzed for both parallel and antiparallel topologies. Consensus secondary structure and CC prediction algorithms (Deleage et al., 1997, Frishman and Argos, 1996, Guermeur et al., 1999, Lupas et al., 1991, Rost and Sander, 1993) suggest that the CC spans residues 18–53 but may extend to residues 14–57 and/or fray at the termini.

Rigid body models for dimers consisting of two autoinhibited Sec7-PH fragments connected to CCs spanning residues 18–53 were determined using CORAL, which optimizes the position and orientation of the structured regions subject to spatial constraints for the missing residues in a library of potential backbone configurations (Petoukhov et al., 2012). The best-fitting rigid body models for both parallel and antiparallel CC topologies have nearly identical R_G_ values in the experimental range (55 versus 53–57 Å) and elongated, dyad symmetric shapes resembling the bead envelopes (Figures 2C and S4C). Nevertheless, the χ^2^ values are higher than expected for well-fitting models (χ^2^ 2.8 versus 1.0 assuming properly estimated errors) and substantial systematic deviations are evident in the residuals. Varying the length of the CC model from residues 18–41 to 14–57 in steps of four residues or restricting the length to a single residue in the middle did not qualitatively alter the overall spatial arrangement of the autoinhibited core or improve the fits, which were characterized by χ^2^ values of 3–4. Similar results were obtained for all hinge conformer combinations and CC topologies, suggesting that the SAXS profile cannot be accurately represented by a single conformation.

Best-fitting minimal ensembles selected from large pools of models with flexible connecting loops and terminal regions treated as random coils provides an alternative to rigid body modeling. The MultiFoXS algorithm was used to select best-fitting ensembles with fewer than 10 models from combined pools of 30,000 structural models generated for the 18–53 CC connected to the autoinhibited core, with equal proportions of dimers containing chain A (AA), chain B (BB), or both (AB). The best-fitting single models (ensemble state 1; ES1) have R_G_ values in the experimental range and resemble the corresponding rigid body models (compare Figures 2E–2C and S4C–S4E), including similar conformations, elevated χ^2^ values (χ^2^ = 4.0–4.1) and large systematic deviations in the residuals (Figures 2D and S4D). In contrast, the SAXS profile is well described by the best-fitting multistate ensembles (ES6) for both CC topologies (χ^2^ = 0.81–0.86; Figures 2D and S4D). These minimal ensembles have overall R_G_ values in the experimental range and are comprised of six models spanning a broad conformational space, with compact and intermediate models contributing more than extended models (Figures 2F and S4F). Here, “compact,” “intermediate,” and “extended” denote models with R_G_ values well below, near, or well above the mean for the pool. Thus, despite having elongated overall shapes, the rigid body and ES1 models are categorized as intermediate with respect to the conformational space represented by the pool.

Although the experimental profile can be fit reasonably well by minimal ensembles with as few as three to four models, the broad range of conformations represented by these ensembles suggests that Grp1 oligomers might adopt a more continuous distribution with many conformations contributing to the SAXS profile. To explore this possibility, the maximum entropy method (MEM) was used to simultaneously fit the profiles for the models in the MultiFoXS pools to the SAXS profile subject to an informational entropy restraint toward an unbiased prior distribution with equal probability for all models. To facilitate comparison and avoid over fitting, the MEM fits were terminated at χ^2^ values corresponding to the best-fitting six-state ensembles. The MEM R_G_ distributions (Figures 2G and S4G) span a broad continuous range, with contributions from many different models and a preference for compact to intermediate conformations as observed for the MultiFoXS ensembles.

We conclude that the autoinhibited Grp1_14-399_ dimer exhibits substantial structural dynamics, involving hinge conformers as observed previously for monomeric Grp1_63-399_ (Malaby et al., 2018) and larger variation in the relative orientation of the CC domain and autoregulatory core due to flexibility in the CC-Sec7 linker.

### Analysis of Grp1 Conformational Heterogeneity by Single-Particle NS-EM

**Figure 3:**
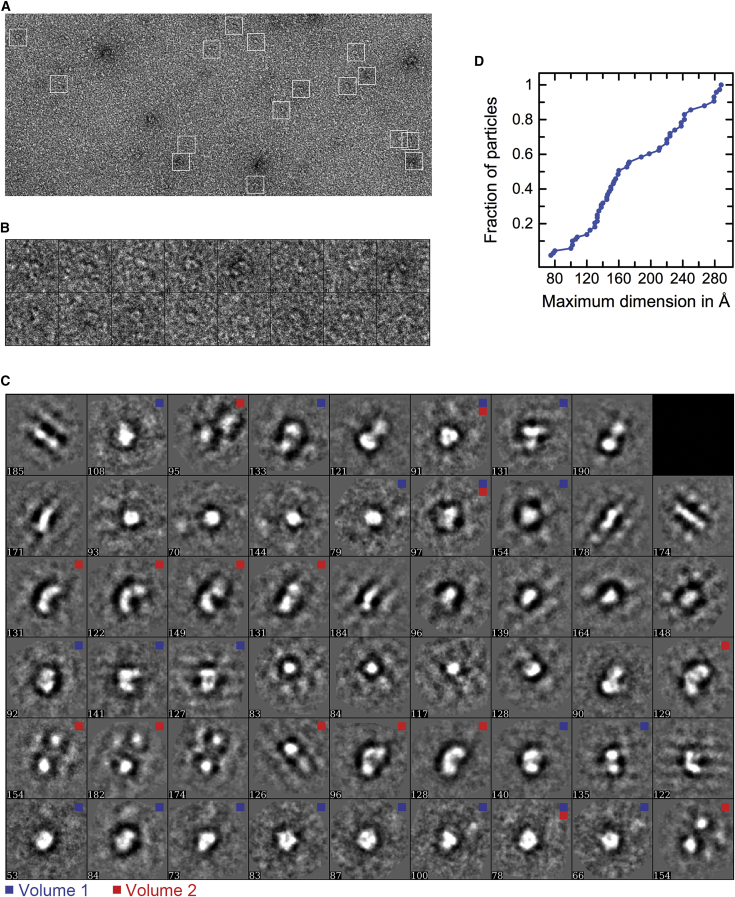
NS-EM Micrograph and Class Averages for Autoinhibited Grp1 Dimers (A) Representative area of micrograph illustrating Grp1_14-399_ particles stained with uranyl formate. Boxes indicate representative examples selected particles. (B) Enlarged views of boxed particles in (A). (C) Class averages with particle numbers in each class. Blue and red squares denote class sets used for 3D reconstruction. (D) Cumulative distribution of maximum dimensions for the class averages in (C).

Since SAXS profiles are conformationally as well as orientationally averaged, the analyses described above do not directly assess conformational heterogeneity or distinguish between small ensembles and distributions with many conformations. To address these issues and gain additional insight, the structural organization and extent of conformational variability of Grp1_14-399_ dimers was independently investigated by single-particle NS-EM. The peak fraction after size exclusion chromatography was immediately diluted, applied to freshly glow discharged carbon-coated grids, and stained with uranyl formate. Individual particles with a variety of orientations and/or shapes were observed on raw micrographs (Figure 3A). Unsupervised reference-free classification of ∼10,000 manually picked particles (Figure 3B) from 500 micrographs yielded 53 good quality classes representing ∼6,500 particles (Figure 3C). The class averages are characterized by a broad range of maximum dimensions (∼70–290 Å) and overall shapes that could in principle represent different views of an elongated dimer with an irregular conformation and/or a conformationally heterogeneous population of dimers.

**Figure 4:**
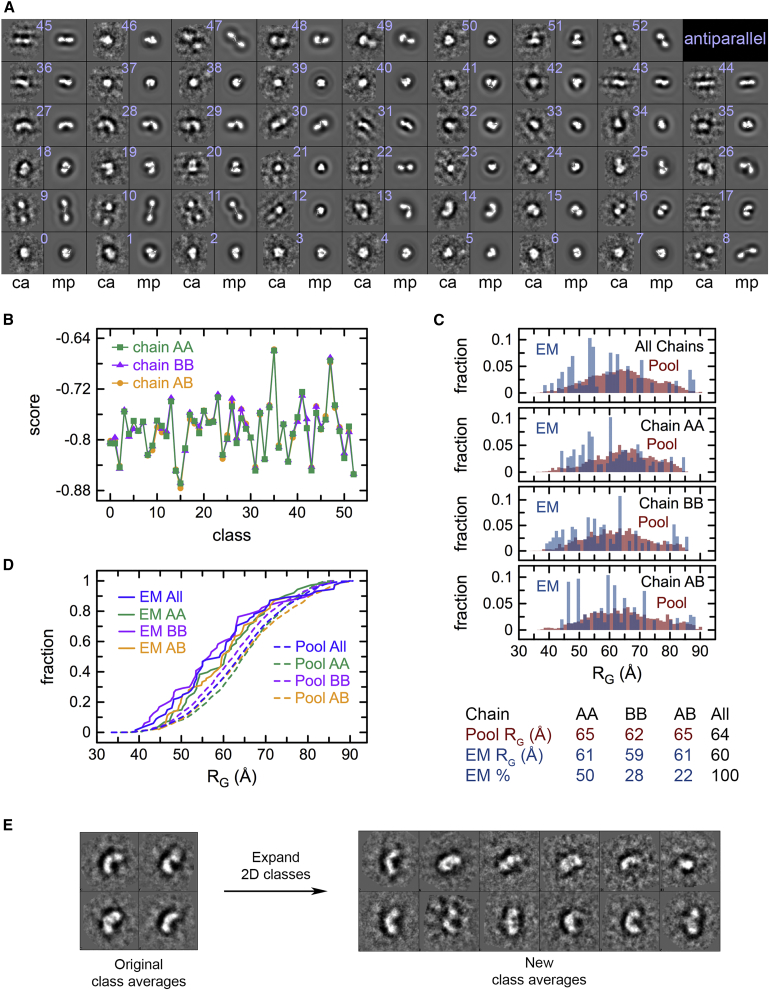
Projection Matching Analysis with Antiparallel MultiFoXS Models (A) Comparison of class averages with 3D volume projections for the best scoring MultiFoXS models. (B) Scores for comparison of class averages with 3D volume projections in (A). (C) Histograms of R_G_ values for the best scoring model in (A). (D) Cumulative distribution of R_G_ values for the best scoring models in (A). (E) Heterogeneity analysis by expansion and reclassification of morphologically similar 2D classes.

Attempts to generate a 3D reconstruction with visually selected particle classes were unsuccessful, suggesting potential conformational heterogeneity. To explore this possibility, the 2D class averages were systematically compared with 3D volume projections over the range of possible views for each model in the MultiFoXS pools (Figures 4 and S5). The best scoring model/projection for each class strongly resembles the class average (Figures 4A and S5A), indicating that the conformational diversity within the pool is sufficient to represent the range of 2D class averages. The variation in scores for the top models is substantially larger between 2D classes than between hinge conformers (Figures 4B and S5B). The best scoring models span a wide range of size and shape, with mean R_G_ values (weighted by the particle number in each class) near the range of the SEC-SAXS experiments and a preference for compact to intermediate conformations (Figures 4C, 4D, S5C, and S5D). The best scoring models for 40 of the 53 classes had antiparallel CC topology; however, the differences in score for antiparallel versus parallel topology were minor compared with the variation between classes, with the exception of a few classes where the score for the antiparallel topology was substantially better. A model-free analysis in which 2D classes with similar morphology were combined and reclassified into a larger set of new classes indicates additional conformational heterogeneity within the original set of particle classes (Figure 4E; note additional shapes in the expanded set).

**Figure 5:**
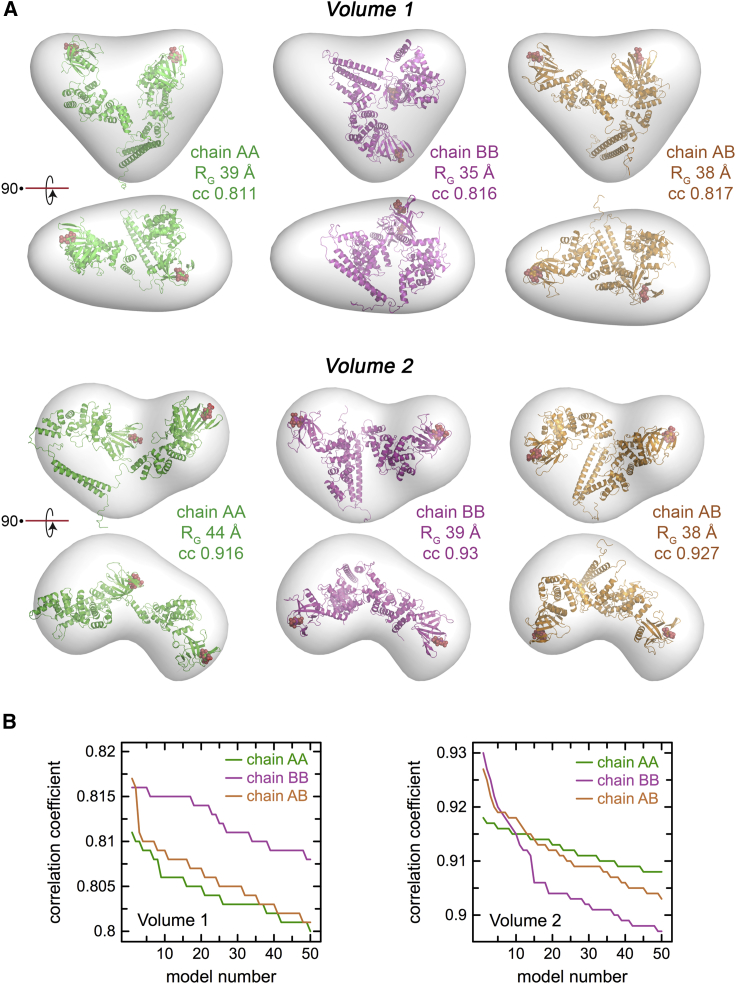
3D Reconstructions and Best-Fitting Antiparallel MultiFoXS Models (A) Comparison of the best-fitting MultiFoXS models with the volumes from 3D reconstruction and refinement for the class sets indicated in Figure 4C. (B) Correlation coefficients for the 50 best-fitting models from the comparison of each volume with the MultiFoXS pools.

The 2D analysis suggested that conformational similarity of best scoring models might improve selection of classes for 3D reconstruction. For two class sets corresponding to compact or intermediate best scoring models, 3D volumes could be built and refined. Automated docking of the MultiFoXS pools with the refined 3D volumes selected best scoring models having relatively compact conformations (Figures 5 and S6) similar to the more compact models in the six-state MultiFoXS ensembles (Figures 2F and S4F). The low resolution of ∼53 Å for the refined 3D volumes (Figure S7) likely reflects conformational heterogeneity in addition to negative staining. Some classes excluded from the sets used for 3D reconstruction have elongated class averages or correspond to views aligned with the long axis of best-fitting models with elongated conformations.

These observations provide direct evidence of substantial structural heterogeneity that lies within the conformational space sampled by the MultiFoXS pool. Flexibility in the CC-Sec7 linker is the main source of conformational variability, with a secondary contribution from hinge conformers that does not appear to be strongly influenced by dimerization. Although the analyses are most consistent with an antiparallel CC topology, the resolution is not sufficient to definitively exclude parallel or mixed topologies. These results and the preference for compact to intermediate conformations are consistent with the SEC-SAXS analysis.

### Structural Organization of Constitutively Active Grp1 Mutant Dimers in Solution

**Figure 6:**
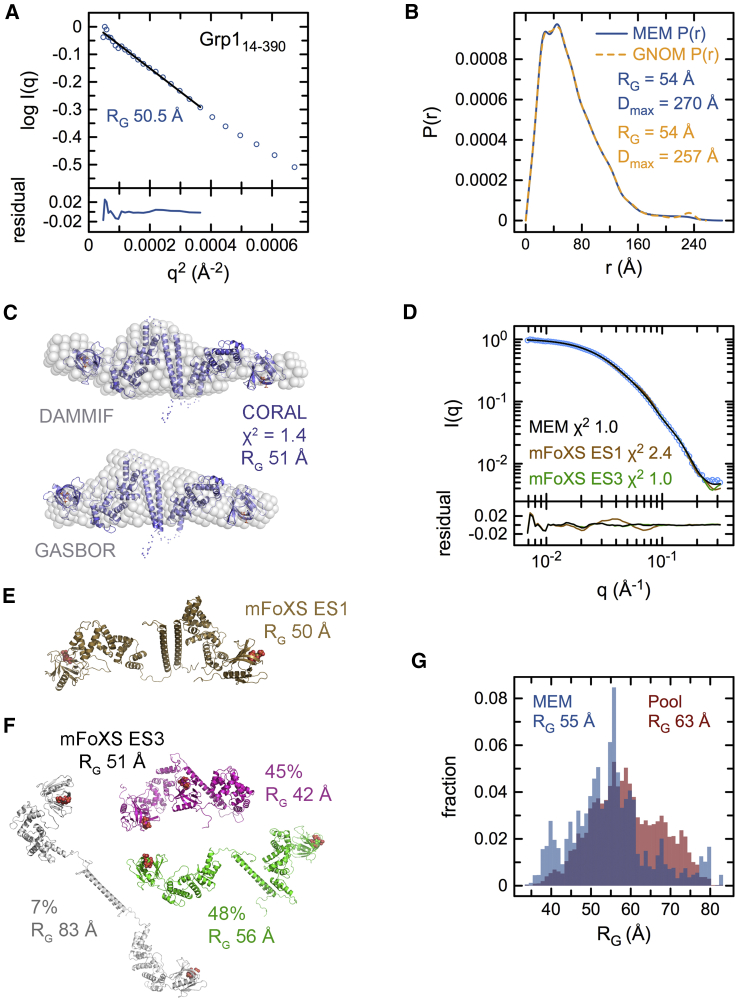
SAXS Analyses of Fully Active Grp1 Dimers (A) Guinier plot and fit for Grp1_14-390_. (B) P(r) distributions calculated with GNOM and MEM. (C) *Ab initio* envelopes calculated with DAMMIF or GASBOR and aligned with the rigid body CORAL model for the antiparallel CC dimer. (D) Comparison of the experimental SAXS profile with the calculated profiles for the best-fitting single model (ES1) and multiple model (ES3) MultiFoXS ensembles as well as the all model MEM distribution for the antiparallel CC dimer. (E) Best-fitting single-state MultiFoXS model (ES1) for the antiparallel CC dimer. (F) Models for the best-fitting MultiFoXS ensemble (ES3) for the antiparallel CC dimer with percentages and R_G_ values. The overall R_G_ for the ensemble was calculated as the fraction-weighted mean of the individual R_G_ values. (G) Fraction-weighted histograms of R_G_ values for the MEM distribution and pool for the antiparallel CC dimer. Fraction-weighted mean R_G_ values and percentages are tabulated below.

We next explored whether the active forms of the dimers are likely to have a similar or distinct structural distribution. Cytohesins lacking the polybasic motif are no longer autoinhibited and can be used as proxies for the active forms (DiNitto et al., 2007). SEC-SAXS data for one such construct (Grp1_14-390_) were collected at a peak concentration of ∼1 mg/mL. Although the signal-to-noise is lower than for the autoinhibited construct, the quality of the reconstructed protein scattering remains sufficiently high to support basic SAXS analyses as well as rigid body and ensemble modeling (Figure S2B). The R_G_ values derived from Guinier analysis (Figure 6A; R_G_ = 50.5 Å) and nearly identical GNOM and MEM P(r) distributions (Figure 6B; R_G_ = 54 Å) are slightly lower than those for Grp1_14-399_, which can be attributed to the smaller size of the construct. The estimated D_max_ values differ slightly for the GNOM (D_max_ = 257 Å) and MEM (D_max_ = 270 Å) distributions but are nevertheless similar to that of Grp1_14-399_. As observed for the autoinhibited construct, the MW estimates are consistent with a dimer (Table S2), there is a pronounced shift of the maximum in a dimensionless Kratky plot compared with monomeric Grp1_63-390_ (Figure S8A), and the *ab initio* bead envelopes are elongated (Figure 6C).

For rigid body and ensemble modeling, the CC for residues 18–53 was combined with the most frequent model in the minimal MultiFoXS ensemble for the experimental profile of the corresponding monomeric construct (Grp1_63-390_). The best-fitting rigid body models for both parallel and antiparallel CC topologies have an elongated shape reminiscent of those for Grp1_14-399_ and approximate the *ab initio* bead envelopes (Figures 6C and S8C). Although the best-fitting single-state MultiFoXS models have R_G_ values near the experimental range and overall shapes similar to the rigid body models (compare Figures 6C–6E and S8C–S8E), the χ^2^ values (1.9–2.4) are nevertheless higher than expected for well-fitting models and systematic deviations are evident in the residuals (Figures 6D and S8D). These discrepancies are largely eliminated for the best-fitting MultiFoXS ensembles and MEM distributions. In both cases, the models span a broad conformational space with overall R_G_ values in the experimental range and a preference for compact to intermediate conformations (Figures 6F, 6G, S8F, and S8G).

The characteristics of the conformational distribution for Grp1_14-390_ generally resemble those of autoinhibited Grp1_14-399_. Flexibility in the Sec7-PH linker is expected to generate additional conformational variability that was not explicitly modeled due to the technical complication of generating representative pools with two flexible linkers. Nevertheless, the results suggest that the active dimers do not have fundamentally different quaternary structural organization or conformational dynamics related to flexibility of the CC-Sec7 linker. Inspection of the models in the MultiFoXS ensemble further suggests that this tertiary/quaternary structural organization does not conflict with accessibility of the Sec7 domain active site to substrate Arf-GDP.

### ARNO Dimers Use Only One PH Domain at a Time to Bind to Membranes

**Figure 7:**
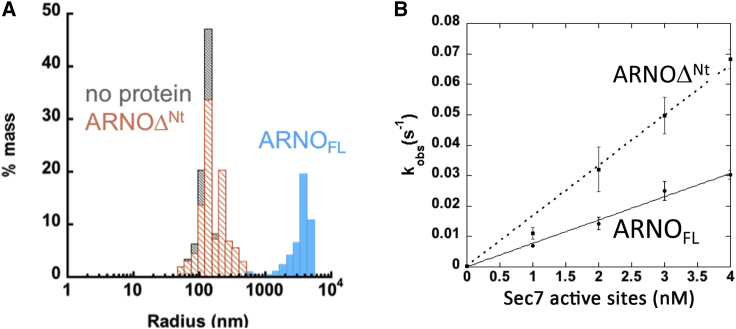
ARNO Uses Only One PH Domain at a Time to Bind to Membranes (A) DLS experiments show that dimeric ARNO_FL_, but not monomeric ARNOΔ^Nt^, aggregates PIP_2_-containing liposomes. (B) Catalytic efficiencies of the ARNO_FL_ and ARNOΔ^Nt^ measured by tryptophan fluorescence in the presence of PIP_2_-containing liposomes. The concentration of Sec7 active sites ranges from 0 to 4 nM. k_obs_ are mean ± SD for n = 2 independent experiments.

The above analysis indicates that the membrane-binding domains of cytohesins are located at the extremities of an elongated structure, where they display significant dynamics. We thus asked how this structural organization affects binding of cytohesins to membranes. First, we analyzed whether the phosphoinositide-binding sites of the two PH domains are aligned such that they can bind simultaneously to the same membrane, or are located in opposition, such that one PH domain could bind to a membrane surface at a time while the other PH domain would point away. We used liposomes that contain the anionic lipids phosphatidylserine and PI(4,5)P_2_, to which ARNO binds strongly, to discriminate between these two possibilities by DLS. Dimeric ARNO_FL_ induced conspicuous aggregation of liposomes, while monomeric ARNO_50-400_ had no effect on liposome size distribution (Figure 7A). Membrane tethering by ARNO_FL_ is possible only if the two lipid-binding sites do not bind to the same membrane at the same time, hence are located in opposition. As a consequence, the Sec7 domains, which need to be in close apposition to the membrane for efficient activation of myristoylated Arf (Karandur et al., 2017), may not be equivalent in the dimer. To test this possibility, we took advantage of the fact that ARNO displays significant GEF activity in the presence of membrane (Peurois et al., 2017) to compare the catalytic efficiencies of dimeric ARNO_FL_ and monomeric ARNO_50-400_ at the same concentration of Sec7 active sites, using myristoylated Arf1 and PIP_2_-containing liposomes (Figure 7B). The concentration range of ARNO used in the kinetics assays was chosen such that no liposome aggregation was observed. As shown in Figure 7B, dimeric ARNO_FL_ was 2-fold less active toward myrArf1 in the presence of liposomes than monomeric ARNO_50-400_ (k_cat_/K_m_ = 8.02 ± 0.48 10^6^ M^−1^ s^−1^ for ARNO_FL_ and 17.61 ± 0.64 10^6^ M^−1^ s^−1^ for ARNO_50-400_), which is consistent with a membrane-binding topology in which only one Sec7 active site is available to activate membrane-attached Arf.

### HDX-MS Analysis Shows Membranes Remodel the Sec7-PH Domain Interface

**Figure 8:**
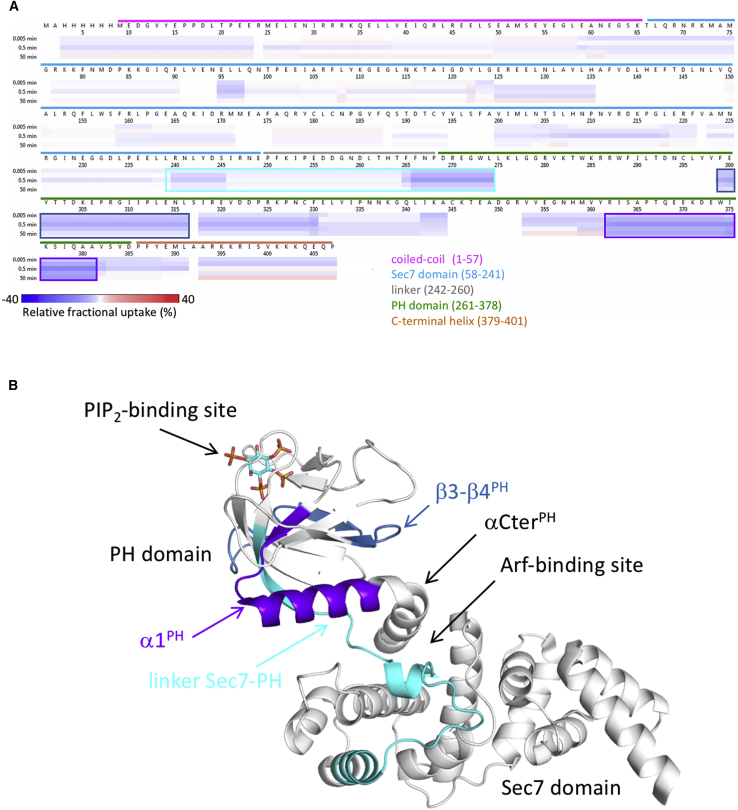
HDX-MS Analysis of the Interaction of ARNO_FL_ with Membranes (A) Heatmap showing changes in HD exchange. Relative fractional deuteration uptakes induced by the presence of PIP_2_-containing liposomes are shown at various time points as color-coded bars ranging from blue (−40%) to red (40%). Regions which can be considered significantly changed, as described in Figure S9, are boxed. Domains are highlighted by colors as indicated. The residue numbers in the His-tagged ARNO_FL_ construct are given below the sequence. The peptic peptide coverage, the butterfly plot of deuterium incorporation and the difference plot are shown in the accompanying Figure S9. (B) Regions significantly affected by PIP_2_-containing liposomes are mapped onto the structure of autoinhibited Grp1 with the color code used for the boxes in Figure 8A.

Previous studies of autoinhibited ARNO and Grp1 in solution highlighted a positive feedback loop, in which Arf-GTP binds to an allosteric site centered on the PH domain to release autoinhibition (Malaby et al., 2013, Stalder et al., 2011). The kinetics analysis above shows that full-length ARNO is readily active in the presence of liposomes, suggesting that membranes contribute to autoinhibition release independently of Arf-GTP. To analyze how membranes affect the conformation of ARNO_FL_, we used HDX-MS. We obtained good peptide coverage, although most of the CC domain, the Sec7 active site, and several phosphoinositide-binding loops in the PH domain are lacking, for which no information can be deduced (Figure S9A; Table S3). Deuterium incorporation was analyzed in the absence and presence of PIP_2_-containing liposomes and mapped on the related structure of autoinhibited Grp1 (Figures 8A and 8B). A marked protection from HD exchange was observed in loop β3–β4 (residues 293–311) in the canonical lipid-binding site of the PH domain in the presence of liposomes, confirming that ARNO binds to liposomes in the HDX-MS setup (Figures 8A, S9B, and S9C). In a more unexpected manner, deuterium incorporation was also decreased in the Sec7-PH linker and in regions immediately upstream and downstream of this linker (residues 234–269). These regions are located opposite to the GTPase-binding and the lipid-binding sites, hence are unlikely to be facing the membrane. Alternatively, protection from HD exchange by liposomes in these regions probably reflects a rearrangement of the Sec7-PH intramolecular interactions. These observations suggest that the membrane remodels intramolecular interactions in ARNO, and that this primes ARNO for full GEF activity toward membrane-attached Arf GTPase.

## Discussion

In this study, we investigated the structure, dynamics, and membrane interactions of full-length cytohesins. The data depict cytohesins as elongated dimers with substantial conformational dynamics, in which the CC dimerization domain is located at the center and the PH domains at opposite ends. We find that a large fraction of cytohesins use only one PH domain at a time to bind membranes, and that binding to membranes perturbs the structural organization by remodeling the Sec7-PH interface. The results further suggest that the CC domain restricts the conformational distribution through interaction with the Sec7-PH core. This organization is consistent with pull-down experiments using cytohesins overexpressed in cells, which indicate that the CC domain binds to the rest of the protein (Hiester and Santy, 2013). However, our data do not support a direct interaction of the CC with the PH domain, which was inferred from mutation of Thr280 in the β1/β2 loop of the phosphoinositide-binding site in the PH domain (Hiester and Santy, 2013), suggesting that the effect of this mutation may be indirect.

Another important aspect is the considerable dynamics at both the interface between the CC domain and the Sec7-PH core, and within the Sec7-PH core. In solution, cytohesins prefer compact to intermediate conformations, which may be related to intrinsic properties of the inter-domain linkers and how they stabilize intra-dimer interactions. The conformational dynamics of cytohesins is also perturbed in the presence of membranes, as shown by the unexpected change in the Sec7-PH interface upon binding to liposomes. A plausible underlying mechanism would involve repositioning of the CtH/PBR in response to favorable electrostatic interactions of the terminal basic residues with anionic phospholipids. PIP_2_ or PIP_3_ binding also reduces the electropositive potential surrounding the phosphoinositide site and may lower the barrier for repositioning the CtH/PBR. Finally, intramolecular interactions between the CtH/PBR and PH domain, such as those observed in the PH domain of Grp1 bound to Arf6-GTP (Malaby et al., 2013), may compensate for loss of interactions between these elements and the GEF site.

Although our data suggest that cytohesin homodimers have an elongated, dynamic quaternary structural organization that supports an asymmetric mode of membrane interaction, it is unlikely that cytohesins use this property to tether membranes in cells. Rather, it is plausible that they exploit it to sample the surface of the membrane one PH domain at a time, possibly using their intrinsic dynamics to convert from asymmetrical membrane binding to subsequent binding of both PH domains upon activation by Arf-GTP. Such symmetrical binding of both PH domains may depend on phosphoinositide and Arf-GTP densities, and thereby contribute to coincident detection of lipid and protein inputs. More detailed kinetic analyses as a function of phosphoinositide density over a range of myristoylated Arf-GTP concentration could help clarify whether symmetric binding occurs and under what conditions.

**Figure 9:**
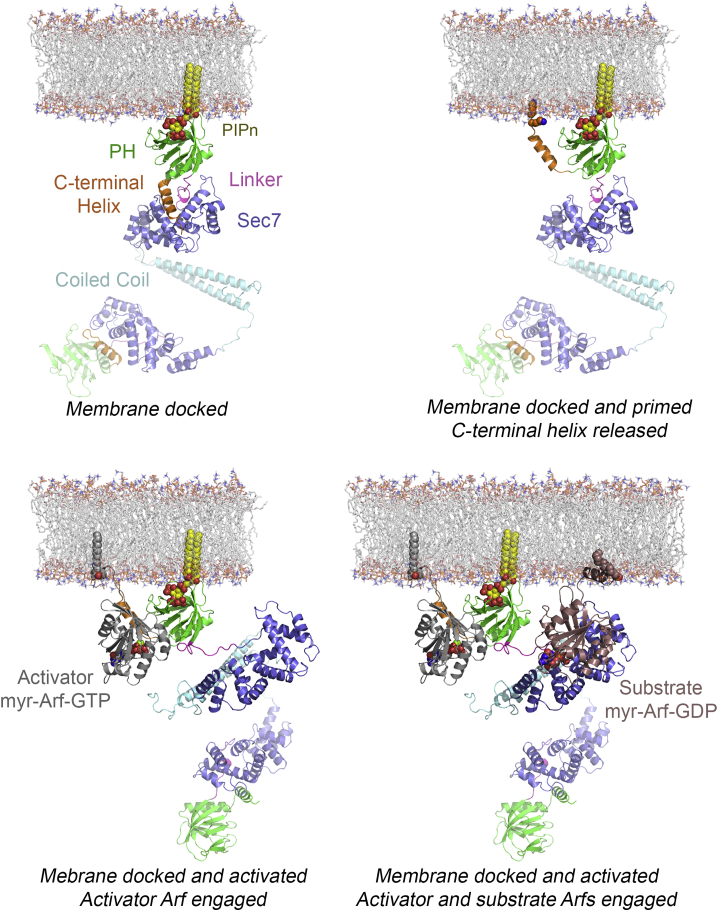
Model for Membrane Recruitment and Allosteric Activation of Cytohesins Membrane recruitment and allosteric activation of cytohesins is depicted as a series of putative intermediates consistent with observations presented here and elsewhere. The intermediates are based on the most frequent model in the six-state MultiFoXS ensemble for Grp1_14-399_ (upper left, see Figure 2F) or composites of that model and the most frequent MultiFoXS model for the Grp1-Arf6 fusion where linker residues 252–259 are flexible (Malaby et al., 2018) (other panels). The PH domain was docked with PIP_3_ in a model phospholipid bilayer based on the bound head group and residues implicated in membrane partitioning as described previously (Malaby et al., 2013). Activator myr-Arf6-GTP is shown in the orientation observed in the allosteric site complex. Substrate myr-Arf1-GDP was acquired by superposition with the Sec7 domain in the ARNO complex with NΔ17Arf1-GDP (Renault et al., 2003). Myristoylated N-terminal helices were modeled in arbitrary configurations consistent with membrane partitioning. The POPC bilayer membrane was derived from the coordinates of a molecular dynamics simulation (Heller et al., 1993).

The observations here and in previous studies suggest a structural dynamic model for allosteric activation of cytohesins by membranes and Arf-GTP (Figure 9). In the cytosol, autoinhibited cytohesin dimers adopt an elongated structural organization with considerable intrinsic dynamics. Membrane recruitment is initially mediated by electrostatic interactions with bulk anionic phospholipids, which allows a lateral hopping search for rare PIP_2_ or PIP_3_, as predicted by molecular dynamics for the monomeric Grp1 PH domain (Lai et al., 2013). Considering that hopping involves transient diffusion into the cytoplasm near the membrane surface (Chen et al., 2012) and that a significant population of cytohesins binds the membrane one PH domain at a time, intramolecular dynamics within the dimers may allow both PH domains of the dimer to participate in alternation to increase efficiency. Once a phosphoinositide is encountered by one of the PH domains, a docked intermediate is formed in which the other subunit of the dimer is disposed toward solution (Figure 9, upper left). This docked intermediate would precede formation of a partially active “primed” intermediate, in which the CtH/PBR is repositioned from the GEF active site through electrostatic interactions with anionic phospholipids (Figure 9, upper right) as well as intramolecular interactions with the PH domain, similar to the conformation in the Arf-GTP-PH complex (Malaby et al., 2013). This would trigger accumulation of a membrane-attached myrArf-GTP pool, allowing subsequent allosteric activation by binding of Arf-GTP to the PH domain, stabilization of the repositioned C-terminal helix and release of the linker from the Sec7 active site to attain a fully active intermediate (Figure 9, lower left). Docking of the last five linker residues (^261^TFFNP^265^) in a groove formed at the PH domain/myr-Arf-GTP interface would further enhance the stability of the complex and promote membrane proximal orientations of the Sec7 domain for engagement of membrane-associated myr-Arf-GDP (Figure 9, lower right) as described for the monomeric Grp1-Arf6-GTP fusion (Malaby et al., 2018). The four intermediates depicted in the model explain the structural, dynamical, and biochemical data currently available. It is plausible that allosteric activation of one subunit by Arf-GTP also facilitates engagement of the second subunit, which together may account for the positive feedback effect mediated by Arf-GTP at the surface of liposomes (Stalder et al., 2011). In the context of this model, conformational dynamics influenced by intra/intermolecular interactions generate multiple conformations for each of the intermediates and contribute to transitions between them.

Together, the analysis of ARNO and Grp1 indicates a structural dynamic pathway of cytohesin dimers in which the central CC and Sec7 domain interface modulates the positions of the lipid-binding sites in the dimer and membranes/phosphoinositides promote conformational changes that prime cytohesins for activation. SAXS modeling and HDX-MS are not expected to capture specific intramolecular interactions or how they are remodeled during the activation process, and further experiments are required to elucidate the structural details. The approach implemented here may be applicable to cytohesin heterodimers as well as other macromolecular complexes with conformationally dynamic states.

## STAR★Methods

### Key Resources Table

**Table:** 

REAGENT or RESOURCE	SOURCE	IDENTIFIER
**Bacterial and Virus Strains**		

BL21(DE3) Competent Cells	Novagen	Cat#69450
XL-10 Gold Ultracompetent Cells	Agilent	Cat#200314

**Chemicals, Peptides, and Recombinant Proteins**		

Inositol 1,3,4,5 tetrakis-phosphate, Potassium Salt (IP4)	Cell Signals	Cat#803
Uranyl Formate	EM Sciences	Cat#22450
phosphatidylcholine (PC)	Avanti	Cat#840053C
phosphatidylethanolamine (PE),	Avanti	Cat#840022C
Phosphatidylserine (PS)	Avanti	Cat#840032C
cholesterol	Sigma	Cat#C8667
NBD-PE	Avanti	Cat#810144C
phosphatidylinositol-4,5-triphosphate (PIP2)	Avanti	Cat#850185P

**Critical Commercial Assays**		

Wizard Plus Miniprep DNA Purification Kit	Promega	Cat#A7510
Wizard SV Gel and PCR Cleanup Kit	Promega	Cat#A9281

**Deposited Data**		

Grp1 63-399 + IP4	DiNitto et al., 2007	PDB: 2R09
Cytohesin-2; ARF nucleotide-binding site opener, ARNO truncation mutant	This paper	SASBDB: SASDEV9 https://www.sasbdb.org
Cytohesin-2; ARF nucleotide-binding site opener, ARNO	This paper	SASBDB: SASDEW9 https://www.sasbdb.org
Grp1 14-399 + IP4 SAXS with DAMMIF and GASBOR models	This paper	SASBDB: SASDG64 https://www.sasbdb.org
Grp1 14-399 + IP4 SAXS with antiparallel CORAL and MultiFoXS models	This paper	SASBDB: SASDG94 https://www.sasbdb.org
Grp1 14-399 + IP4 SAXS with parallel CORAL and MultiFoXS models	This paper	SASBDB: SASDGA4 https://www.sasbdb.org
Grp1 14-390 + IP4 SAXS with DAMMIF and GASBOR models	This paper	SASBDB: SASDG74 https://www.sasbdb.org
Grp1 14-390 + IP4 SAXS with antiparallel CORAL and MultiFoXS models	This paper	SASBDB: SASDGB4 https://www.sasbdb.org
Grp1 14-390 + IP4 SAXS with parallel CORAL and MultiFoXS models	This paper	SASBDB: SASDGC4 https://www.sasbdb.org
ARNO 2-400 + IP4 SAXS with DAMMIF, GASBOR and antiparallel CORAL models	This paper	SASBDB: SASDG84 https://www.sasbdb.org
Grp1 14-399 + IP4 NS-EM Volume 1 with best antiparallel model	This paper	EMDB: EMD-20628 PDB: 6U3Ehttp://www.emdatabank.org
Grp1 14-399 + IP4 NS-EM Volume 2 with best antiparallel model	This paper	EMDB: EMD-20629 PDB: 6U3Ghttp://www.emdatabank.org

**Recombinant DNA**		

Plasmid: Modified pET15 (pDL2)	DiNitto et al., 2007	N/A
Mouse Grp1 14-399 in pDL2	DiNitto et al., 2007	N/A
Mouse ARNO 2-400 (diglycine variant) in pDL2	DiNitto et al., 2007	N/A
Human FL ARNO 3G pET-8c	Antonny et al., 1997	N/A
Human ARNO 3-299 pET-8c	Antonny et al., 1997	N/A
Arf1 pET-3c	Franco et al., 1993	N/A

**Software and Algorithms**		

ADP_EM	Garzón et al., 2007	http://chaconlab.org/hybrid4em/adp-em
ATSAS	Petoukhov et al., 2012	www.embl-hamburg.de/biosaxs/software.html
CHIMERA	Pettersen et al., 2004	https://www.cgl.ucsf.edu/chimera/download.html
CORAL	Petoukhov et al., 2012	www.embl-hamburg.de/biosaxs/software.html
DAMAVER (DAMSEL, DAMSUP, DAMAVER and DAMFILT)	Volkov and Svergun, 2003	www.embl-hamburg.de/biosaxs/software.html
DAMMIF	Franke and Svergun, 2009	www.embl-hamburg.de/biosaxs/software.html
DELA	Malaby et al., 2015	DOI: 10.1107/S1600576715010420
EMAN2	Tang et al. (2007)	http://blake.bcm.edu/emanwiki/EMAN2
FoXS	Schneidman-Duhovny et al., 2013	https://integrativemodeling.org
GASBOR	Svergun et al., 2001	www.embl-hamburg.de/biosaxs/software.html
GNOM	Svergun (1992)	www.embl-hamburg.de/biosaxs/software.html
IMOD	Kremer et al. (1996)	http://bio3d.colorado.edu/imod/
IMP	Russel et al., 2012	https://integrativemodeling.org
MODELLER	Webb and Sali, 2014	https://salilab.org/modeller/
MultiFoXS	Carter et al., 2015	https://integrativemodeling.org
PRIMUS	Konarev et al., 2003	www.embl-hamburg.de/biosaxs/software.html
PyMol	SBGRID	https://pymol.org
RRT_SAMPLE	Raveh et al., 2009	https://integrativemodeling.org
SBGRID	Morin and Sliz, 2013	https://sbgrid.org
SUPCOMB	Kozin and Svergun, 2001	www.embl-hamburg.de/biosaxs/software.html
dammif.sh	Malaby et al., 2018	N/A
e2classvsproj.py	EMAN2	http://blake.bcm.edu/emanwiki/EMAN2
e2classesvsprojs.py	Malaby et al., 2018	N/A
e2classesvsprojs_best_scores.py	Malaby et al., 2018	N/A
e2classesvsprojs_extract_best.py	Malaby et al., 2018	N/A
e2classesvsprojs_generate_best_list.py	Malaby et al., 2018	N/A
e2pdb2mrc.py	EMAN2	http://blake.bcm.edu/emanwiki/EMAN2
e2pdbs2mrcs.py	Malaby et al., 2018	N/A
e2classesvsprojs_pipeline.txt	Malaby et al., 2018	N/A
extract_models.sh	This paper	N/A
extract_rg.sh	This paper	N/A
filenames_rg.py	This paper	N/A
foxs.sh	Malaby et al., 2018	N/A
foxs_component_summation_resample.sh	This paper	N/A
foxs_component_summation.py	This paper	N/A
foxs_resample.py	This paper	N/A
gasbor.sh	Malaby et al., 2018	N/A
histogram.py	This paper	N/A
histogram_fraction.py	This paper	N/A
multifoxs_filenames.py	This paper	N/A
multifoxs_mem_pipeline.txt	This paper	N/A
multifoxs_pipeline.txt	Malaby et al., 2018	N/A

**Other**		

HiTrap Q HP	GE Healthcare Life Sciences	Cat#17-1154-01
HiTrap SP HP	GE Healthcare Life Sciences	Cat#17115201
His-Trap HP	GE Healthcare Life Sciences	Cat#17-5248-02
Gilder Copper grids, 400 Mesh	Ted Pella	Cat#G400
Half Area 96 Well Microplate	Corning	Cat#3679
HiLoad Superdex 75 PG 16/60	GE Healthcare Life Sciences	Cat#28989333
HiLoad Superdex 200 PG 16/60	GE Healthcare Life Sciences	Cat#28989335
Superdex 200 Increase 5/150	GE Healthcare Life Sciences	Cat#28990945

### Lead Contact and Materials Availability

Further information and requests for resources and reagents should be directed to and will be fulfilled by the Lead Contact, David Lambright (David.Lambright@umassmed.edu). This study did not generate new unique reagents.

### Experimental Model and Subject Details

Constructs of the diglycine splice variants of mouse Grp1 and ARNO with N-terminal 6xHis tags were purified after heterologous expression in the bacterial strain BL21(DE3).

### Method Details

#### Constructs, Expression and Purification

Constructs corresponding to the diglycine variants of Grp1 and ARNO_2-400_ were amplified using Vent polymerase, digested with BamHI/SalI, and ligated into modified pET15b vectors that incorporate an N-terminal his tag (MGHHHHHHGS) (DiNitto et al., 2007). BL21(DE3) cells (Novagen) transformed with the plasmids were grown in 2xYT supplemented with 100 mg/L ampicillin to OD_600_ 0.2-0.4 and induced with 50 μM IPTG for 14-18 hrs at 18°C. Cells pellets were resuspended in buffer (50 mM Tris, pH 8.0, 150 mM NaCl, 2 mM MgCl_2_, 0.1% 2-mercaptoethanol) and incubated with 0.1 mM PMSF, 0.2 mg/ml lysozyme, and 0.01 mg/ml protease free DNAse I (Worthington). Lysates were sonicated, centrifuged at 30,000×g for 1 hr with 0.5% Triton X-100 and purified over Ni-NTA followed by ion exchange with HiTrap Q, and gel filtration on Superdex-200 (GE Healthcare).

Myristoylated Arf1 (myrArf1) was co-expressed in *Escherichia coli* with yeast N-myristoyl transferase (NMT) and purified as described previously (Beraud-Dufour et al., 1998). Full-length ARNO carrying a 3G sequence in the membrane-binding site (ARNO_FL_), which binds PI(4,5)P_2_ and PI(3,4,5)P_3_ equally (Klarlund et al., 2000) and a construct truncated for the N-terminal CC domain (residues 50-400; ARNOΔ^Nt^) were cloned into pET-8c vector (kind gift of Bruno Antonny, CNRS, Sophia-Antipolis, France) and overexpressed in *Escherichia coli*. Untagged ARNOΔ^Nt^ was purified as described previously (Peurois et al., 2017). Expression of ARNO_FL_, which carries a N-terminal 7-His tag, was induced for 3h at 37°C by addition of 0.5 mM of IPTG. Cell pellets were resuspended in 20 mM NaPO_4_ pH 7.4, 500 mM NaCl, 2 mM betamercaptoethanol and 10 mM imidazole, and then disrupted using a French press. The cleared lysate supernatant was first purified by a Ni-NTA affinity chromatography step (HisTrap FF, GE Healthcare) and then submitted to a Superdex 200 16/600 column (GE Healthcare) equilibrated with 20 mM Tris pH 7.5, 250 mM NaCl and 2 mM betamercaptoethanol. Proteins were more than 95% pure as judged by SDS-PAGE analysis.

#### SEC-SAXS Data Collection and Processing

SEC-SAXS data sets for Grp1 constructs and ARNO_2-400_ were collected at the BioCAT Sector 18-ID beamline at the Argonne National Laboratory Advanced Photon Source. Samples were incubated with a 1.2 molar excess of inositol 1,3,4,5-tetrakis phosphate (IP_4_) for 1-5 hrs, concentrated to 10-20 mg/ml and injected onto 3 ml Superdex-200 Increase columns (GE Healthcare) equilibrated with 20 mM Tris, pH 8.0, 150 mM NaCl, 2 mM MgCl_2_, 0.1% 2-mercaptoethanol, 5% glycerol, and 1 μM IP_4_. Column outlets were connected to the flow cell and SAXS data sets acquired with 1 s exposures at 5 s intervals during elution. Raw SAXS images were radially averaged on a log scale over the q range 0.00621-0.333 Å^-1^, normalized by the incident beam intensity, and further processed to reconstruct scattering profiles for the protein by buffer subtraction with or without automatic determination of an optional scaling constant or by singular value decomposition and linear combination (SVD-LC) as described (Malaby et al., 2015). The SVD-LC profiles typically had higher signal-to-noise, fewer subtraction artifacts, and were used for subsequent analyses. SEC-SAXS data sets for Grp1_63-399_ and Grp1_63-390_ were collected and processed as described previously (Malaby et al., 2018).

ARNOΔ^Nt^ data were collected using an inline HPLC-coupled SAXS instrument (SWING beamline, SOLEIL Synchrotron, France). 350 μg ARNOΔ^Nt^ was injected in a 40 μL volume (8 mg/ml) into a size exclusion chromatography column (Bio SEC-3 300 Å, Agilent Technologies, Inc.) equilibrated with elution buffer (20 mM Tris pH 8.0, 150 mM NaCl and 1 mM DTT), prior to the SAXS data acquisition. The buffer scattering signal was recorded for the first 90 images, then 240 images for the sample. SAXS images were processed with the FOXTROT suite (SOLEIL synchrotron, SWING beamline) to generate individual curves. Data intensity and quality was evaluated by plotting the I(0) and R_G_ as a function of frames. Curves from consecutive images showing high intensity I(0) and similar R_G_ were averaged. ARNO_FL_ data were collected at BM29 beamline, ESRF, France. Images were recorded throughout the HPLC elution process using the Bio Sec300 column. 600 μg in 60 μL volume was injected. Data reduction to absolute unit, subtraction and averaging was done with the EDNA pipeline implemented in the ISPyB software (ESRF BM29 beamline).

#### Basic SAXS Analyses and Ab Initio Modeling

For the diglycine variants of Grp1 and ARNO_2-400_, Guinier analyses and dimensionless Kratky plots (Durand et al., 2010) were calculated in DELA (Malaby et al., 2015). P(r) distributions were calculated using GNOM (Svergun, 1992) in PRIMUS (Konarev et al., 2003) and MEM with a sine prior in DELA (Malaby et al., 2015, Malaby et al., 2018). *Ab initio* bead envelopes were calculated using DAMMIF (Franke and Svergun, 2009) and GASBOR (Svergun et al., 2001). Typically, 100 bead envelopes were averaged/filtered in groups of 10 using DAMAVER (Volkov and Svergun, 2003) and the process repeated on the averaged/filtered models to generate the final models, which were aligned with atomic coordinates using SUPCOMB (Kozin and Svergun, 2001).

For the triglycine variants of ARNO, SAXS data analyses were performed with the ATSAS 2.8.3 package (Franke et al., 2017). Radii of gyration (R_G_) were evaluated by Guinier Wizard using the data within the range of Guinier approximation sR_G_<1.3 and by Distance Distribution Wizard, both of which are modules of the PRIMUS program. The maximum distance D_max_ was estimated with PRIMUS and refined by trial and error with GNOM. The distance distribution functions P(r) were calculated with GNOM. The dimensionless Kratky plot was calculated by plotting (qR_G_)^2^I(q)/I(0) against qR_G_. Molecular weights were estimated by Primus Molecular Weight Wizard using different algorithms. The fit between scattering amplitude calculated for the crystal structure of autoinhibited Grp1Δ^Nt^ (residues 63-399) and the SAXS curve of ARNOΔ^Nt^ was calculated with CRYSOL. *Ab initio* envelopes were calculated with GASBOR and DAMMIN over the q range 0.0025-0.5 Å^−1^ for ARNO_FL_ and 0.01-0.600 Å^−1^ for ARNOΔ^Nt^. P2 symmetry was imposed for ARNO. The resulting models were further compared using SUPCOMB and clustered with DAMCLUST. The consensus of the calculated models was represented by the lowest Normalized Spatial Discrepancy (NSD), which was determined by DAMSEL. The comparison of models and superposition was performed with SUPCOMB.

#### Rigid Body and Ensemble Modeling

Models for the autoinhibited and active cores were derived from the crystal structure of Grp1_63-399_ (PDB: 2R09) or the most frequent model in the minimal three state MultiFoXS ensemble for Grp1_63-390_, respectively. CC models were generated with CCBuilder (Wood et al., 2014). The CC-Sec7 domain linker and missing terminal residues were built with MODELLER (Webb and Sali, 2014) in CHIMERA (Pettersen et al., 2004). Rigid body modeling was performed with CORAL (Petoukhov et al., 2012). For ensemble and MEM analyses, pools of 10,000 models for each chain/topology were generated with RRT_SAMPLE (Raveh et al., 2009). The head group was represented as atoms in glycine residues that retained the chemical information in the last column of the PDB file required to specify the correct scattering form factors. Components for partial scattering profiles were calculated with FoXS (Schneidman-Duhovny et al., 2013) and minimal best fitting ensembles determined using MultiFoXS (Carter et al., 2015). For MEM distributions, scattering profiles were calculated by summation of the components generated by FoXS, with coefficients for the best-fitting MultiFoXS ensemble, as described (Schneidman-Duhovny et al., 2013) and resampled with linear interpolation to match the q sampling of the experimental profile. MEM distributions were calculated with an unbiased prior in DELA (Malaby et al., 2015) as described (Skilling and Bryan, 1984).

#### Electron Microscopy Sample Preparation and Negative Staining

Grp1_14-399_ was incubated with IP_4_ for 2 hrs prior to concentration and size exclusion chromatography on a Superdex-200 column equilibrated with 20 mM Tris, pH 8.0, 150 mM NaCl, 0.1% 2-mercaptoethanol, and 1 μM IP_4_. Protein from the peak fraction was immediately diluted, applied to glow discharged carbon coated Gilder copper 400 mesh grids (Ted Pella), incubated for 1 min, rinsed with deionized water, and stained with 0.75% (w/v) uranyl formate (EM Sciences) as described (Booth et al., 2011). Images were acquired on a Philips CM120 electron microscope operated at 120 kV using a TVIPS 2k x 2k CCD (TemCam-F224HD) camera with a nominal magnification of 28,000, corresponding to a calibrated pixel size of 6.5 Å at the specimen level. A total of 500 micrographs were collected with a nominal defocus range of −1.2 to −3.2 μm and a low dose of ∼30 electrons/Å.

#### Image Processing, Particle Picking and 2D Classification

Images were processed with EMAN2 (Tang et al., 2007) after X-ray removal with IMOD (Kremer et al., 1996). Approximately 10,000 particles were manually picked with a box size of 80×80 pixels. Following contrast transfer function (CTF) fitting and preprocessing of extracted images, particle sets were built. One hundred 2D class averages were generated by unsupervised reference-free classification. After discarding poor quality classes, 53 classes comprising 6504 particles remained.

#### 2D Heterogeneity Analysis

Heterogeneity within 2D classes was analyzed using the 2D heterogeneity module in the 3D refinement section of EMAN2. Particles for similar classes were grouped to generate new sets, which were reclassified by reference free class averaging with the center set to the center of mass.

#### 3D Volume Reconstruction and Refinement

Initial models were built for two particle sets consisting of 20 (Volume 1) or 15 (Volume 2) classes selected on the basis of qualitative similarity in the overall size and shape of the best fitting model projections for each class. Final 3D refinement with full CTF correction against the starting models was carried out by the gold-standard procedure in EMAN2 without imposed symmetry (i.e. C1). The resolution of the final 3D reconstructions was conservatively estimated to be 53 Å based on a Fourier shell correlation (FSC) cut-off of 0.5 (Rosenthal and Henderson, 2003). The refined 3D volumes were validated by EMAN2 validation methods.

#### Comparison of 2D Class Averages and 3D Volumes with Atomic Resolution Models

Atomic resolution models were converted to 40 Å resolution volumes with the e2pdb2mrc.py and volume projections compared with 2D class averages at 10° increments using e2classsvsproj.py. Two python scripts (e2pdbs2mrcs.py and e2classesvsprojs_best_scores.py) were previously developed to automate these steps and rank order models based on best scoring projections for each class (Malaby et al., 2018). For comparison with 3D volumes, automated rigid body docking of models at a resolution of 40 Å was performed with ADP_EM (Garzón et al., 2007). Models and volumes were visualized in Chimera (Pettersen et al., 2004).

#### Liposomes

All lipids were obtained from Avanti Polar Lipids. Liposomes were prepared as described previously (Aizel et al., 2013, Stalder et al., 2011) in a buffer containing 50 mM HEPES pH 7.4 and 120 mM potassium acetate. All liposomes contained 37.9% phosphatidylcholine (PC), 20% phosphatidylethanolamine (PE), 20% phosphatidylserine (PS), 20% cholesterol, 0.1 % NBD-PE and 2% phosphatidylinositol-4,5-triphosphate (PIP_2_) and were extruded through a 0.2 μm filter (Whatman). For kinetics assays, NBD-PE was omitted and PC was adjusted to 38%.

#### Nucleotide Exchange Kinetics

Kinetics of activation of myristoylated Arf1 were monitored at 37°C by tryptophan fluorescence (emission/excitation wavelengths of 292/340 nm) in 50 mM HEPES pH 7.4, 120 mM potassium acetate, 1 mM MgCl_2_ and 1 mM DTT (HKM buffer). 100 μM of liposomes were incubated for 2 minutes at 37°C, before the addition of ARNO constructs at different concentrations and 0.4 μM myristoylated Arf1. Nucleotide exchange was initiated by addition of 150 μM GTP.

#### DLS Experiments

Dynamic Light Scattering (DLS) experiments were performed on a DynaPro NanoStarTM instrument (Wyatt Technology). 1 mM of liposomes were incubated without or with 3 μM of the indicated protein at room temperature prior to analysis by DLS as described previously (Benabdi et al., 2017).

#### HDX-MS Experiments

ARNO_FL_ was diluted in HKM buffer to 1 μM and incubated for 10 min with or without 100 μM of liposomes prior addition of D_2_O. Deuterium exchange reactions were initiated by diluting the protein in D_2_O (99.8% D_2_O ACROS, Sigma, UK) in 25 mM HEPES pH 7.5, 125 mM potassium acetate, 1 mM TCEP to give a final D_2_O percentage of ∼95%. Deuterium labelling was carried out at 23°C (unless otherwise stated) at five time points: 0.3 (3 seconds on ice), 3, 30, 300 and 3000 seconds. The labelling reaction was quenched by the addition of chilled 2.4% v/v formic acid in 2M guanidinium hydrochloride and immediately frozen in liquid nitrogen. Samples were stored at -80°C prior to analysis. Each experiment was performed in triplicate.

The quenched protein samples were rapidly thawed and subjected to proteolytic cleavage with pepsin followed by reversed phase HPLC separation. Briefly, the protein was passed through an Enzymate BEH immobilized pepsin column, 2.1 x 30 mm, 5 μm (Waters, UK) at 200 μL/min for 2 min, the peptic peptides were trapped and desalted on a 2.1 x 5 mm C18 trap column (Acquity BEH C18 Van-guard pre-column, 1.7 μm, Waters, UK). Trapped peptides were subsequently eluted over 11 min using a 3-43% gradient of acetonitrile in 0.1% v/v formic acid at 40 μL/min. Peptides were separated on a reverse phase column (Acquity UPLC BEH C18 column 1.7 μm, 100 mm x 1 mm (Waters, UK) and detected on a SYNAPT G2-Si HDMS mass spectrometer (Waters, UK) over a m/z of 300 to 2000, with the standard electrospray ionization (ESI) source with lock mass calibration using [Glu1]-fibrino peptide B (50 fmol/μL). The mass spectrometer was operated at a source temperature of 80°C and a spray voltage of 2.6 kV. Spectra were collected in positive ion mode.

Peptide identification was performed by MSe (Silva et al., 2006) using an identical gradient of increasing acetonitrile in 0.1% v/v formic acid over 11 min. The resulting MSe data were analyzed using Protein Lynx Global Server software (Waters, UK) with an MS tolerance of 5 ppm.

Mass analysis of the peptide centroids was performed using the DynamX HDX data analysis software 3.0 (Waters, UK). Only peptides with a score > 6.4 were considered. The first round of analysis and identification was performed automatically by the DynamX software, however, all peptides (deuterated and non-deuterated) were manually verified at every time point for the correct charge state, presence of overlapping peptides, and correct retention time. Deuterium incorporation was not corrected for back-exchange and represents relative, rather than absolute changes in deuterium levels. Changes in H/D amide exchange in any peptide may be due to a single amide or a number of amides within that peptide.

#### Software Resources

Software available through the SBGRID Consortium was used for supported applications (Morin and Sliz, 2013).

### Quantification and Statistical Analysis

#### SAXS Profiles

Errors for SAXS profiles reconstructed by SVD-LC were estimated as the root mean squared deviation of the residuals for the fit with a maximum entropy model for the discretized inverse pair-distribution transformation

$$\text{I}\left(\text{q}\right)=4\text{π}\sum \text{P}\left(\text{r}\right)\;\text{sin}\left(\text{qr}\right)/\text{qr}$$

calculated on a real space grid of 1 Å over the range from 0.01 Å to an upper limit approximately 10-20% larger than D_max_. The informational entropy was calculated using a sine function on the interval 0-π radians as the prior distribution. The χ^2^ values reported here thus reflect the quality of fits with *ab initio*, rigid body and ensemble models compared to the nearly ideal best fit attainable with the maximum entropy inverse pair-distribution model. This approach for estimating errors avoids non-trivial and likely inaccurate error propagation associated with SVD-LC reconstruction of SAXS profiles.

#### Comparison with Class Averages and Volumes

Correlation coefficients and scoring functions for comparison of 2D class averages and 3D volumes with projections and volumes derived from atomic coordinates are presented as calculated by the software applications described in the Method Details and references therein.

#### Nucleotide Exchange Kinetics

All experiments were performed in duplicate, and means of two independent experiments are given ± the standard deviation (s.d.). k_obs_ were determined from monoexponential fits and k_cat_/k_M_ were calculated by linear regression of kobs values as a function of GEF concentration as described in (Aizel et al., 2013).

### Data and Code Availability

#### Data and Model Depositions

SAXS profiles, P(r) distributions, fits and models have been deposited with the Small Angle Scattering Biological Data Bank (Valentini et al., 2015) under the accession codes SASBDB: SASDEV9 (Cytohesin-2; ARF nucleotide-binding site opener, ARNO truncation mutant), SASBDB: SASDEW9 (Cytohesin-2; ARF nucleotide-binding site opener, ARNO), SASBDB: SASDG64 (Grp1 14-399 + IP4 SAXS with DAMMIF and GASBOR models), SASBDB: SASDG94 (Grp1 14-399 + IP4 SAXS with antiparallel CORAL and MultiFoXS models), SASBDB: SASDGA4 (Grp1 14-399 + IP4 SAXS with parallel CORAL and MultiFoXS models), SASBDB: SASDG74 (Grp1 14-390 + IP4 SAXS with DAMMIF and GASBOR models), SASBDB: SASDGB4 (Grp1 14-390 + IP4 SAXS with antiparallel CORAL and MultiFoXS models), SASBDB: SASDGC4 (Grp1 14-390 + IP4 SAXS with parallel CORAL and MultiFoXS models), SASBDB: SASDG84 (ARNO 2-400 + IP4 SAXS with DAMMIF, GASBOR and antiparallel CORAL models). EM envelopes have been deposited with the EM Data Bank (Lawson et al., 2016) under the accession codes EMDB: EMD-20628 (Grp1 14-399 + IP4 NS-EM Volume 1 with best antiparallel model) and EMDB: EMD-20629 (Grp1 14-399 + IP4 NS-EM Volume 2 with best antiparallel model). The best-fitting MultiFoXS models selected by ADP_EM have been deposited with the Protein Data Bank (Berman et al., 2000) under the accession codes PDB: 6U3E (Grp1 14-399 + IP4 NS-EM Volume 1 with best antiparallel model) and PDB: 6U3G (Grp1 14-399 + IP4 NS-EM Volume 2 with best antiparallel model). Accession codes are also included in Key Resources Table. Other data and models are available on request to the Lead Contact.

#### Software

The Mac OSX application DELA and associated Python scripts for processing and analysis of SEC-SAXS data sets and SAXS profiles have been described previously (Malaby et al., 2015). Python scripts (.py), bash shell scripts (.sh), and "pipelines" (_pipeline.txt) for SAXS and EM analyses described below can be downloaded as a zip file (Data S1), which also includes the application bundle and associated Python scripts for DELA. This version of DELA supports calculation of MEM distributions using model profiles derived from MultiFoXS pools. Although the scripts and pipelines are distributed as Open Source (https://opensource.org), the command line tools, programs or source code executed by these automation scripts are subject to the licensing terms of the relevant packages.

#### Shell Scripts

##### calculate_extract_rg.sh

Automates calculation and extraction of Rg values using the IMP program rg.

##### dammif.sh

Automates generation of ab initio bead models with DAMMIF, systematic pairwise alignment and selection with DAMSEL, alignment against the most representative bead model with DAMSUP, 'averaging' with DAMAVER, filtering with DAMFILT, and generation of an input file for DAMMIN with DAMSTART.

##### extract_models.sh

Automates extraction of individual models from multi model pdb files.

##### foxs.sh

Automates calculation of SAXS profiles using the command line version FoXS. Can be run in parallel batches.

##### foxs_component_summation_resample.sh

Automates summation of FoXS partial profiles and resampling to match data q values.

##### gasbor.sh

Equivalent to dammif.sh except that generation of ab initio bead models is done with GASBOR.

#### Python Scripts

##### e2pdbs2mrcs.py

Automates generation of volumes from atomic coordinates using the EMAN2 python script e2pdb2mrc.py. Can be run in parallel batches.

##### e2classesvsprojs.py

Automates comparison of class averages with volume projections using the EMAN2 python script e2classvsproj.py. Can be run in parallel batches.

##### e2classesvsprojs_best_scores.py

Identifies the best score and volume projection for each class average as well as the overall best score and volume projection for all class averages using the output of e2classesvsprojs.py.

##### e2classesvsprojs_extract_best.py

Extracts the best scoring coordinate files and corresponding image stacks using the output of e2classesvsprojs_best_scores.py.

##### e2classesvsprojs_generate_best_list.py

Generates a list of the images for the best scoring volume projection versus class average comparisons using the output of e2classesvsprojs_best_scores.py. The resulting list in "fast LST format" can be used as input for compilation of the images into an image stack in EMAN2.

##### extract_rg.py

Extracts Rg values embedded in a text file containing output generated by the IMP program rg.

##### filenames_rg.py

Combines filenames from one file with Rg values from another.

##### foxs_component_summation.py

Sums FoXS partial profiles using c1 and c2 constants from MultiFoXS.

##### foxs_resample.py

Resamples a FoXS profile to match q values from a reference profile using linear interpolation.

##### histogram_fractions.py

Generates a histogram of values with corresponding fractions after sorting in ascending order.

##### histogram.py

Generates a histogram of values after sorting in ascending order.

##### multifoxs_filenames.py

Generates a file containing the filenames for input to the command line version of multi_foxs.

#### Pipelines

The following "pipelines" are intended to illustrate the sequence of command line tools and scripts. Although they can be converted to a fully automated shell script if desired, we prefer to run the instructions individually to allow the output at each step to be monitored for quality control.

##### e2classesvsprojs_pipeline.txt

Example "pipeline" illustrating sequence of command line instructions used for comparison of 2D class averages with volume projections calculated from a pool of models generated by RRT_SAMPLE.

##### multifoxs_pipeline.txt

Example "pipeline" illustrating the sequence of command line instructions used for Multi_FoXS model generation, profile calculation and analysis with the IMP command line tools RRT_SAMPLE, foxs, and multi_foxs.

##### multifoxs_mem_pipeline.txt

Example "pipeline" illustrating the sequence of command line instructions used to prepare Multi_FoXS output for MEM in DELA. This "pipeline" requires partial profiles from FoXS (with -p option) and uses constants (c1 and c2) from MultiFoXS. The required partial profiles and constant values are available after the multifoxs_pipeline.txt "pipeline" completes.

## Supplemental Information

Document S1. Figures S1–S9 and Tables S1 and S2

Table S3. HDX-MS Data Table, Related to Figure 8

Data S1. Software, Related to the STAR Methods

Document S2. Article plus Supplemental Information
