# Supplementary material for: Structural Organization and Dynamics of Homodimeric Cytohesin Family Arf GTPase Exchange Factors in Solution and on Membranes
Source: Structure. 2019 Dec 3;27(12):1782–1797.e7. doi: 10.1016/j.str.2019.09.007 (PMC6948192; doi:10.1016/j.str.2019.09.007)
Supplement: Document S1. Figures S1–S9 and Tables S1 and S2 [file mmc1.pdf]

Structure, Volume *27*

Supplemental Information

Structural Organization and Dynamics of Homodimeric Cytohesin Family Arf GTPase Exchange Factors in Solution and on Membranes

Sanchaita Das, Andrew W. Malaby, Agata Nawrotek, Wenhua Zhang, Mahel Zeghouf, Sarah Maslen, Mark Skehel, Srinivas Chakravarthy, Thomas C. Irving, Osman Bilsel, Jacqueline Cherfils, and David G. Lambright

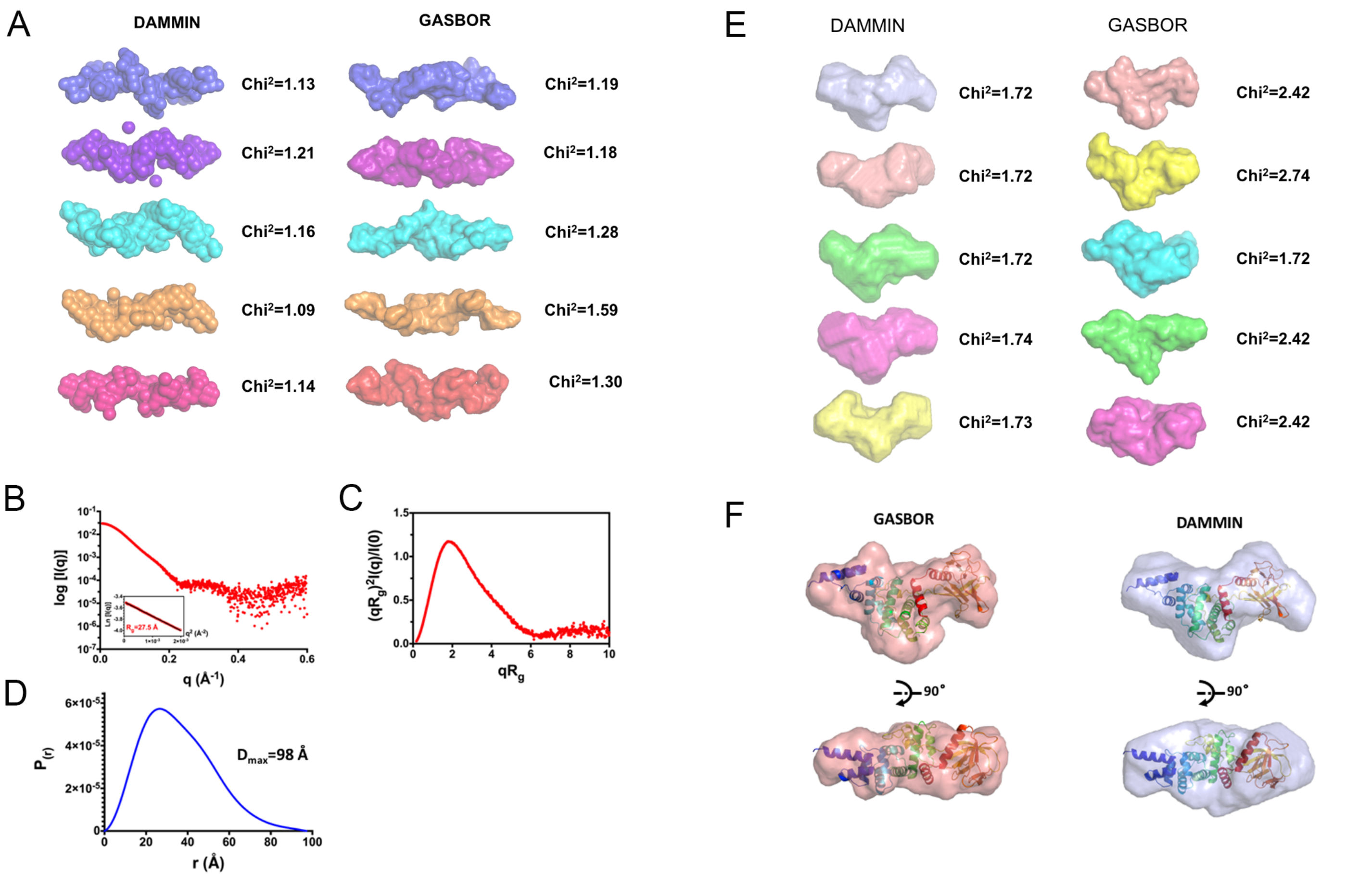

Figure S1. SAXS analyses of ARNOΔ^{Nt} and ARNO$_{FL}$. Related to Figure 1

(A) SAXS profile of ARNOΔ^{Nt}. The insert shows the Guinier plot ($R_g \times q_{max}$ = 1.22). (B) The dimensionless Kratky plot analysis shows a fully globular protein. (C) The P(r) plot analysis gives an estimated D_{max} of 98 Å. (D) Envelopes calculated with GASBOR and DAMMIN. (E) Fit of autoinhibited GRP1 structure in a representative ARNOΔ^{Nt} envelope. (F) Additional envelopes of ARNO$_{FL}$ calculated with GASBOR and DAMMIN. The Chi2 values are indicated.

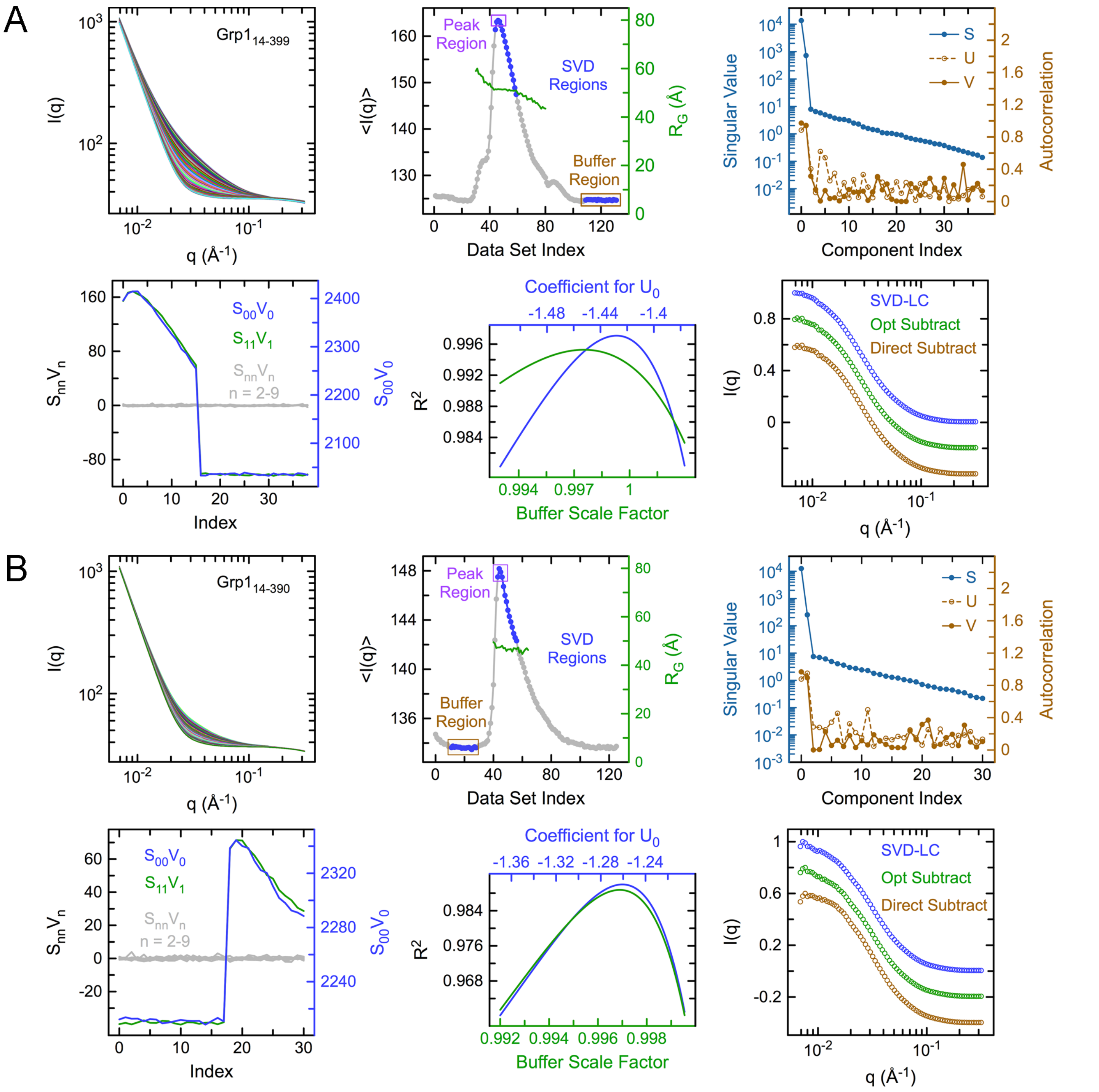

Figure S2. SEC-SAXS Analysis and Reconstruction of Protein Scattering for Dimeric Grp1 Constructs with IP_4. Related to Figure 1

(A) Autoinhibited $Grp1_{14\text{-}399}$. (B) Fully active $Grp1_{14\text{-}390}$. (A and B) *Upper left*, SAXS profiles after radial averaging and normalization. *Upper middle*, mean scattering during elution with regions used for buffer subtraction or SVD indicated. Also shown are R_G values from Guinier analysis of buffer subtracted scattering profiles. *Upper right*, singular values (diagonal elements of the diagonal matrix **S**) and autocorrelations for the rank ordered components (columns of the matrix **U**) and corresponding variable coefficients (columns of the symmetric matrix **V**) after singular value decomposition (SVD; $\mathbf{A} = \mathbf{U} \bullet \mathbf{S} \bullet \mathbf{V}^T$) of the SAXS profiles (columns of the matrix **A**). *Lower left*, Singular value-weighted columns of **V**, where n is the component index and the x-axis corresponds to the index of the SAXS data sets used for SVD. *Lower middle*, analysis of linearity in the Guinier region as a function of the buffer scale factor for optimized buffer subtraction or the U_0 coefficient for linear combination of the two most significant SVD components (SVD-LC). *Lower right*, comparison of methods for reconstruction of the protein scattering profile as $c * U_0 + U_1$ (SVD-LC), <I(q) peak region> - c * <I(q) buffer region(s)> (Opt Subtract), or <I(q) peak region> - <I(q) buffer region(s)> (Direct Subtract).

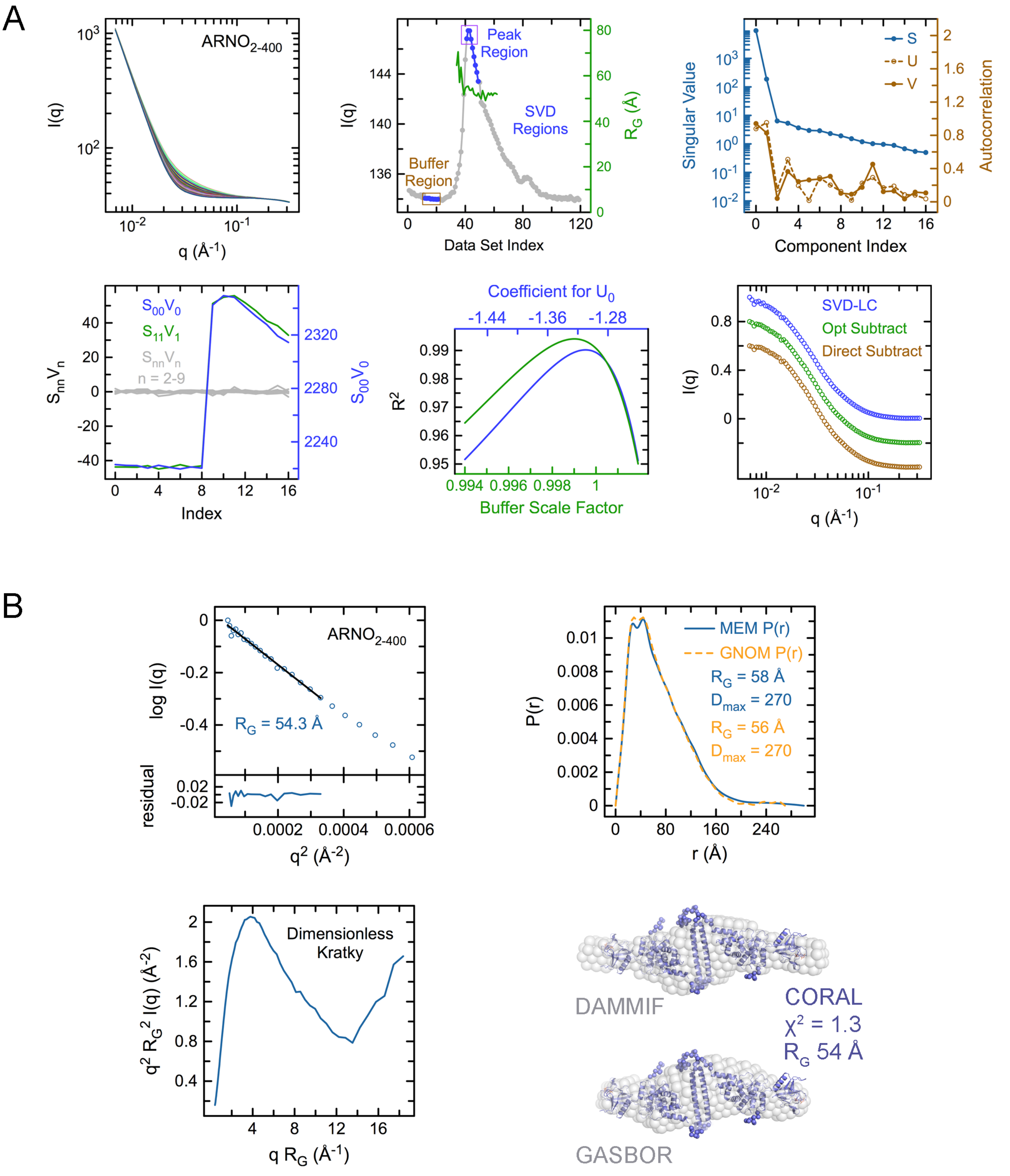

Figure S3. SEC-SAXS Analysis for Homodimeric ARNO$_{2\text{-}400}$ with IP$_4$. Related to Figure 2
(A) SEC-SAXS and reconstruction of protein scattering profiles for homodimeric ARNO$_{2\text{-}400}$ bound to IP$_4$. See Figure S2 legend for description of panels. (B) Basic SAXS analyses and *ab initio* models. *Upper left*, Guinier plot and fit. *Upper right*, MEM and GNOM P(r) distributions. *Lower left*, dimensionless Kratky plot. *Lower right*, *ab initio* models calculated with DAMMIF and GASBOR. Also shown is the rigid body CORAL model based on the Grp1 autoinhibited core and antiparallel coiled coil.

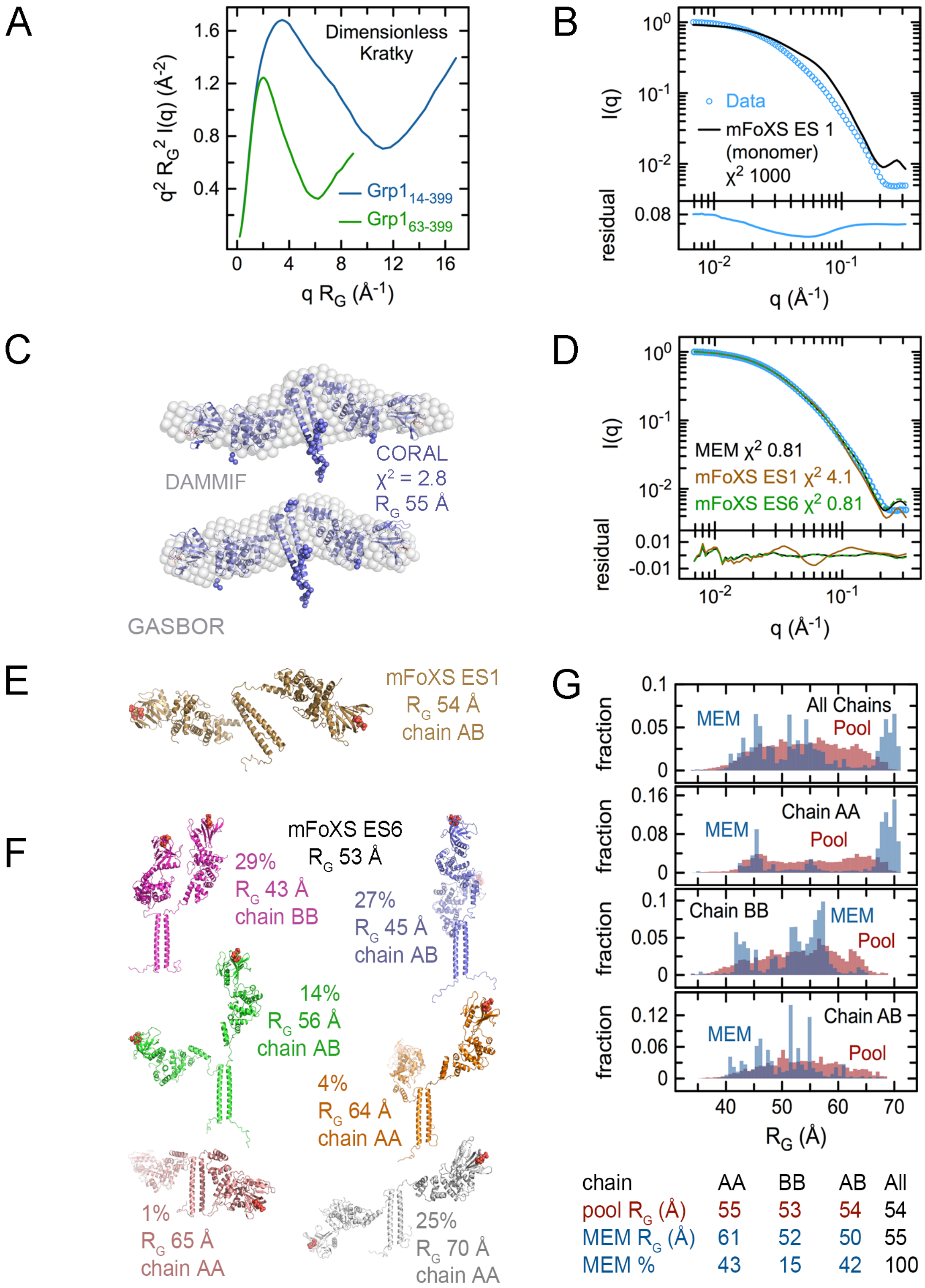

Figure S4. SAXS Analyses of Autoinhibited Grp1 Dimers. Related to Figure 2

(A) Dimensionless Kratky plot for autoinhibited Grp1 constructs with or without the CC. (B) Experimental SAXS profile and best-fitting single model MultiFoXS profile for a monomer pool. No multistate ensembles were identified. (C) *Ab initio* models calculated with DAMMIF or GASBOR and aligned with the rigid body CORAL model for the parallel CC dimer. (D) Comparison of the experimental SAXS profile with the profiles for the best-fitting single model (ES1) and multiple model (ES6) MultiFoXS ensembles as well as the all model MEM distribution for the parallel CC dimer. (E) Best-fitting single state MultiFoXS model (ES1) for the parallel CC dimer. (F) Models for the best-fitting MultiFoXS ensemble (ES6) for the parallel CC dimer with percentages and R_G values. The overall R_G for the ensemble was calculated as the fraction-weighted mean of the individual R_G values. (G) Fraction-weighted histograms of R_G values for the MEM distribution and pool for the parallel CC dimer. Fraction-weighted mean R_G values and percentages are tabulated below.

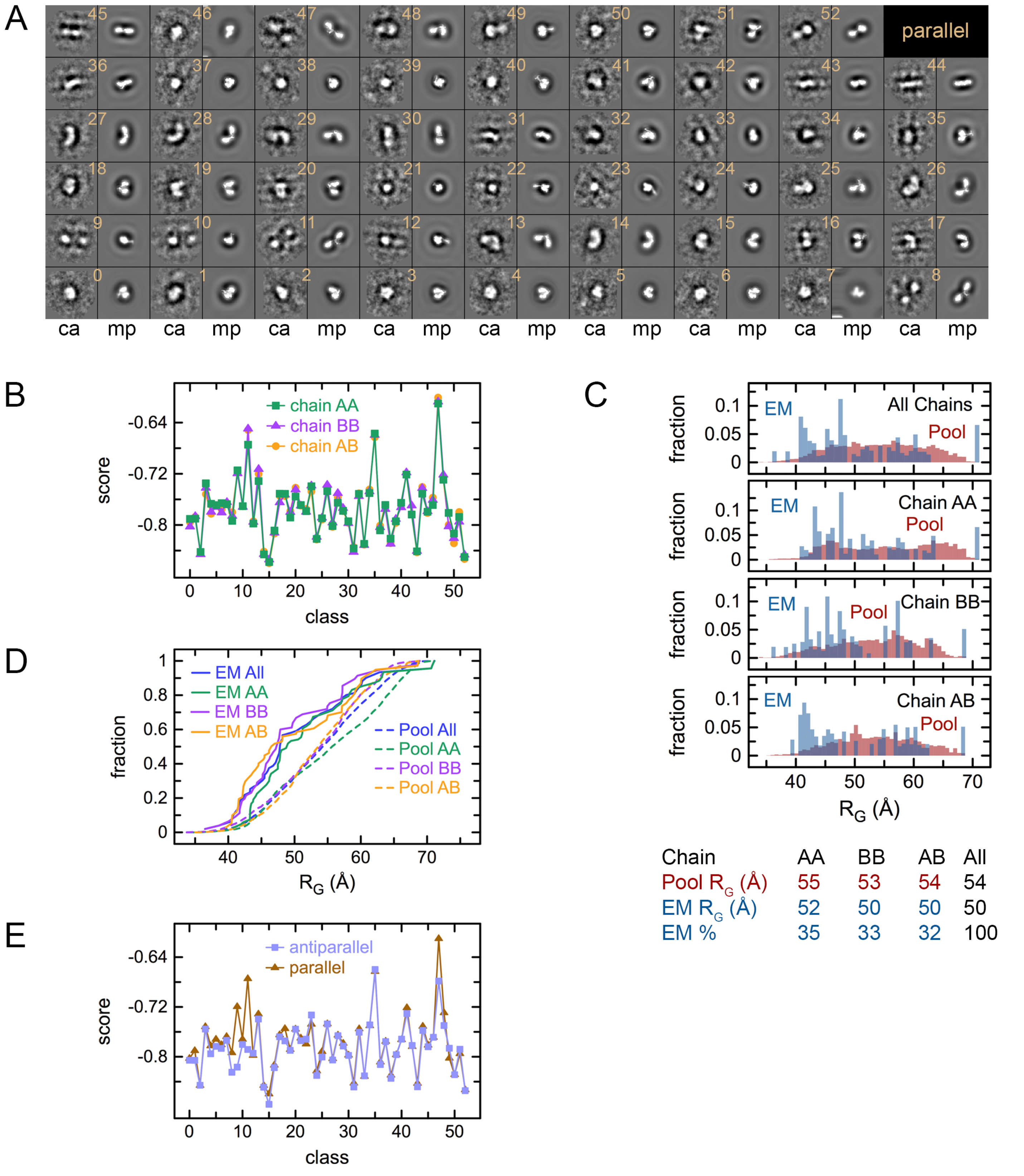

Figure S5. Projection Matching Analysis with Parallel MultiFoXS Models. Related to Figure 4

(A) Class averages compared with the best scoring 3D volume projection from the models in the MultiFoXS pools. (B) Scores for comparison of class averages with the best scoring 3D volume projection from the parallel models in the MultiFoXS pools. (C) Histograms of R_G values for the best scoring model for each class average.(D) Cumulative distribution of R_G values for the best scoring model for each class average.
(E) Comparison of scores for parallel and parallel CC topologies.

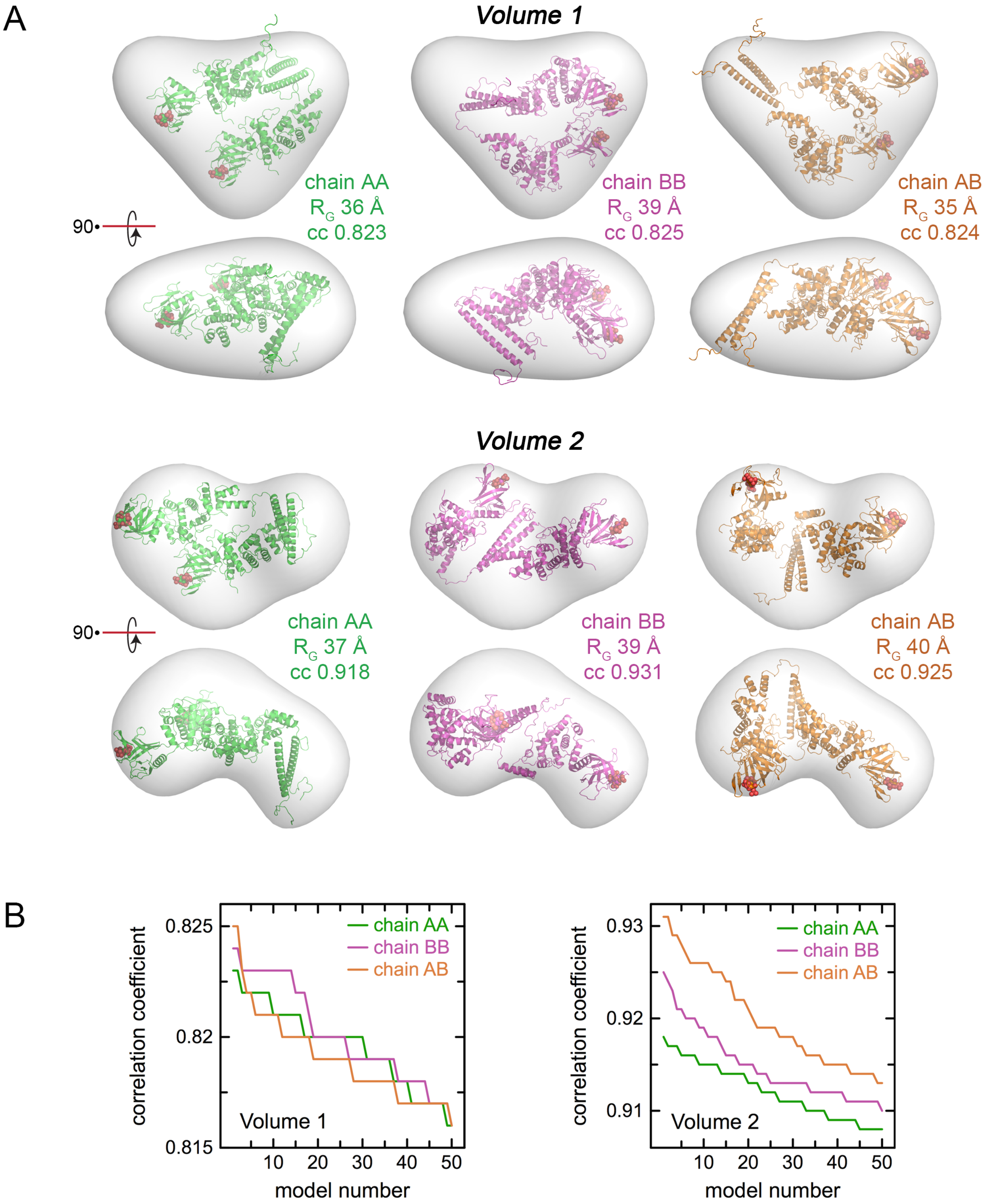

Figure S6. 3D Reconstructions and Best Fitting Parallel MultiFoXS Models. Related to Figure 5
(A) Comparison of the best fitting MultiFoXS models with the volumes from 3D reconstruction and refinement of the class sets indicated in Figure 4C. (B) Correlation coefficients for the 50 best-fitting models from the comparison of each volume with the MultiFoXS pools.

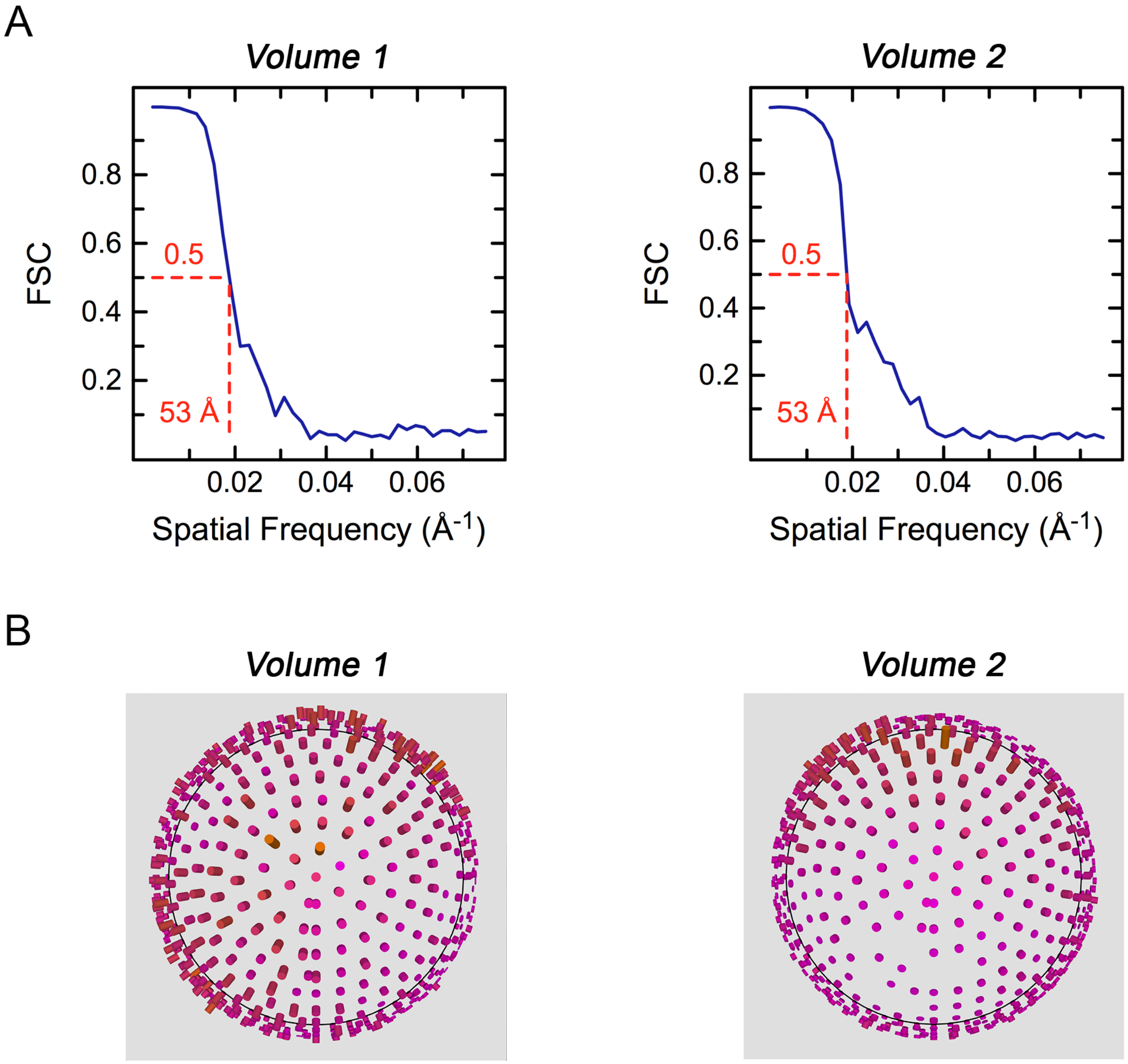

Figure S7. Assessment of 3D Reconstructions. Related to Figure 5
(A) Resolution estimation for 3D reconstructions. (B) Euler angle distribution of particles used to for 3D reconstructions.

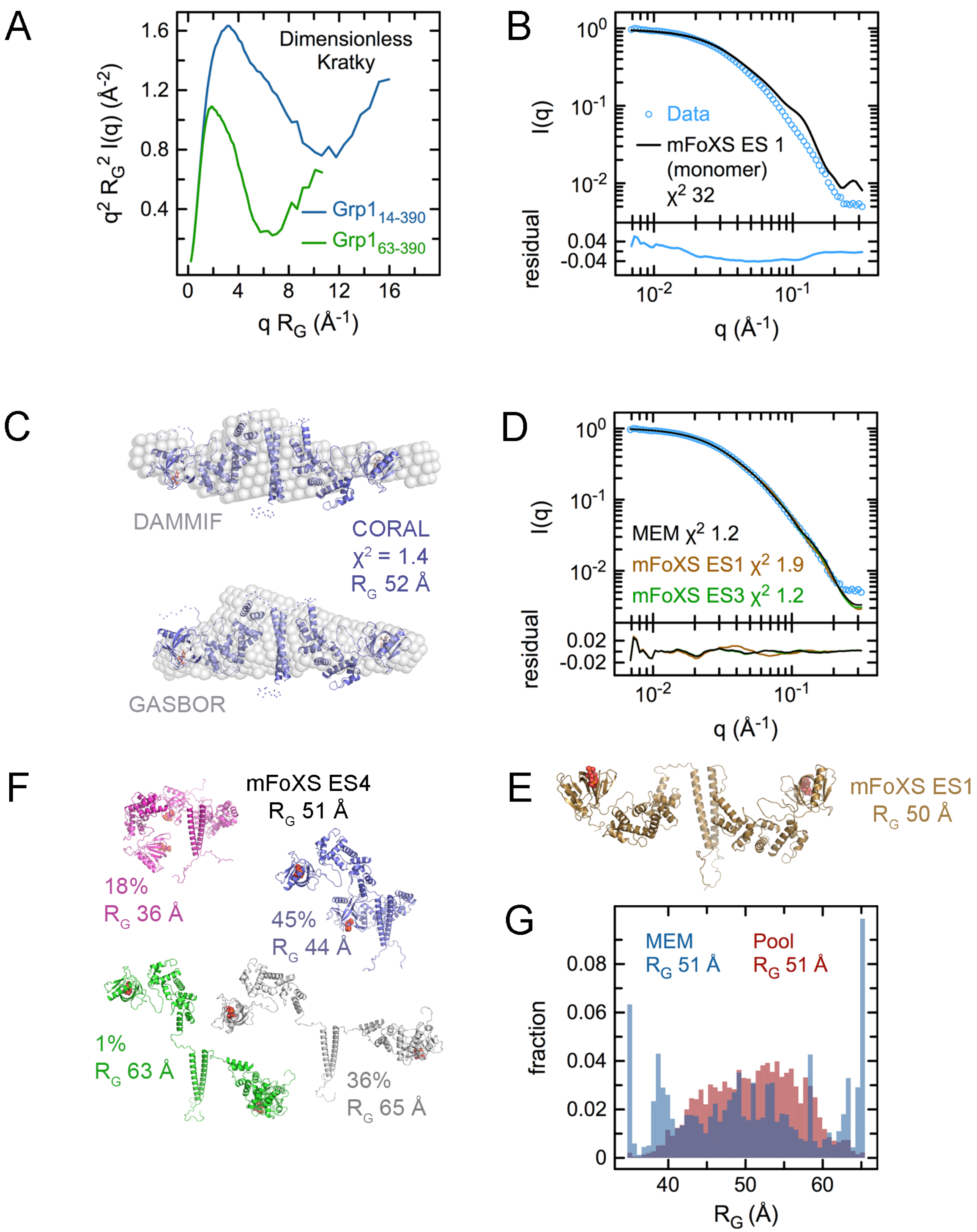

Figure S8. SAXS Analyses of Fully Active Grp1$_{13\text{-}390}$ Dimers. Related to Figure 6
(A) Dimensionless Kratky plot for autoinhibited Grp1 constructs with and without the CC. (B) Comparison of the experimental SAXS profile with the best-fitting single model MultiFoXS profile for a monomer pool. No multistate ensembles were identified. (C) *Ab initio* models calculated with DAMMIF or GASBOR and aligned with the rigid body CORAL model for the parallel CC dimer. (D) Comparison of the experimental SAXS profile with the profiles for the best-fitting single model (ES1) and multiple model (ES4) MultiFoXS ensembles as well as the all model MEM distribution for the parallel CC dimer. (E) Best-fitting single state MultiFoXS model (ES1) for the parallel CC dimer. (F) Models for the best-fitting MultiFoXS ensemble (ES4) for the parallel CC dimer with percentages and R_G values. The overall R_G for the ensemble was calculated as the fraction-weighted mean of the individuals R_G values. (G) Fraction-weighted histograms of R_G values for the MEM distribution and pool for the parallel CC dimer. Fraction-weighted mean R_G values and percent fractions are tabulated below.

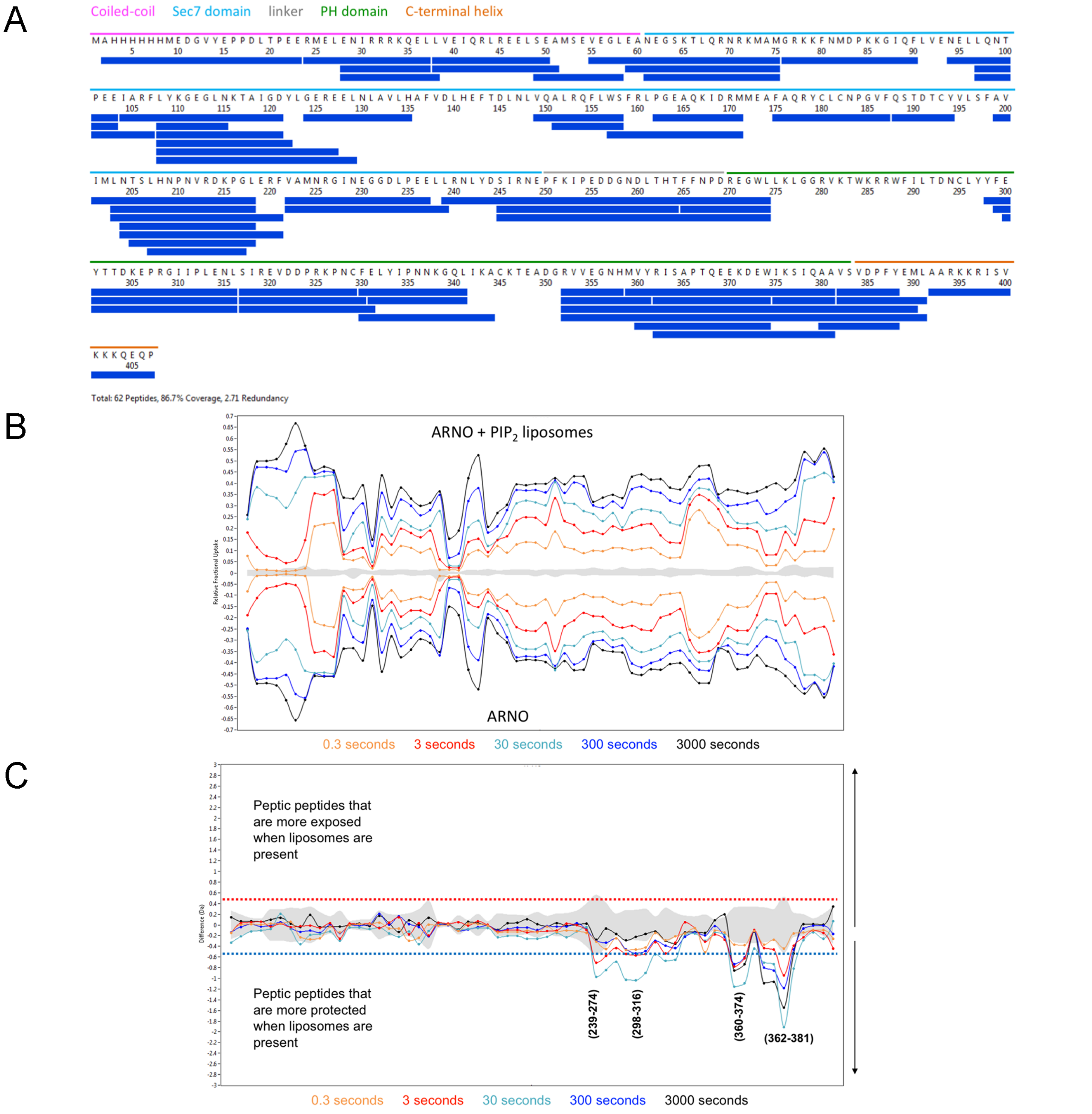

Figure S9. HDX-MS analysis of the ARNO$_{FL}$ interaction with PIP$_2$ containing liposomes. Related to Figure 8

(A) Peptic peptide coverage of ARNO$_{FL}$ with domains indicated above the amino acid sequence. (B) Butterfly or mirror plot displayed in DynamX 3.0 (Waters, UK) showing the incorporation of deuterium at successive time points indicated by the different colors. Relative fractional uptake is shown on the y-axis,without correction for back-exchange, while the experimental error is reported as a grey bar on the x-axis. (C) Difference plot obtained by subtracting the incorporation of deuterium in the absence of PIP$_2$ containing liposomes to the one obtained in the presence of PIP$_2$ containing liposomes. Experimental error is reported in grey, while different colors represent different D_2O incubation times as above. More exposed and protected regions are located, respectively, on the higher and lower part of the graph. Peptide aa numbers from highly protected regions of ARNO$_{FL}$ are shown. ±0.5 Dalton difference is indicated with red/blue dotted lines, which represent a 98% confidence limit, so that peptides above or below these lines can be considered significantly changed.

Table S1. Statistics of SEC-SAXS analysis of ARNOΔ^{Nt} and ARNO^{FL}. Related to Figure 1

		**ARNOΔ^{Nt}**		**ARNO^{FL}**	
Instrument		SOLEIL synchrotron SWING beamline		ESRF BM29	
Detector		PCCD170170 Aviex Detector		Pilatus 1M detector	
Beam geometry		Pinhole		Pinhole	
Wavelength (Å)		1.033		0.99	
q range ($Å^{-1}$)		0.01–0.600		0.0025-0.5	
Exposure time (s)		1.5 per frame		1	
Temperature (°C)		15		15	
**Structural Parameters**					
From Guinier fit	I_0 (cm^{-1})	0.03±2e-05		43.99±6.7e-02	
	R_g (Å)	27.46±0.16		47.9	
From $P_{(r)}$	I_0 (cm^{-1})	0.03±1.4e-05		45.01	
	Volume($Å^3$)	63000		14614	
	R_g (Å)	27.7		50.9	
	D_{max} (Å)	98		200	
**Molar Mass Determination**					
Molar mass (kDa) from sequence		39.9		93.2 (homodimer)	
Molar mass (kDa) from Qp		42		102	
Molecular mass (kDa) from MoW		40		89	
Molecular mass (kDa) from Vc		41		89	
**Model Evaluation**					
		Average Fit	Spatial Discrepancy	Average Fit	Spatial Discrepancy
GASBOR (n=5)		Chi^2=2.42±0.18	NSD=1.046±0.045	Chi^2=1.3±0.19	NSD=1.72±0.04
DAMMIN (n=5)		Chi^2=1.72±0.006	NSD=0.631±0.027	Chi^2=1.14±0.05	NSD=0.84±0.05

Table S2. Statistics of SEC-SAXS analysis of Grp1 and ARNO constructs with IP_4. Related to Figures 2 and 6

		**Grp1$_{14\text{-}399}$**	**Grp1$_{14\text{-}390}$**	**ARNO$_{2\text{-}400}$**
Instrument		APS BioCAT 18-ID	APS BioCAT 18-ID	APS BioCAT 18-ID
Detector		MAR 165 CCD	MAR 165 CCD	MAR 165 CCD
Beam geometry		Pinhole	Pinhole	Pinhole
Wavelength (Å)		1.033	1.033	1.033
q range (Å^{-1})		0.0062–0.333	0.0062-0.333	0.0062-0.333
Exposure time (s)		1 per frame	1 per frame	1 per frame
Temperature (°C)		20	20	20
**Structural Parameters**				
From Guinier fit	I_0 (cm^{-1})	1.05±1e-3	1.02±2e-3	1.03±2e-3
	R_g (Å)	54.56±0.17	50.61±0.4	53.03±0.36
From $P_{(r)}$	I_0 (cm^{-1})	1.06±2e-3	1.03±5e-3	1.04±4e-3
	Volume(Å^3)	194000	168000	180000
	R_g (Å)	57.32±0.6	54.27±1.4	55.98±1.3
	D_{max} (Å)	260	257	270
**Molar Mass Determination**				
Molar mass (kDa) from sequence		93.6 (homodimer)	91.2 (homodimer)	95.2 (homodimer)
Molar mass (kDa) from Qp		135	118	127
Molecular mass (kDa) from MoW		97	86	95
Molecular mass (kDa) from Vc		86	76	82
Molecular mass (kDa) from Bayes Inference		94	86	91
**Model Evaluation**				

	Grp1$_{14\text{-}399}$ Average χ^2	Grp1$_{14\text{-}399}$ Normalized Spatial Discrepancy	Grp1$_{14\text{-}390}$ Average χ^2	Grp1$_{14\text{-}390}$ Normalized Spatial Discrepancy	ARNO$_{2\text{-}400}$ Average χ^2	ARNO$_{2\text{-}400}$ Normalized Spatial Discrepancy
GASBOR (n=100)	4.68±0.13	0.604±0.015	1.96±0.033	0.631±0.025	2.18±1.20	0.606±0.022
DAMMIF (n=100)	1.22±0.003	0.570±0.011	0.591±0.015	0.84±0.05	1.19±0.04	0.609±0.017
