# Supplementary material for: Structural Organization and Dynamics of Homodimeric Cytohesin Family Arf GTPase Exchange Factors in Solution and on Membranes
Source: Structure. 2019 Dec 3;27(12):1782–1797.e7. doi: 10.1016/j.str.2019.09.007 (PMC6948192; doi:10.1016/j.str.2019.09.007)
Supplement: Data S1. Software, Related to the STAR Methods [file mmc3.zip › Software/Readme.pdf]

Notes

The DELA application package is supported for OSX 10.9 or later.

Shell script were tested in bash on OSX 10.13.

Python scripts were tested with Python 2.7 in bash on OSX 10.13.

Command line help for Bash and Python scripts is available by typing the name of the script followed by -h or --help.

Although these scripts and pipelines are distributed as Open Source (https://opensource.org), the command line tools, programs or source code executed by these automation scripts are subject to the licensing terms of the relevant packages.

DELA

DELA is a document-based OSX application with built-in functionality including data processing, publication quality graphics, linear matrix and maximum entropy methods, local and global maximum likelihood fitting with model functions and reaction schemes, storage of data and derived results, and other capabilities. It is extensible via an embedded Python interpreter. SAXS Python scripts are included for processing and analysis of SAXS profiles and SEC-SAXS data sets. See SAXS_Tutorial.pdf for more information including installation. Documentation is also available from the help menu within the application. This version of DELA includes support for calculation of MEM distributions of scattering profiles from a pool of structural models.

Shell Scripts

calculate_extract_rg.sh
Automates calculation and extraction of Rg values using the IMP program rg.

dammif.sh
Automates generation of *ab initio* bead models with DAMMIF, systematic pairwise alignment and selection with DAMSEL, alignment against the most representative bead model with DAMSUP, 'averaging' with DAMAVER, filtering with DAMFILT, and generation of an input file for DAMMIN with DAMSTART.

extract_models.sh
Automates extraction of individual models from multi model pdb files.

foxs.sh
Automates calculation of SAXS profiles using the command line version FoXS. Can be run in parallel batches.

foxs_component_summation_resample.sh
Automates summation of FoXS partial profiles and resampling to match data q values.

gasbor.sh
Equivalent to dammif.sh except that generation of *ab initio* bead models is done with GASBOR.

Python Scripts

e2pdbs2mrcs.py
Automates generation of volumes from atomic coordinates using the EMAN2 python script e2pdb2mrc.py. Can be run in parallel batches.

e2classesvsprojs.py
Automates comparison of class averages with volume projections using the EMAN2 python script e2classvsproj.py. Can be run in parallel batches.

e2classesvsprojs_best_scores.py
Identifies the best score and volume projection for each class average as well as the overall best score and volume projection for all class averages using the output of e2classesvsprojs.py.

e2classesvsprojs_extract_best.py
Extracts the best scoring coordinate files and corresponding image stacks using the output of e2classesvsprojs_best_scores.py.

e2classesvsprojs_generate_best_list.py
Generates a list of the images for the best scoring volume projection versus class average comparisons using the output of e2classesvsprojs_best_scores.py. The resulting list in "fast LST format" can be used as input for compilation of the images into an image stack in EMAN2.

extract_rg.py
Extracts Rg values embedded in a text file containing output generated by the IMP program rg.

filenames_rg.py
Combines filenames from one file with Rg values from another.

foxs_component_summation.py
Sums FoXS partial profiles using c1 and c2 constants from MultiFoXS.

foxs_resample.py

Resamples FoXS profile to match q values from reference profile using linear interpolation.

histogram_fractions.py
Generates a histogram of values with corresponding fractions after sorting in ascending order.

histogram.py
Generates a histogram of values after sorting in ascending order.

multifoxs_filenames.py
Generates a file containing the filenames for input to the command line version of multi_foxs.

Pipelines

The following "pipelines" are intended to illustrate the sequence of command line tools and scripts. Although they can be converted to a fully automated shell script if desired, we prefer to run the instructions individually to allow the output at each step to be monitored for quality control.

e2classesvsprojs_pipeline.txt
Example "pipeline" illustrating sequence of command line instructions used for comparison of 2D class averages with volume projections calculated from a pool of models generated by RRT_SAMPLE.

multifoxs_pipeline.txt
Example "pipeline" illustrating the sequence of command line instructions used for Multi_FoXS model generation, profile calculation and analysis with the IMP command line tools RRT_SAMPLE, foxs, and multi_foxs.

multifoxs_mem_pipeline.txt
Example "pipeline" illustrating the sequence of command line instructions used to prepare Multi_FoXS output for MEM in DELA. This "pipeline" requires partial profiles from FoXS (with -p option) and uses constants (c1 and c2) from MultiFoXS. The required partial profiles and constant values are available after the multifoxs_pipeline.txt "pipeline" completes.
