# Supplementary material for: Structural Organization and Dynamics of Homodimeric Cytohesin Family Arf GTPase Exchange Factors in Solution and on Membranes
Source: Structure. 2019 Dec 3;27(12):1782–1797.e7. doi: 10.1016/j.str.2019.09.007 (PMC6948192; doi:10.1016/j.str.2019.09.007)
Supplement: Data S1. Software, Related to the STAR Methods [file mmc3.zip › Software/Dela App Scripts/SAXS_Tutorial.pdf]

SAXS data processing with DELA

What's in this tutorial:

Essentials

Installation and setup

Basic manipulations and tips

Step by step examples for SAXS and SEC-SAXS data processing and analyses combining built-in functionality with Python scripts

See the help pages in the application for additional info, including descriptions of SAXS Python scripts and available DELA Python modules.

DELA

DELA is a document-based Intel Mac OS X application

Tools are provided for data import/export, graphical visualization/analysis, processing, local and global maximum likelihood fitting, and other functions. Data can be imported/exported, documents saved/opened, and publication quality plots rendered in common image formats.

The application is extensible via an embedded Python interpreter

An extensive API with automatically defined modules facilitates data transfer and control of application functionality within Python scripts.

Data are stored in objects with arrays for x, y, error, and Boolean mask values

Every data object has a unique identifying ‘Label’ of the form Data#, where # is an integer automatically assigned when the data objected is created.

Data objects are organized in sheets and plots

Unlike typical spread sheets, data objects may be shared between different sheets. Data objects can be edited individually or collectively at the level of a sheet. Data, sheets and plots can be organized into custom ‘groups’ of related content.

A three button mouse with a scroll wheel is recommended but not essential

A right mouse button click, for example, is equivalent to a control-click.

DELA document window

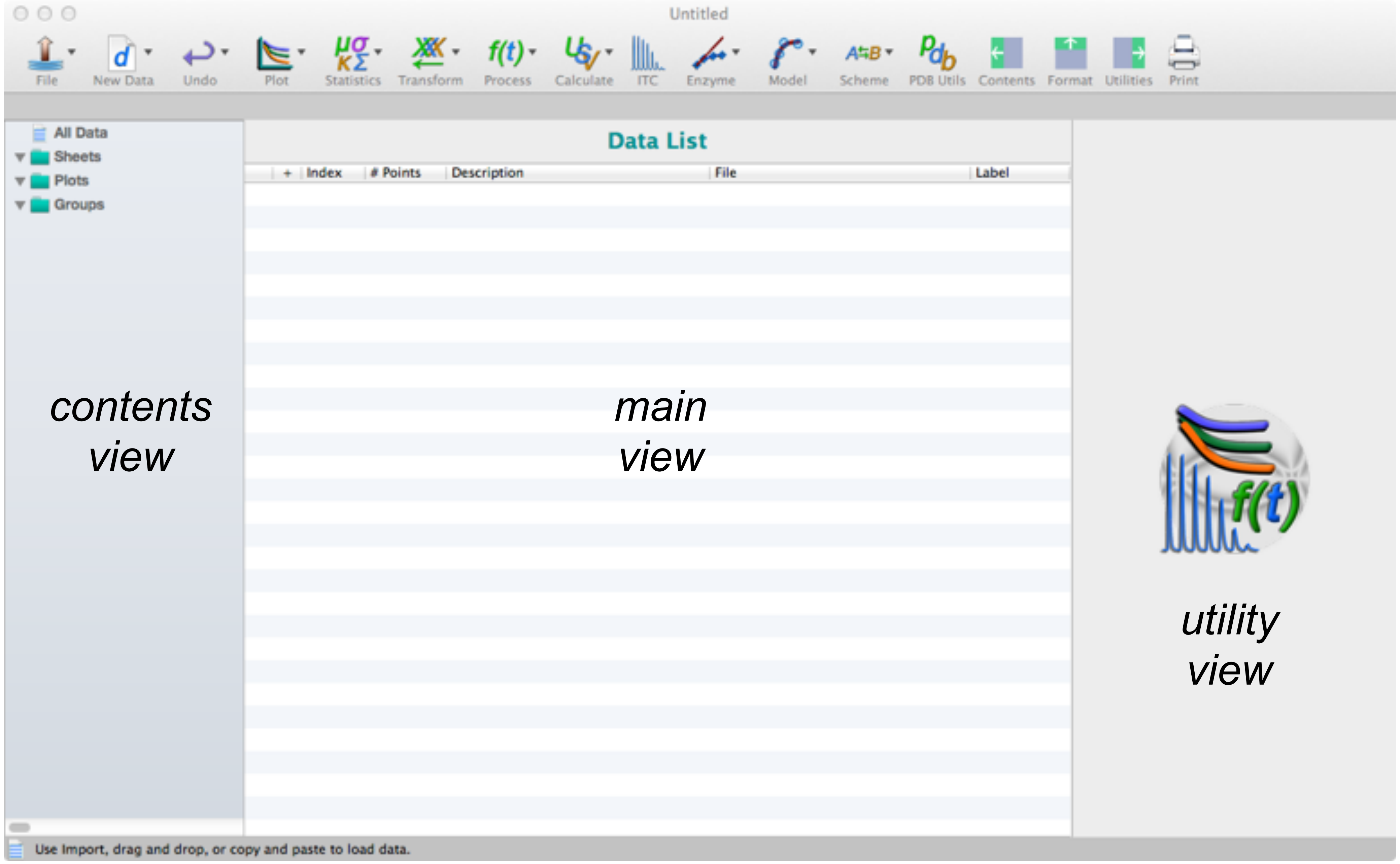

tool bar
format bar

info strip

Data, sheets, or plots are selected in the contents view

Selected items become the target(s) for actions including processing and plotting. The most recently selected item is displayed in the main view, and controls for active tools in the utility view. Clicking on disclosure triangles toggles item visibility in the contents. Clicking on a view may be required to activate it for keyboard input.

Installation and setup

1. System requirements: Intel Mac running OSX 10.7 or later.

Performance depends on CPU, memory, the graphics card etc. Available system memory is the only limitation on the size or number of data objects. For very large data sets, memory excessive usage can be reduced by disabling undo from the Undo menu in the document tool bar. Likewise, the drawing speed for plots can be substantially improved by reducing the line stroke width (e.g. to 0.5 or less) and/or using points as the symbol type.

2. Drag the DELA app to the Applications folder

3. Start DELA

When started for the first time by a new user, DELA will create a hidden .Dela directory in the user's home directory, where user-specific data are stored including imported script files and user created model functions or reaction schemes. Preference files will also be created in the user's <Home>/Library/Preferences directory.

4. Import the Python SAXS scripts

Scripts can be imported from the Interpret Script utility, which is activated by selecting Process ➞ Interpret Script in the document tool bar.

Interpret Script utility

select script *imported scripts can be selected here*

Optional path for script file

Optional path for log file

Save log info to a file

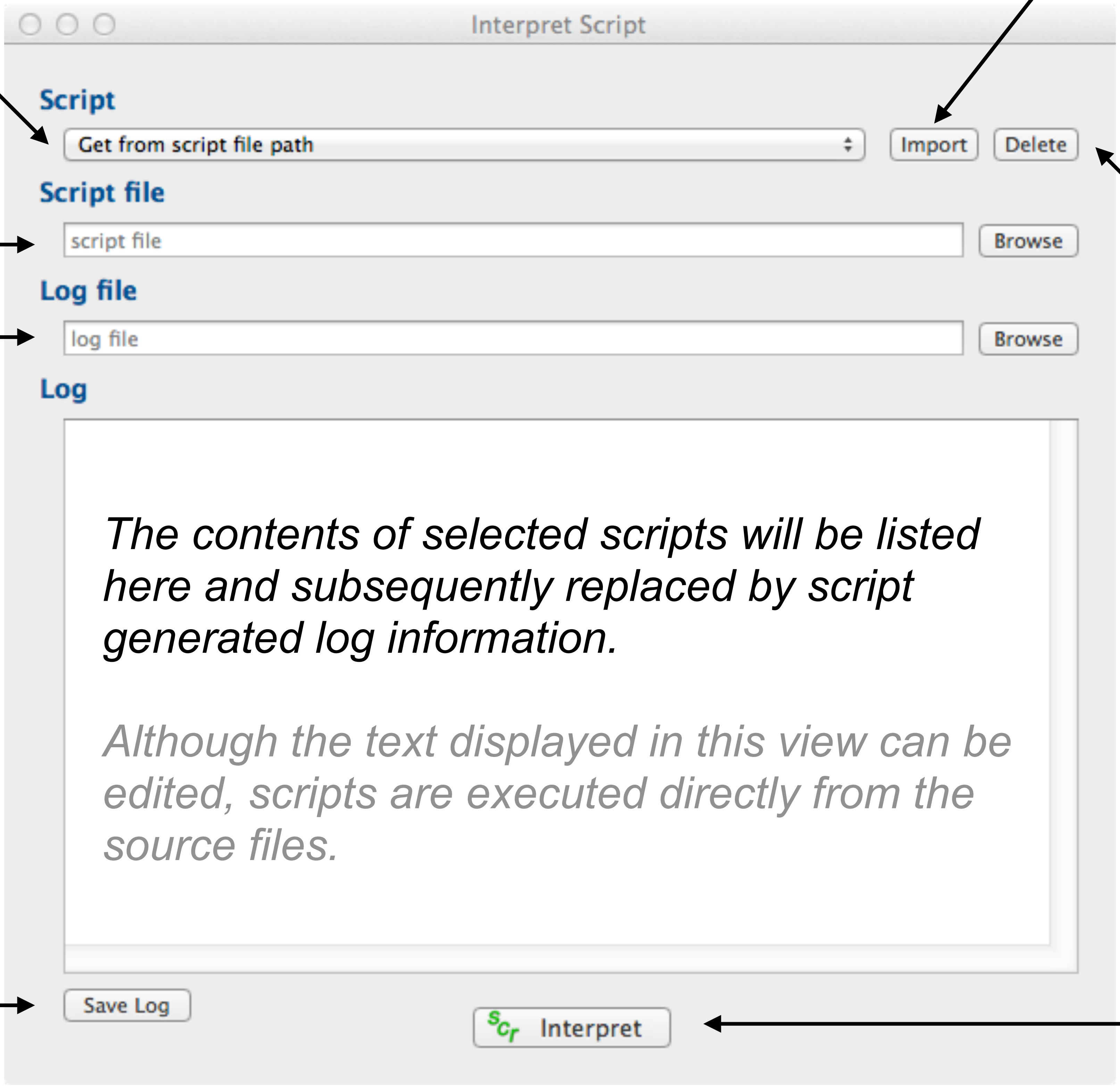

The contents of selected scripts will be listed here and subsequently replaced by script generated log information.

Although the text displayed in this view can be edited, scripts are executed directly from the source files.

import scripts

remove currently selected script from the import directory but not from other locations

interpret selected script

Import radially averaged SAXS data from files

1. Select and drag data files from a Finder window and drop them onto the main view of the document window – OR – select File → Import in the tool bar

2. Select X-ray Scattering in the Data Type menu and click Import

If the files are not BioCAT format, it may be necessary to adjust other settings. Optional data and/or column labels should be separated by a single tab. A new sheet with the data will appear in the contents view.

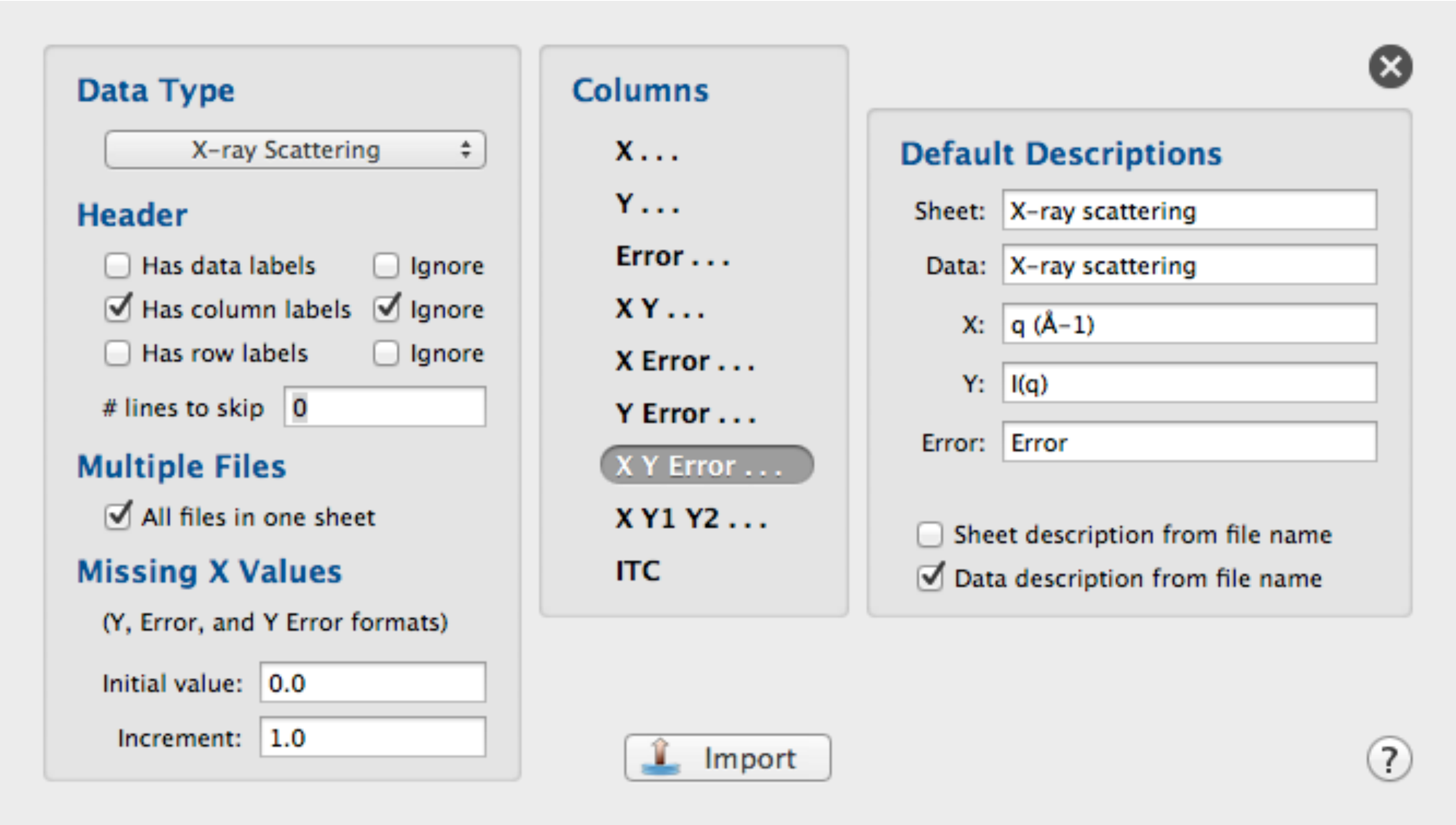

During import, a new sheet with the scattering is created

Toggle visibility of sheet contents

Nonvolatile data selection

Unicode sheet description

Data label

Click to view/edit Annotated Sheet Description

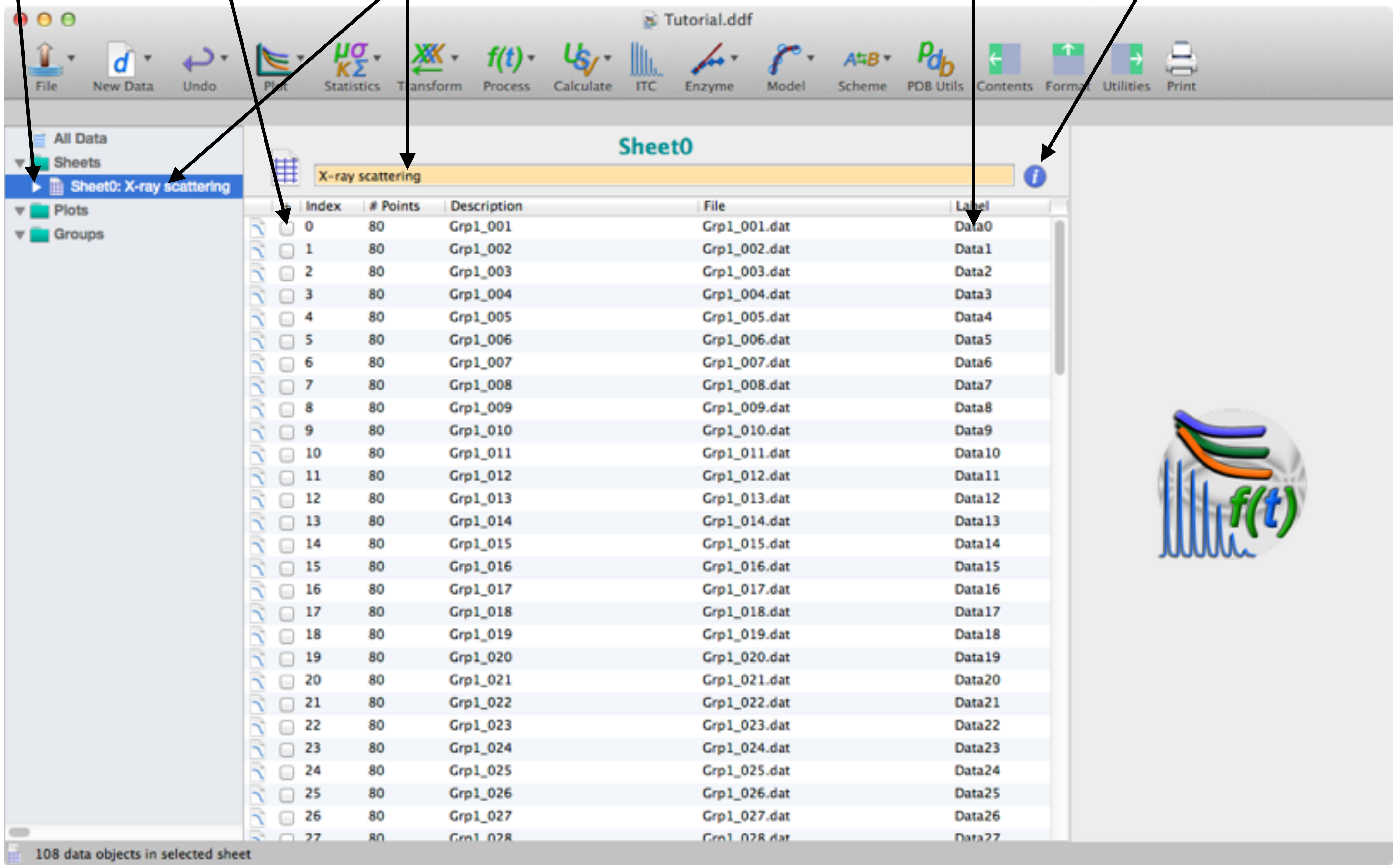

Plot scattering data

1. Click on the new sheet in the contents view to select it

2. Select Plot → New Plot in the tool bar and click Plot

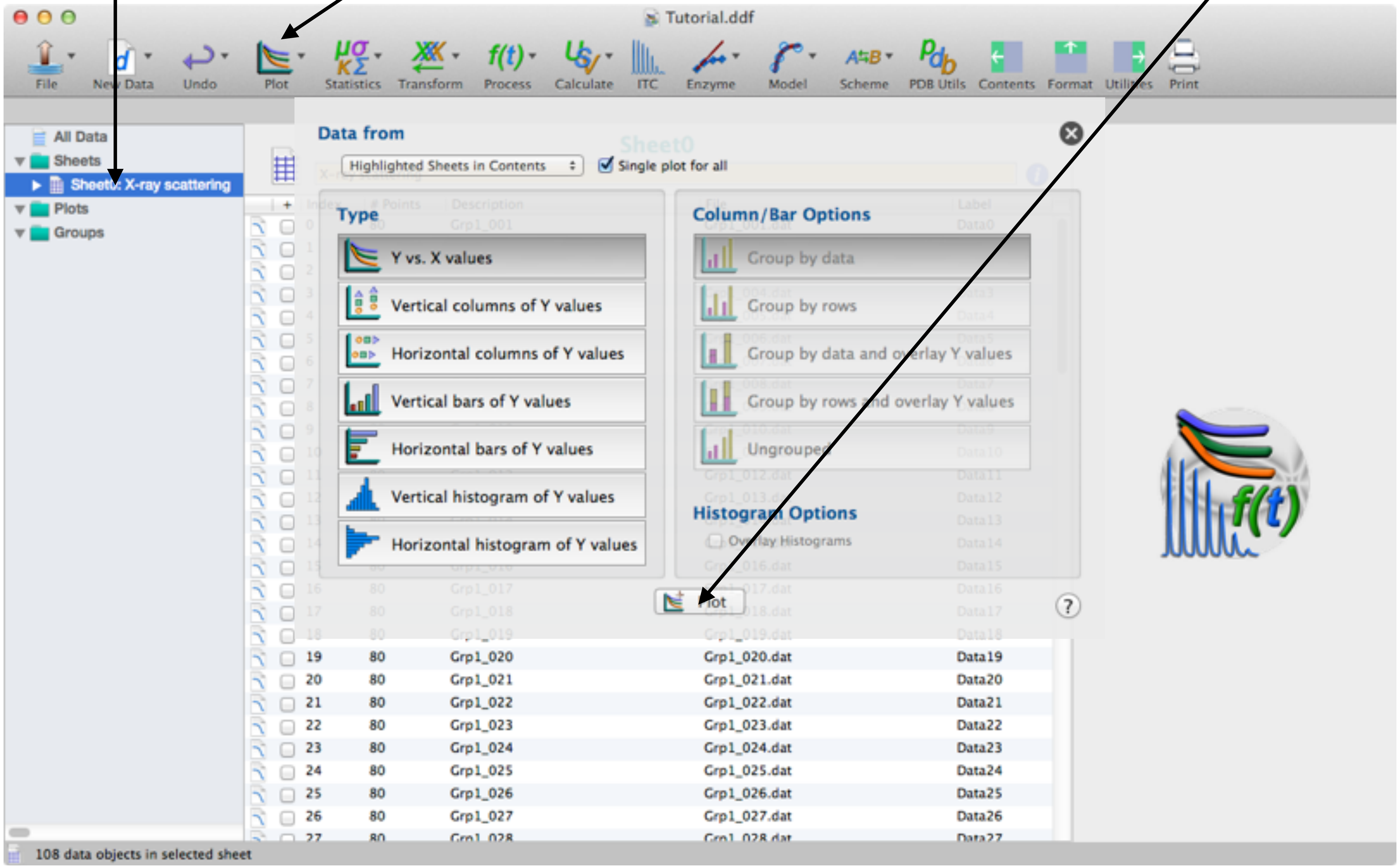

Plot of scattering data

Single click to select axes or text; double click to edit

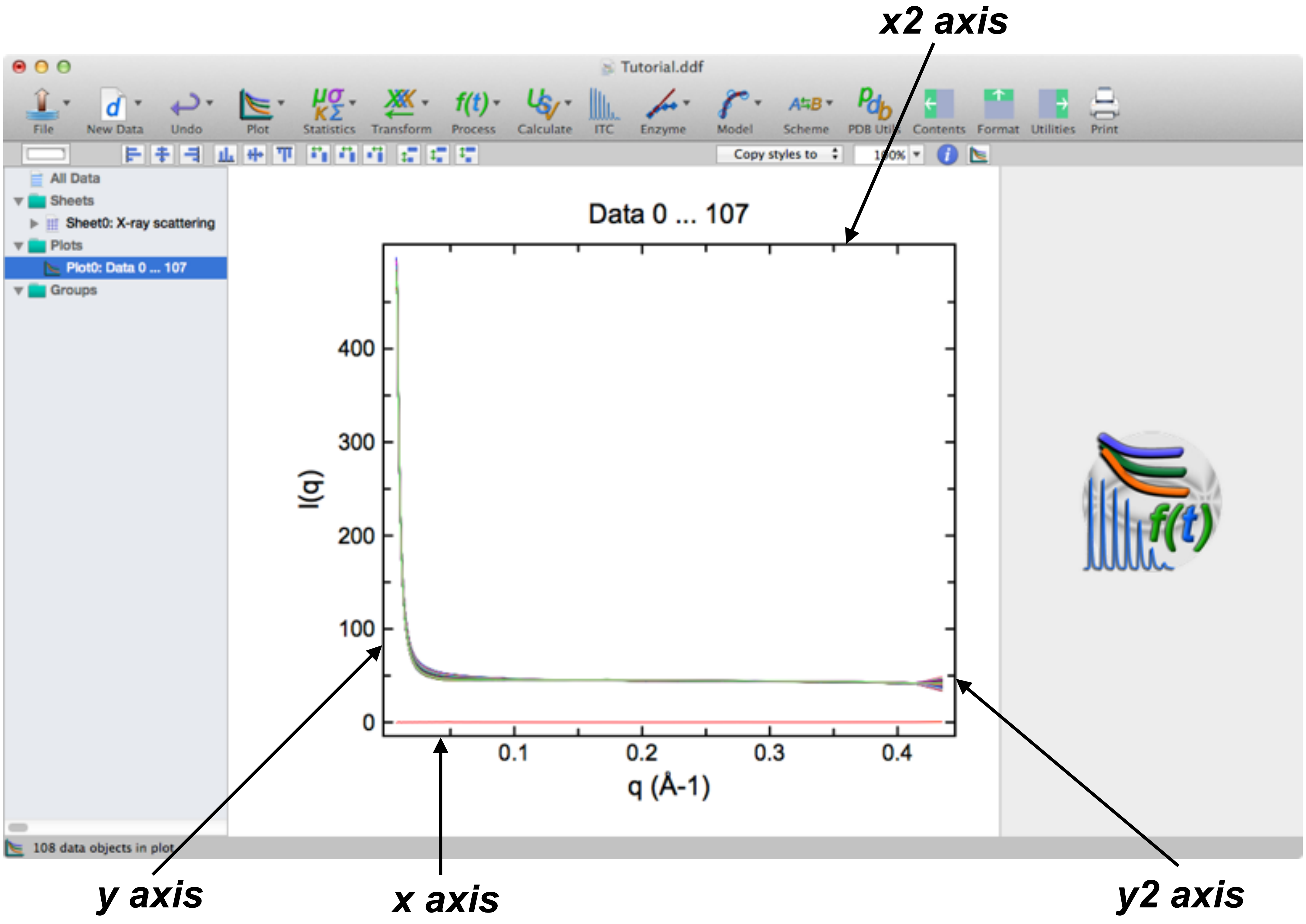

Play …

1. Click on an axis or intersection of two axes to select; double click to open the Plot Axes utility
Note appearance of the Axis Format bar, which can be used to change axis properties and styles.

2. Select log10 from the scale menu
The scale is set to linear by default.

3. Right mouse click (or control-click) for popup menu with various options
Note keyboard shortcuts for some menu items.

4. Click on text to select, double click to edit
Note appearance of the Text Format bar.

5. Single click on a data point to select the corresponding data object; double click to open the Plot Symbols utility
Note appearance of the Symbols Format bar.

5. Single click on the plot background
Note appearance of the Plot Format bar. Click on the Plot Control button in the Plot Format bar to open a panel that controls visibility of data objects and alternative plot axes.

Edit plot of scattering data

1. Click on the plot containing the scattering data in the contents view

2. Remove any data objects with errant data points as well as data objects lacking significant scattering content
Click on any data point to select a data object. Press the delete key to remove the selected data object from the plot. If necessary, use the Plot Control panel to scan through data objects individually. Note that removing a data object from a plot does not remove it from a sheet and vice versa. Likewise, removing a data object from all plots and sheets does not remove it from the document. However, removing a data object from the All Data master list, removes it from document including plots and sheets. Any data, sheet or plot removed from the document can be recovered if undo is active, by reverting to the most recent save, or by closing and reopening the document.

3. Save the document
Select File ➞ Save or Save As in document tool bar. DELA does not automatically save the document, so periodic saves are recommended.

Edit plot of scattering data

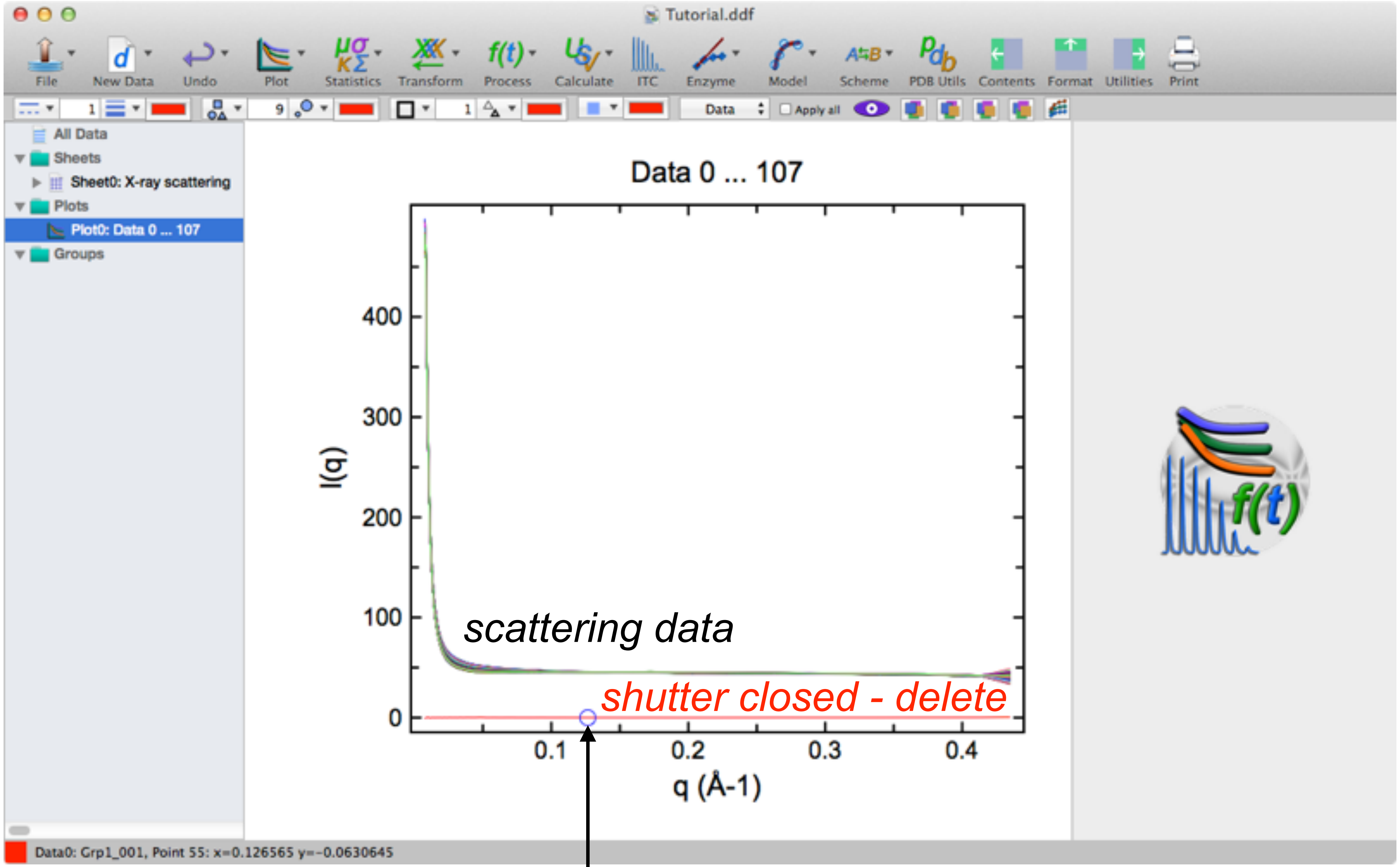

selected data point
and
related info

Scattering data on $\log_{10}$ I(q) vs. $\log_{10}$ q scale

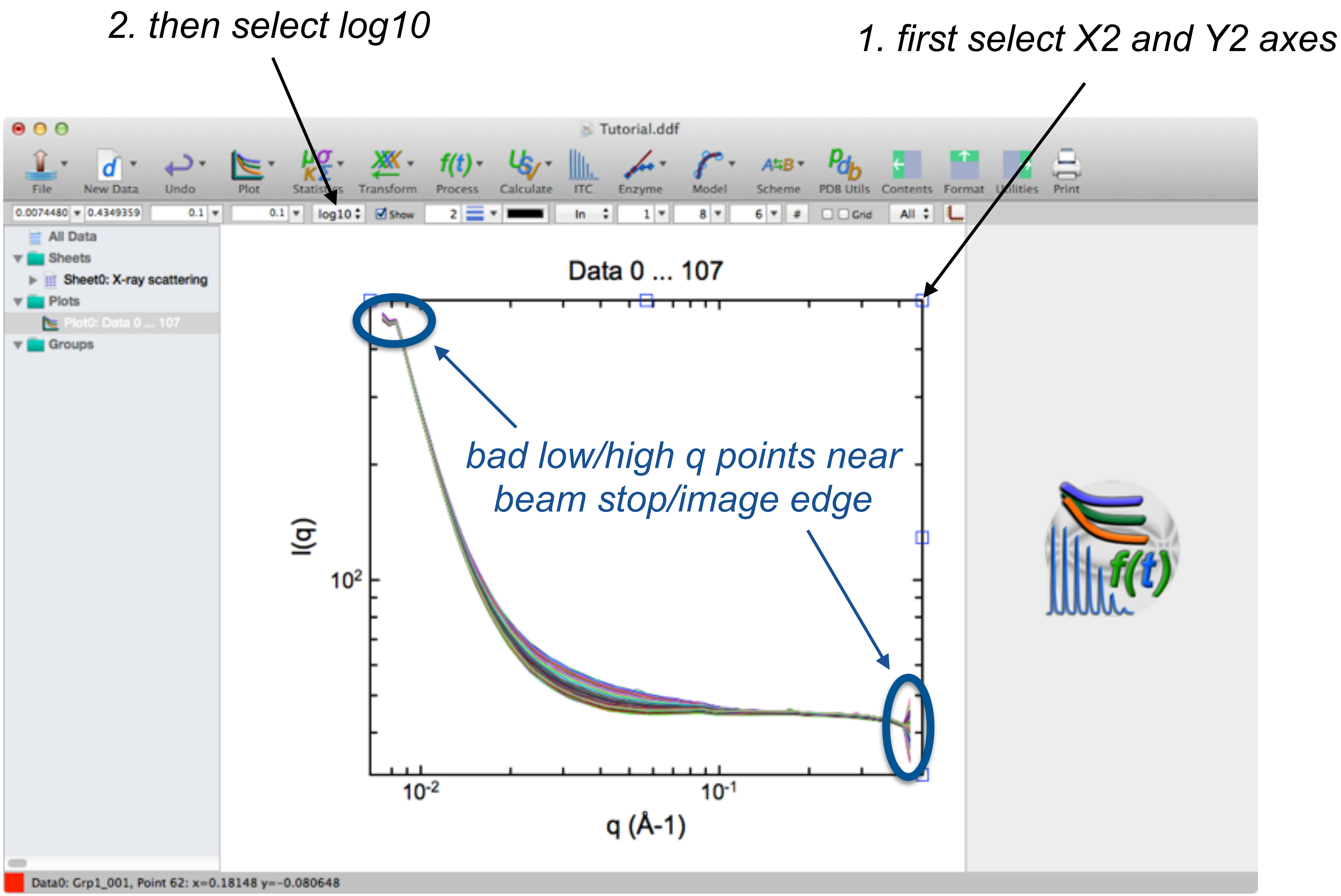

View and edit the scattering data in tabular format

1. Click on the sheet containing the scattering data in the contents view
Note listing of the data objects in the sheet. Also note disclosure triangle in the contents view, which can be used to toggle visibility of individual data objects.

2. Double click on any data object in the list displayed in the main view
The data object will be selected in the contents view and displayed in tabular form in the main view.

In addition to individual data objects, expanded sheets in the contents view include an item at the top that can be selected to display the sheet contents as a whole.

3. Click on the first item in the expanded sheet

4. Delete poor quality data points (if any) at the beginning and end
Select the entire row(s) for the poor quality data points and hit the delete key. It is important that the number and alignment of data points is consistent between data objects.

5. Right mouse click to access popup menu with display/editing options

6. Click the disclosure triangle to collapse the sheet

View and edit the scattering data in tabular format

1. Select to edit all data in sheet

2. Select first two rows and press delete key

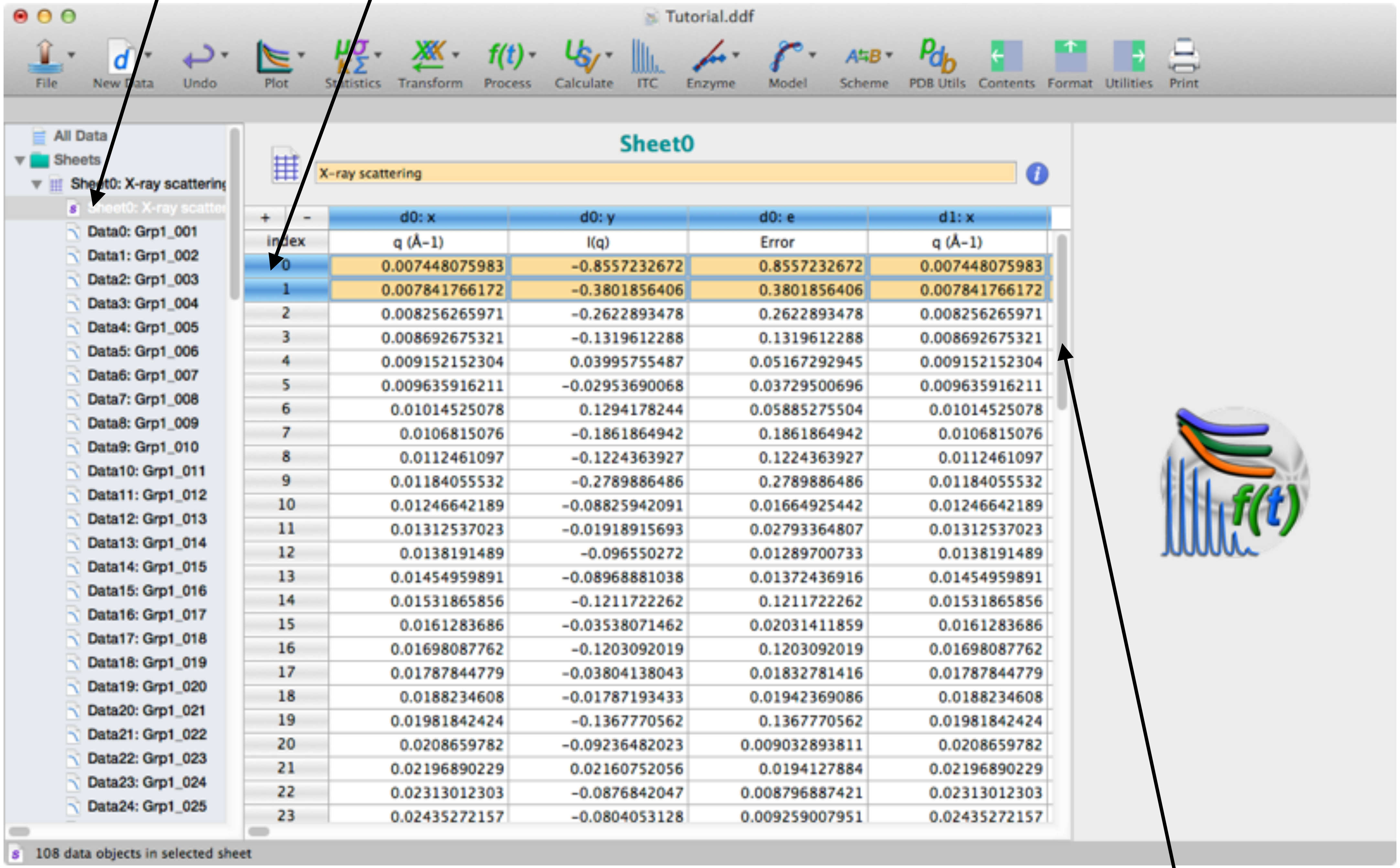

3. Scroll down to select and delete the last row

Scattering data plot after editing

1. Click to activate Plot Control utility

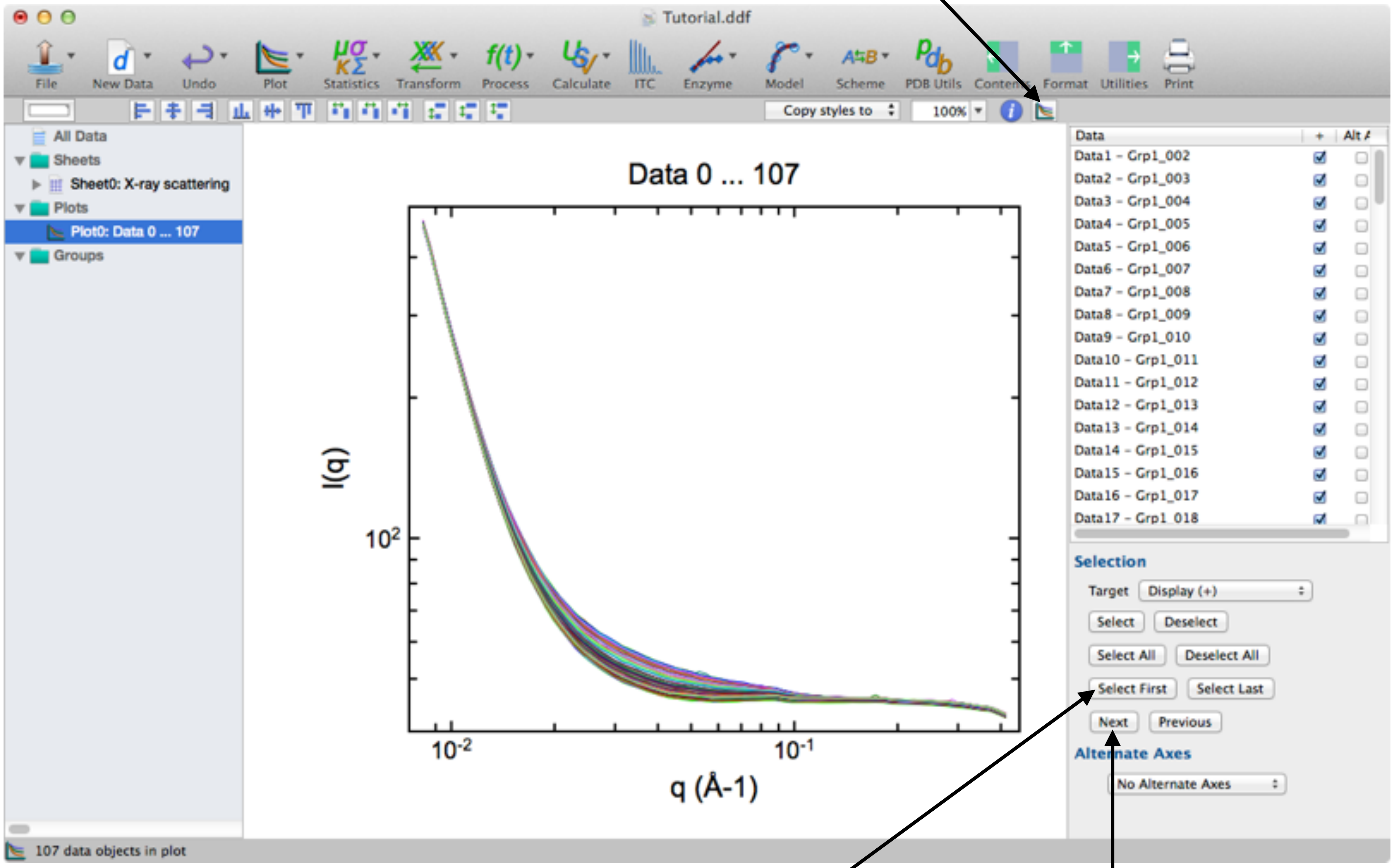

2. Click to display only the first data object

3. Click to display only the next data object …

Generate a profile of the total (or mean) scattering

The profile is analogous to an ‘chromatogram’ where the total (or mean) intensity of each data set is plotted as a function of the data set index. Note that the data index will be used by certain scripts so don’t change the x values for now. If desired, the data index can be converted to elution volume at a later stage when the data index is no longer needed.

1. Click on the plot containing the scattering data in the contents view

2. Select Process → Interpret Script in the tool bar

3. Select SAXS_Total_Scattering.py or SAXS_Mean_Scattering.py in the script menu and click Interpret

Generate a profile of the total (or mean) scattering

1. Select Interpret Script from Process Menu

2. Select SAXS_Total_Scattering.py

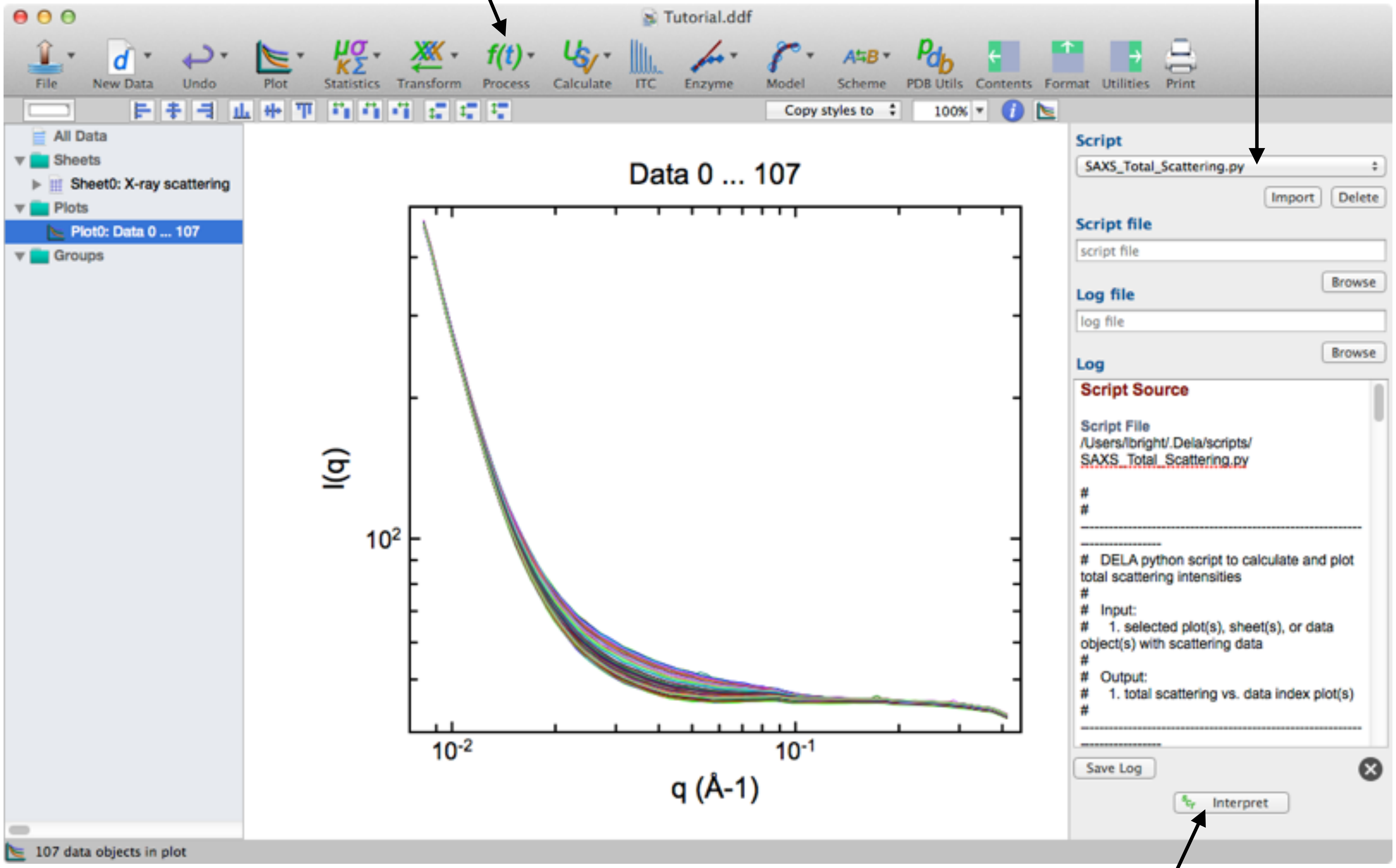

3. Press Interpret

Plot of total (or mean) scattering vs. data index

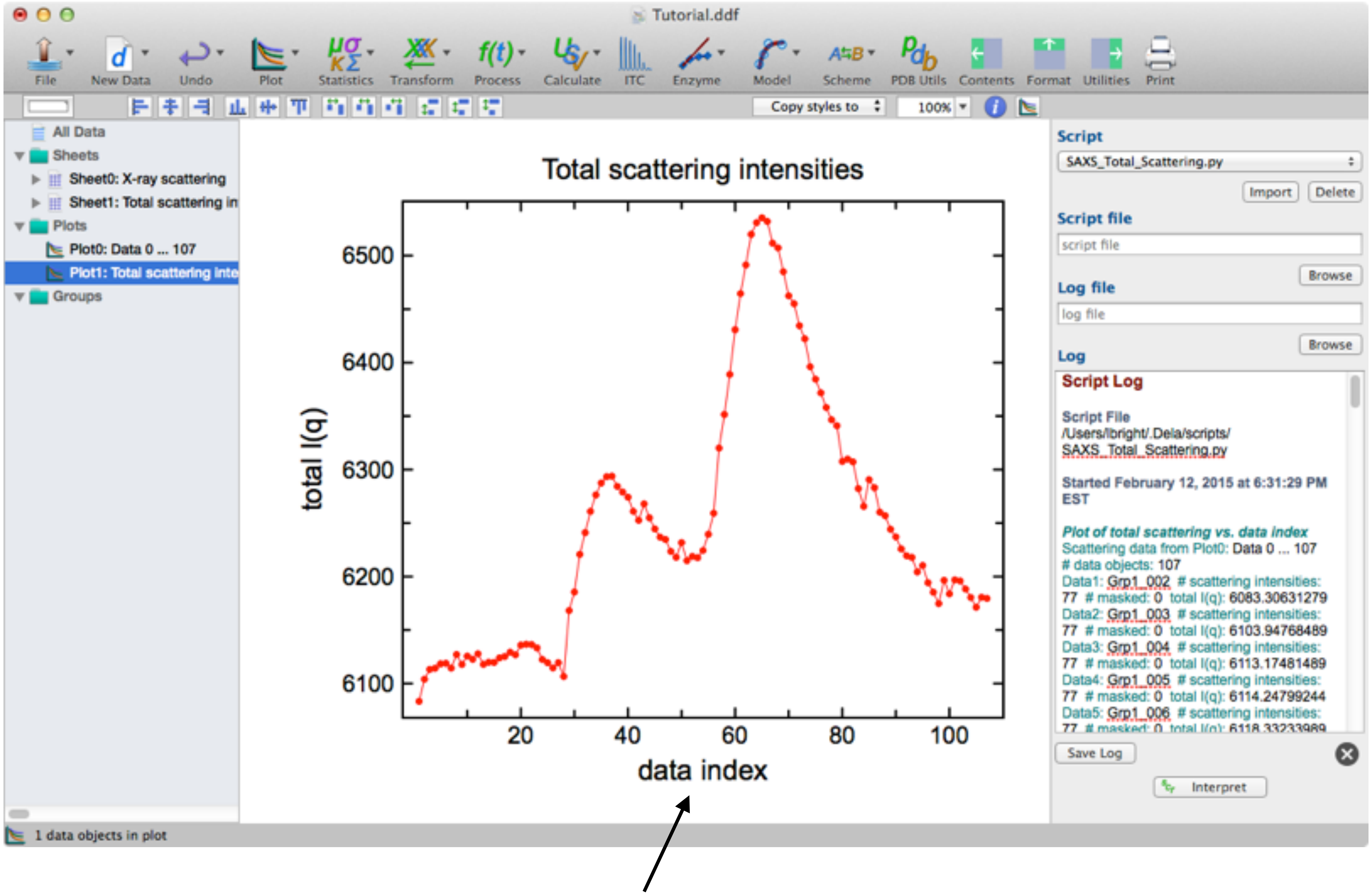

Index of the data in the All Data master list

Plotting selected regions of interest

1. Select the plot of the total (or mean) scattering profile in the contents view

2. Display the selection regions

Right mouse button ➞ Display ➞ Selection Regions or type the keyboard short cut 's' to toggle display of the selection regions. One region is displayed by default. Addition regions can be added using Right mouse button ➞ Add Region.

3. Click on a region and adjust the bounds to encompass the data points of interest

4. Run the SAXS_Plot_Data_With_Indices_in_Selection_Regions.py script

Non-redundant data sets corresponding to points bounded by the region(s) in the total (or mean) scattering plot will be plotted.

Plotting data sets near the monomer peak maximum

Selection Region bounding points corresponding to three data sets at the maximum of the monomer peak

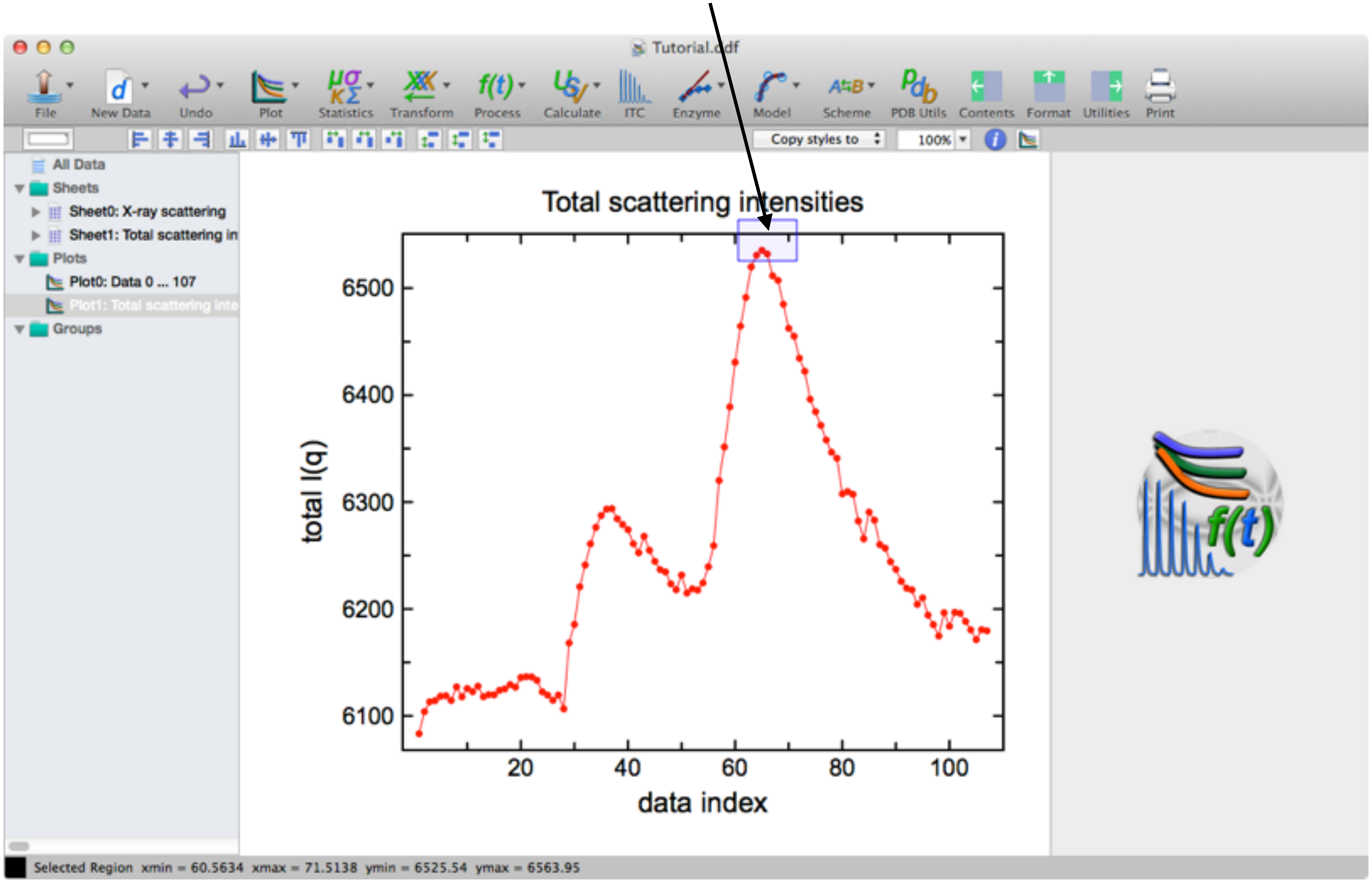

SEC-SAXS 'peak − pre and/or post peak buffer subtraction

1. Select the plot of the total (or mean) scattering profile in the contents view and display the selection regions

2. Click on a region and adjust the bounds to encompass data points representing protein+buffer scattering (e.g. point near a peak maximum).

4. Run the SAXS_Plot_Data_With_Indices_in_Selection_Regions.py script

5. Select the newly created plot, delete outliers if necessary, and select Transform → Average from the document tool bar.
If desired, the curves can be scaled prior to averaging by selecting Calculate → General Linear Least Squares from the document tool bar.

6. Repeat steps 3-5 with one or more regions representing buffer scattering.

7. Select the averaged protein+buffer data in the contents view and run the SAXS_Buffer_Subtraction.py or SAXS_Buffer_Subtraction_Guinier_Analysis.py script
The data label for the averaged buffer data object from step 6 will need to be provided. A new sheet and plot with the subtracted data will be generated.

Plot with data from peak maximum on $\log_{10}$ $\log_{10}$ scale

1. Optionally scale by selecting General Linear Least Squares from the Calculate menu

2. Select average from Transform menu

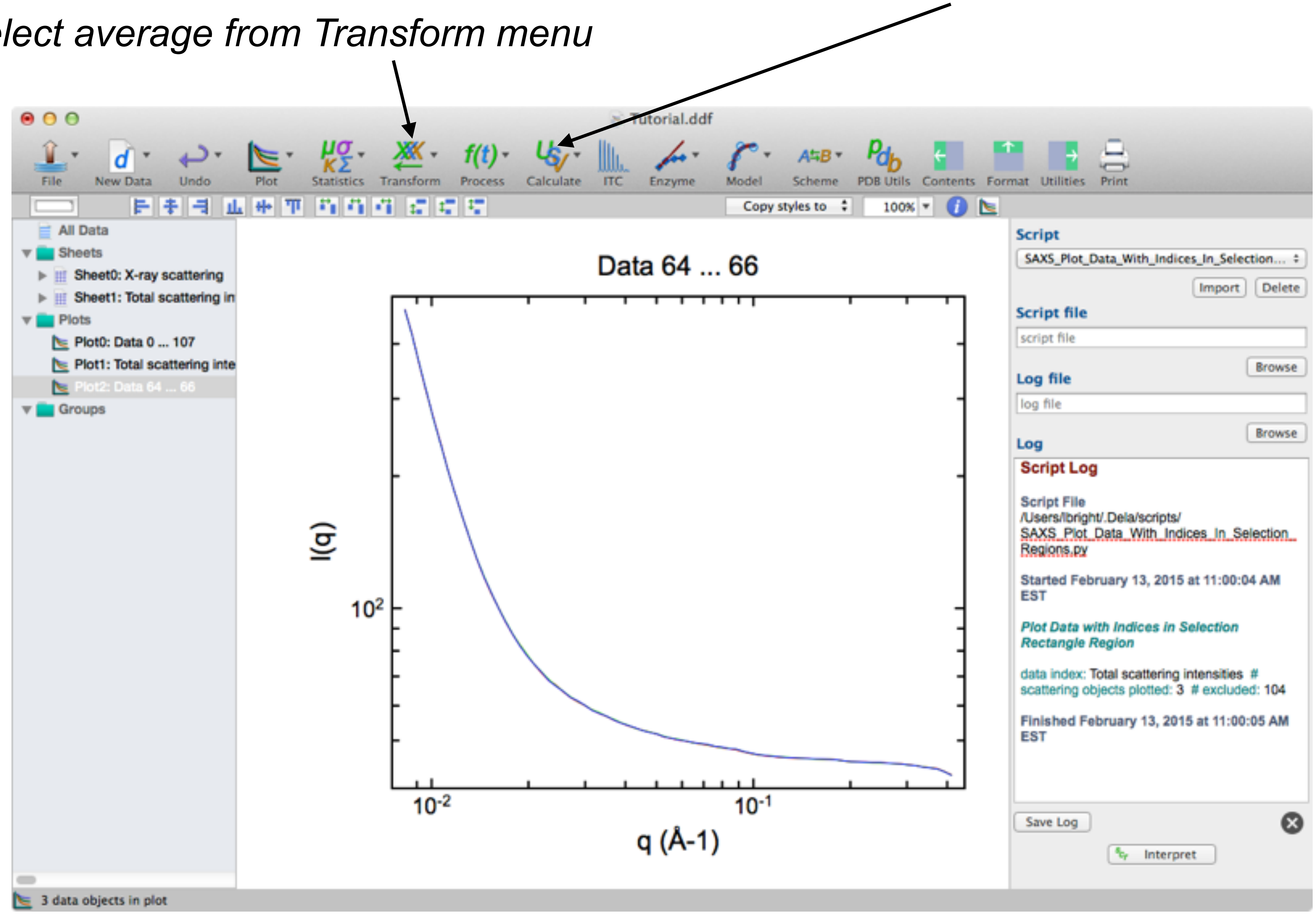

Plotting data sets from a pre-peak 'buffer' region

Selection Region adjusted to encompass a pre-peak region that may (or may not) be a well matched buffer

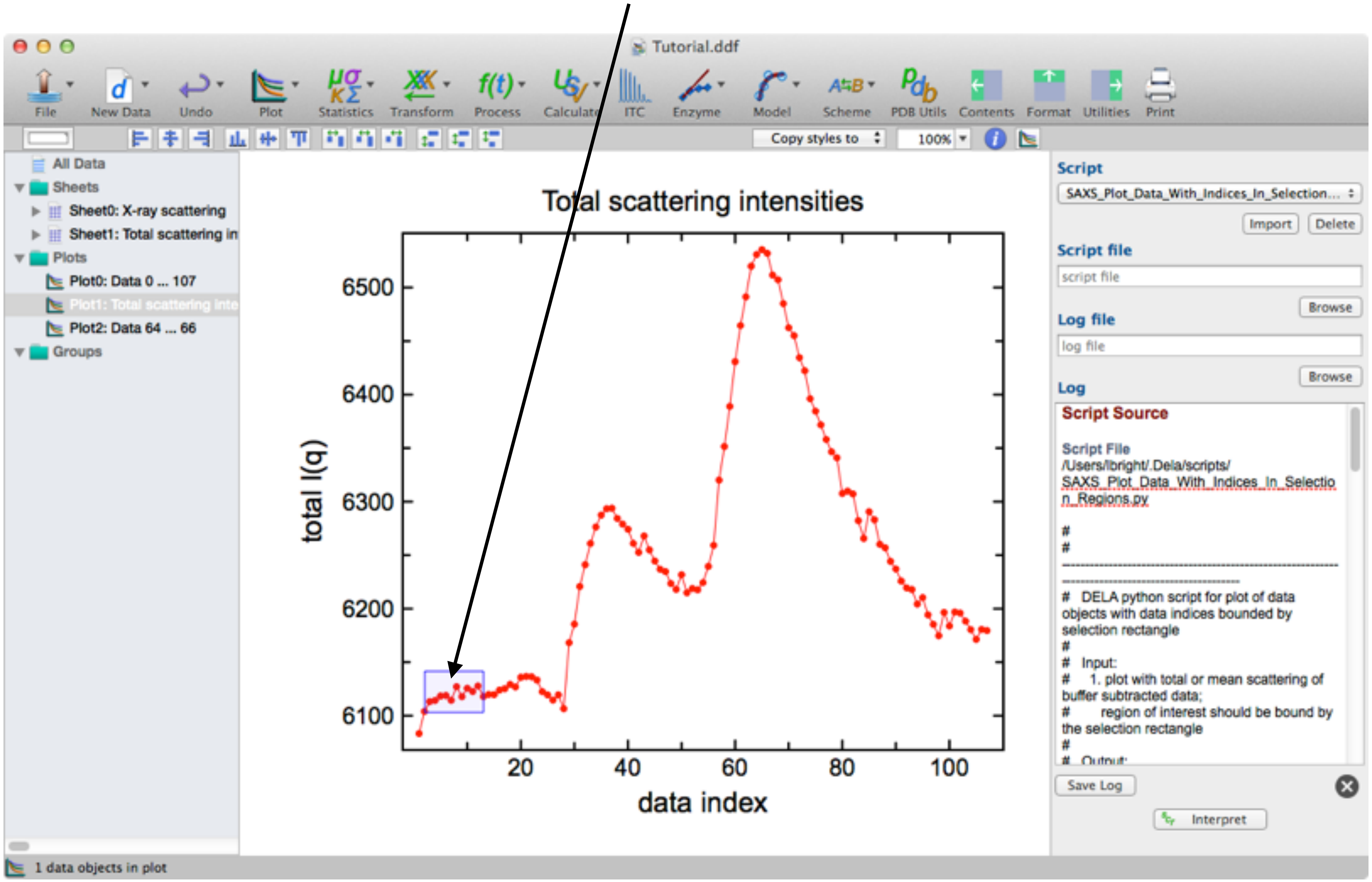

Plot with data from pre-peak 'buffer' on $\log_{10}$ $\log_{10}$ scale

1. *Optionally scale by selecting General Linear Least Squares from the Calculate menu*
2. *Select average from Transform menu*

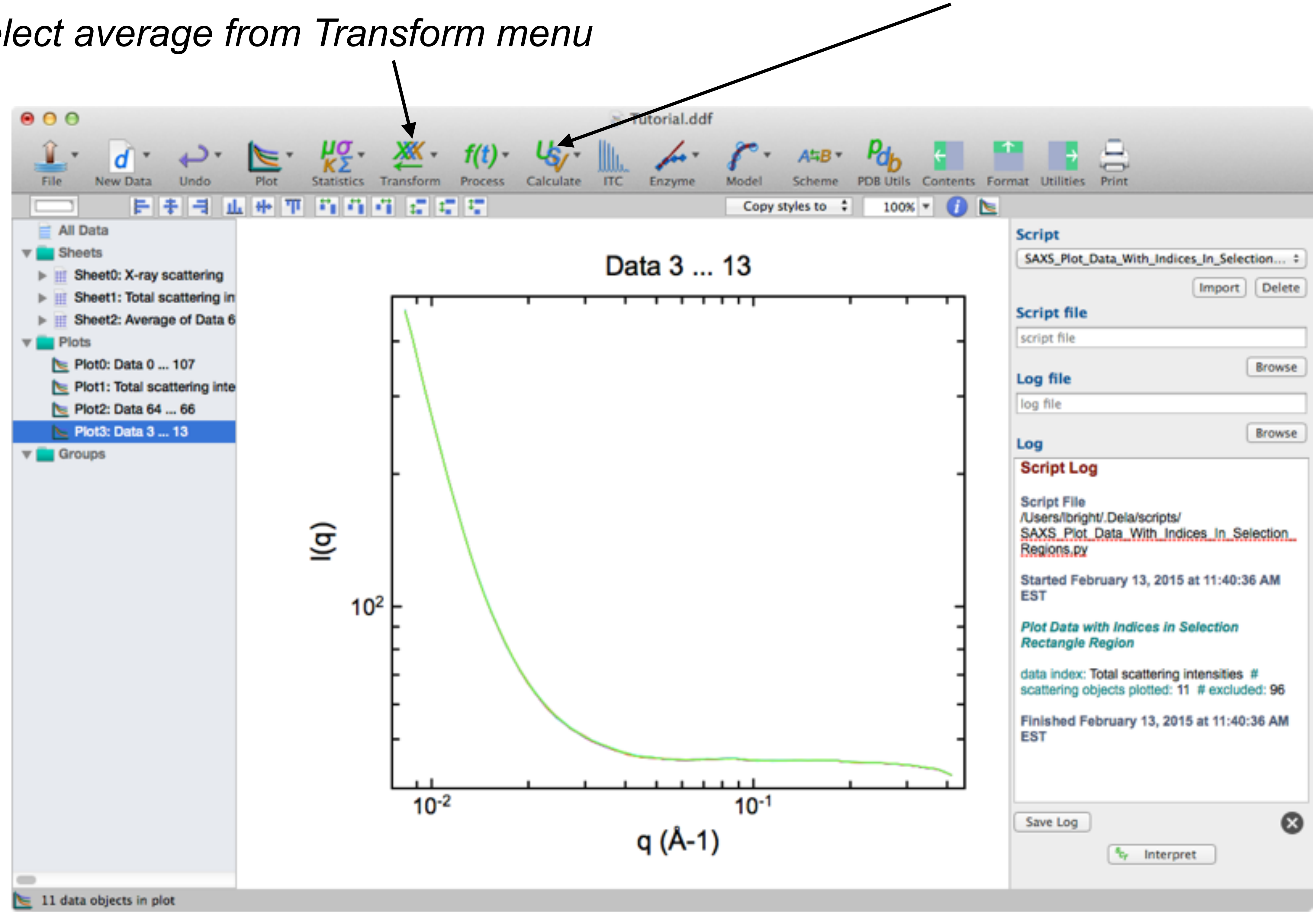

Plot with averaged peak and buffer data on log_{10} log_{10} scale

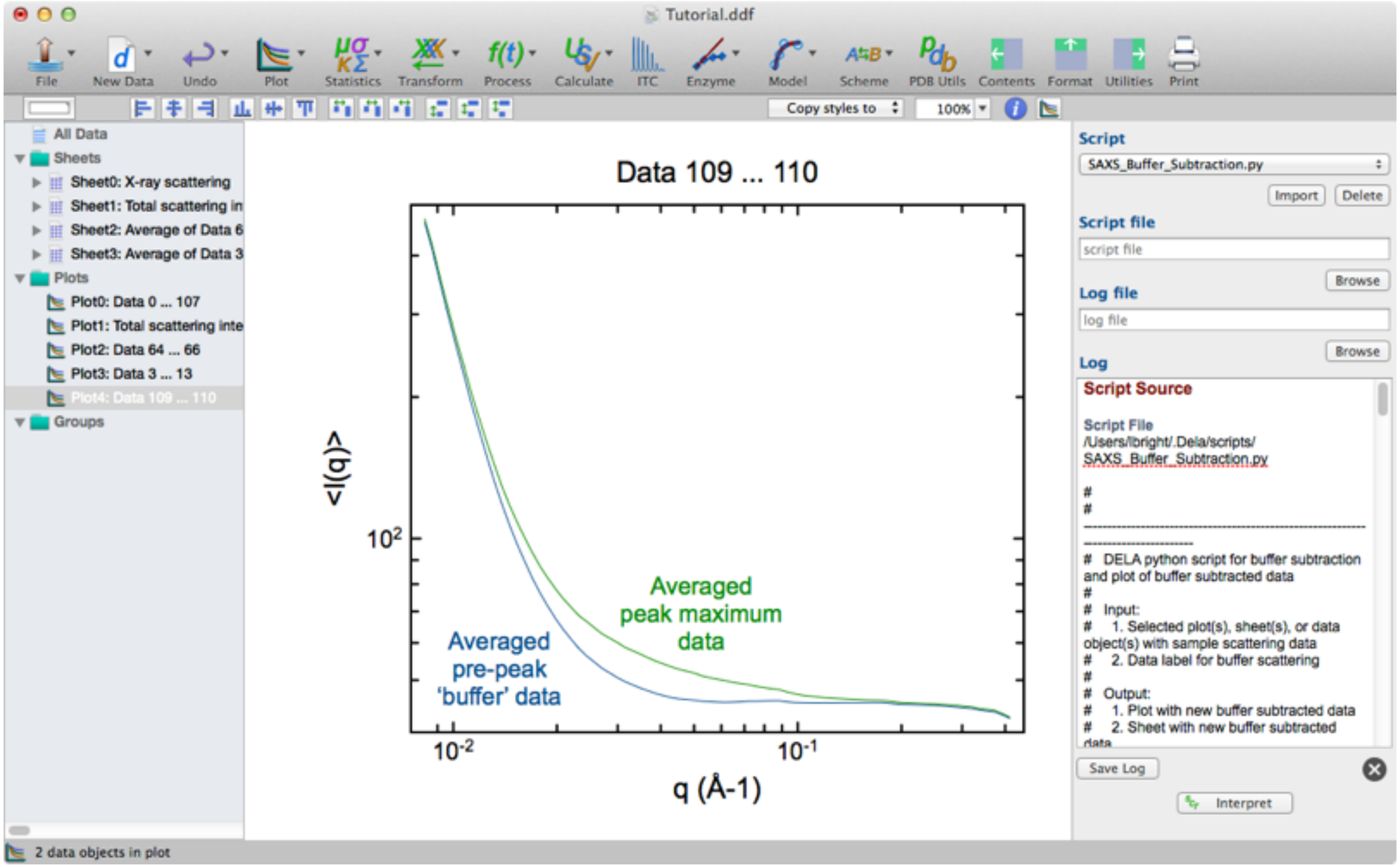

Looks ok at this stage so proceed to buffer subtraction …

Conventional SAXS buffer subtraction

1. Select a sheet or plot containing the protein+buffer scattering and select Transform → Average from the document tool bar.

If desired, the curves can be scaled prior to averaging by selecting Calculate → General Linear Least Squares from the document tool bar.

2. Repeat step 1 for the buffer scattering.

3. Select the averaged protein+buffer data in the contents view and run the SAXS_Buffer_Subtraction.py or SAXS_Buffer_Subtraction_Guinier_Analysis.py script

The data label for the averaged buffer data object from step 6 will need to be provided. A new sheet and plot with the subtracted data will be generated.

Buffer subtraction

1. Select sheet with averaged peak maximum data

2. Note data label for 'buffer' (Data110)

3. Run SAXS_Buffer_Subraction.py

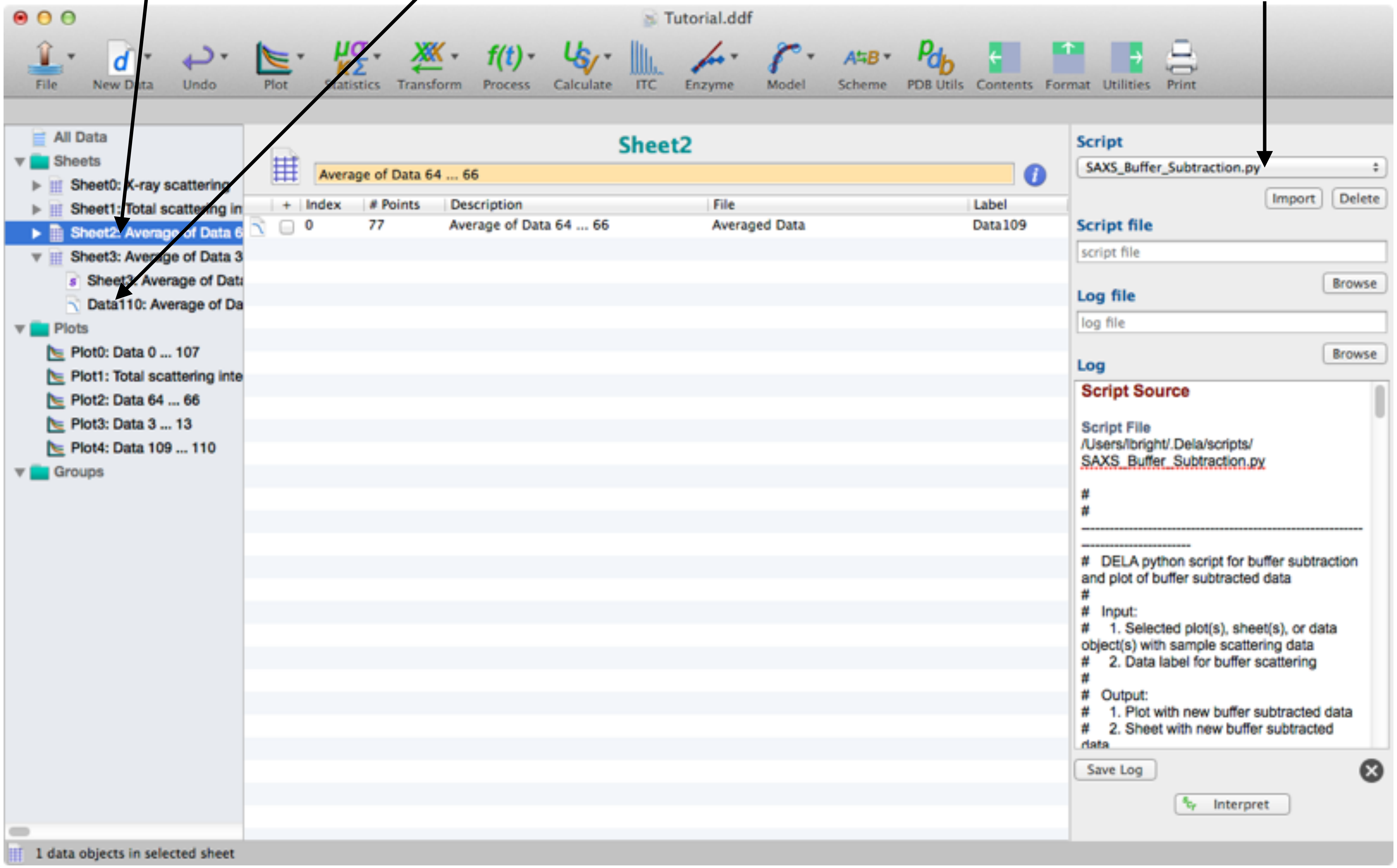

Plot with peak − pre-peak buffer on $\log_{10}$ q scale

Negative artifact at low q may be normalization error, buffer mismatching, etc.

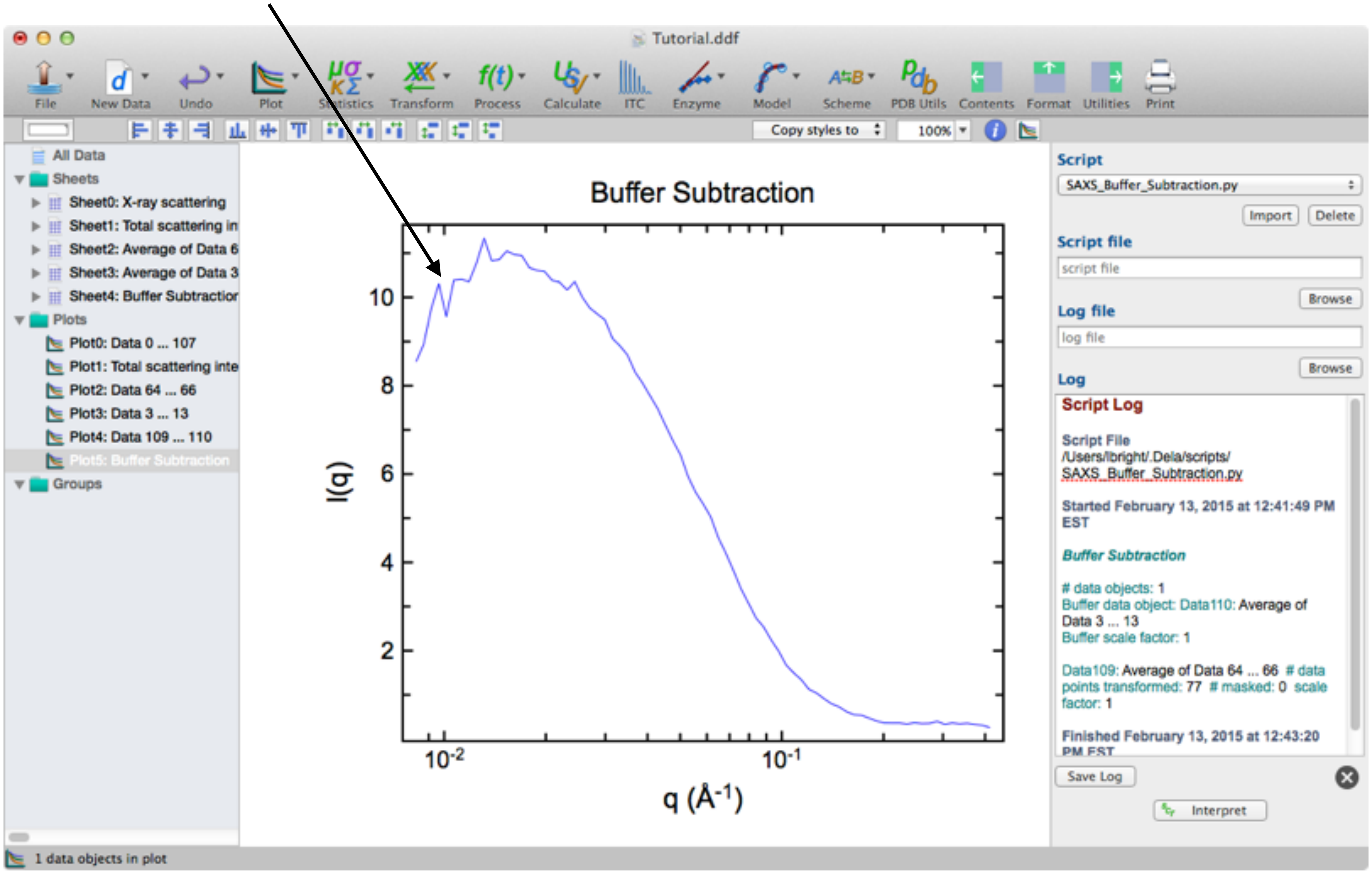

Could try different buffer region, maybe post peak …

Plotting data sets from a post-peak 'buffer' region

Selection Region adjusted to encompass a post-peak region

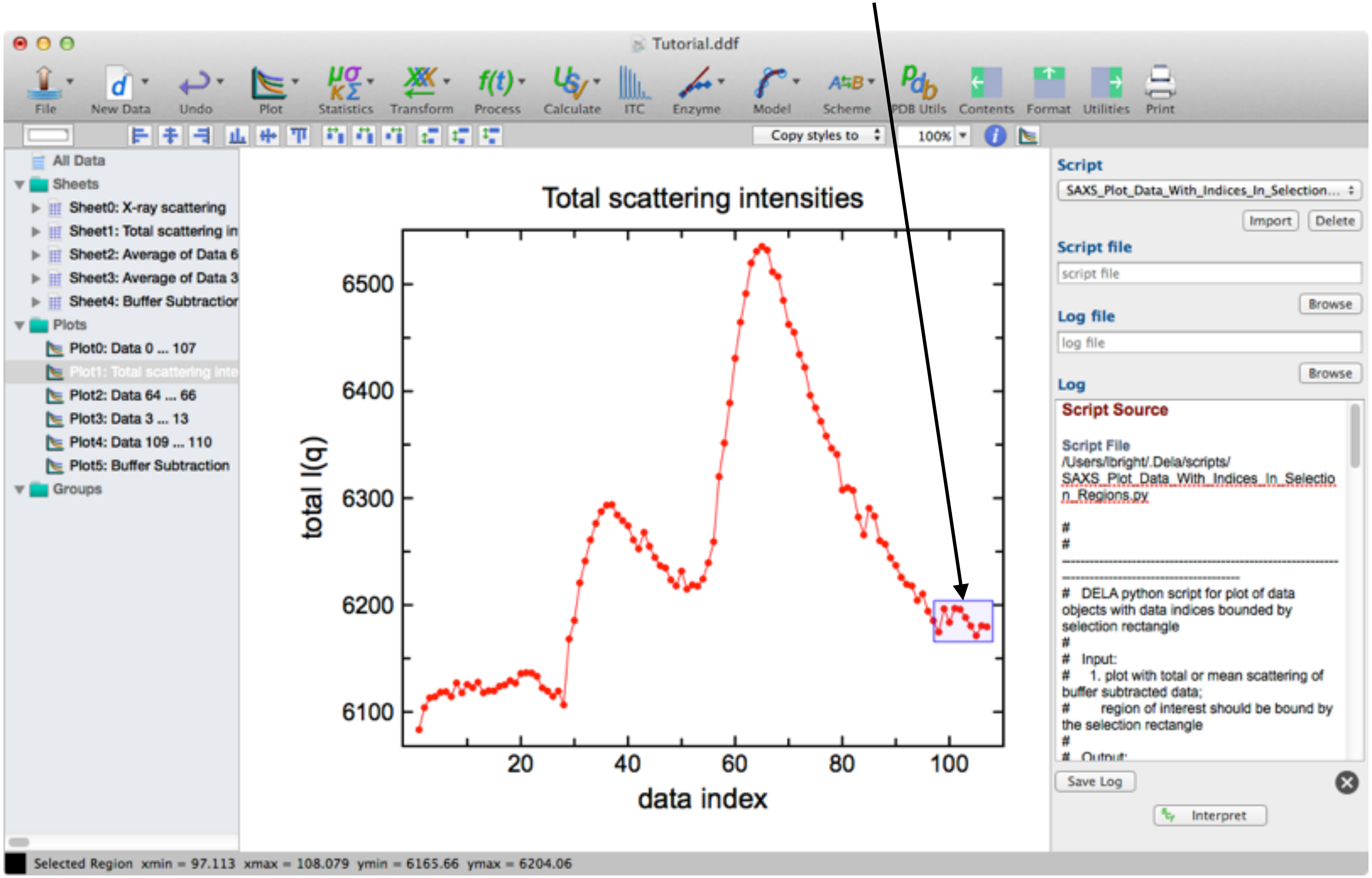

Plot with peak − pre-peak buffer on $\log_{10}$ q scale

Smaller but still negative artifact at low q

High q data negative

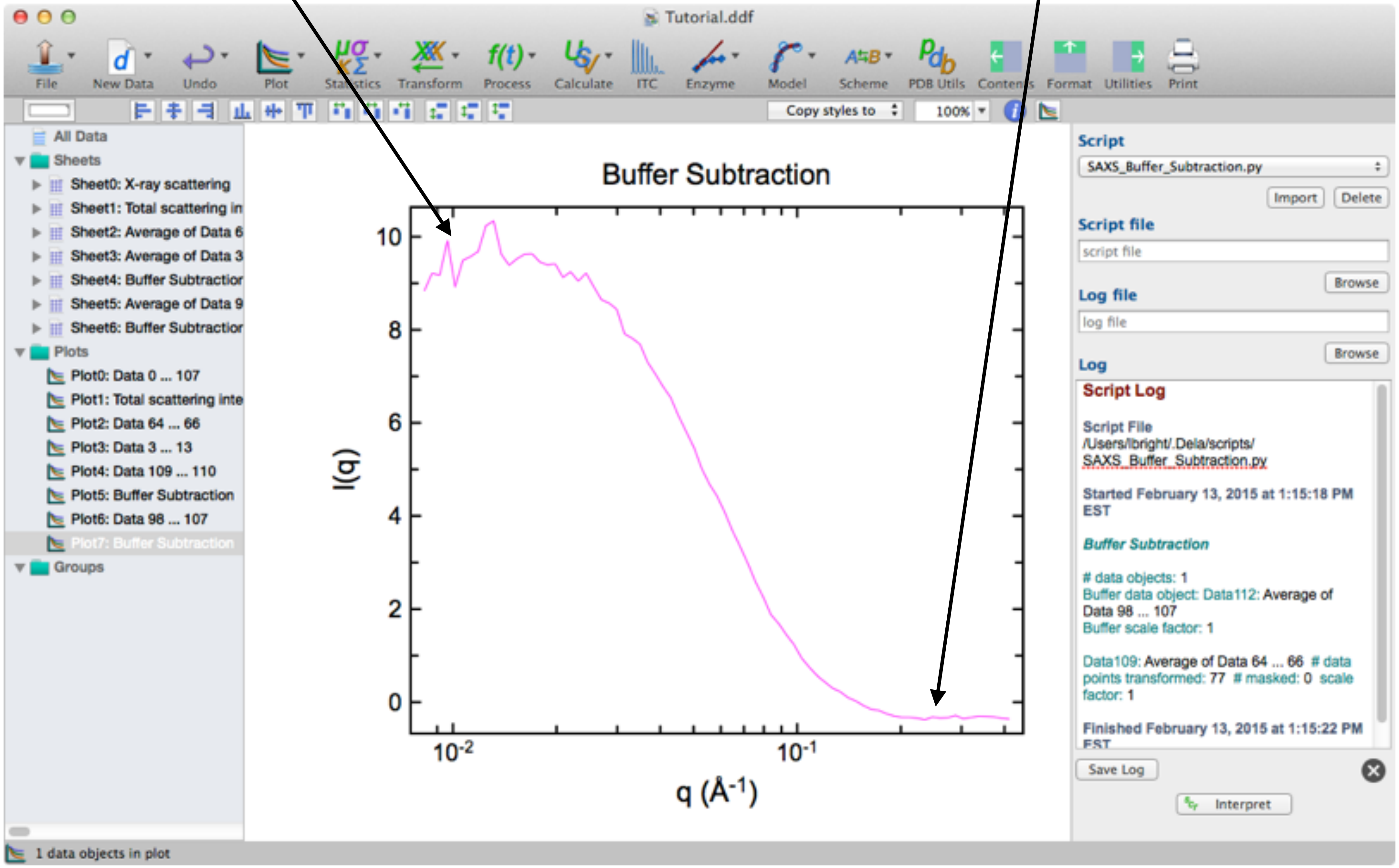

Could try subtracting average of pre- and post-peak buffer regions …

Plotting data sets from pre- and post-peak 'buffer' regions

Add second selection region and adjust to encompass a pre- and post-peak regions

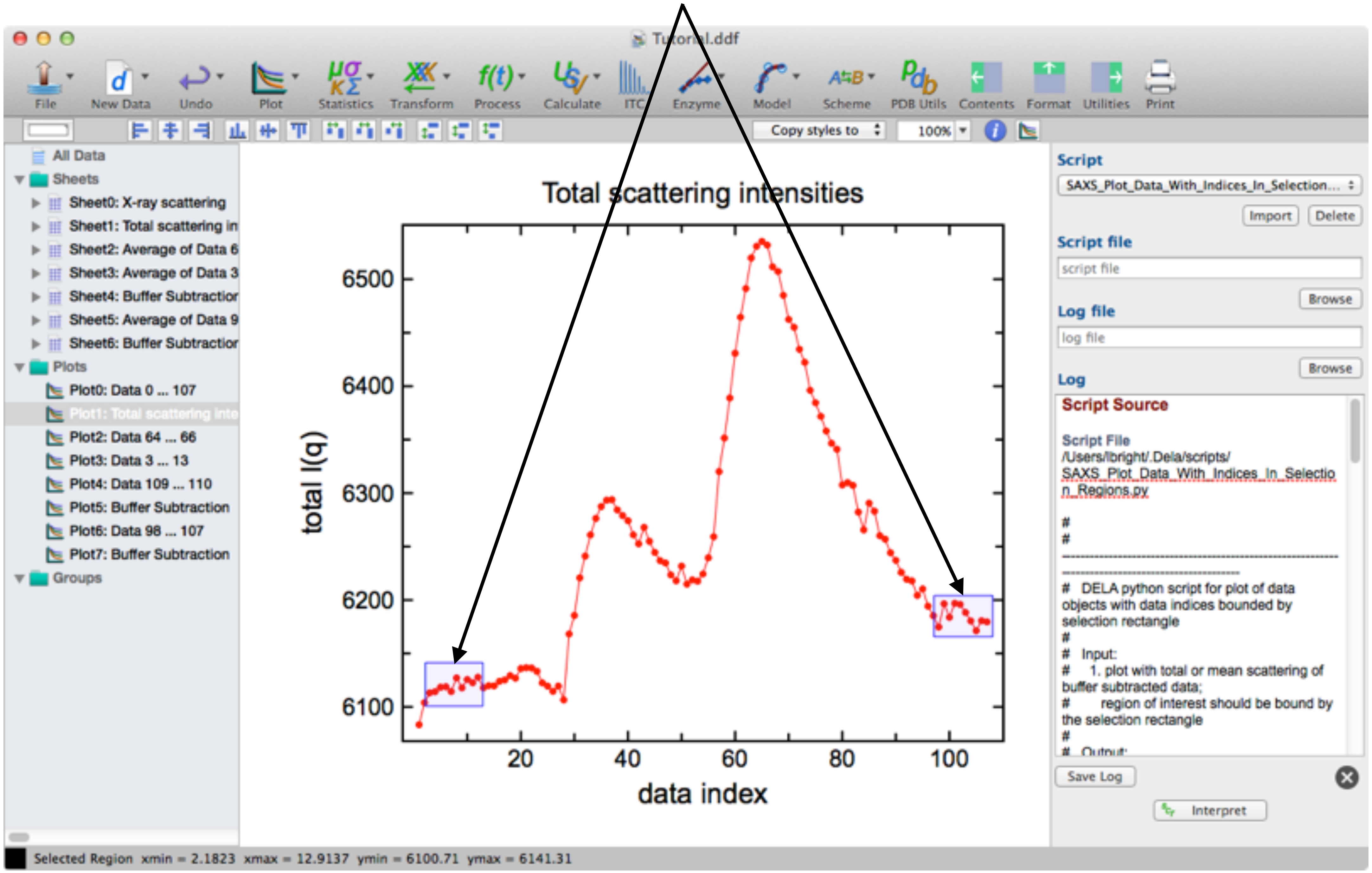

Plot with peak − pre/post-peak buffer on $\log_{10}$ q scale

Intermediate negative artifact at low q

High q data with few slightly negative points

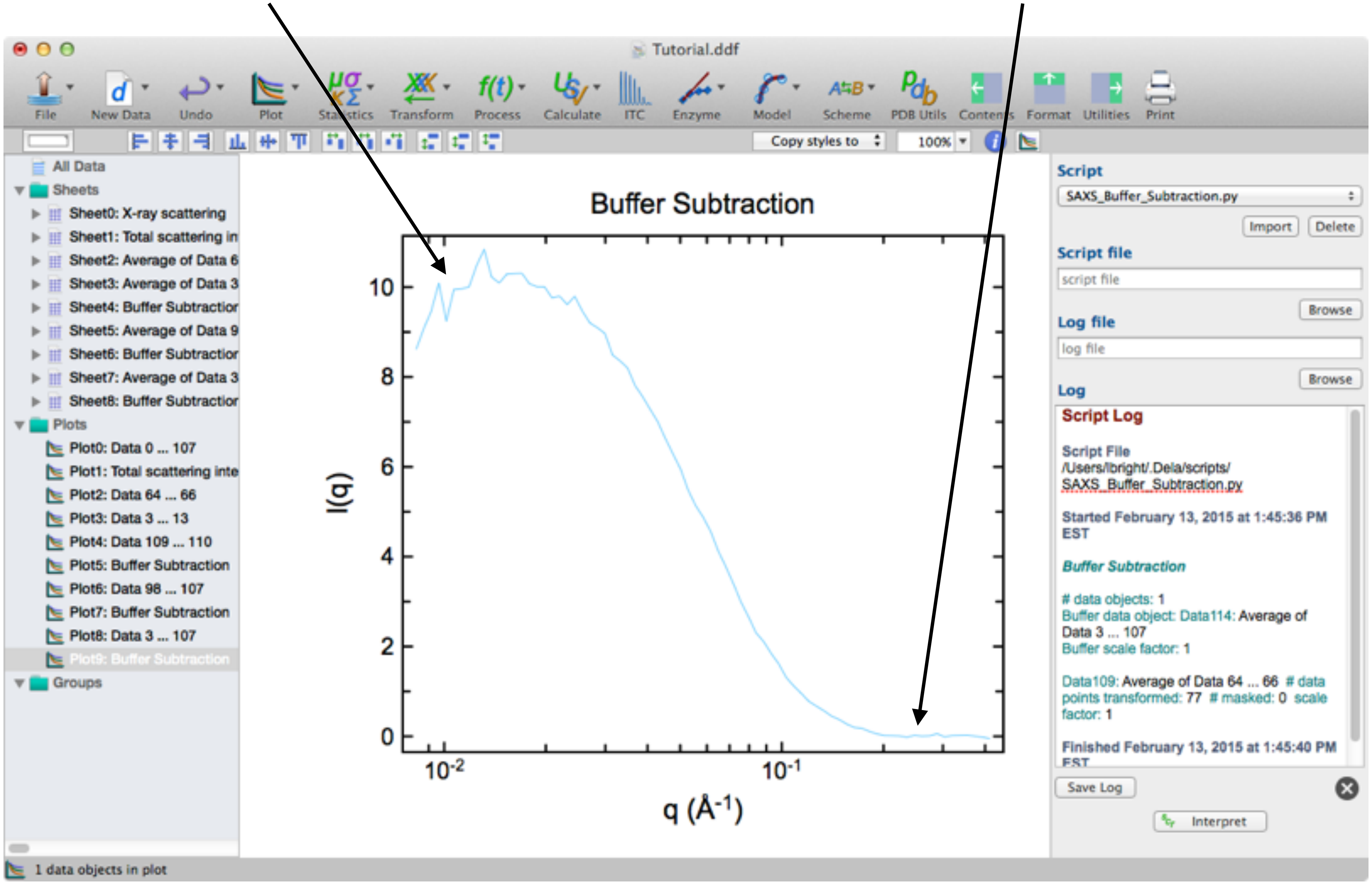

Looks like intermediate result …

Buffer subtraction with Guinier optimized scaling constant

1. Select sheet with averaged peak maximum data

2. Note data label for 'buffer' (Data110)

3. Run SAXS_Buffer_Subraction_Guinier_Analysis.py

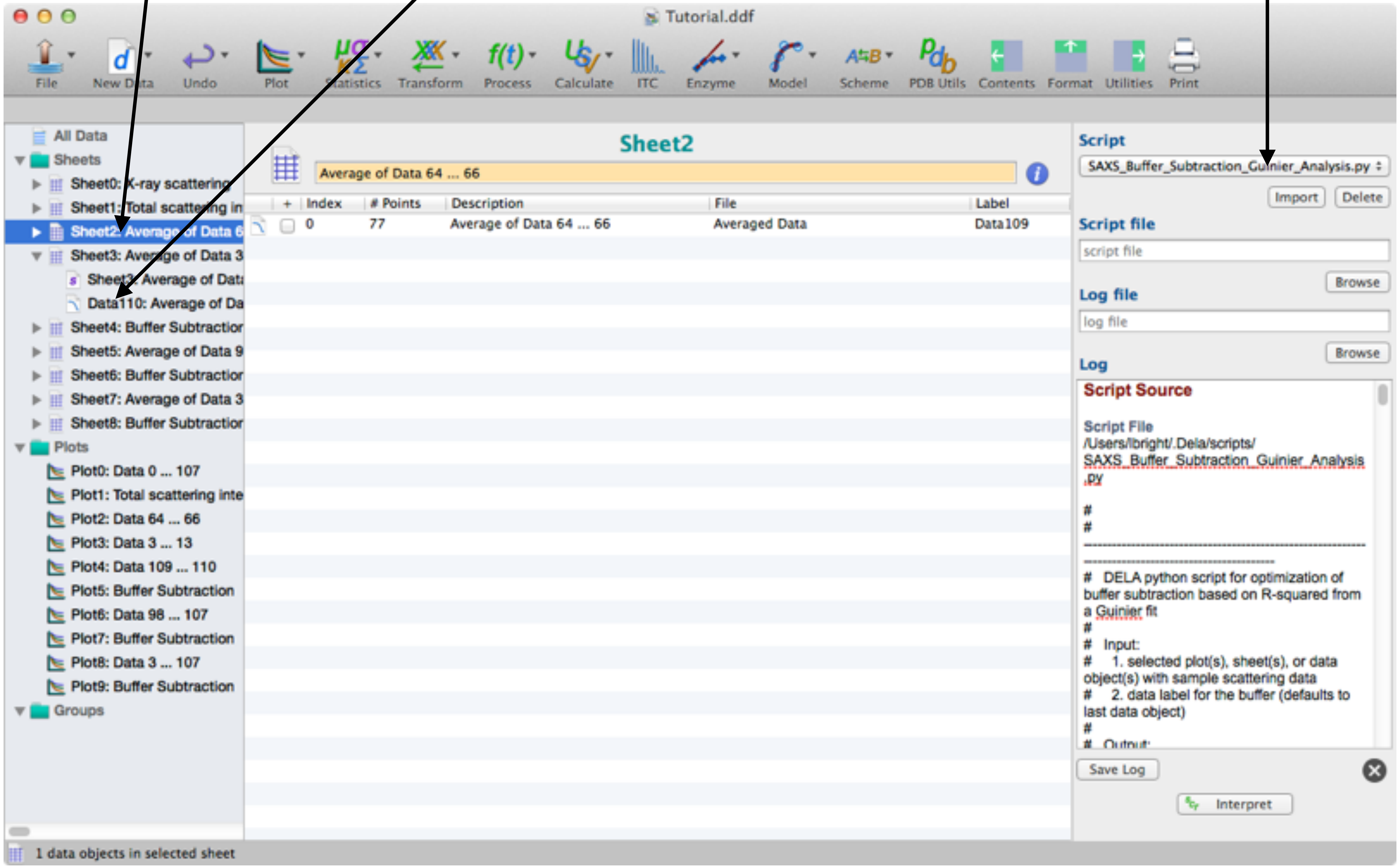

Guinier Analysis of peak − pre-peak buffer

Clear maximum in R^2 vs. buffer scaling constant

Reasonable value for buffer scaling constant near 1

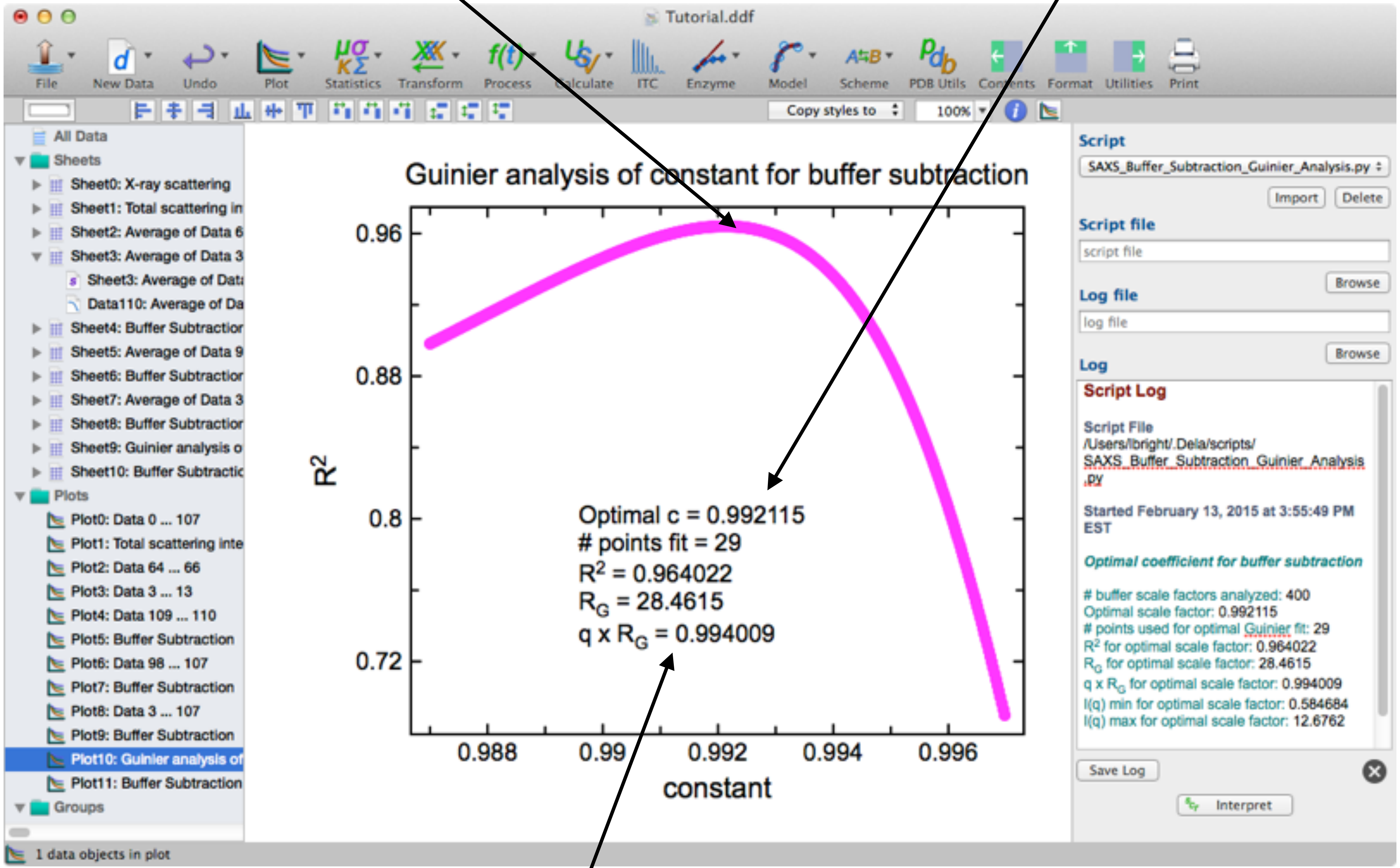

The other info refers to the subtraction with the Guinier optimized scaling constant

Plot of Guinier optimized peak − post-peak on log_{10} q scale

Minimal negative artifact at low q

High q data with no negative points

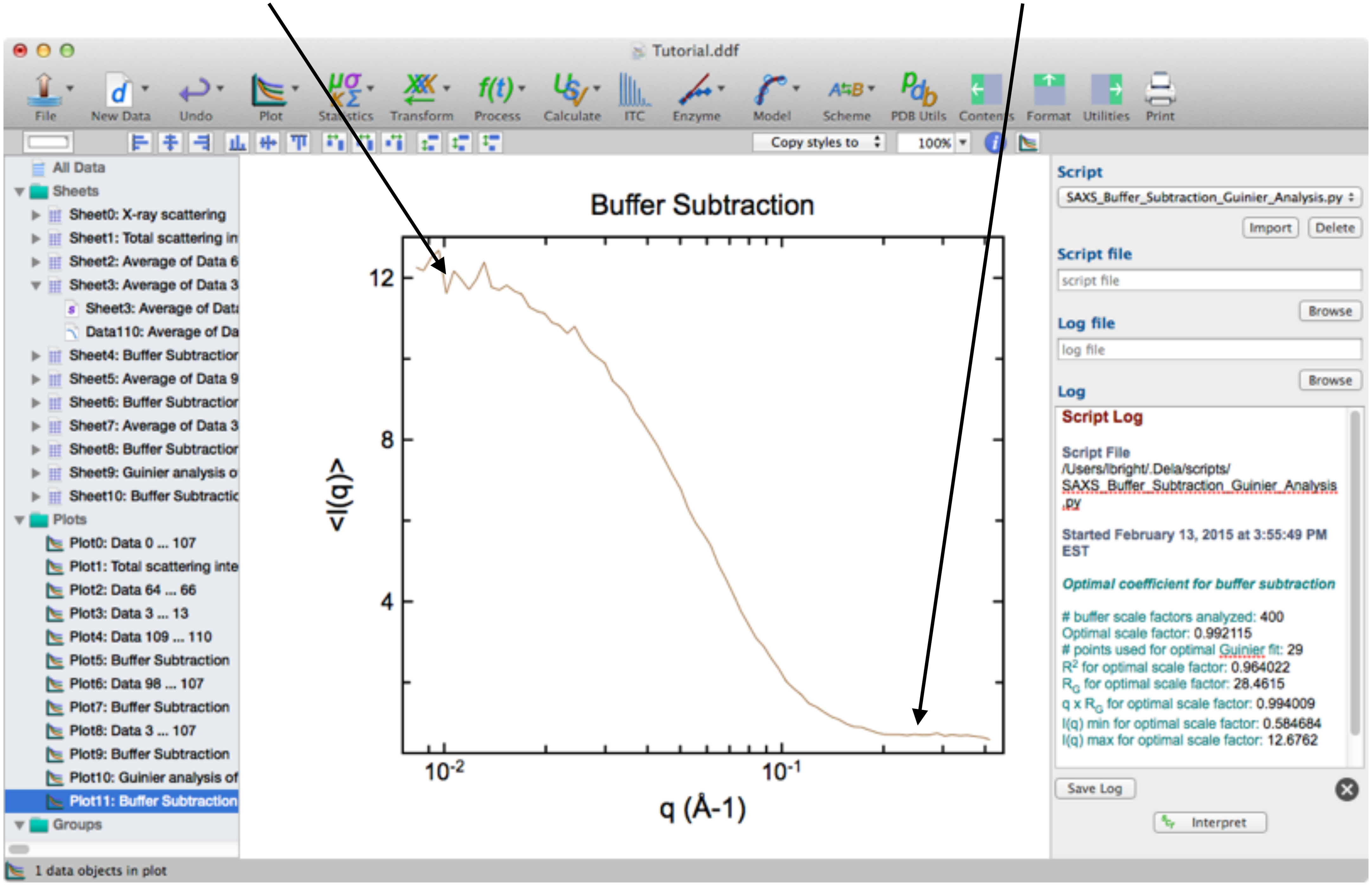

Looks like a reasonable result. Next try with post-peak buffer region …

Guinier Analysis of peak − post-peak buffer

Clear maximum in R^2 vs. buffer scaling constant

Reasonable value for buffer scaling constant near 1

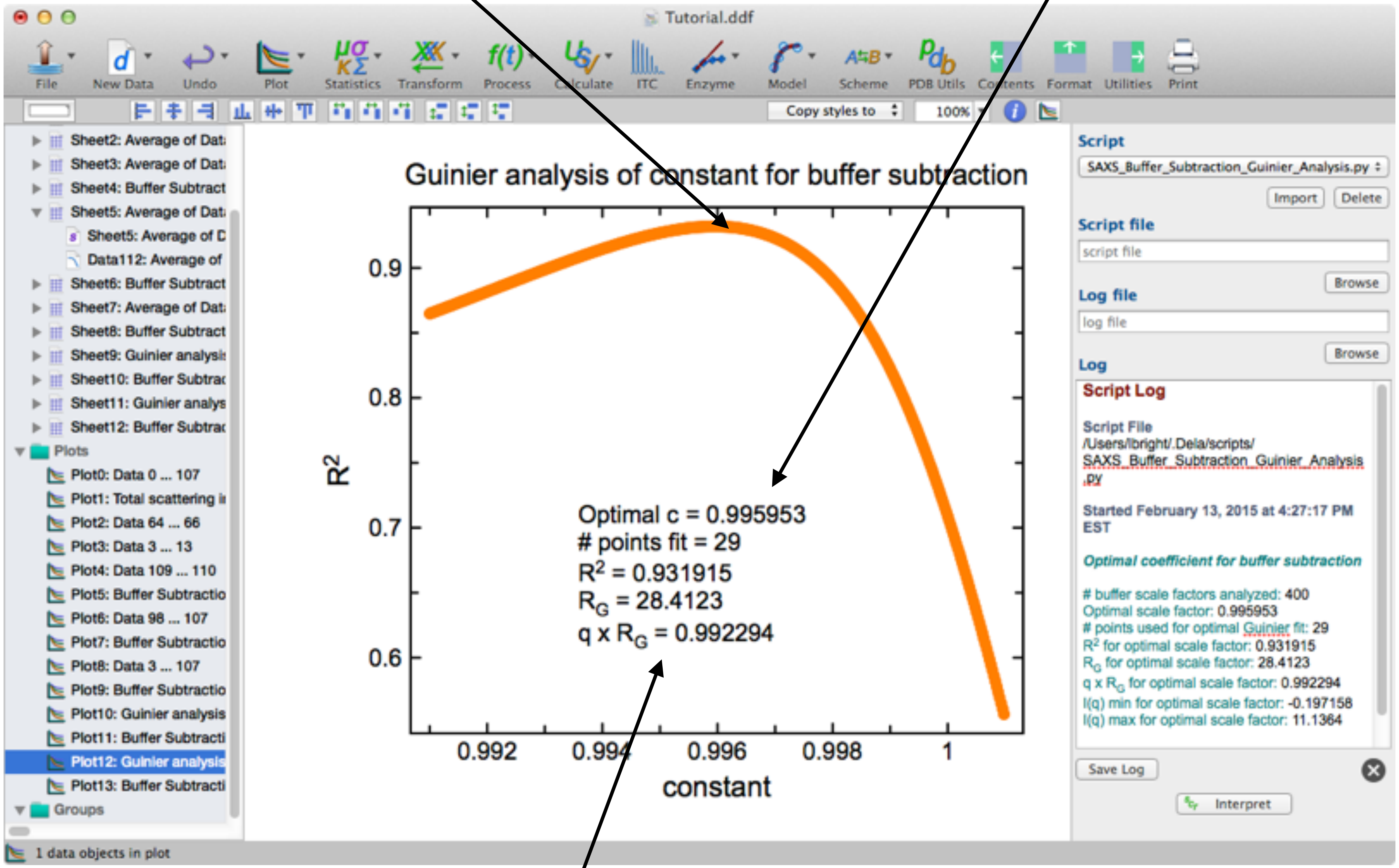

The other info refers to the subtraction with the Guinier optimized scaling constant

Plot of Guinier optimized peak − post-peak on $\log_{10}$ q scale

Minimal negative artifact at low q

Slightly negative in high q region

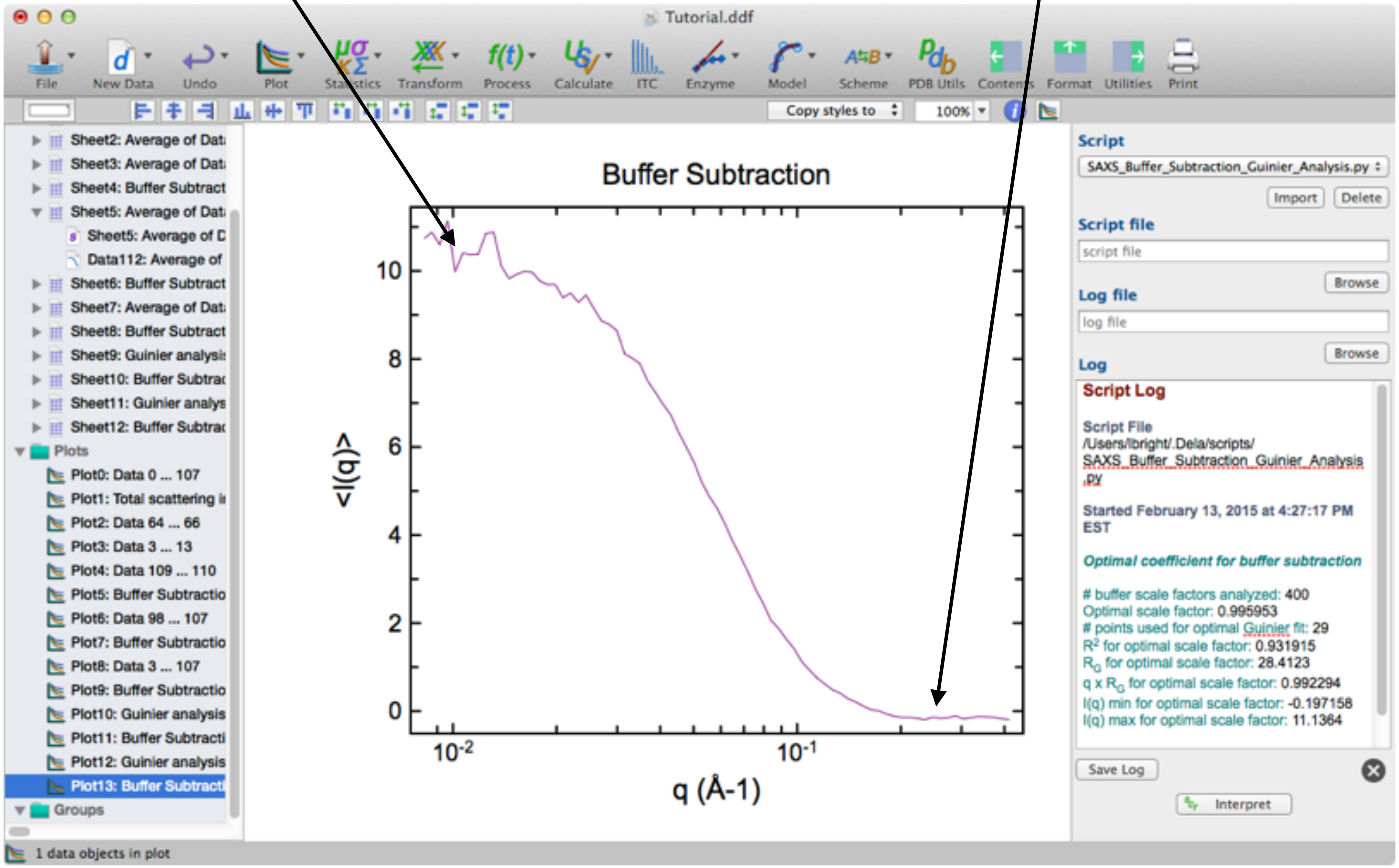

Also looks like a reasonable result. Next try with pre- and post-peak buffer regions …

Guinier Analysis of peak – pre/post-peak buffer

Clear maximum in R^2 vs. buffer scaling constant

Reasonable value for buffer scaling constant near 1

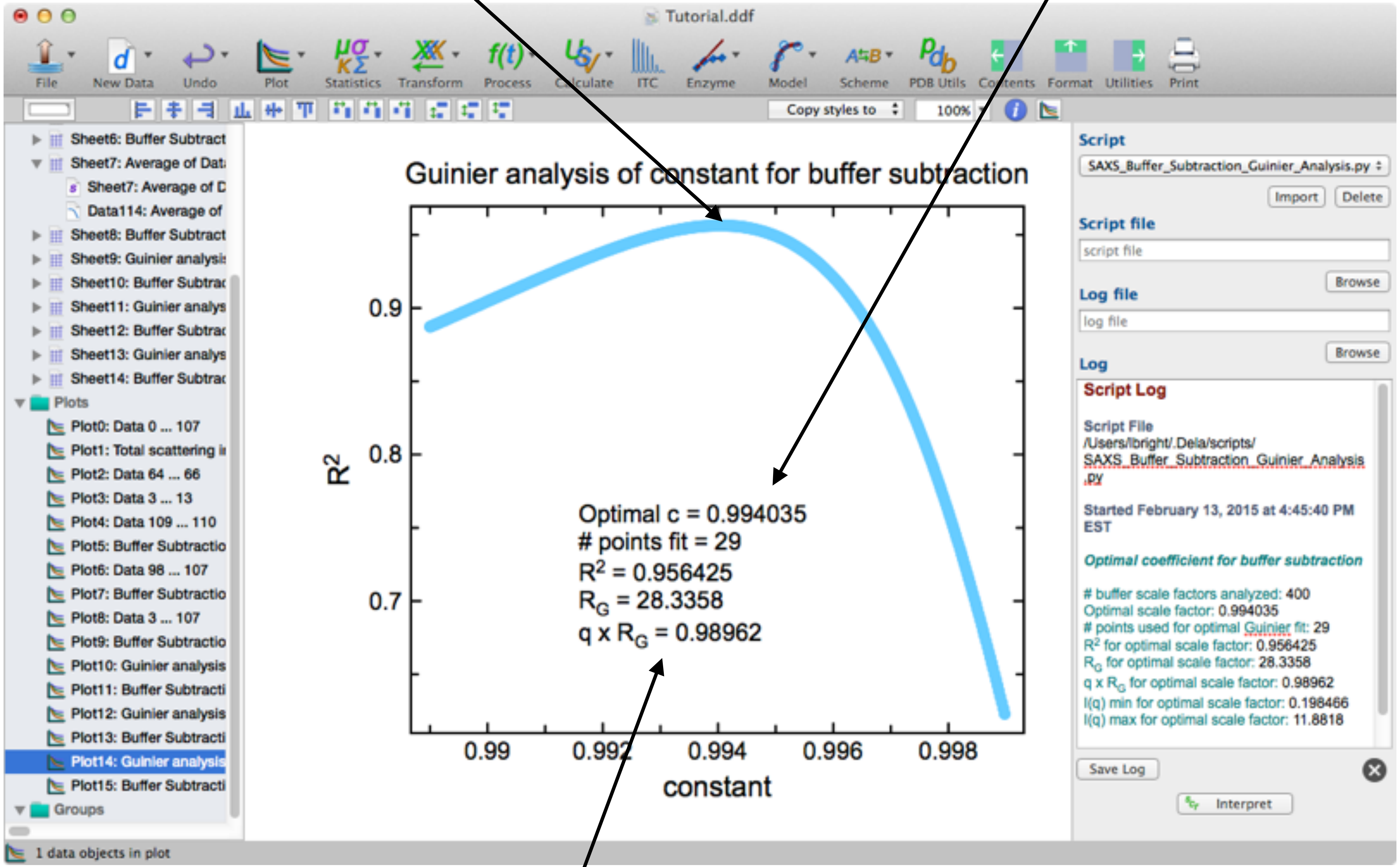

The other info refers to the subtraction with the Guinier optimized scaling constant

Plot of Guinier optimized peak − pre/post-peak on $\log_{10}$ q scale

Minimal negative artifact at low q

High q data with no negative points

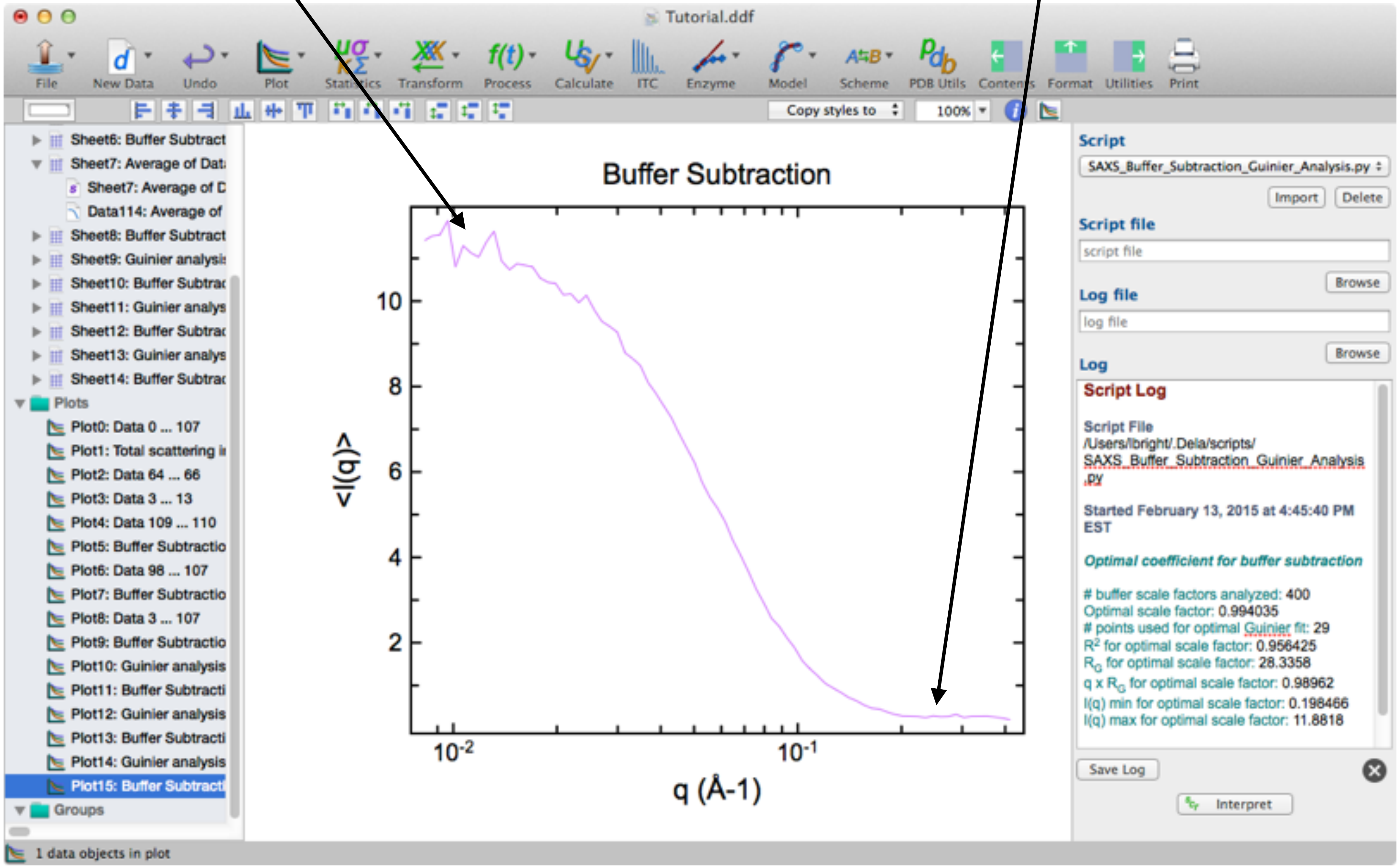

Also looks reasonable. Next try alternative approach using SVD …

Plotting data sets from monomer peak

For SVD, select a region that doesn't overlap too much with adjacent peaks and run SAXS_Plot_Data_With_Indices_In_Selection_Regions.py

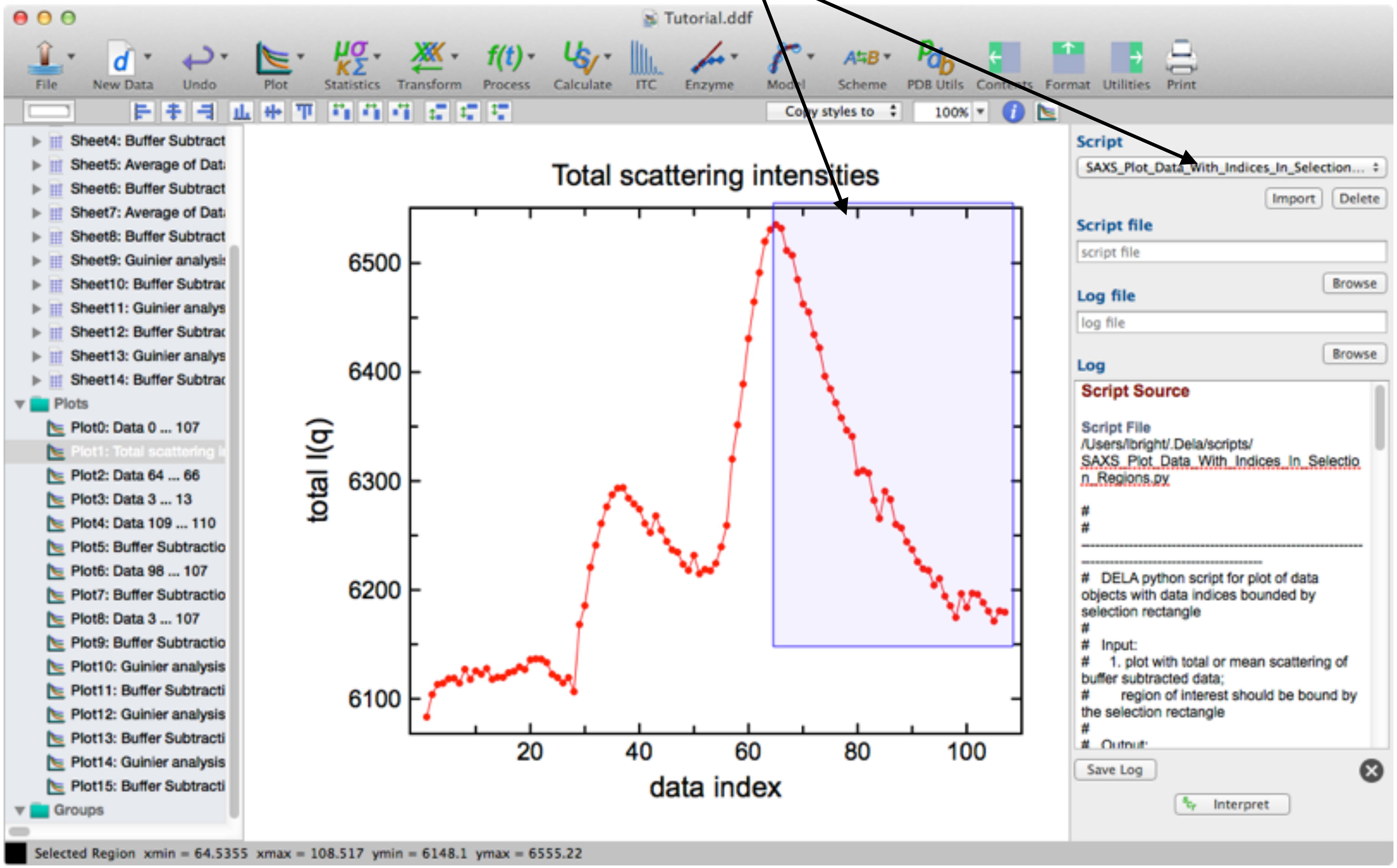

Singular value decomposition

1. Select a plot with scattering data in the contents view

Can be all the data or any combination of regions in the SEC elution profile.

2. Select Singular Value Decomposition from the Calculate tool bar menu

3. Click the calculate button

A sheet will be generated with the top columns of U listed in order of descending significance, followed by the top columns of V premultiplied by the corresponding singular values (also in descending order), the singular values, and finally the autocorrelations of the columns of U and V.

4. Select the singular value and autocorrelation data objects in the contents view and generate separate plots for each

It is helpful to switch the y-axis of the singular value plot to a log scale and display symbols for the various plots to more clearly identify the individual points. Inspect the singular values to determine how many significant components are present (i.e. the number of singular values that clearly stand out from the monotonically decreasing noise components. Significant singular values will also have high autocorrelation values approaching 1 whereas noise components will have low autocorrelation values. Note that the some columns of U may have high autocorrelations but contribute very little to data, so pay particular attention to the singular values and autocorrelations of V.

Plot with data from monomer peak on $\log_{10}$ $\log_{10}$ scale

Select Singular Value Decomposition from the Calculate menu

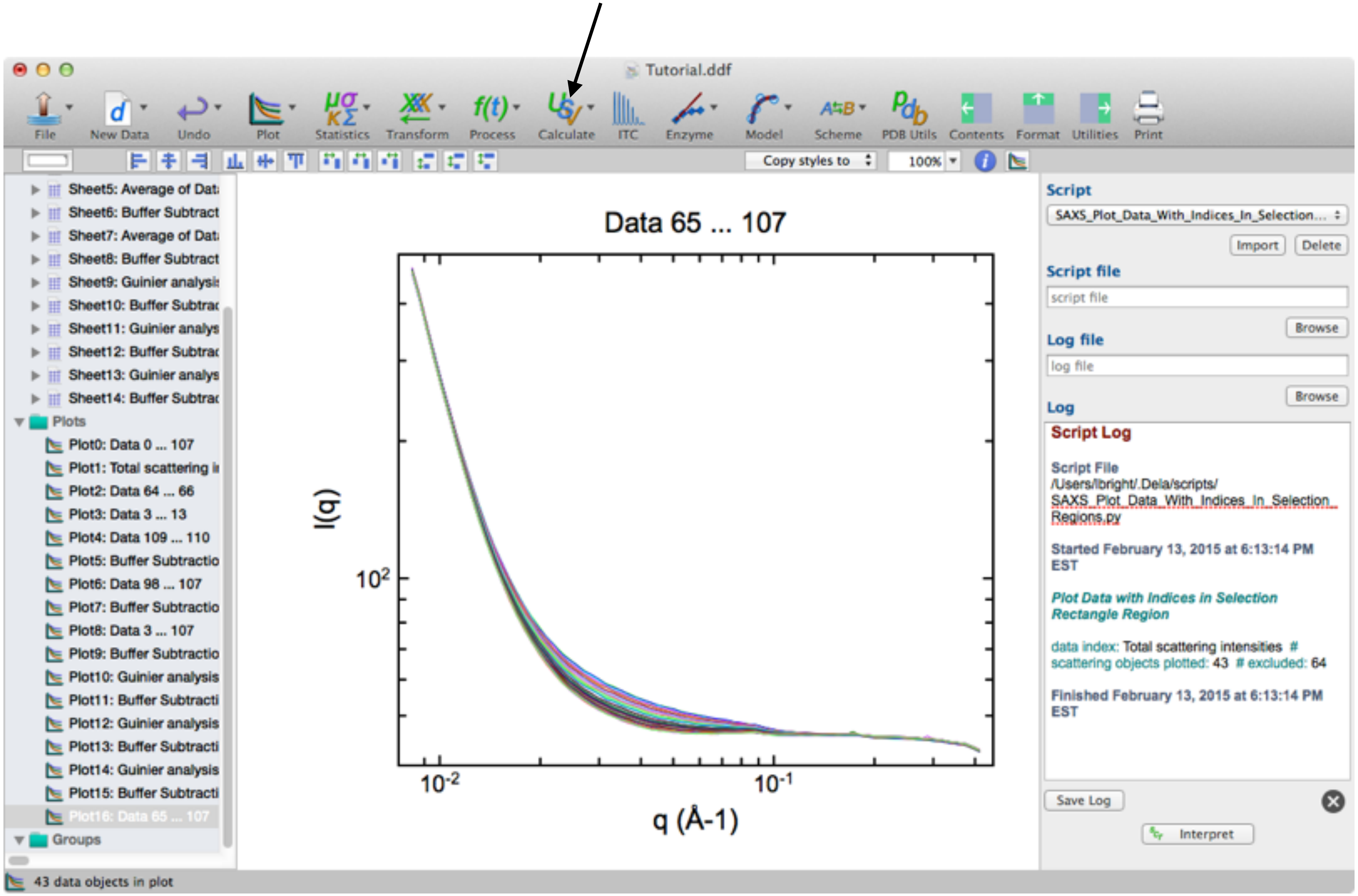

Sheet with SVD components and autocorrelations

1. Select Diagonal Elements and Autocorrelations

2. Select New Plot from the Plot menu

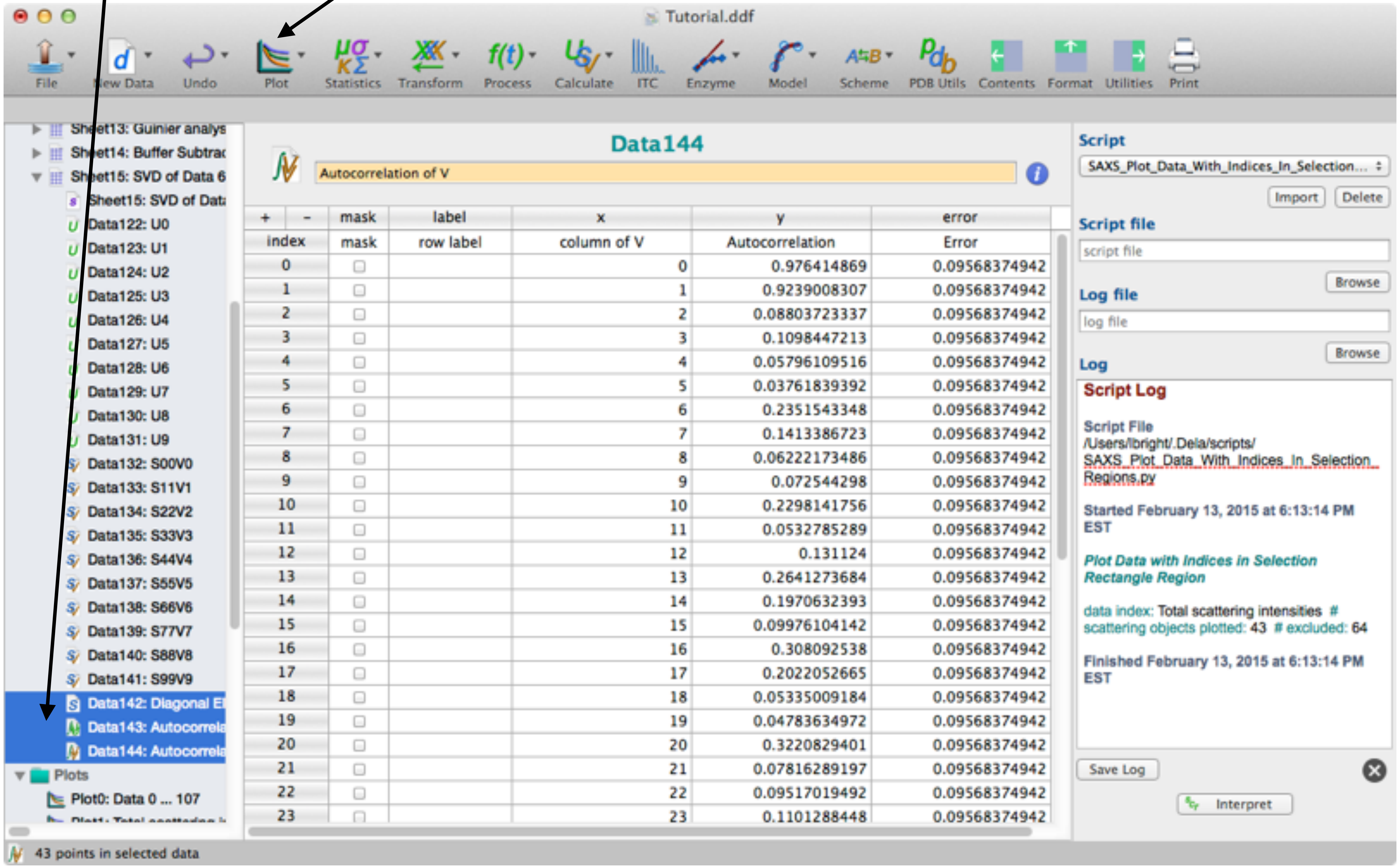

Plot with Singular Values on $\log_{10}$ scale and Autocorrelations on alternate Y axis

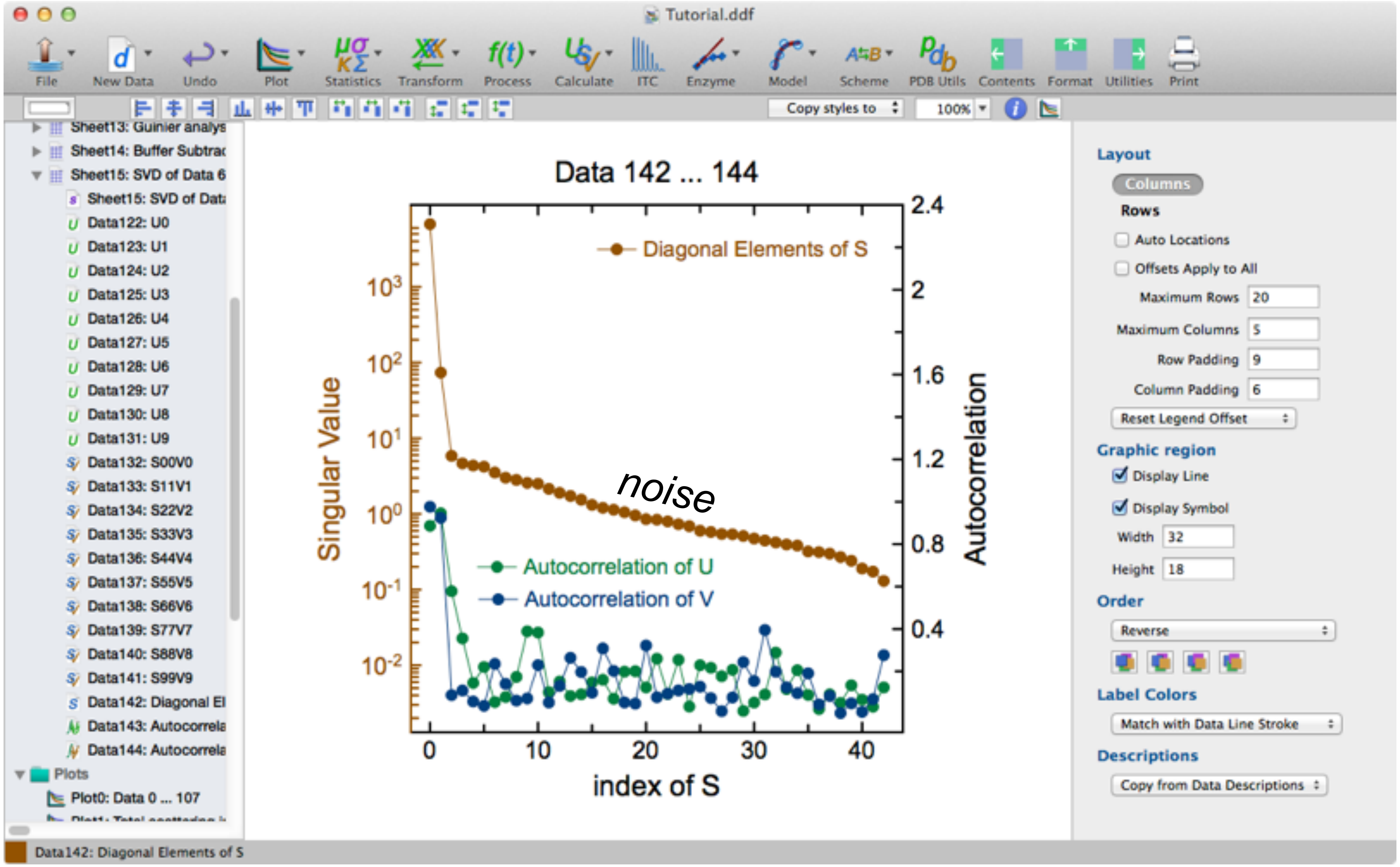

Only U_0 and U_1 have significant Singular Values and Autocorrelations near 1.

In some cases $U_{N>1}$ may also have Autocorrelations approaching 1, but $S_{N>1}$ should be relatively small and the Autocorrelations of $V_{N>1}$ should be << 1.

Plot with Columns of U on $\log_{10}$ q scale

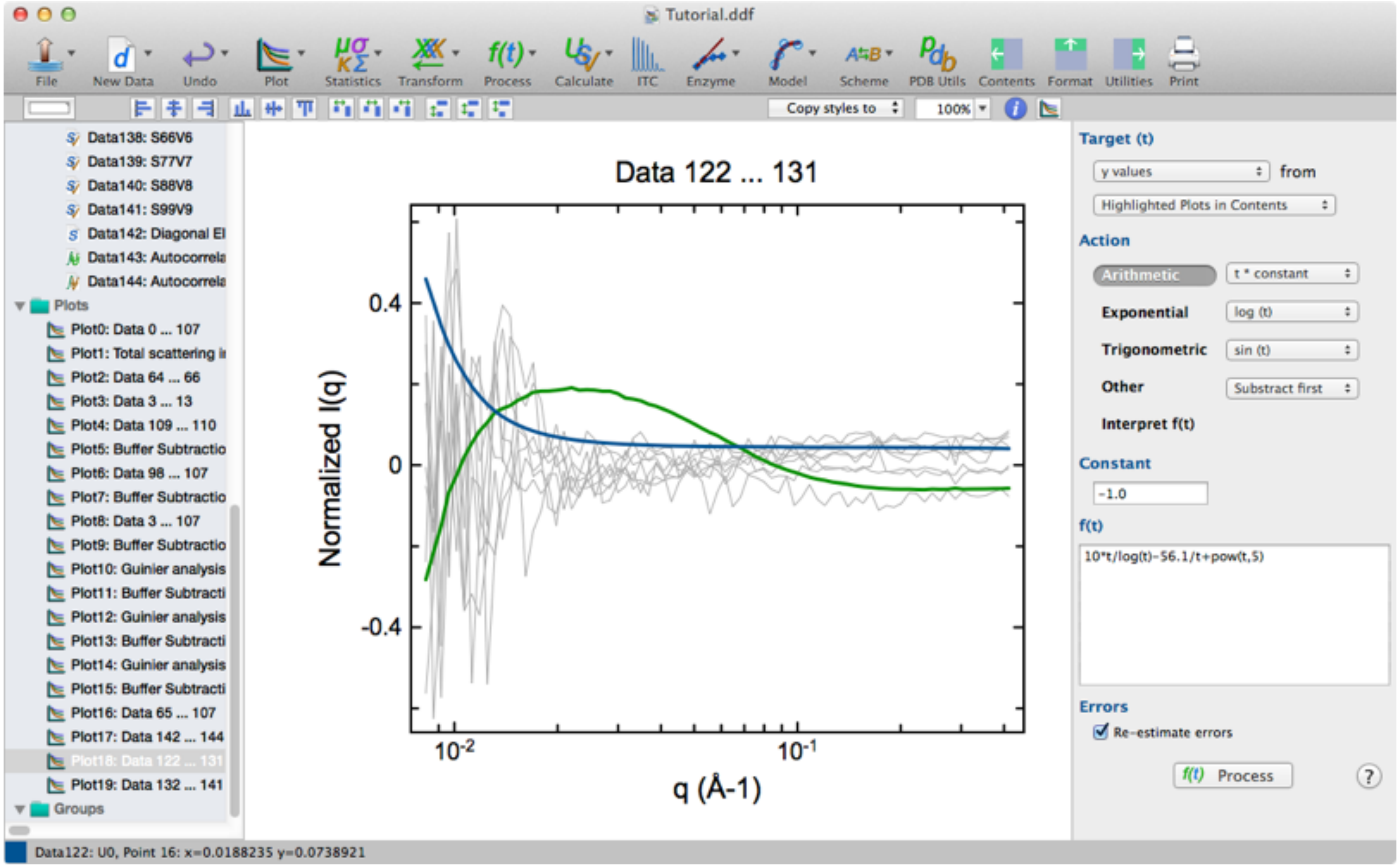

For display, U_0 was multiplied by -1.

Plot with Columns of $S_{nn}V_n$

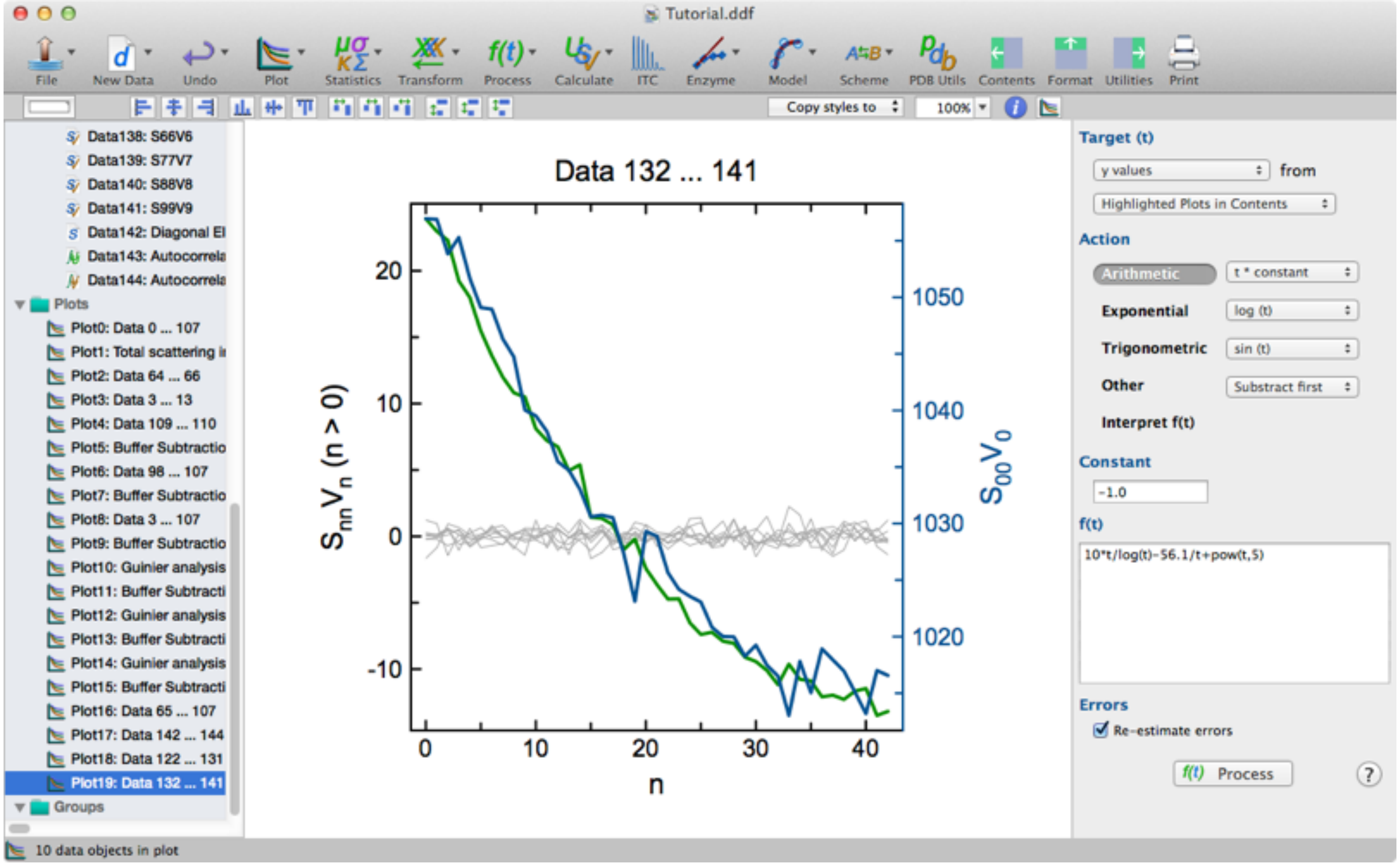

For display, $S_{00}V_0$ was multiplied by -1.

Reconstruction of protein scattering by linear combination

1. Select both the U0 and U1 data objects in the contents view

2. Run the SAXS_Linear_Combination_Guinier_Analysis.py script

It may be desirable to decrease the q x Rg limit (e.g. to 1.0 or 0.8). Lowering the q x Rg limit should in principle give more accurate results and will likely work well if the signal to noise is reasonably good. For cases where the signal to noise is low, a higher cutoff may be necessary.

3. Inspect the Guinier analysis and Linear Combination plots

If the analysis worked, there should be a clear maximum in R^2 towards the middle of the range of linear coefficients for U0. If necessary, the script can be re-run with a different range or different q x Rg cutoff. The linear combination should should look like a typical SAXS curve after buffer subtraction. It may be helpful to use a log scale for the y and/or x axes.

Since the optimization procedure focusses exclusively on the Guinier region, it can not correct for imperfect buffer matching, capillary fouling, etc. These problems presumably manifest as deviations from ideality that are most obvious at high q. Thus, the curves may approach non-physical values less than 0 or values higher than expected at high q. Adding or subtracting a constant to the entire curve may partially correct such deficiencies. This is done by default in many ATSAS programs (e.g. GASBOR, EOM, CORAL, etc) but usually not in dammif if the curve is ‘too short’. Manual constant subtraction can be done in DELA using the Process panel.

Reconstruction of protein scattering by linear combination

4. Select the linear combination plot in the contents view

5. Select Statistics → Estimate Errors in the tool bar

This is an important step, since errors are not propagated through SVD. Non-uniform polynomial error estimation with a window size of 11 and order of 2 appears to give reasonable results for log sampled data sets with roughly 80 points. The window size may need to be adjusted depending on the number of points in the data set.

Reconstruction of protein scattering by linear combination

1. Select U_0 and U_1

2. Run SAXS_Linear_Combination_Guinier_Analysis.py

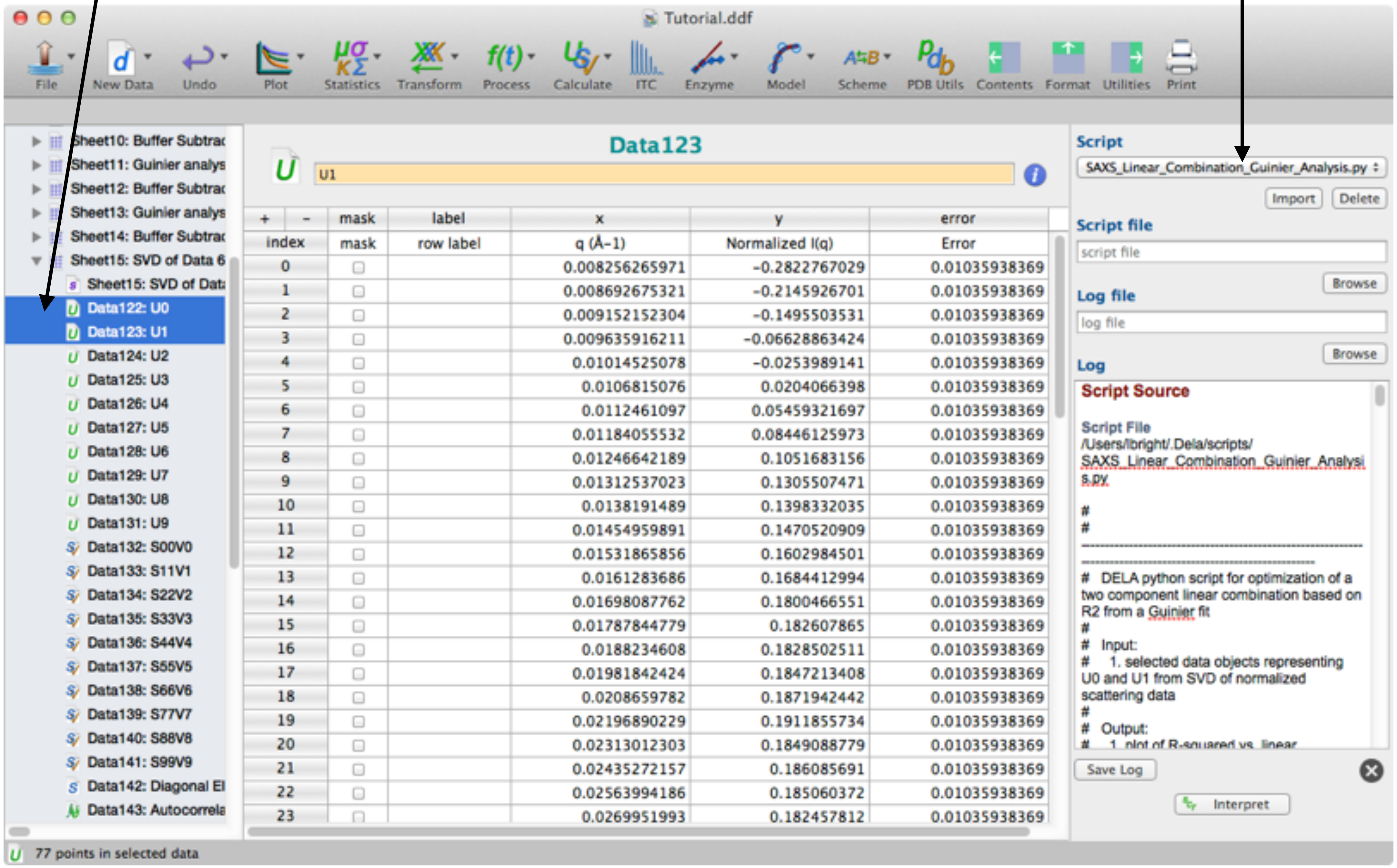

Reconstruction of protein scattering by linear combination

Clear maximum in R^2 vs. linear coefficient

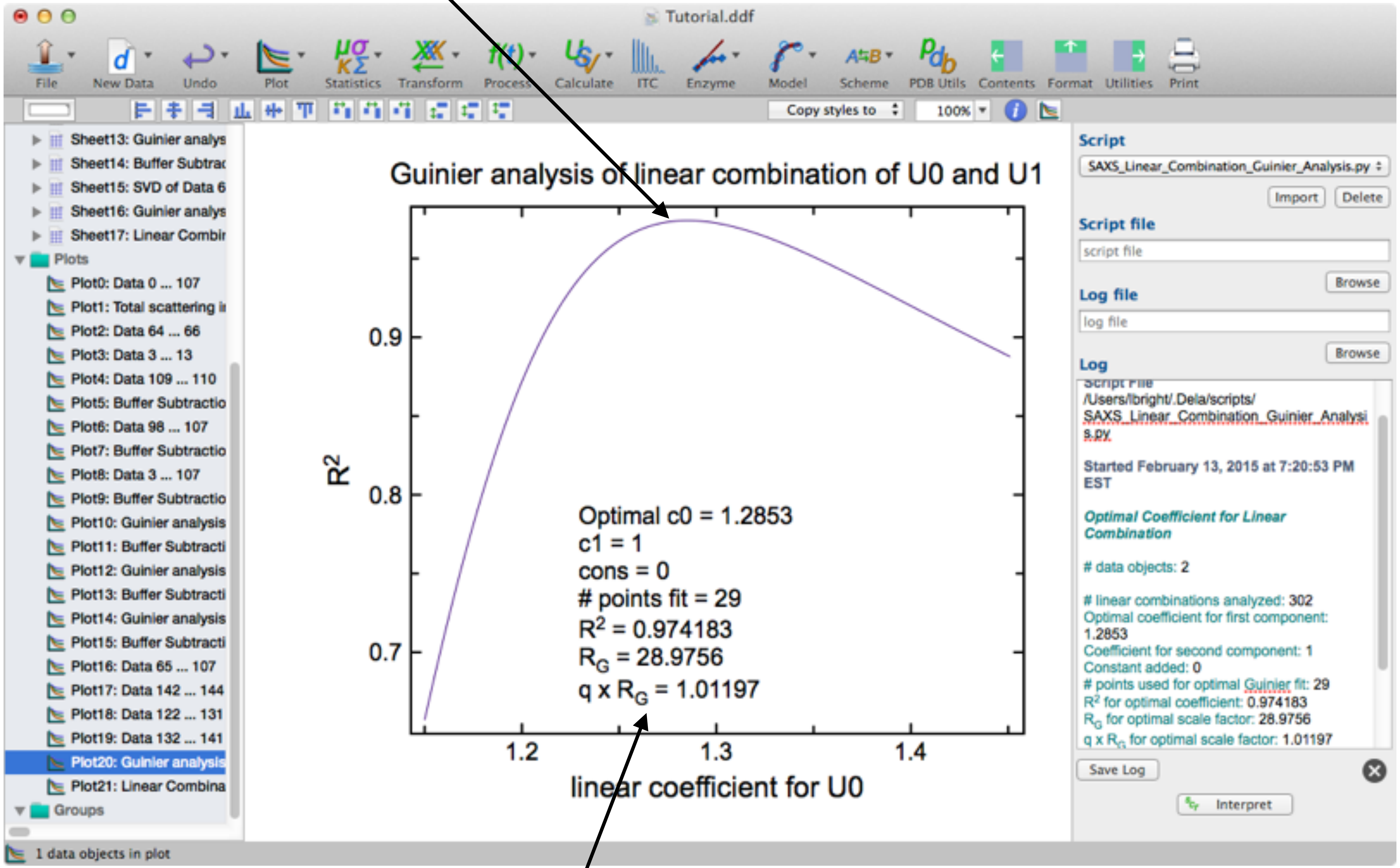

The other info refers to the subtraction with the Guinier optimized linear coefficient

Reconstruction of protein scattering by linear combination

Minimal negative artifact at low q

Slightly negative in high q region

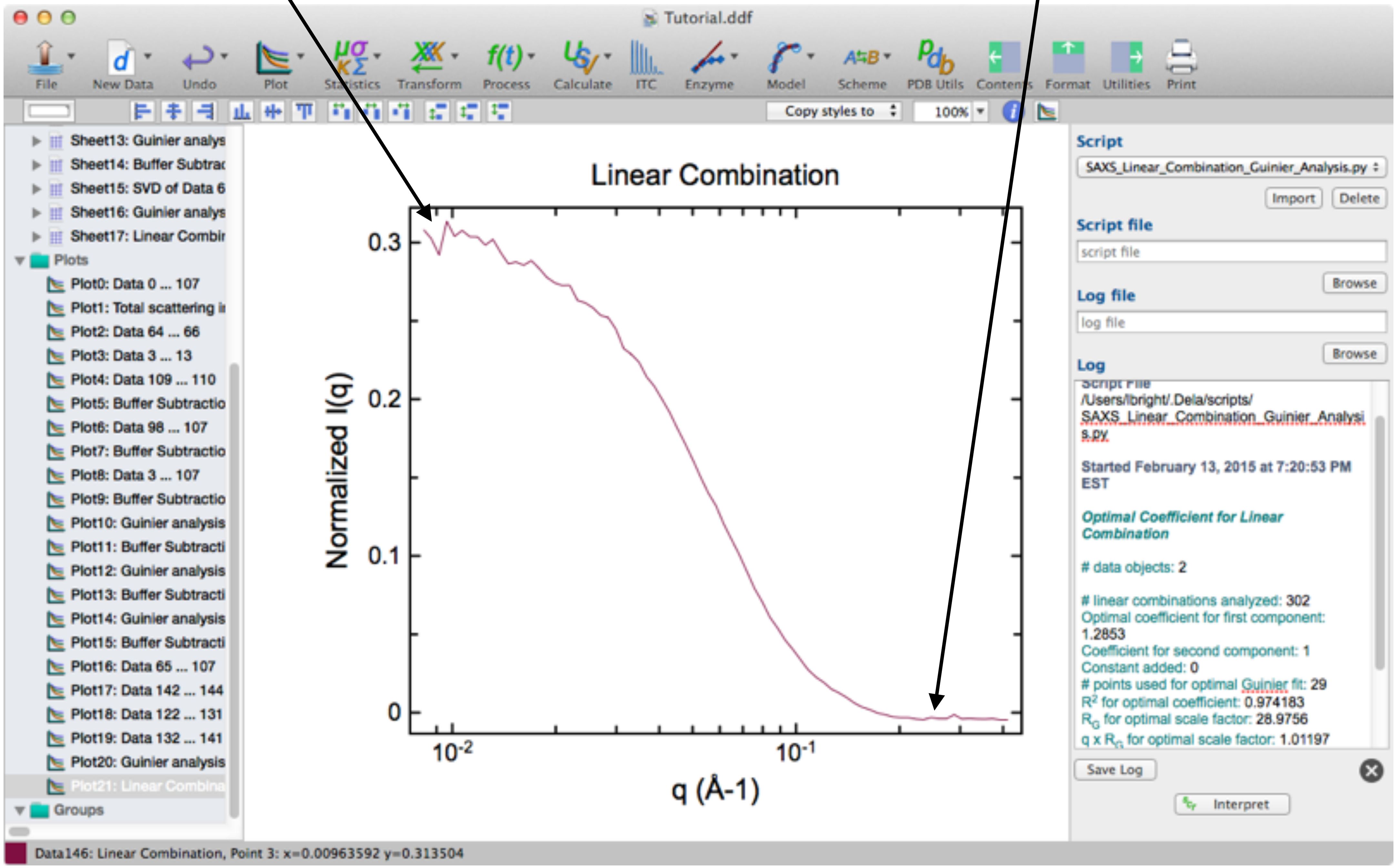

Also looks like a reasonable result. Next compare with buffer subtraction …

Estimate errors for SVD-LC reconstruction

1. Select plot with SVD-LC reconstruction

2. Select Statistics → Estimate Errors

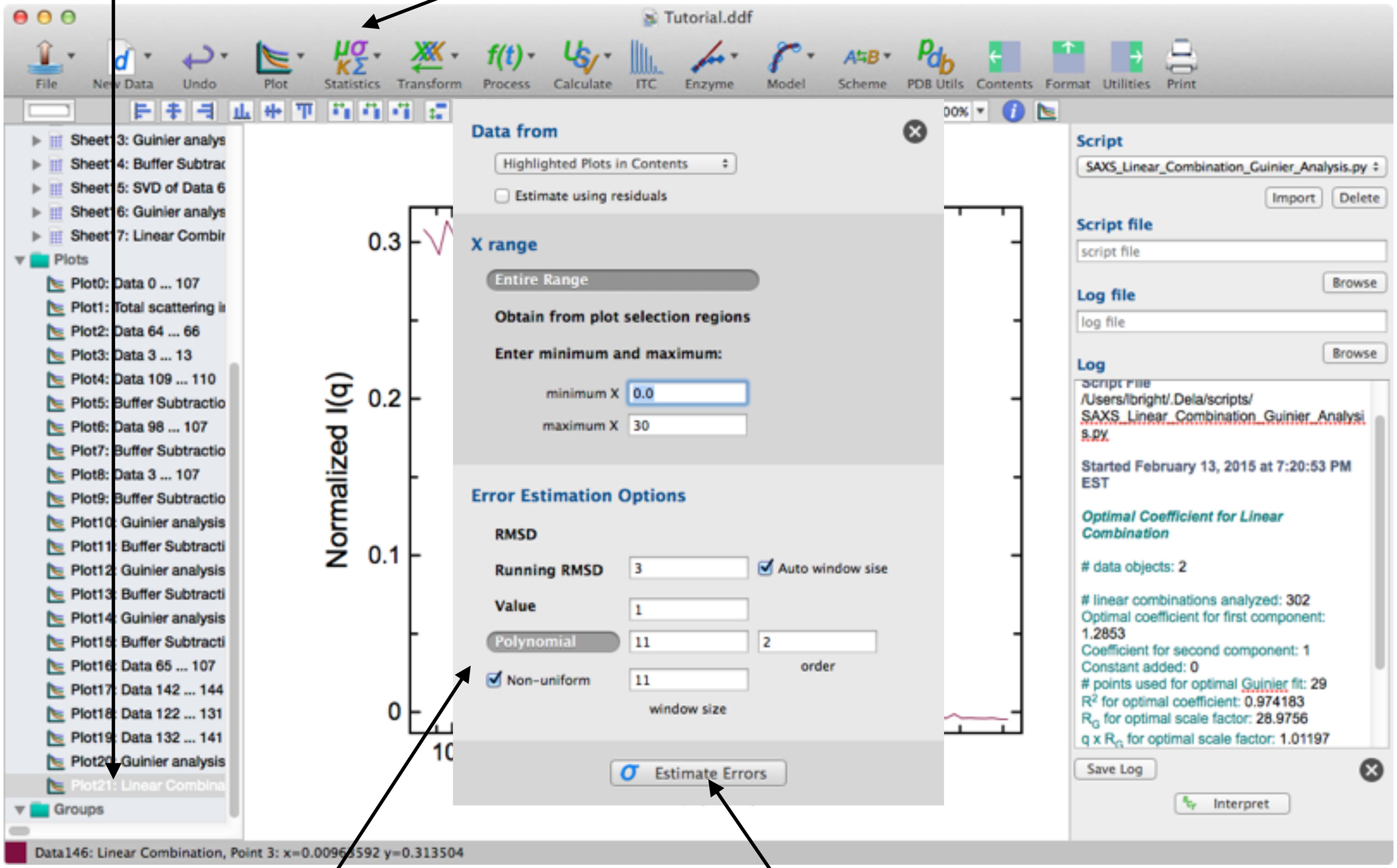

3. Reasonable settings for smoothly varying data with ~80 points

4. Click Estimate Errors

Comparison of SVD-LC with buffer subtraction

1. Select plots with direct or Guinier optimized buffer subtraction or SVD-LC

2. Select New Plot from the Plot menu

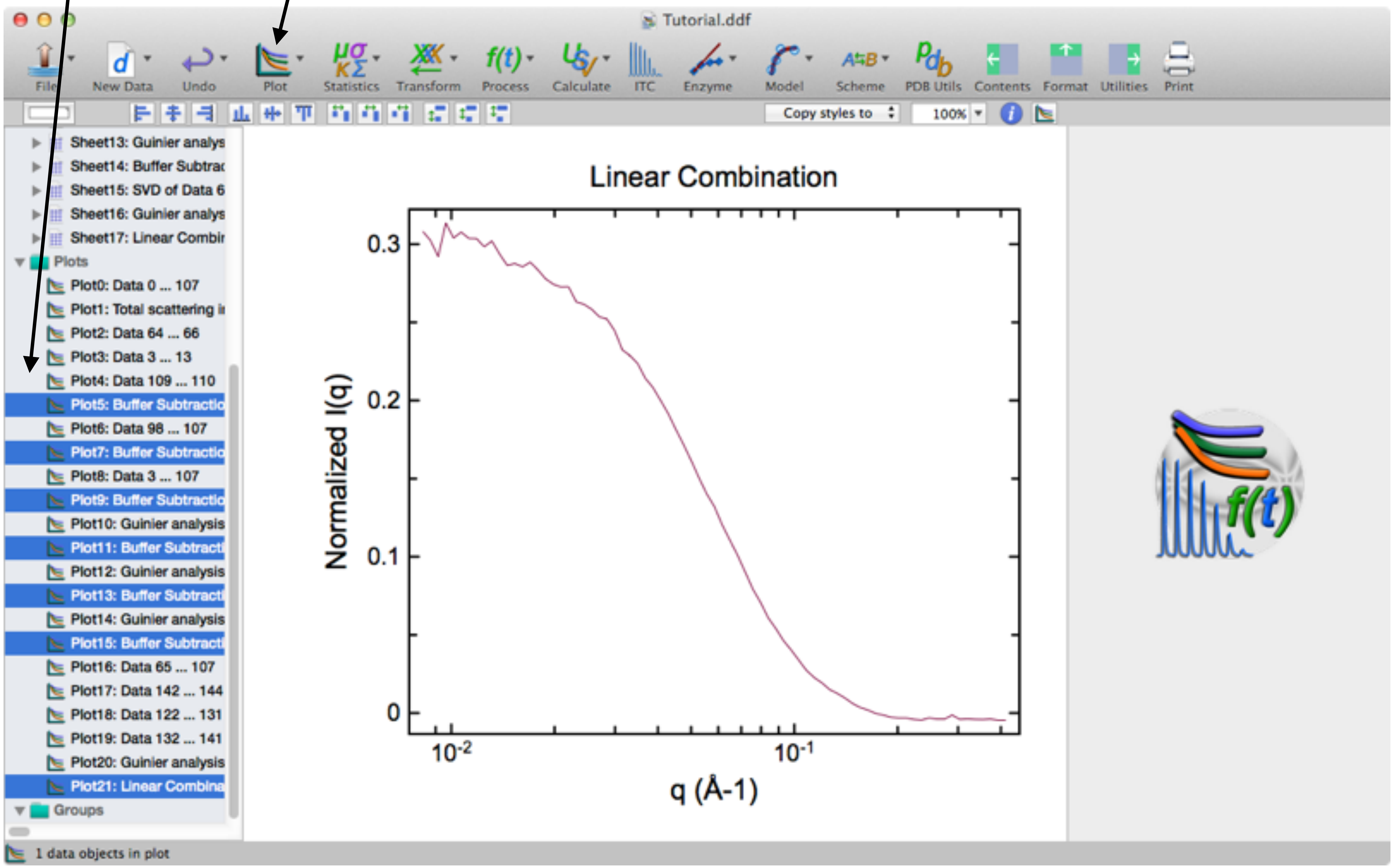

Comparison of SVD-LC with buffer subtraction

Select Calculate → General Linear Least Squares to scale to the most representative curve (though not necessarily the best!)

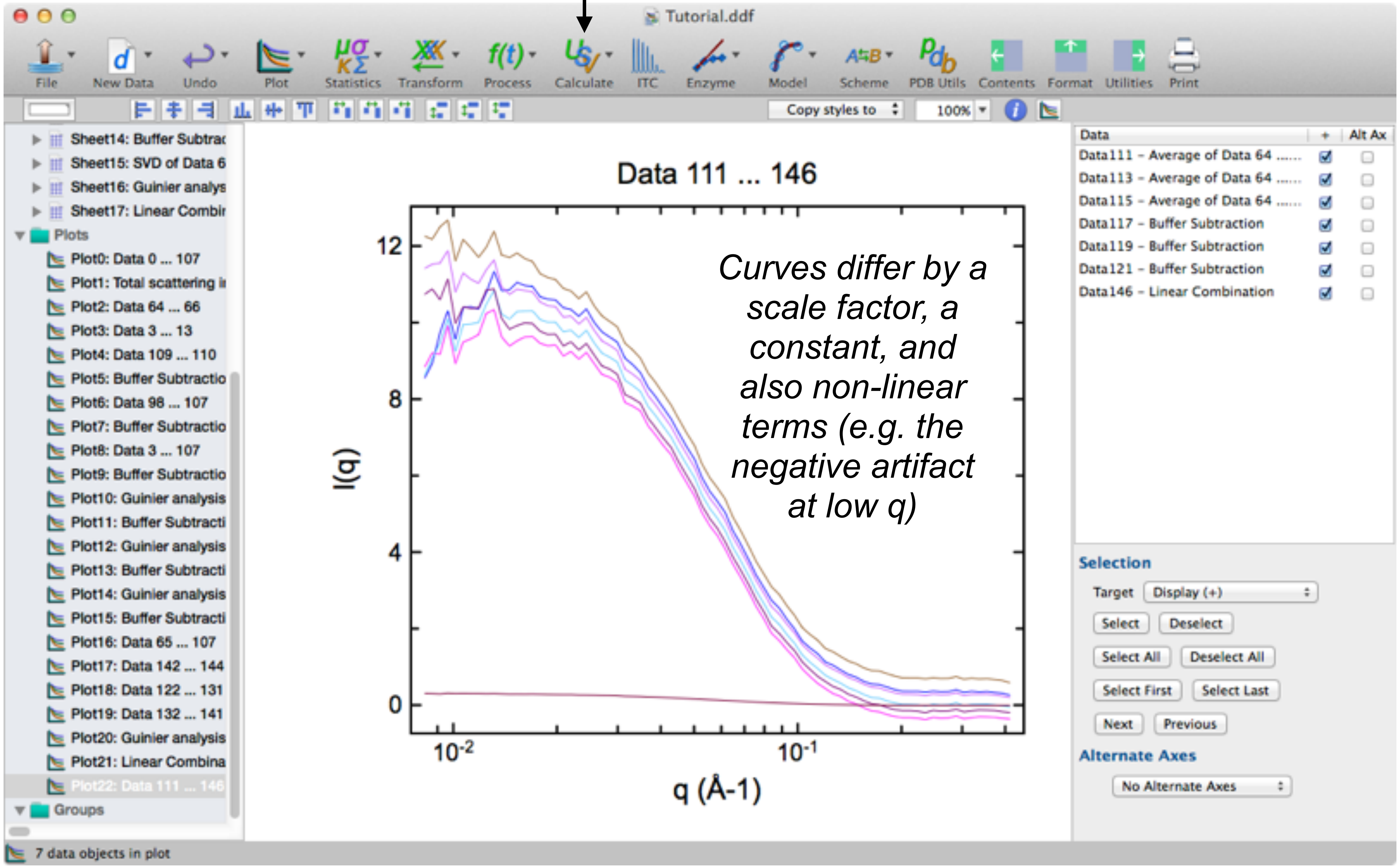

The curves could also be duplicated before GLLS to preserve the original scale/constant.

Select the data objects in the contents view and select Duplicate Highlighted from the right mouse button menu.

Comparison of SVD-LC with buffer subtraction

Toggle display of curves to compare particular combinations

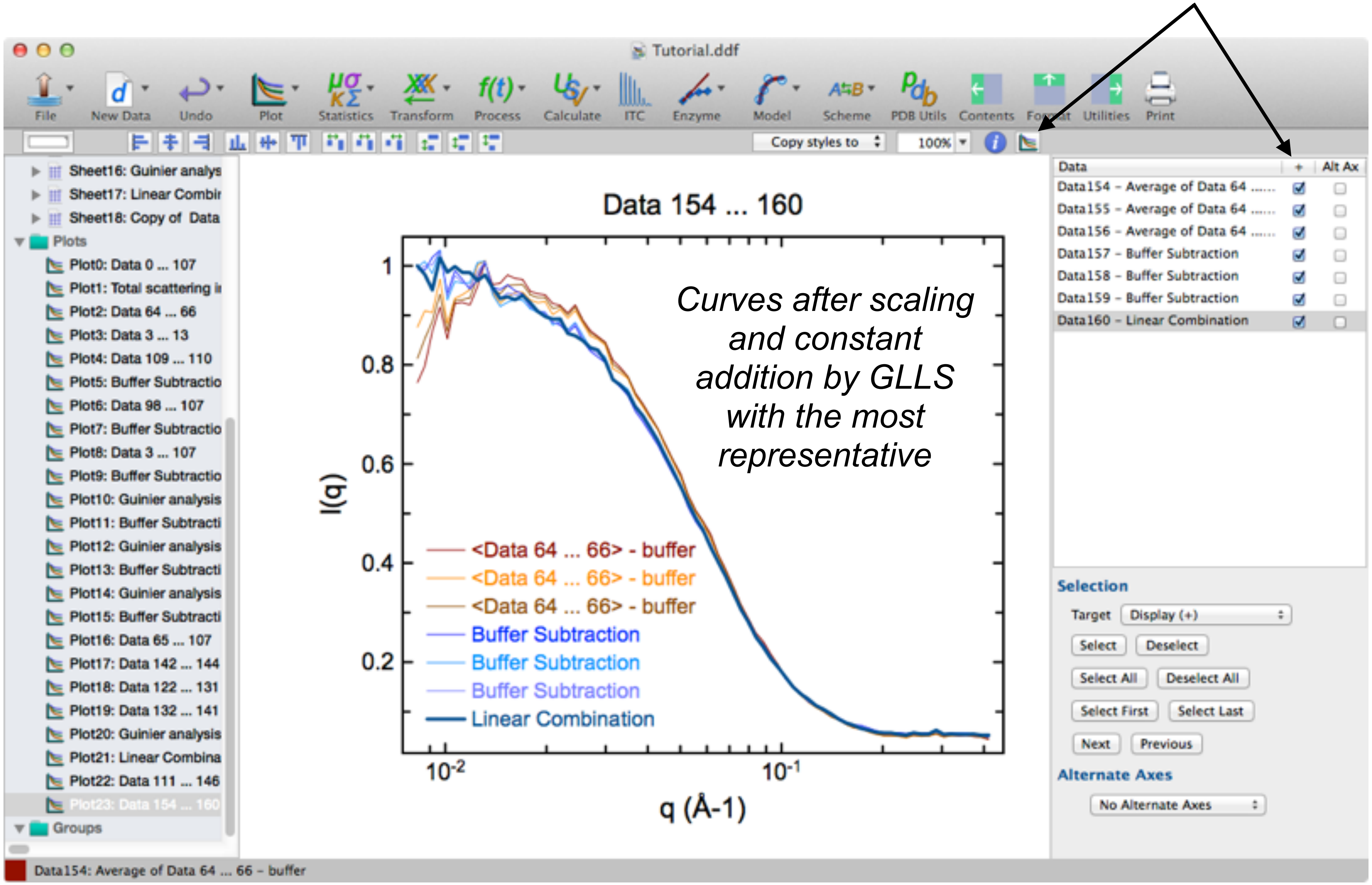

After GLLS, the SVD-LC and Guinier-optimized curves (blue colors) are very similar except that the SVD-LC reconstruction has higher signal-to-noise. The direct subtractions (red/brown/orange) differ primarily in the negative artifact at low q.

Comparison of SVD-LC with buffer subtraction

Same comparison with I(q) axis also on Log_{10} scale

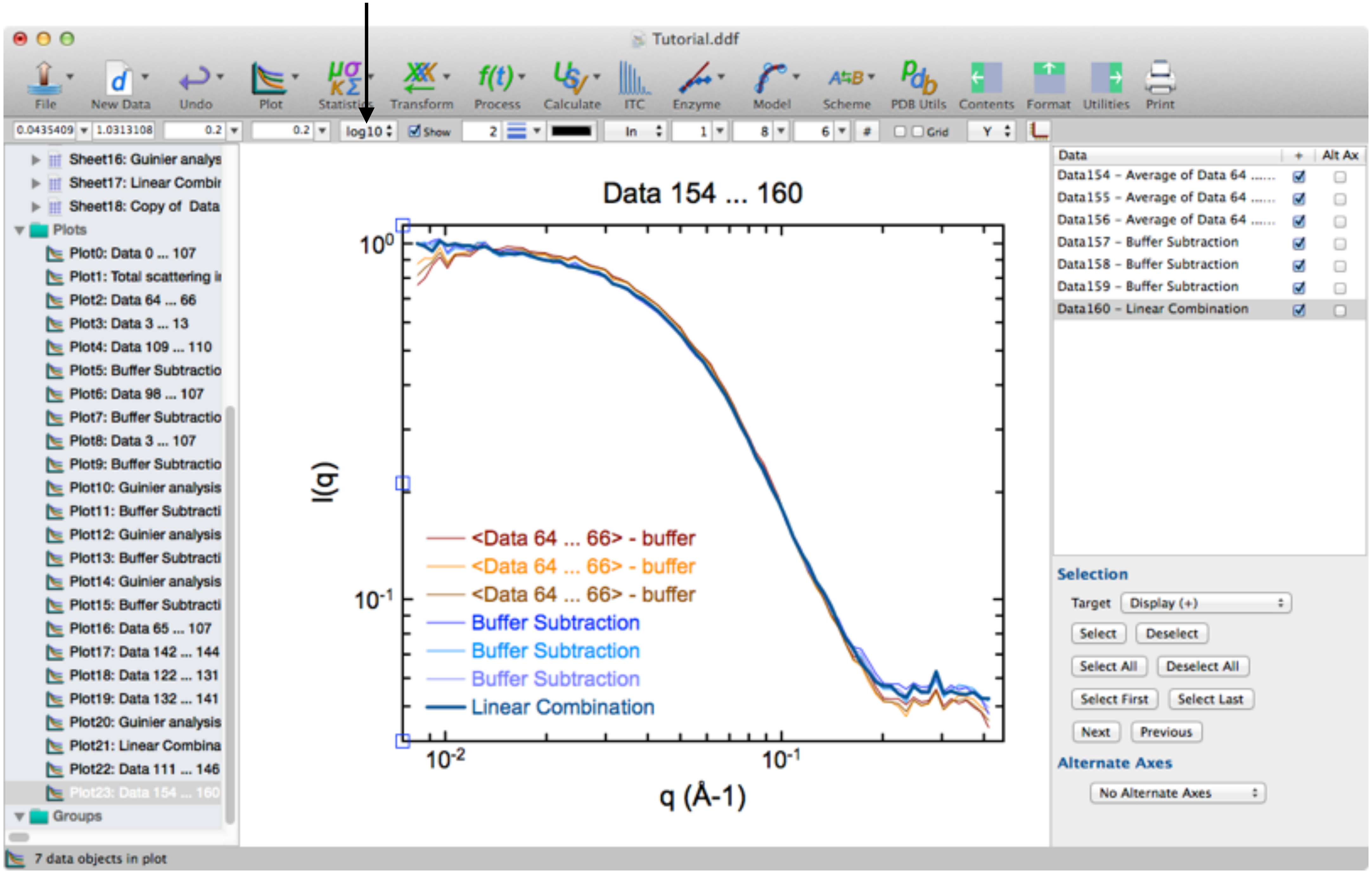

Comparison of SVD-LC with buffer subtraction

Same comparison after GLLS based on data in selection region

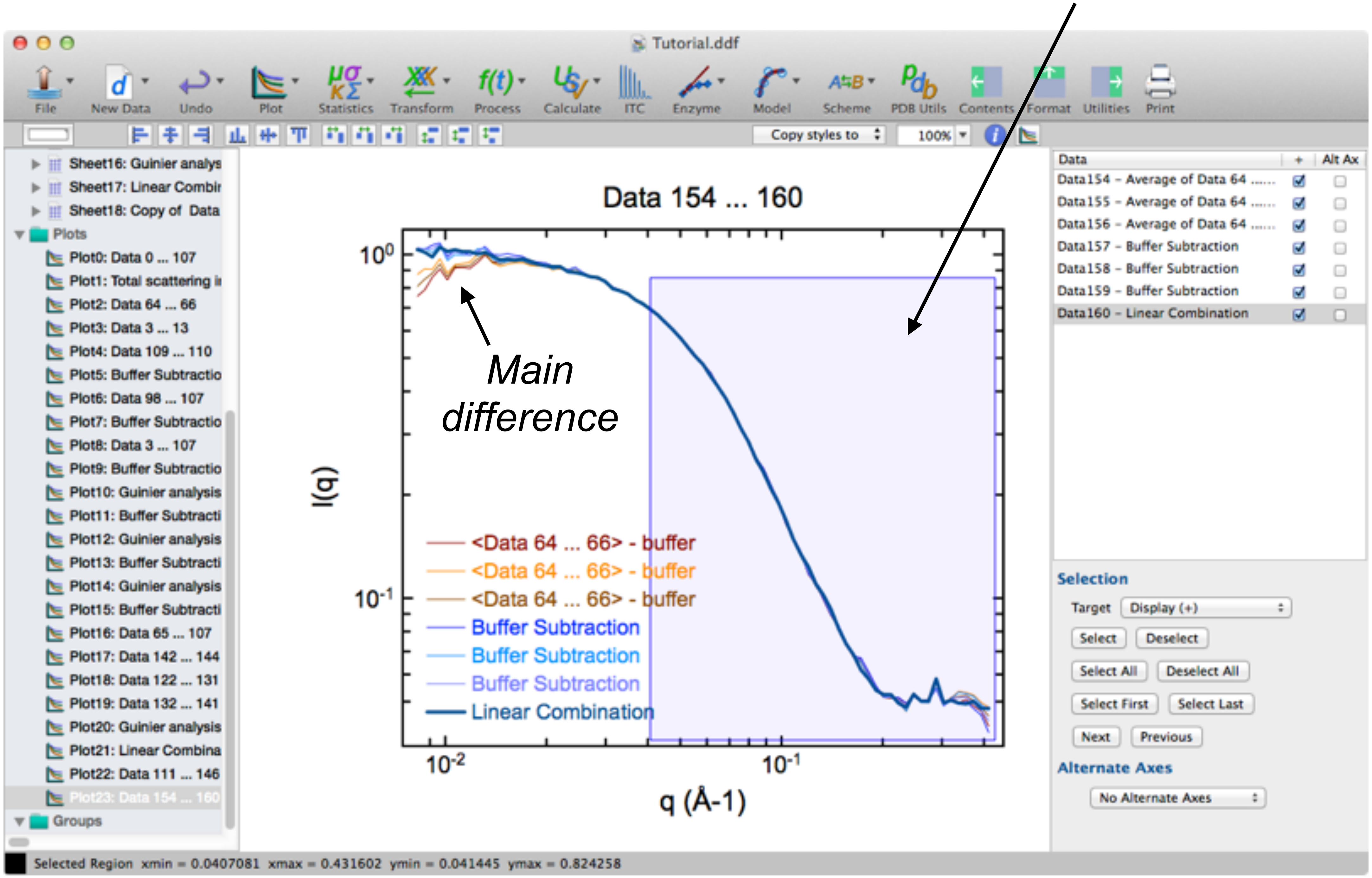

Evidently the GLLS scaled/constant added curves are all very similar at q > 0.02 Å^{-1}. Apart from scaling and a constant, the main difference is the negative subtraction artifact at q < 0.02 Å^{-1} in the direct subtraction curves.

Comparison with CRYSOL model

Import model from CRYSOL fit to any of the curves, drag data object from CRYSOL model into the plot, and scale/add constant with GLLS

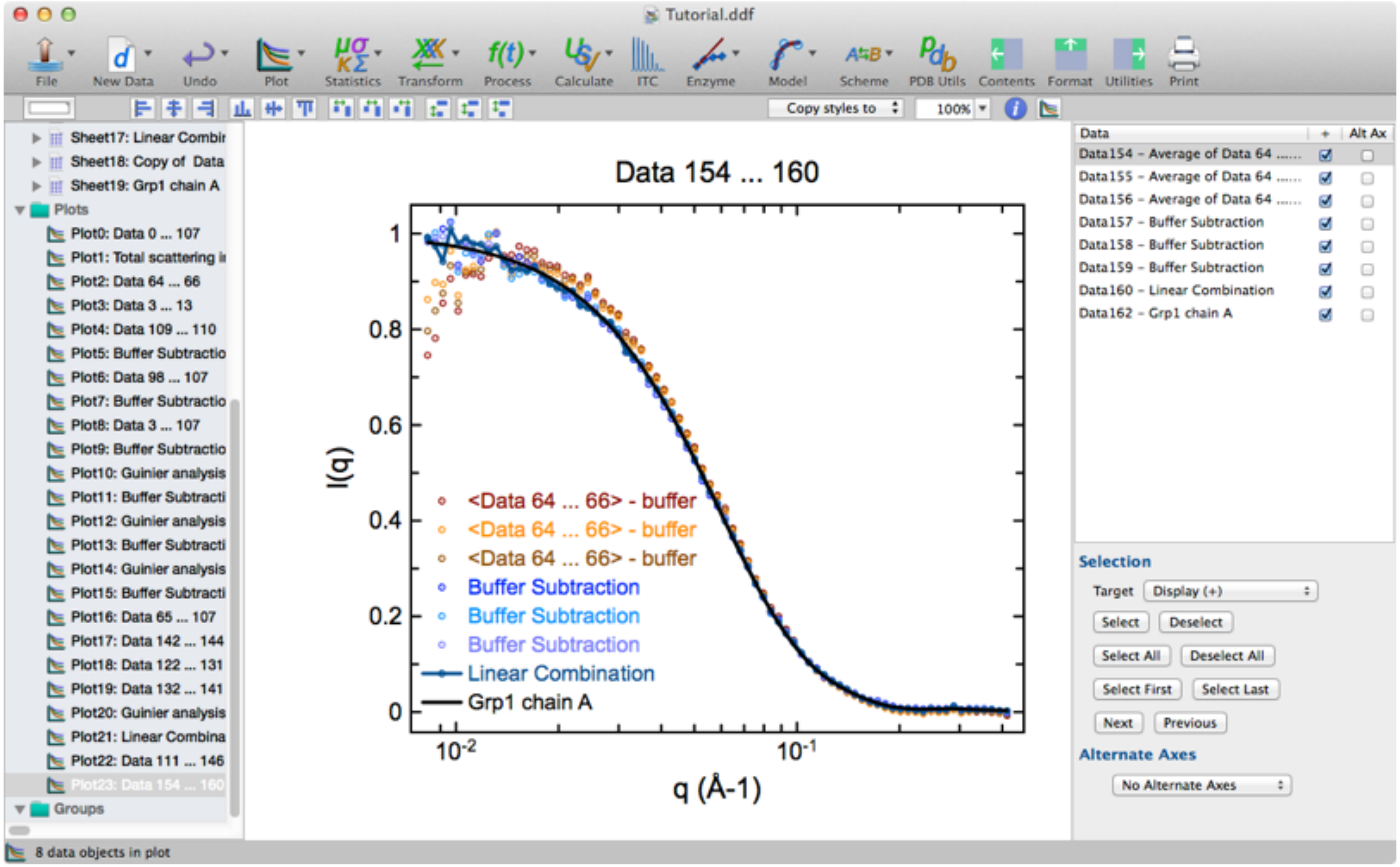

After scaling/adding constant over the full q range based on the CRYSOL model, the agreement with the SVD-LC and Guinier optimized subtractions is excellent.

Calculating and Displaying Residuals for the CRYSOL model

1. Note Data Label for the CRYSOL model

2. Select New Data → New Model Data

3. Select Model → Select → Linear Combination of Data

4. Calculate the model

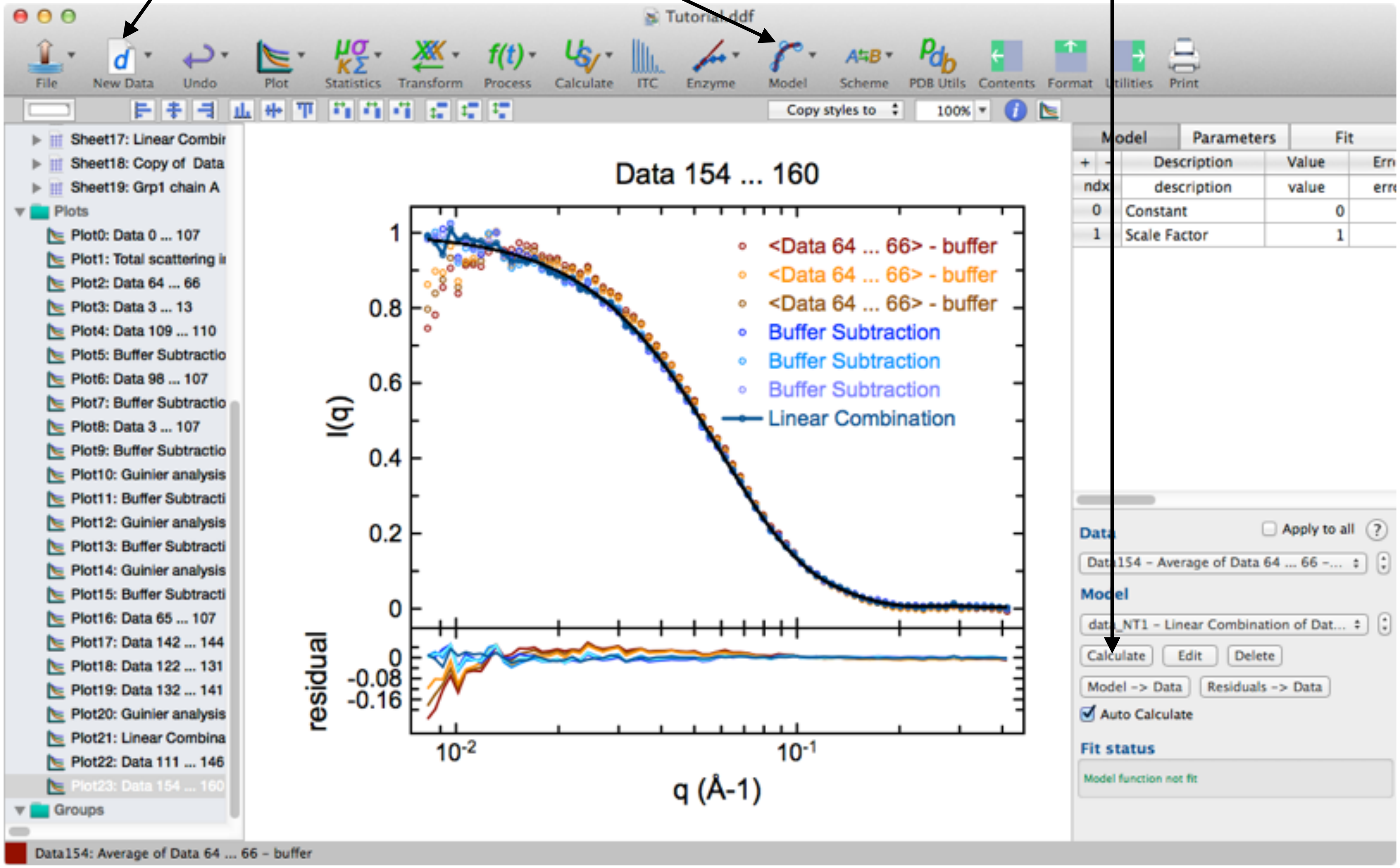

5. Select Right mouse button → Display → Residuals

Guinier plots, fits and profile

The following scripts generate Guinier plots and/or fits:

1. SAXS_Guinier_Plot.py
   Guinier plot of log I(q) vs. q^2. Select a data object, sheet or plot with protein scattering before running the script.

2. SAXS_Guinier_Plot_Fit.py
   Guinier plot of log I(q) vs. q^2 and linear least squares fit subject to a $q \times R_G$ cutoff. Select a data object, sheet or plot with protein scattering before running the script.

3. SAXS_Guinier_Fit.py
   Linear least squares fit subject to a $q \times R_G$ cutoff. Select a Guinier plot in the document contents before running the script.

4. SAXS_Guinier_Fit_with_defaults.py
   Linear least squares fit using default options. Useful for fast iterative manual fitting. Select a Guinier plot in the document contents before running the script.

5. SAXS_Guinier_Profile.py
   Profile of Guinier fits subject to a $q \times R_G$ cutoff for a range of protein scattering data (e.g. for an SEC-SAXS elution profile or concentration series). Select a plot or sheet containing the protein scattering data before running the script.

I vs. Q, Kratky, Porod, Log-Log and GNOM P(r) plots

The following scripts generate common SAXS plots:

1. SAXS_IvsQ_Plot.py
   Convenience script for plotting I(q) vs. q with formatted axis labels.

2. SAXS_Kratky_Plot.py
   Kratky plot of q^2 I(q) vs. q.

3. SAXS_Porod_Plot.py
   Porod plot of q^4 I(q) vs. q.

4. SAXS_Log_Log_Plot.py
   Convenience script for plotting log I(q) vs. log q with formatted axis labels.

5. SAXS_Log_Log_Plot.py
   Convenience script for plotting GNOM P(r) distributions from GNOM .out files.

Select a data object, sheet or plot with protein scattering before running the scripts.

Calculating a Maximum Entropy P(r) distribution

1. Select a plot with protein scattering data

2. Select Calculate → Maximum Entropy

3. Select Pair Distribution

5. Click Fit

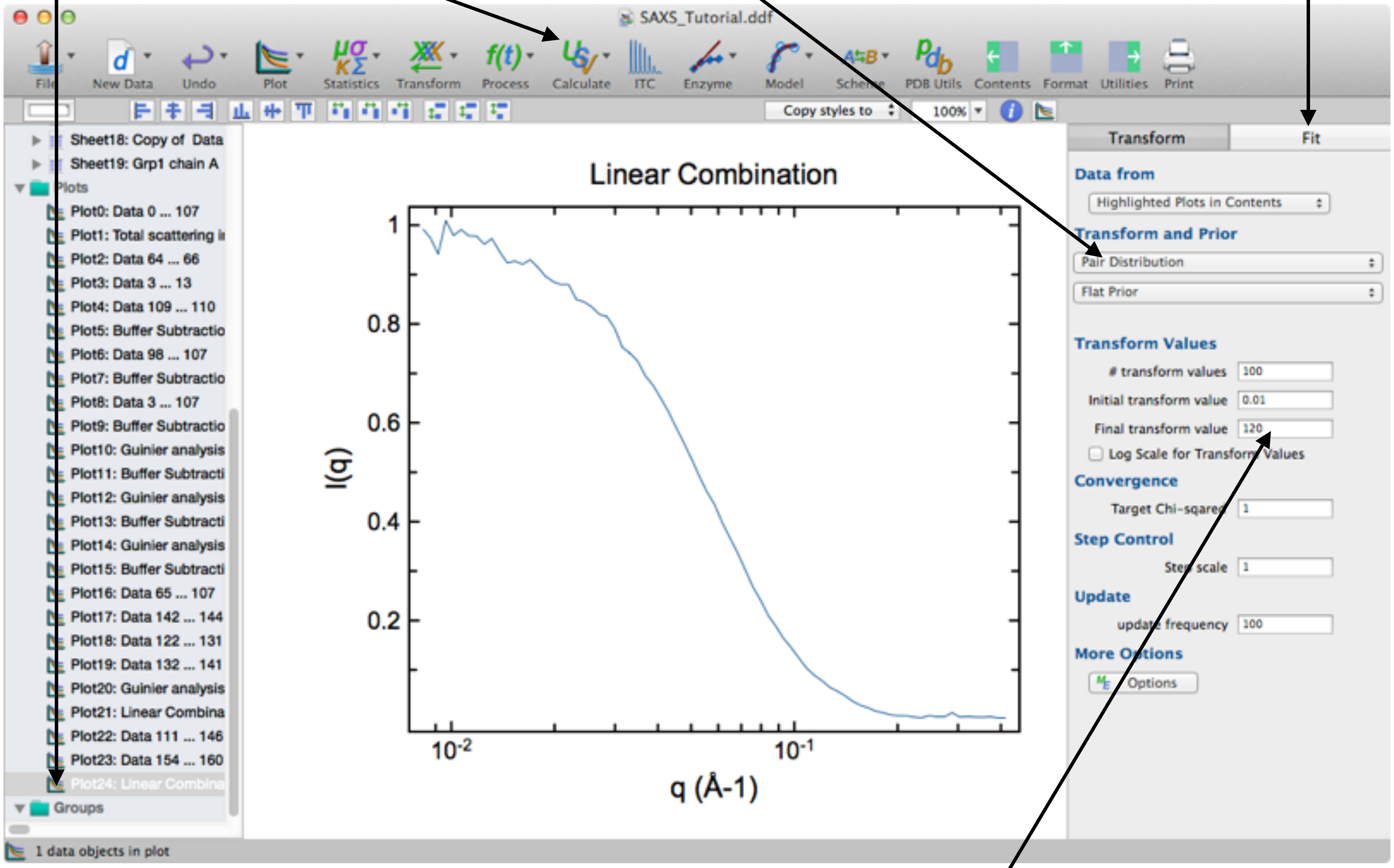

4. Set the final transform value a little large than the expected Dmax

Calculating a Maximum Entropy P(r) distribution

1. Click on Calculate

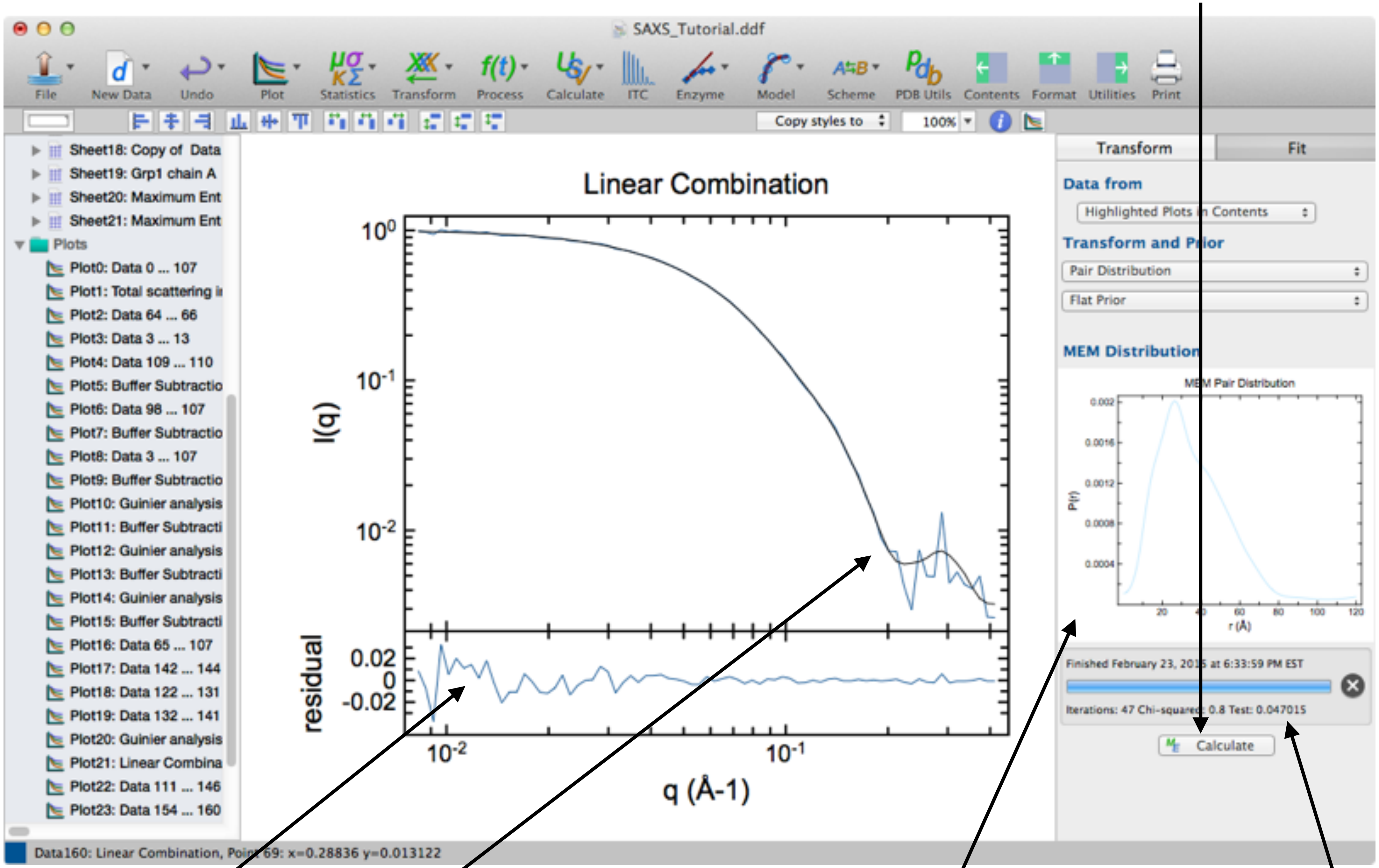

2. Flat residuals?

3. Model fits data but not noise?

3. Reasonable distribution?

not too peaky and tails approach zero

4. Test < ~0.1?

Normalization by incident and/or transmitted beam intensities

The following slides were developed in the context of APS BioCAT beam line and explain how to:

1. import incident and transmitted beam intensities from a .log file

2. undo previous normalization if any

3. explore/compare normalization options using a total (or mean) scattering profile

4. normalize unnormalized scattering curves

Normalization is beam line dependent. Some beam lines measure both the incident and transmitted beam intensities, while others measure only the transmitted intensity. Normalization by the transmitted intensity is standard. Normalization by I0 is also used for dilute sample with minimal absorbance. More complex normalization schemes including by both incident and transmitted beam intensities can be applied to total (or mean) scattering profiles, and may improve appearance and interpretability by reducing normalization related artifacts. The choice of normalization scheme should have little or no effect on Guinier optimized reconstructions/buffer subtractions (apart from the value of the linear coefficient/scaling constant), and may or may not improve direct buffer subtraction.

Import incident (I0) and transmitted (I1) beam intensities

1. Drag the .log file from a Finder window and drop it onto the main view of the document window – OR – select Import from tool bar File menu

2. Select Unknown from the Data Type menu

3. Under Header, check the following: 1) Has data labels; 2) Has row labels; 3) Ignore (the row labels). Under Columns select Y … (i.e. a series of Y columns)

4. Click the import button.

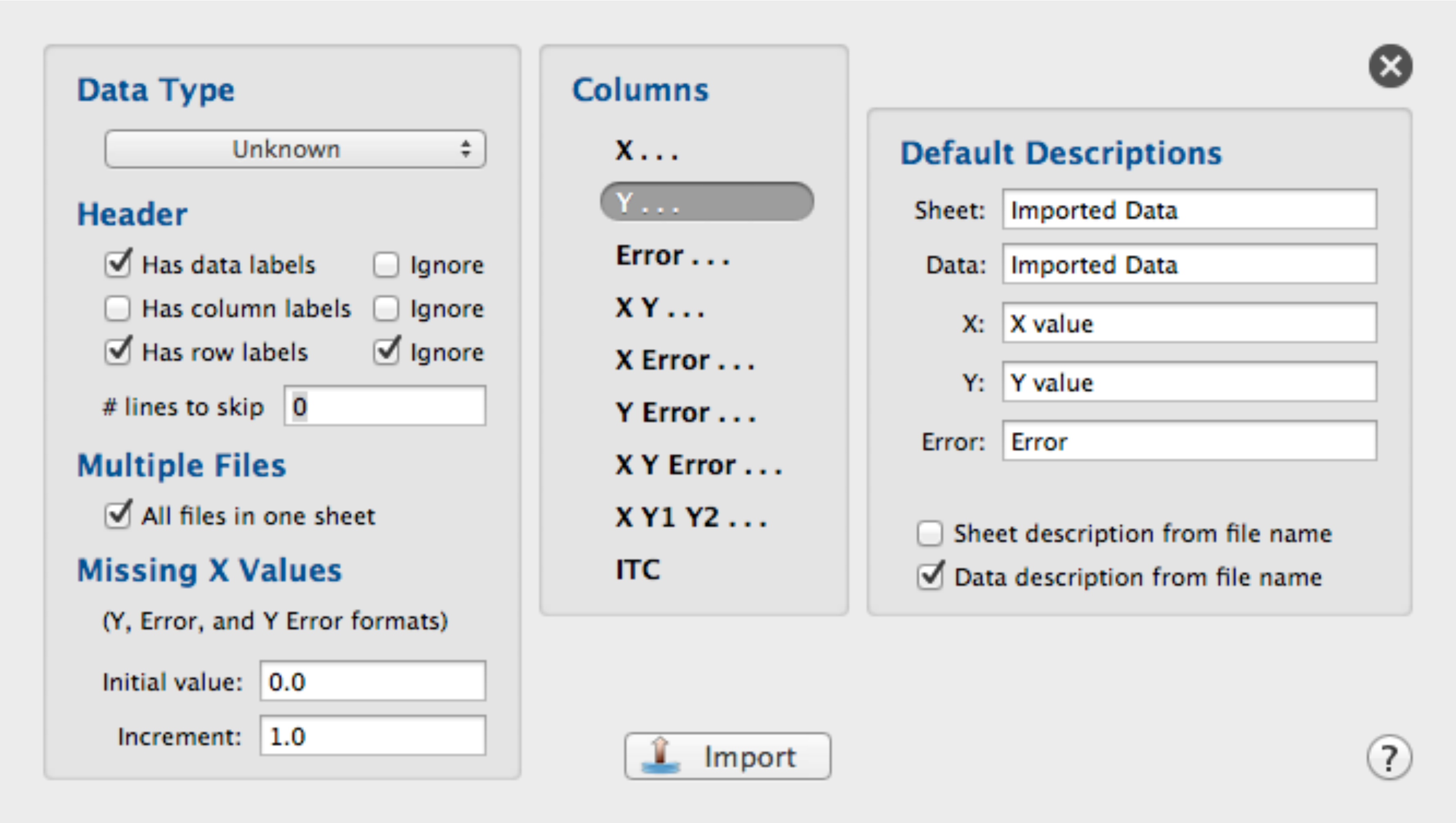

Plot the incident (I0) and transmitted (I1) beam intensities

1. Select the new sheet with the beam intensities and generate a new plot

2. Edit the sheet with the beam intensities and delete any rows that correspond to data objects that were deleted in the plot of the scattering data

It is important to exactly preserve the relationship between the sequence of scattering data objects in the plot of scattering intensities and the sequence of points in I0 and I1. Additional editing if needed (or preferred) can be done after plotting the total (or mean) scattering intensities as described next.

Note that if the alignment is offset to begin with (maybe the detector acquisition and beam intensity measurements didn't get started at precisely the same time), it may be necessary to delete a data object in the plot but not the corresponding point in the profile (or vice versa) to align the beam intensities with the scattering profile (SAXS chromatogram) described next.

3. Make sure I0 and I1 exactly align with the total (or mean) scattering profile and have at least as many points as the scattering profile

This step is very important. If the scattering profile is offset from I0 and I1, subsequent normalization will give incorrect results! If there are fewer points in I0 and I1 than the profile, the relevant data objects at the end of the profile can not be normalized. In this case, the extra data objects should be deleted.

Undo previous normalization

This step is useful if the data were previously normalized with either I0 or I1 alone. It will effectively undo the previous normalization, replacing the original data in the process. The script assumes the data were normalized with the IGOR/BioCAT macros during data reduction. The script may require modification to work with data normalized with other software packages.

1. Click on the plot containing the scattering data in the contents view

2. Select Interpret Script from tool bar Process menu

3. Select SAXS_Undo_Normalization.py from the script menu

4. Click the Interpret button

Normalization

1. Save the document, make a copy, and open the copy.
Exploring normalization options and optimal weights/constant can generate many plots so its probably best to do it in a separate document.

2. Select the plot of the total (or mean) scattering profile in the contents view

3. Select the SAXS_Normalization_exploration.py script and hit Interpret
Inspect the various plots to determine which normalization option and combination of weights or constant give the best overall correction (i.e. the least artifacts correlating with variations in I0 and/or I1).

4. Open the original document and select the plot with the unnormalized scattering data

5. Run the SAXS_Normalization.py script, with the best option, weights, and constant from the normalization exploration
