# Supplementary material for: Structural Organization and Dynamics of Homodimeric Cytohesin Family Arf GTPase Exchange Factors in Solution and on Membranes
Source: Structure. 2019 Dec 3;27(12):1782–1797.e7. doi: 10.1016/j.str.2019.09.007 (PMC6948192; doi:10.1016/j.str.2019.09.007)
Supplement: Document S2. Article plus Supplemental Information [file mmc4.pdf]

Structure

Article

Structural Organization and Dynamics of Homodimeric Cytohesin Family Arf GTPase Exchange Factors in Solution and on Membranes

Graphical Abstract

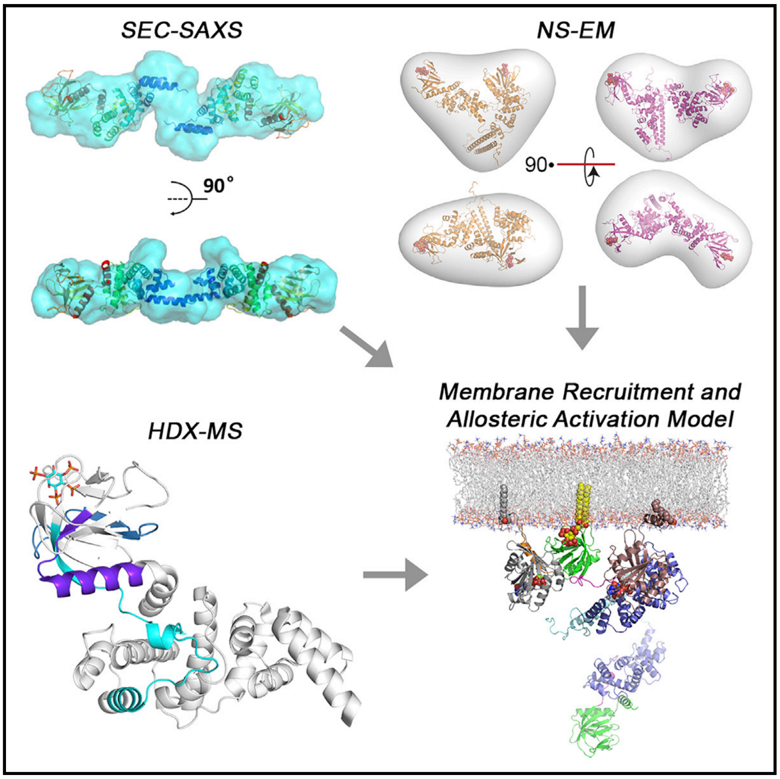

Highlights

- Structural organization of cytohesins investigated by SEC-SAXS, NS-EM, DLS, and HDX-MS
- Cytohesin dimers have an elongated and conformationally dynamic quaternary structure
- Cytohesin dimers bind membranes using one PH domain at a time
- Membrane binding perturbs the structural organization to prime allosteric activation

Authors

Sanchaita Das, Andrew W. Malaby, Agata Nawrotek, ..., Osman Bilsel, Jacqueline Cherfils, David G. Lambright

Correspondence

jacqueline.cherfils@ens-paris-saclay.fr (J.C.),
david.lambright@umassmed.edu (D.G.L.)

In Brief

Arf GEFs activate Arf GTPases to control membrane dynamics. Das et al. investigate the structural organization of cytohesin dimers in solution and on membranes. The dimers adopt elongated, dynamic conformations and use one PH domain at a time to bind membranes, which perturbs the structural organization to prime allosteric activation.

Das et al., 2019, Structure *27*, 1782–1797
December 3, 2019 © 2019 MRC Laboratory of Molecular Biology.
Published by Elsevier Ltd.
https://doi.org/10.1016/j.str.2019.09.007

Cell Press

Structure
Article

Structural Organization and Dynamics of Homodimeric Cytohesin Family Arf GTPase Exchange Factors in Solution and on Membranes

Sanchaita Das,[1,7] Andrew W. Malaby,[1,2,7] Agata Nawrotek,[4,7] Wenhua Zhang,[4,6] Mahel Zeghouf,[4] Sarah Maslen,[5] Mark Skehel,[5] Srinivas Chakravarthy,[3] Thomas C. Irving,[3] Osman Bilsel,[2] Jacqueline Cherfils,[4,*] and David G. Lambright[1,2,8,*]
[1]Program in Molecular Medicine, University of Massachusetts Medical School, Worcester, MA 01605, USA
[2]Department of Biochemistry and Molecular Pharmacology, University of Massachusetts Medical School, Worcester, MA 01655, USA
[3]The Biophysics Collaborative Access Team (BioCAT), Department of Biological Sciences, Illinois Institute of Technology, Chicago, IL 60616, USA
[4]CNRS and Ecole Normale Supérieure Paris-Saclay, 94235 Cachan, France
[5]MRC Laboratory of Molecular Biology, Cambridge CB2 0QH, UK
[6]Present address: School of Life Sciences, Lanzhou University, Lanzhou 730000, China
[7]These authors contributed equally
[8]Lead Contact
*Correspondence: jacqueline.cherfils@ens-paris-saclay.fr (J.C.), david.lambright@umassmed.edu (D.G.L.)
https://doi.org/10.1016/j.str.2019.09.007

SUMMARY

Membrane dynamic processes require Arf GTPase activation by guanine nucleotide exchange factors (GEFs) with a Sec7 domain. Cytohesin family Arf GEFs function in signaling and cell migration through Arf GTPase activation on the plasma membrane and endosomes. In this study, the structural organization of two cytohesins (Grp1 and ARNO) was investigated in solution by size exclusion-small angle X-ray scattering and negative stain-electron microscopy and on membranes by dynamic light scattering, hydrogen-deuterium exchange-mass spectrometry and guanosine diphosphate (GDP)/guanosine triphosphate (GTP) exchange assays. The results suggest that cytohesins form elongated dimers with a central coiled coil and membrane-binding pleckstrin-homology (PH) domains at opposite ends. The dimers display significant conformational heterogeneity, with a preference for compact to intermediate conformations. Phosphoinositide-dependent membrane recruitment is mediated by one PH domain at a time and alters the conformational dynamics to prime allosteric activation by Arf-GTP. A structural model for membrane targeting and allosteric activation of full-length cytohesin dimers is discussed.

INTRODUCTION

Arf GTPases play fundamental roles in vesicle biogenesis and membrane dynamics (Donaldson and Honda, 2005; Donaldson and Jackson, 2011; Hashimoto et al., 2004; Muralidharan-Chari et al., 2009; Nie et al., 2003; Sztul et al., 2019). Activation is controlled by guanine nucleotide exchange factors (GEFs) containing a Sec7 domain, which catalyzes conversion from the inactive guanosine diphosphate (GDP)-bound state to the active guanosine triphosphate (GTP)-bound conformation (Casanova, 2007; Chardin et al., 1996; Cherfils et al., 1998). Additional domains mediate membrane recruitment through interactions with phosphoinositides, anionic phospholipids, and proteins including active Arf or Arl GTPases (Cherfils and Zeghouf, 2013; DiNitto and Lambright, 2006; Lemmon, 2004; Nawrotek et al., 2016). Membrane recruitment of Arf GTPases is mediated by a myristoylated N-terminal amphipathic helix and is required for activation by GEFs (Franco et al., 1995; Goldberg, 1998; Liu et al., 2009, 2010; Pasqualato et al., 2001, 2002; Randazzo et al., 1995).

Cytohesins comprise a metazoan Arf GEF family with four mammalian paralogs (Grp1, ARNO, and cytohesins-1/4) that function in receptor signaling, endocytic trafficking, and cell adhesion/migration (Chardin et al., 1996; Fuss et al., 2006; Hafner et al., 2006; Hickman et al., 2018; Ito et al., 2018; Kolanus et al., 1996; Li et al., 2012; Mohanan et al., 2018; Ogasawara et al., 2000; Rafiq et al., 2017). All cytohesins share a common architecture comprised of a heptad repeat coiled coil (CC) domain, the Sec7 domain, and a pleckstrin-homology (PH) domain. The PH domain binds phosphatidyl inositol 3,4,5-trisphosphate (PIP_3) and/or phosphatidyl inositol 4,5-bisphosphate (PIP_2) (Chardin et al., 1996; Kavran et al., 1998; Klarlund et al., 1997), with affinities, specificities and spatiotemporal distributions dependent on splice variation in the phosphoinositide-binding pocket (Cronin et al., 2004; Klarlund et al., 2000; Ratcliffe et al., 2018). Two autoinhibitory elements, the Sec7-PH linker and C-terminal helix/polybasic region (CtH/PBR), strongly suppress GEF activity by occluding the active site in the Sec7 domain (DiNitto et al., 2007). Mutations in either autoinhibitory element increase GEF activity and truncation of the PBR suffices to render cytohesins constitutively active *in vitro* (DiNitto et al., 2007), albeit with reduced membrane targeting capacity (Nagel et al., 1998). Binding of membrane-associated Arf6-GTP to an allosteric site centered on the PH domain enhances membrane recruitment and relieves autoinhibition by

This is an open access article under the CC BY license (http://creativecommons.org/licenses/by/4.0/).

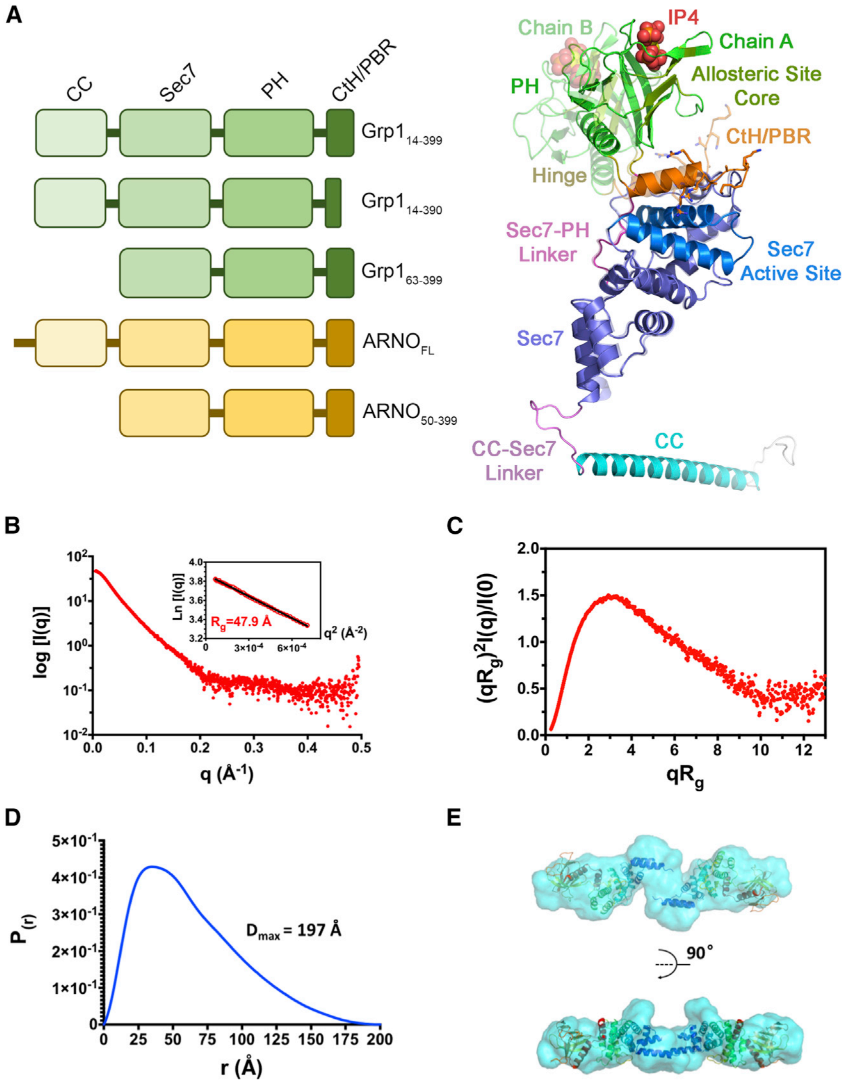

Figure 1. Cytohesin Architecture, Constructs and SAXS Analyses of Full-Length ARNO

(A) Cytohesin constructs used in this study and hypothetical model highlighting relevant structural features. The model is based on chain A from the crystal structure of autoinhibited Grp1$_{63-399}$ with missing regions modeled as described in the STAR Methods. Chain B is rendered as a transparent overlay after alignment of the Sec7 domains. PBR residues are depicted as sticks and the lipid head group as spheres.

(B) SAXS profile of ARNO. The insert shows the Guinier plot ($R_g \times q_{max} = 1.22$).

(C) Dimensionless Kratky plot. The maximum is slightly shifted with respect to a fully globular protein but less than for Grp1.

(D) P(r) plot giving an estimated D_{max} of 197 Å.

(E) Fit of autoinhibited Grp1 structure in a representative envelope calculated by GASBOR and DAMMIN with 2-fold symmetry imposed. Additional envelopes are shown in Figure S1F.

sequestering the CtH/PBR in a groove at the Arf6-GTP/PH interface (Cohen et al., 2007; DiNitto et al., 2007; Malaby et al., 2013; Stalder et al., 2011). The CC domain is implicated in homodimerization (Chardin et al., 1996; Klarlund et al., 2001), heterodimerization with other proteins (DiNitto et al., 2010; Mansour et al., 2002), and intramolecular interactions with the Sec7-PH core (Hiester and Santy, 2013).

Atomic resolution studies have delineated structural bases for phosphoinositide recognition by the PH domain (Cronin et al., 2004; Ferguson et al., 2000; Lietzke et al., 2000), Arf substrate activation by the Sec7 domain (Renault et al., 2003), autoinhibition of the Sec7 active site (DiNitto et al., 2007), and interaction of Arf6-GTP with a linker-PH-CtH/PBR allosteric site fragment (Malaby et al., 2013). The structural organization and conformational dynamics of the monomeric autoregulatory core of Grp1, alone or artificially tethered to Arf6, was further investigated by size exclusion chromatography-small angle X-ray scattering (SEC-SAXS) in combination with single-particle negative stain-electron microscopy (NS-EM) (Malaby et al., 2018). These studies provided evidence for multiple conformations arising from flexibility of hinge residues at the N/C termini of the PH domain in the autoinhibited state as well as flexibility of the Sec7-PH linker in the allosterically activated complex. Hinge flexibility, which can be approximated by a mixture of the two conformers observed in the crystal structure of autoinhibited Grp1 (DiNitto et al., 2007), allows the Sec7 and PH domains to adopt alternative dispositions with distinct accessibility of the allosteric site. Sec7-PH linker flexibility is necessary to expose the active site and may further enhance membrane proximity of the Sec7 through partially ordered conformations in which the last five linker residues are docked in a groove at the Arf-GTP/PH interface as observed in the allosteric site complex (Malaby et al., 2013). However, our understanding of the structure and dynamics of full-length cytohesins and how dimerization mediated by the CC domain affects membrane interactions and Arf activation remains fragmentary.

To gain insight into the structural organization and conformational dynamics of the homodimers, cytohesins with and without the CC domain or PBR (Figure 1A) were investigated in solution using SEC-SAXS and NS-EM and in the presence of membranes using hydrogen-deuterium exchange-mass spectrometry (HDX-MS) and biochemical analyses. The results suggest an elongated, although dynamic, structural organization, with the CC domain at the center and PH domains at opposite ends. Membrane binding involves one PH domain at a time and alters the conformational dynamics within the Sec7-PH core. Our observations support the first complete model for the structural and dynamic organization of full-length cytohesins in solution and on membranes. Functional implications for autoregulation and membrane recruitment are discussed.

RESULTS

Solution Structures of Full-Length ARNO and Grp1 Depict Elongated Dimers

To gain insight into the structural organization of full-length cytohesins, we analyzed the solution structure of ARNO and Grp1, which share 80% identity, using SEC-SAXS. First, we analyzed the structure of full-length ARNO ($ARNO_{FL}$), in the absence of phosphoinositide head groups (statistics in Table S1). The SAXS profile, Kratky plot, and P(r) distribution are shown in Figures 1B–1D. $ARNO_{FL}$ is a dimer in solution, with an estimated radius of gyration (R_G) of 47.9 Å and a D_{max} of 197 Å. The shifted maximum of the Kratky plot (Figure 1C), the shoulder of the P(r) (Figure 1D), and *ab initio* envelopes calculated with DAMMIN and GASBOR with 2-fold symmetry imposed indicate an elongated shape (Figures 1E and S1A). By comparison, $ARNO_{50\text{-}400}$, a construct that lacks the CC domain, has an R_G of 27.5 Å and a D_{max} of 98 Å, which is consistent with a monomeric structure and confirms that the CC domain drives dimerization (SAXS profile with Guinier plot, Kratky plot, and distance distribution function in Figures S1B–S1D, statistics in Table S1). *Ab initio* envelopes for this monomeric construct give a good fit with the crystal structure of autoinhibited Grp1 (DiNitto et al., 2007) (Figures S1E and S1F), indicating that the Sec7 and PH domains of ARNO likely adopt an autoinhibited conformation in solution similar to Grp1. Fitting the autoinhibited Sec7-PH tandem of Grp1 (DiNitto et al., 2007) into the SAXS envelopes of $ARNO_{FL}$ leaves an unoccupied volume in the middle, which is predicted to correspond to the CC domain dimer (Figure 1E). These observations suggest that the CC domain is located at the center of the dimer in close proximity to the Sec7 domain, and that the PH domain is located at the extremities of the elongated structure where it makes no contact with the CC domain.

Next, we analyzed the solution structure of the diglycine variant of autoinhibited $Grp1_{14\text{-}399}$, a construct that includes the CC domain, in complex with the head group of PIP_3 (Figure S2A). Sedimentation equilibrium experiments indicate that this construct is dimeric in the low micromolar concentration range (DiNitto et al., 2007, 2010). In the SEC-SAXS experiment, the peak concentration is ~80 μM and the buffer-subtracted SAXS profiles over the main peak are characterized by a uniform R_G. Minor peaks before and after the main peak may represent a higher-order oligomer and monomer, respectively. Singular value decomposition (SVD) of the SAXS profiles from the main peak and a post-peak buffer region revealed two significant components from which a high-quality protein scattering profile was reconstructed by Guinier-optimized linear combination (SVD-LC) as described previously (Malaby et al., 2015). Guinier analysis of the low q region yielded an R_G of 54.5 Å (Figure 2A; Table S2), which is approximately twice the value of 28 Å for monomeric $Grp1_{63\text{-}399}$, which lacks the CC domain (Malaby et al., 2018). Nearly indistinguishable P(r) distributions calculated by two different algorithms (Figure 2B) provide slightly larger, and likely more accurate, estimates of R_G (57 Å), with tails extending to D_{max} ~260 Å. Molecular weight (MW) estimates are near the calculated value for a dimer (Table S2), with the exception of methods prone to overestimation for non-globular geometries. The shifted maximum in a dimensionless Kratky plot (Figure S4A) indicates that dimeric $Grp1_{14\text{-}399}$ has a more elongated structure than monomeric $Grp1_{63\text{-}399}$. Thus, the scattering profile of $Grp1_{14\text{-}399}$ is consistent with an elongated dimer.

The larger R_G and D_{max} for liganded $Grp1_{14\text{-}399}$ compared with unliganded $ARNO_{FL}$ suggests that head group binding to the PH domain influences the overall tertiary/quaternary structural organization and may be related to changes in H-D exchange rates accompanying membrane-association of $ARNO_{FL}$ described below. It is unlikely that the differences are due to structural variation among cytohesins because R_G and D_{max} are similar for the liganded forms of $ARNO_{2\text{-}400}$ and $Grp1_{14\text{-}399}$ (compare Figures 2A and S3B). Scattering from the head group as well as differences related to splice variants, experimental conditions, and/or details of data processing/analysis may also contribute. Apart from these differences, our analysis of ARNO and Grp1 supports a conserved quaternary architecture for cytohesin family homodimers. We therefore used either Grp1 or ARNO for subsequent structural and biochemical analyses depending on other considerations. Given the availability of structural information for the autoinhibited core, Grp1 was a logical choice for more detailed structural modeling of SEC-SAXS and NS-EM data, whereas ARNO is thought to form the most stable homodimers and was used for GEF assays, and dynamic light scattering (DLS) and HDX-MS experiments.

Modeling of Autoinhibited Grp1 Conformational Dynamics Using SEC-SAXS

To obtain further insight into the conformational dynamics of autoinhibited full-length cytohesins, we carried out *ab initio* and structure-based modeling of the SAXS profile for phosphoinositide-bound $Grp1_{14\text{-}399}$. Averaged/filtered *ab initio* bead envelopes calculated without imposed symmetry using two different algorithms (DAMMIF and GASBOR) have similar elongated shapes with pseudo 2-fold symmetry consistent with the expected dyad symmetry of the CC domain (Figure 2C). More detailed information was provided by rigid body and ensemble analyses based on the crystal structure of the autoinhibited Sec7-PH core ($Grp1_{63\text{-}399}$) connected by flexible linkers to a canonical CC model. Since the SAXS profile for $Grp1_{63\text{-}399}$ is more accurately represented by a combination of two hinge conformations (corresponding to chains A and B from the crystal structure; see also Figure 1A) than either alone (Malaby et al., 2018) and since the topology of the CC is not known, all combinations of hinge conformers (hereafter denoted AA, BB, and AB for the dimer) were analyzed for both parallel and antiparallel topologies. Consensus secondary structure and CC prediction algorithms (Deleage et al., 1997; Frishman and Argos, 1996; Guermeur et al., 1999; Lupas et al., 1991; Rost and Sander, 1993) suggest that the CC spans residues 18–53 but may extend to residues 14–57 and/or fray at the termini.

Rigid body models for dimers consisting of two autoinhibited Sec7-PH fragments connected to CCs spanning residues 18–53 were determined using CORAL, which optimizes the position and orientation of the structured regions subject to spatial constraints for the missing residues in a library of potential backbone configurations (Petoukhov et al., 2012). The best-fitting rigid body models for both parallel and antiparallel CC topologies have nearly identical R_G values in the experimental range (55 versus 53–57 Å) and elongated, dyad symmetric shapes resembling the bead envelopes (Figures 2C and S4C).

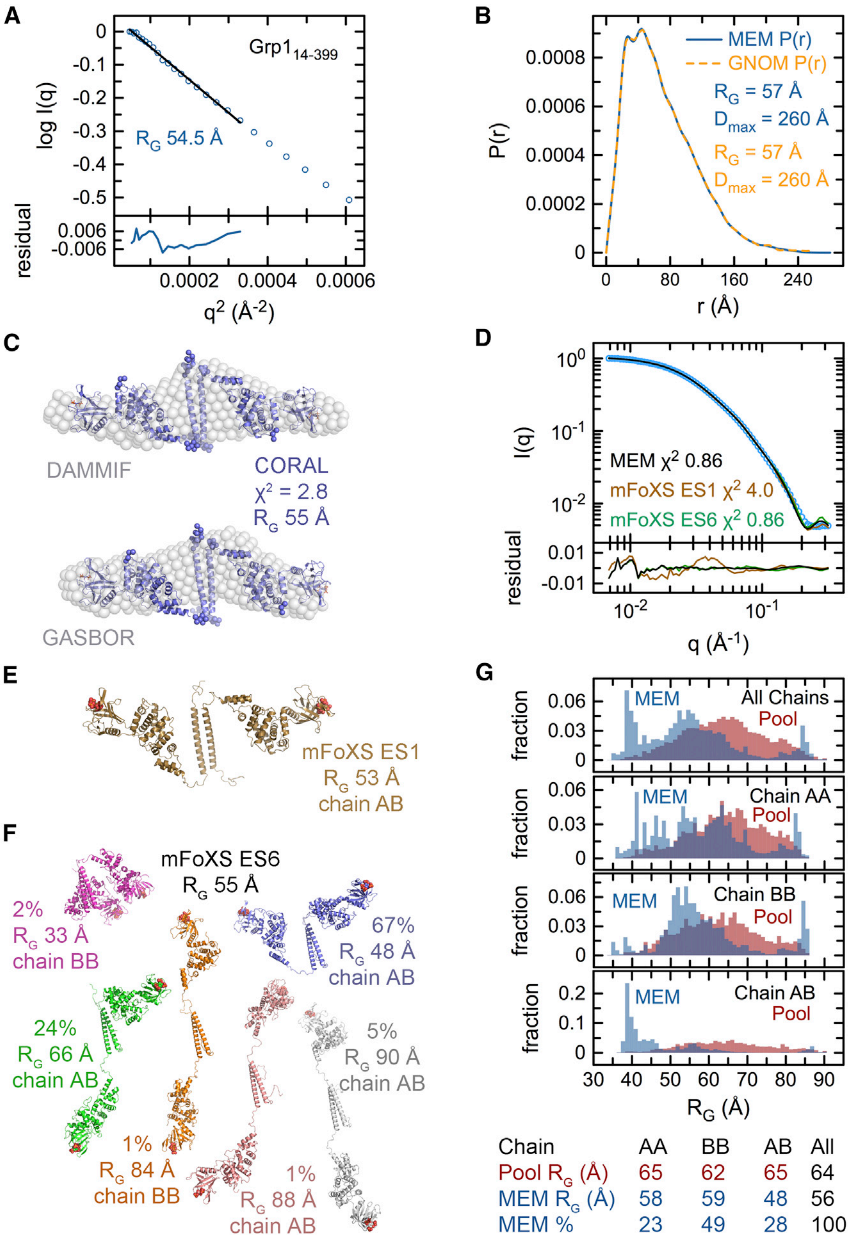

Chain	AA	BB	AB	All
Pool R_G (Å)	65	62	65	64
MEM R_G (Å)	58	59	48	56
MEM %	23	49	28	100

Figure 2. SAXS Analyses of Autoinhibited Grp1 Dimers

(A) Guinier plot and fit for Grp1$_{14\text{-}399}$.

(B) P(r) distributions calculated with GNOM and MEM.

(C) *Ab initio* envelopes calculated with DAMMIF or GASBOR and aligned with the rigid body CORAL model for the antiparallel CC dimer.

(D) Comparison of the experimental SAXS profile with calculated profiles for the best-fitting single model (ES1) and multi-model (ES6) MultiFoXS ensembles as well as the all model MEM distribution for the antiparallel CC dimer.

(E) Best-fitting single-state MultiFoXS model (ES1) for the antiparallel CC dimer.

(F) Models for the best-fitting MultiFoXS ensemble (ES6) for the antiparallel CC dimer with percentages and R_G values. The overall R_G for the ensemble was calculated as the fraction-weighted mean of the individual R_G values.

(G) Fraction-weighted histograms of R_G values for the MEM distribution and pool for the antiparallel CC dimer. Fraction-weighted mean R_G values and percentages are tabulated below.

Nevertheless, the χ^2 values are higher than expected for well-fitting models (χ^2 2.8 versus 1.0 assuming properly estimated errors) and substantial systematic deviations are evident in the residuals. Varying the length of the CC model from residues 18–41 to 14–57 in steps of four residues or restricting the length to a single residue in the middle did not qualitatively alter the overall spatial arrangement of the autoinhibited core or improve the fits, which were characterized by χ^2 values of 3–4. Similar results were obtained for all hinge conformer combinations and CC topologies, suggesting that the SAXS profile cannot be accurately represented by a single conformation.

Best-fitting minimal ensembles selected from large pools of models with flexible connecting loops and terminal regions treated as random coils provides an alternative to rigid body modeling. The MultiFoXS algorithm was used to select best-fitting ensembles with fewer than 10 models from combined pools of 30,000 structural models generated for the 18–53 CC connected to the autoinhibited core, with equal proportions of dimers containing chain A (AA), chain B (BB), or both (AB). The best-fitting single models (ensemble state 1; ES1) have R_G values in the experimental range and resemble the corresponding rigid body models (compare Figures 2E–2C and S4C–S4E), including similar conformations, elevated χ^2 values (χ^2 = 4.0–4.1) and large systematic deviations in the residuals (Figures 2D and S4D). In contrast, the SAXS profile is well described by the best-fitting multistate ensembles (ES6) for both CC topologies (χ^2 = 0.81–0.86; Figures 2D and S4D). These minimal ensembles have overall R_G values in the experimental range and are comprised of six models spanning a broad conformational space, with compact and intermediate models contributing more than extended models (Figures 2F and S4F). Here, "compact," "intermediate," and "extended" denote models with R_G values well below, near, or well above the mean for the pool. Thus, despite having elongated overall shapes, the rigid body and ES1 models are categorized as intermediate with respect to the conformational space represented by the pool.

Although the experimental profile can be fit reasonably well by minimal ensembles with as few as three to four models, the broad range of conformations represented by these ensembles suggests that Grp1 oligomers might adopt a more continuous distribution with many conformations contributing to the SAXS profile. To explore this possibility, the maximum entropy method (MEM) was used to simultaneously fit the profiles for the models

in the MultiFoXS pools to the SAXS profile subject to an informational entropy restraint toward an unbiased prior distribution with equal probability for all models. To facilitate comparison and avoid over fitting, the MEM fits were terminated at χ^2 values corresponding to the best-fitting six-state ensembles. The MEM R_G distributions (Figures 2G and S4G) span a broad continuous range, with contributions from many different models and a preference for compact to intermediate conformations as observed for the MultiFoXS ensembles.

We conclude that the autoinhibited $Grp1_{14\text{-}399}$ dimer exhibits substantial structural dynamics, involving hinge conformers as observed previously for monomeric $Grp1_{63\text{-}399}$ (Malaby et al., 2018) and larger variation in the relative orientation of the CC domain and autoregulatory core due to flexibility in the CC-Sec7 linker.

Analysis of Grp1 Conformational Heterogeneity by Single-Particle NS-EM

Since SAXS profiles are conformationally as well as orientationally averaged, the analyses described above do not directly assess conformational heterogeneity or distinguish between small ensembles and distributions with many conformations. To address these issues and gain additional insight, the structural organization and extent of conformational variability of $Grp1_{14\text{-}399}$ dimers was independently investigated by single-particle NS-EM. The peak fraction after size exclusion chromatography was immediately diluted, applied to freshly glow discharged carbon-coated grids, and stained with uranyl formate. Individual particles with a variety of orientations and/or shapes were observed on raw micrographs (Figure 3A). Unsupervised reference-free classification of ~10,000 manually picked particles (Figure 3B) from 500 micrographs yielded 53 good quality classes representing ~6,500 particles (Figure 3C). The class averages are characterized by a broad range of maximum dimensions (~70–290 Å) and overall shapes that could in principle represent different views of an elongated dimer with an irregular conformation and/or a conformationally heterogeneous population of dimers.

Attempts to generate a 3D reconstruction with visually selected particle classes were unsuccessful, suggesting potential conformational heterogeneity. To explore this possibility, the 2D class averages were systematically compared with 3D volume projections over the range of possible views for each model in the MultiFoXS pools (Figures 4 and S5). The best scoring model/projection for each class strongly resembles the class average (Figures 4A and S5A), indicating that the conformational diversity within the pool is sufficient to represent the range of 2D class averages. The variation in scores for the top models is substantially larger between 2D classes than between hinge conformers (Figures 4B and S5B). The best scoring models span a wide range of size and shape, with mean R_G values (weighted by the particle number in each class) near the range of the SEC-SAXS experiments and a preference for compact to intermediate conformations (Figures 4C, 4D, S5C, and S5D). The best scoring models for 40 of the 53 classes had antiparallel CC topology; however, the differences in score for antiparallel versus parallel topology were minor compared with the variation between classes, with the exception of a few classes where the score for the antiparallel topology was substantially better. A model-free analysis in which 2D classes with similar morphology were combined and reclassified into a larger set of new classes indicates additional conformational heterogeneity within the original set of particle classes (Figure 4E; note additional shapes in the expanded set).

The 2D analysis suggested that conformational similarity of best scoring models might improve selection of classes for 3D reconstruction. For two class sets corresponding to compact or intermediate best scoring models, 3D volumes could be built and refined. Automated docking of the MultiFoXS pools with the refined 3D volumes selected best scoring models having relatively compact conformations (Figures 5 and S6) similar to the more compact models in the six-state MultiFoXS ensembles (Figures 2F and S4F). The low resolution of ~53 Å for the refined 3D volumes (Figure S7) likely reflects conformational heterogeneity in addition to negative staining. Some classes excluded from the sets used for 3D reconstruction have elongated class averages or correspond to views aligned with the long axis of best-fitting models with elongated conformations.

These observations provide direct evidence of substantial structural heterogeneity that lies within the conformational space sampled by the MultiFoXS pool. Flexibility in the CC-Sec7 linker is the main source of conformational variability, with a secondary contribution from hinge conformers that does not appear to be strongly influenced by dimerization. Although the analyses are most consistent with an antiparallel CC topology, the resolution is not sufficient to definitively exclude parallel or mixed topologies. These results and the preference for compact to intermediate conformations are consistent with the SEC-SAXS analysis.

Structural Organization of Constitutively Active Grp1 Mutant Dimers in Solution

We next explored whether the active forms of the dimers are likely to have a similar or distinct structural distribution. Cytohesins lacking the polybasic motif are no longer autoinhibited and can be used as proxies for the active forms (DiNitto et al., 2007). SEC-SAXS data for one such construct ($Grp1_{14\text{-}390}$) were collected at a peak concentration of ~1 mg/mL. Although the signal-to-noise is lower than for the autoinhibited construct, the quality of the reconstructed protein scattering remains sufficiently high to support basic SAXS analyses as well as rigid body and ensemble modeling (Figure S2B). The R_G values derived from Guinier analysis (Figure 6A; R_G = 50.5 Å) and nearly identical GNOM and MEM P(r) distributions (Figure 6B; R_G = 54 Å) are slightly lower than those for $Grp1_{14\text{-}399}$, which can be attributed to the smaller size of the construct. The estimated D_{max} values differ slightly for the GNOM (D_{max} = 257 Å) and MEM (D_{max} = 270 Å) distributions but are nevertheless similar to that of $Grp1_{14\text{-}399}$. As observed for the autoinhibited construct, the MW estimates are consistent with a dimer (Table S2), there is a pronounced shift of the maximum in a dimensionless Kratky plot compared with monomeric $Grp1_{63\text{-}390}$ (Figure S8A), and the *ab initio* bead envelopes are elongated (Figure 6C).

For rigid body and ensemble modeling, the CC for residues 18–53 was combined with the most frequent model in the minimal MultiFoXS ensemble for the experimental profile of the corresponding monomeric construct ($Grp1_{63\text{-}390}$). The best-fitting

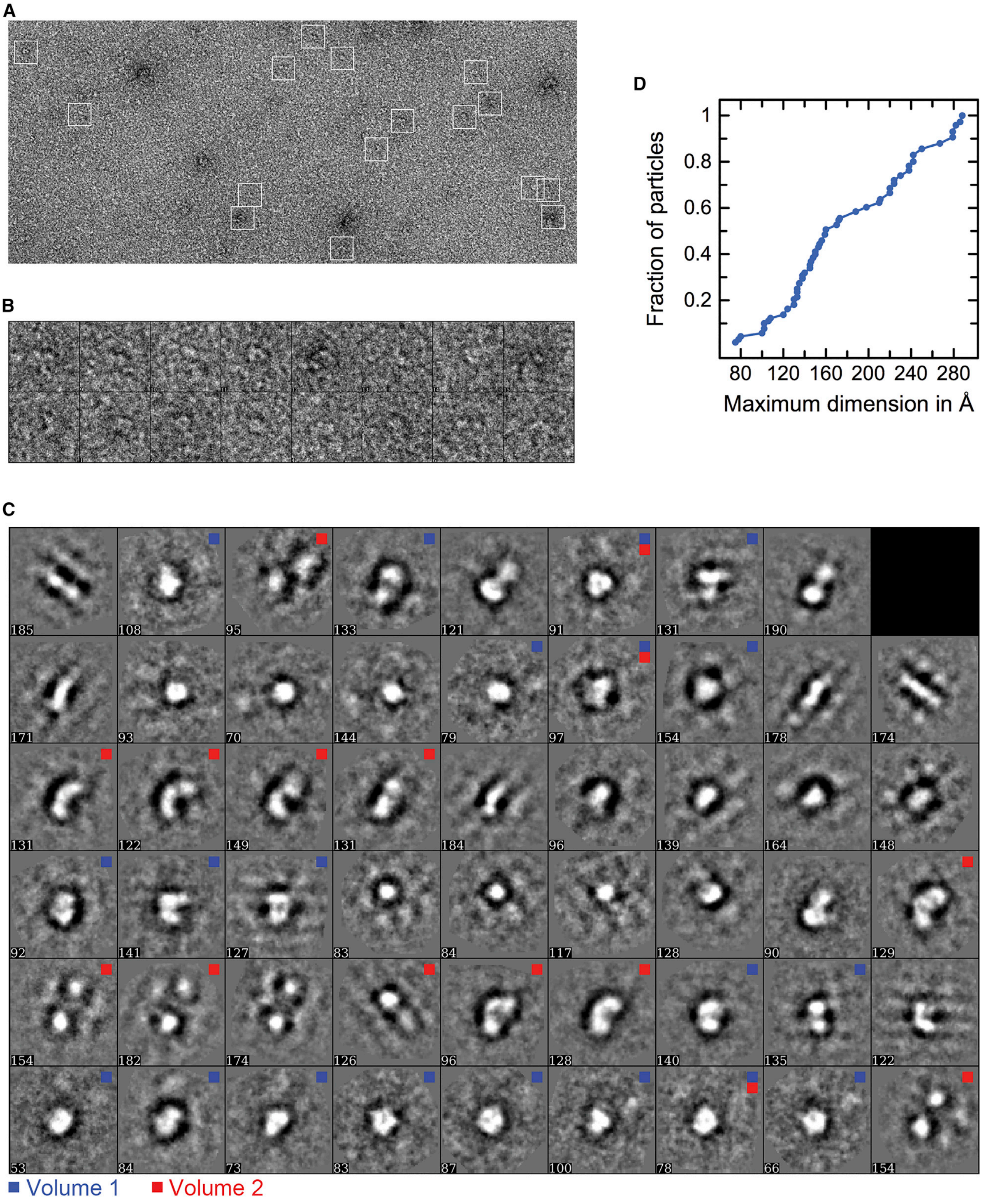

Figure 3. NS-EM Micrograph and Class Averages for Autoinhibited Grp1 Dimers

(A) Representative area of micrograph illustrating Grp1$_{14\text{-}399}$ particles stained with uranyl formate. Boxes indicate representative examples selected particles.

(B) Enlarged views of boxed particles in (A).

(C) Class averages with particle numbers in each class. Blue and red squares denote class sets used for 3D reconstruction.

(D) Cumulative distribution of maximum dimensions for the class averages in (C).

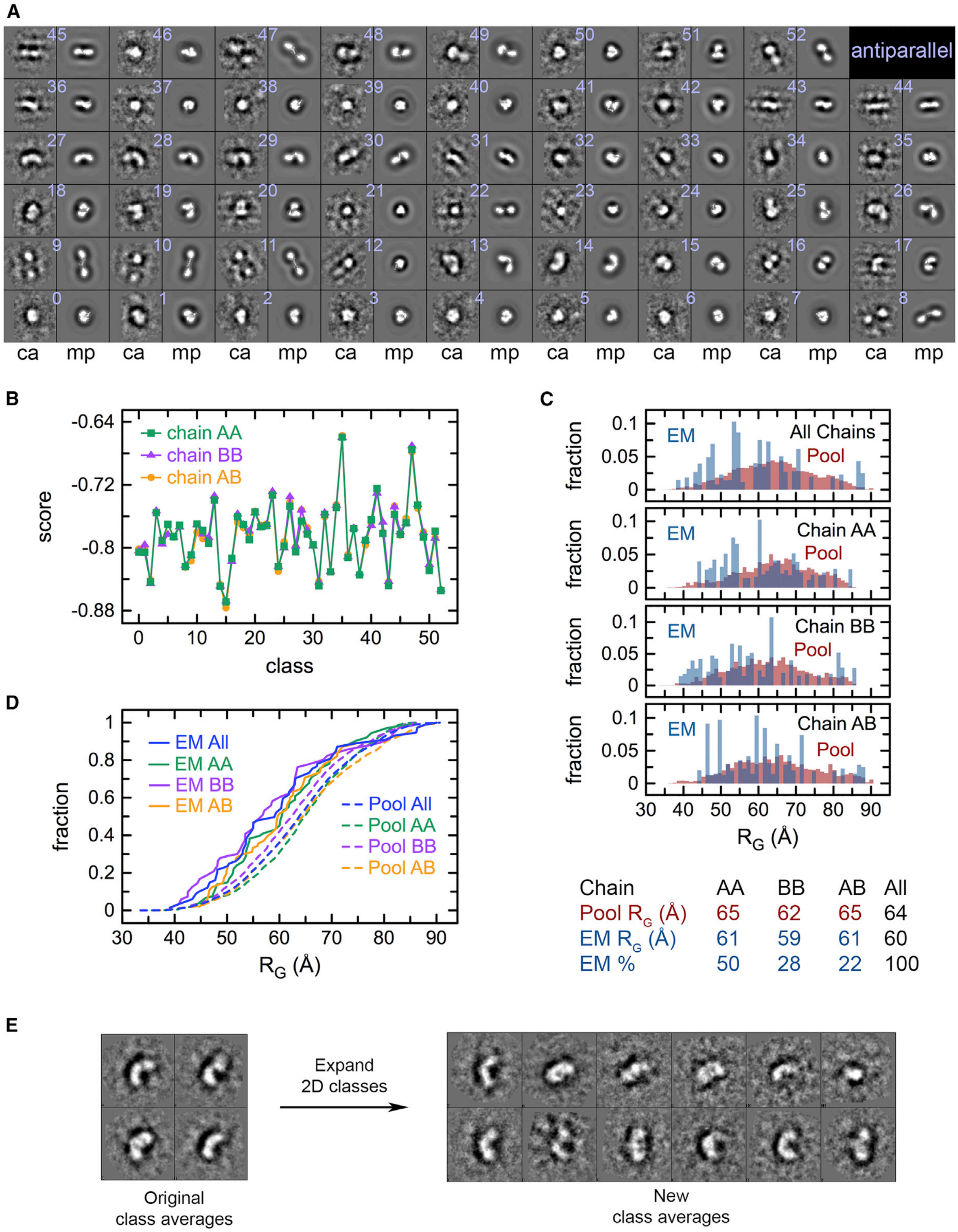

Figure 4. Projection Matching Analysis with Antiparallel MultiFoXS Models

(A) Comparison of class averages with 3D volume projections for the best scoring MultiFoXS models.

(B) Scores for comparison of class averages with 3D volume projections in (A).

(C) Histograms of R_G values for the best scoring model in (A).

(D) Cumulative distribution of R_G values for the best scoring models in (A).

(E) Heterogeneity analysis by expansion and reclassification of morphologically similar 2D classes.

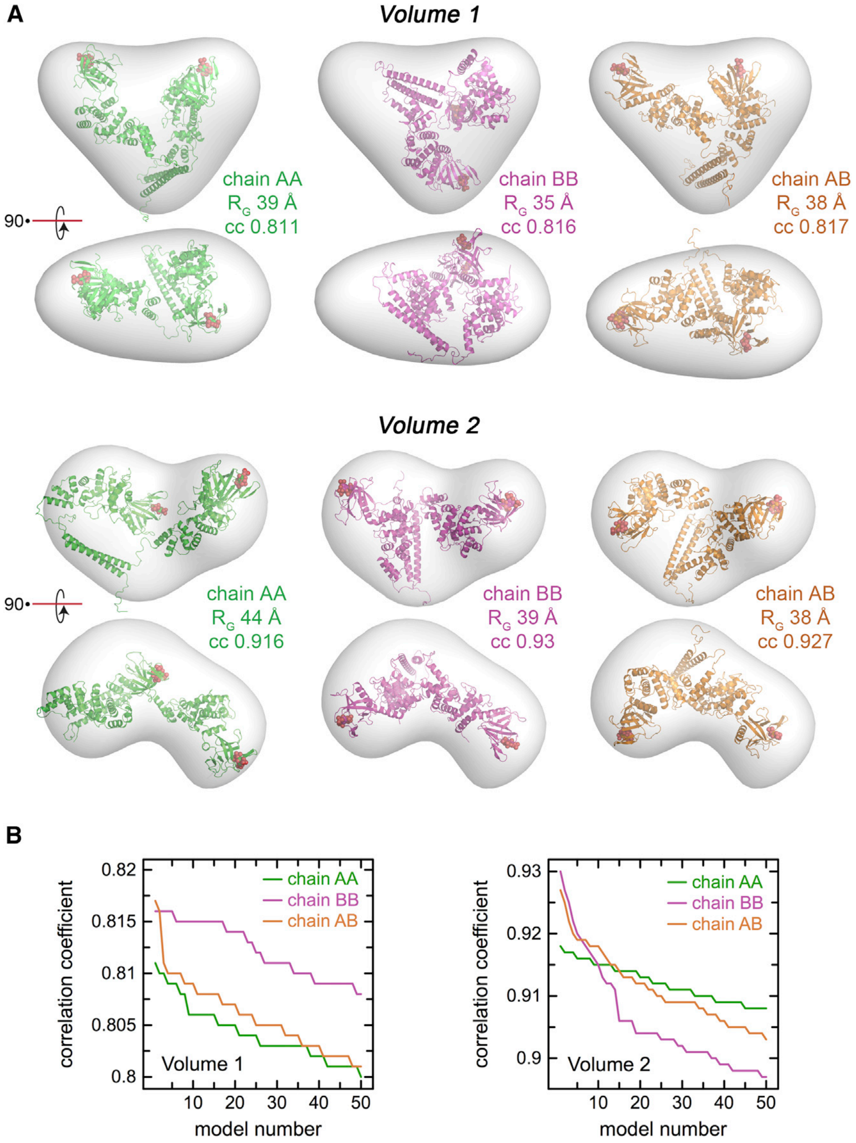

Figure 5. 3D Reconstructions and Best-Fitting Antiparallel MultiFoXS Models

(A) Comparison of the best-fitting MultiFoXS models with the volumes from 3D reconstruction and refinement for the class sets indicated in Figure 4C.

(B) Correlation coefficients for the 50 best-fitting models from the comparison of each volume with the MultiFoXS pools.

rigid body models for both parallel and antiparallel CC topologies have an elongated shape reminiscent of those for Grp1$_{14-399}$ and approximate the *ab initio* bead envelopes (Figures 6C and S8C). Although the best-fitting single-state MultiFoXS models have R_G values near the experimental range and overall shapes similar to the rigid body models (compare Figures 6C–6E and S8C–S8E), the χ^2 values (1.9–2.4) are nevertheless higher than expected for well-fitting models and systematic deviations are evident in the residuals (Figures 6D and S8D). These discrepancies are largely eliminated for the best-fitting MultiFoXS ensembles and MEM distributions. In both cases, the models span a broad conformational space with overall R_G values in the experimental range and a preference for compact to intermediate conformations (Figures 6F, 6G, S8F, and S8G).

The characteristics of the conformational distribution for Grp1$_{14-390}$ generally resemble those of autoinhibited Grp1$_{14-399}$. Flexibility in the Sec7-PH linker is expected to generate additional conformational variability that was not explicitly modeled due to the technical complication of generating representative pools with two flexible linkers. Nevertheless, the results suggest that the active dimers do not have fundamentally different quaternary structural organization or conformational dynamics related to flexibility of the CC-Sec7 linker. Inspection of the models in the MultiFoXS ensemble further suggests that this tertiary/quaternary structural organization does not conflict with accessibility of the Sec7 domain active site to substrate Arf-GDP.

ARNO Dimers Use Only One PH Domain at a Time to Bind to Membranes

The above analysis indicates that the membrane-binding domains of cytohesins are located at the extremities of an elongated structure, where they display significant dynamics. We thus asked how this structural organization affects binding of cytohesins to membranes. First, we analyzed whether the phosphoinositide-binding sites of the two PH domains are aligned such that they can bind simultaneously to the same membrane, or are located in opposition, such that one PH domain could bind to a membrane surface at a time while the other PH domain would point away. We used liposomes that contain the anionic lipids phosphatidylserine and PI(4,5)P_2, to which ARNO binds strongly, to discriminate between these two possibilities by DLS. Dimeric ARNO$_{FL}$ induced conspicuous aggregation of liposomes, while monomeric ARNO$_{50-400}$ had no effect on liposome size distribution (Figure 7A). Membrane tethering by ARNO$_{FL}$ is possible only if the two lipid-binding sites do not bind to the same membrane at the same time, hence are located in opposition. As a consequence, the Sec7 domains, which need to be in close apposition to the membrane for efficient activation of myristoylated Arf (Karandur et al., 2017), may not be equivalent in the dimer. To test this possibility, we took advantage of the fact that ARNO displays significant GEF activity in the presence of membrane (Peurois et al., 2017) to compare the catalytic efficiencies of dimeric ARNO$_{FL}$ and monomeric ARNO$_{50-400}$ at the same concentration of Sec7 active sites, using myristoylated Arf1 and PIP$_2$-containing liposomes (Figure 7B). The concentration range of ARNO used in the kinetics assays was chosen such that no

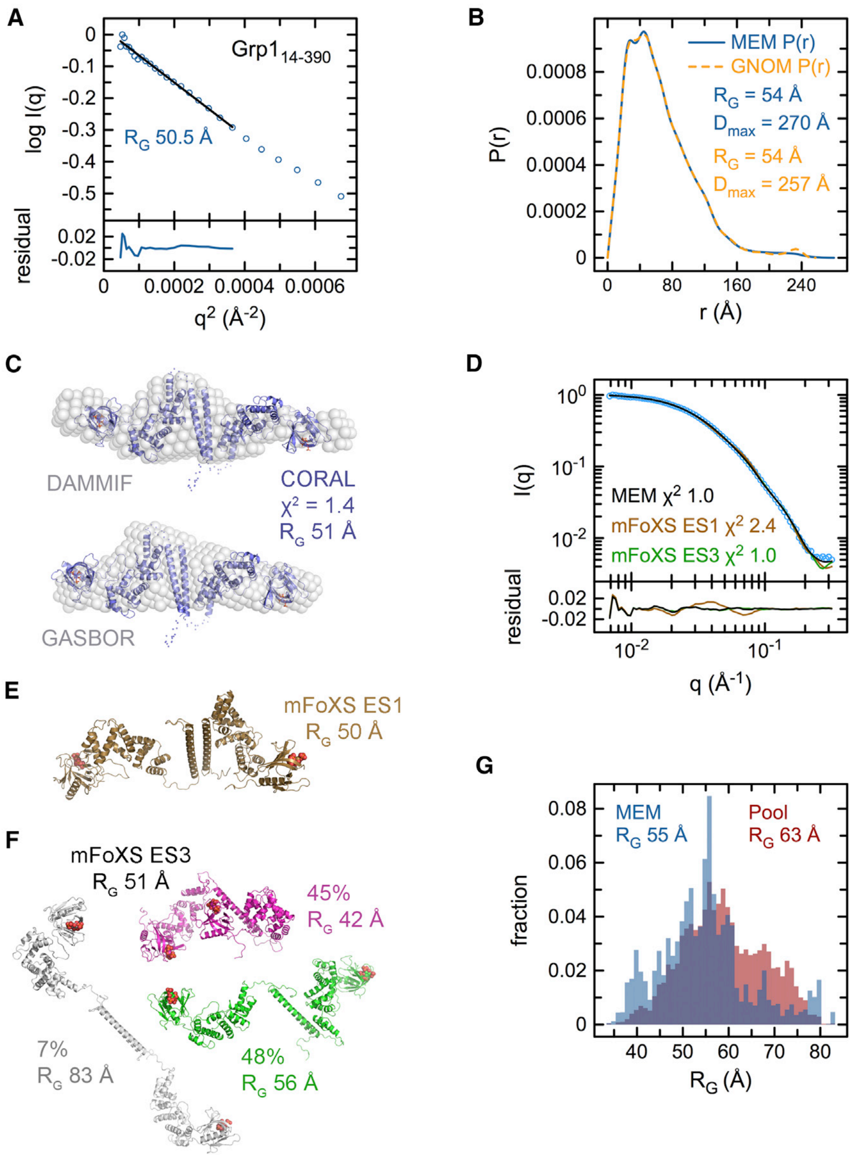

Figure 6. SAXS Analyses of Fully Active Grp1 Dimers

(A) Guinier plot and fit for $Grp1_{14-390}$.

(B) P(r) distributions calculated with GNOM and MEM.

(C) *Ab initio* envelopes calculated with DAMMIF or GASBOR and aligned with the rigid body CORAL model for the antiparallel CC dimer.

(D) Comparison of the experimental SAXS profile with the calculated profiles for the best-fitting single model (ES1) and multiple model (ES3) MultiFoXS ensembles as well as the all model MEM distribution for the antiparallel CC dimer.

(E) Best-fitting single-state MultiFoXS model (ES1) for the antiparallel CC dimer.

(F) Models for the best-fitting MultiFoXS ensemble (ES3) for the antiparallel CC dimer with percentages and R_G values. The overall R_G for the ensemble was calculated as the fraction-weighted mean of the individual R_G values.

(G) Fraction-weighted histograms of R_G values for the MEM distribution and pool for the antiparallel CC dimer. Fraction-weighted mean R_G values and percentages are tabulated below.

liposome aggregation was observed. As shown in Figure 7B, dimeric $ARNO_{FL}$ was 2-fold less active toward myrArf1 in the presence of liposomes than monomeric $ARNO_{50-400}$ (k_{cat}/K_m = 8.02 ± 0.48 10^6 M^{-1} s^{-1} for $ARNO_{FL}$ and 17.61 ± 0.64 10^6 M^{-1} s^{-1} for $ARNO_{50-400}$), which is consistent with a membrane-binding topology in which only one Sec7 active site is available to activate membrane-attached Arf.

HDX-MS Analysis Shows Membranes Remodel the Sec7-PH Domain Interface

Previous studies of autoinhibited ARNO and Grp1 in solution highlighted a positive feedback loop, in which Arf-GTP binds to an allosteric site centered on the PH domain to release autoinhibition (Malaby et al., 2013; Stalder et al., 2011). The kinetics analysis above shows that full-length ARNO is readily active in the presence of liposomes, suggesting that membranes contribute to autoinhibition release independently of Arf-GTP. To analyze how membranes affect the conformation of $ARNO_{FL}$, we used HDX-MS. We obtained good peptide coverage, although most of the CC domain, the Sec7 active site, and several phosphoinositide-binding loops in the PH domain are lacking, for which no information can be deduced (Figure S9A; Table S3). Deuterium incorporation was analyzed in the absence and presence of PIP_2-containing liposomes and mapped on the related structure of autoinhibited Grp1 (Figures 8A and 8B). A marked protection from HD exchange was observed in loop β3–β4 (residues 293–311) in the canonical lipid-binding site of the PH domain in the presence of liposomes, confirming that ARNO binds to liposomes in the HDX-MS setup (Figures 8A, S9B, and S9C). In a more unexpected manner, deuterium incorporation was also decreased in the Sec7-PH linker and in regions immediately upstream and downstream of this linker (residues 234–269). These regions are located opposite to the GTPase-binding and the lipid-binding sites, hence are unlikely to be facing the membrane. Alternatively, protection from HD exchange by liposomes in these regions probably reflects a rearrangement of the Sec7-PH intramolecular interactions. These observations suggest that the membrane remodels intramolecular interactions in ARNO, and that this primes ARNO for full GEF activity toward membrane-attached Arf GTPase.

DISCUSSION

In this study, we investigated the structure, dynamics, and membrane interactions of full-length cytohesins. The data depict cytohesins as elongated dimers with substantial conformational dynamics, in which the CC dimerization domain is

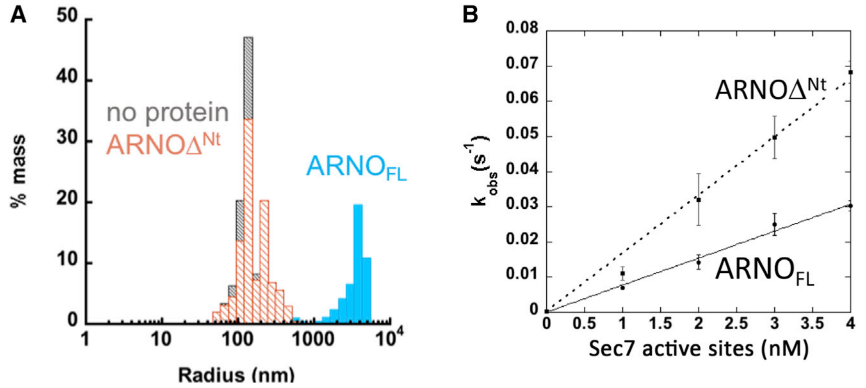

Figure 7. ARNO Uses Only One PH Domain at a Time to Bind to Membranes

(A) DLS experiments show that dimeric $ARNO_{FL}$, but not monomeric $ARNO\Delta^{Nt}$, aggregates PIP_2-containing liposomes.

(B) Catalytic efficiencies of the $ARNO_{FL}$ and $ARNO\Delta^{Nt}$ measured by tryptophan fluorescence in the presence of PIP_2-containing liposomes. The concentration of Sec7 active sites ranges from 0 to 4 nM. k_{obs} are mean ± SD for n = 2 independent experiments.

located at the center and the PH domains at opposite ends. We find that a large fraction of cytohesins use only one PH domain at a time to bind membranes, and that binding to membranes perturbs the structural organization by remodeling the Sec7-PH interface. The results further suggest that the CC domain restricts the conformational distribution through interaction with the Sec7-PH core. This organization is consistent with pull-down experiments using cytohesins overexpressed in cells, which indicate that the CC domain binds to the rest of the protein (Hiester and Santy, 2013). However, our data do not support a direct interaction of the CC with the PH domain, which was inferred from mutation of Thr280 in the β1/β2 loop of the phosphoinositide-binding site in the PH domain (Hiester and Santy, 2013), suggesting that the effect of this mutation may be indirect.

Another important aspect is the considerable dynamics at both the interface between the CC domain and the Sec7-PH core, and within the Sec7-PH core. In solution, cytohesins prefer compact to intermediate conformations, which may be related to intrinsic properties of the inter-domain linkers and how they stabilize intra-dimer interactions. The conformational dynamics of cytohesins is also perturbed in the presence of membranes, as shown by the unexpected change in the Sec7-PH interface upon binding to liposomes. A plausible underlying mechanism would involve repositioning of the CtH/PBR in response to favorable electrostatic interactions of the terminal basic residues with anionic phospholipids. PIP_2 or PIP_3 binding also reduces the electropositive potential surrounding the phosphoinositide site and may lower the barrier for repositioning the CtH/PBR. Finally, intramolecular interactions between the CtH/PBR and PH domain, such as those observed in the PH domain of Grp1 bound to Arf6-GTP (Malaby et al., 2013), may compensate for loss of interactions between these elements and the GEF site.

Although our data suggest that cytohesin homodimers have an elongated, dynamic quaternary structural organization that supports an asymmetric mode of membrane interaction, it is unlikely that cytohesins use this property to tether membranes in cells. Rather, it is plausible that they exploit it to sample the surface of the membrane one PH domain at a time, possibly using their intrinsic dynamics to convert from asymmetrical membrane binding to subsequent binding of both PH domains upon activation by Arf-GTP. Such symmetrical binding of both PH domains may depend on phosphoinositide and Arf-GTP densities, and thereby contribute to coincident detection of lipid and protein inputs. More detailed kinetic analyses as a function of phosphoinositide density over a range of myristoylated Arf-GTP concentration could help clarify whether symmetric binding occurs and under what conditions.

The observations here and in previous studies suggest a structural dynamic model for allosteric activation of cytohesins by membranes and Arf-GTP (Figure 9). In the cytosol, autoinhibited cytohesin dimers adopt an elongated structural organization with considerable intrinsic dynamics. Membrane recruitment is initially mediated by electrostatic interactions with bulk anionic phospholipids, which allows a lateral hopping search for rare PIP_2 or PIP_3, as predicted by molecular dynamics for the monomeric Grp1 PH domain (Lai et al., 2013). Considering that hopping involves transient diffusion into the cytoplasm near the membrane surface (Chen et al., 2012) and that a significant population of cytohesins binds the membrane one PH domain at a time, intramolecular dynamics within the dimers may allow both PH domains of the dimer to participate in alternation to increase efficiency. Once a phosphoinositide is encountered by one of the PH domains, a docked intermediate is formed in which the other subunit of the dimer is disposed toward solution (Figure 9, upper left). This docked intermediate would precede formation of a partially active "primed" intermediate, in which the CtH/PBR is repositioned from the GEF active site through electrostatic interactions with anionic phospholipids (Figure 9, upper right) as well as intramolecular interactions with the PH domain, similar to the conformation in the Arf-GTP-PH complex (Malaby et al., 2013). This would trigger accumulation of a membrane-attached myrArf-GTP pool, allowing subsequent allosteric activation by binding of Arf-GTP to the PH domain, stabilization of the repositioned C-terminal helix and release of the linker from the Sec7 active site to attain a fully active intermediate (Figure 9, lower left). Docking of the last five linker residues (^{261}TFFNP^{265}) in a groove formed at the PH domain/myr-Arf-GTP interface would further enhance the stability of the complex and promote membrane proximal orientations of the Sec7 domain for engagement of membrane-associated myr-Arf-GDP (Figure 9, lower right) as described for the monomeric Grp1-Arf6-GTP fusion (Malaby et al., 2018). The four intermediates depicted in the model explain the structural, dynamical, and biochemical data currently available. It is plausible that allosteric activation of one subunit by Arf-GTP also facilitates engagement of the

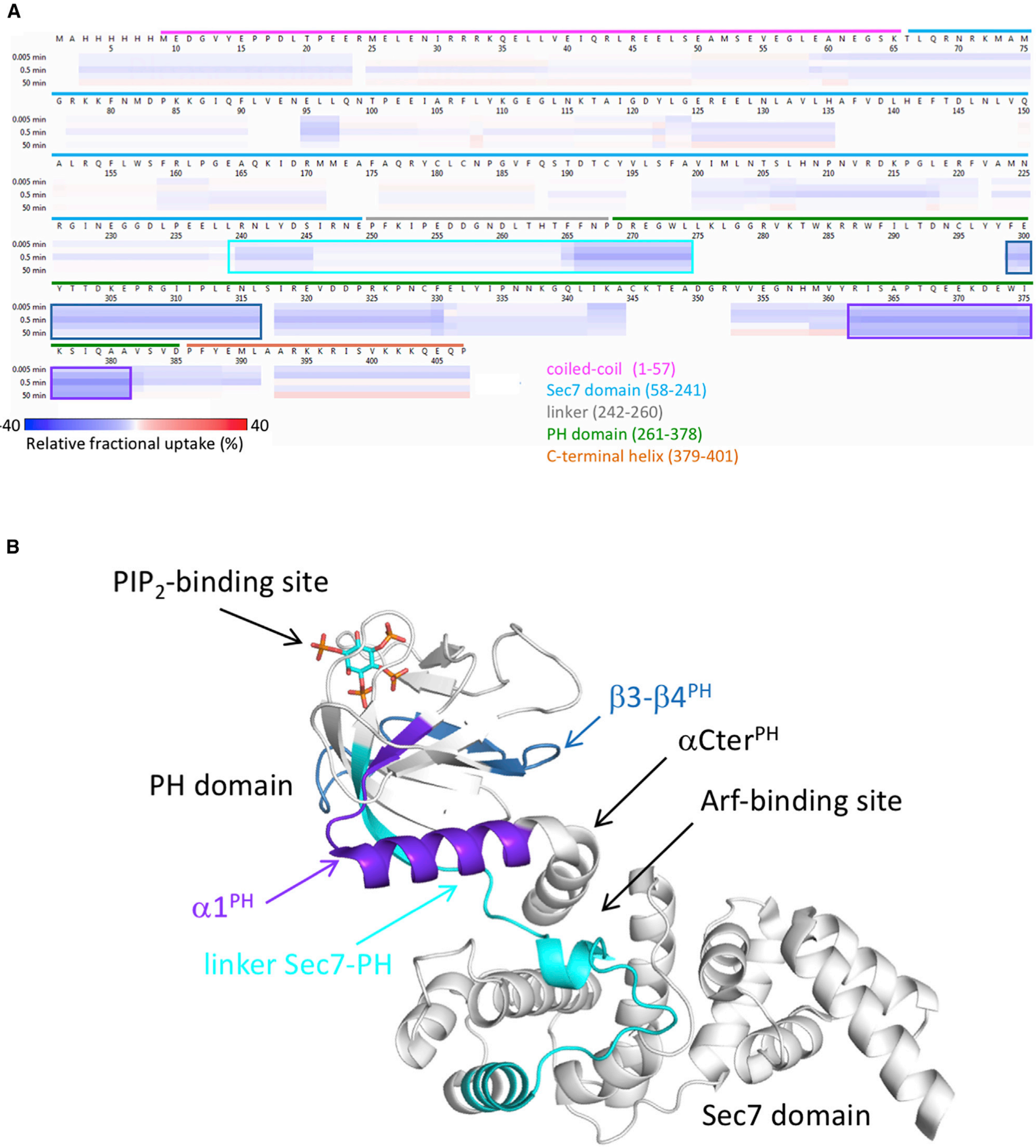

Figure 8. HDX-MS Analysis of the Interaction of $ARNO_{FL}$ with Membranes

(A) Heatmap showing changes in HD exchange. Relative fractional deuteration uptakes induced by the presence of PIP_2-containing liposomes are shown at various time points as color-coded bars ranging from blue (−40%) to red (40%). Regions which can be considered significantly changed, as described in Figure S9, are boxed. Domains are highlighted by colors as indicated. The residue numbers in the His-tagged $ARNO_{FL}$ construct are given below the sequence. The peptic peptide coverage, the butterfly plot of deuterium incorporation and the difference plot are shown in the accompanying Figure S9.

(B) Regions significantly affected by PIP_2-containing liposomes are mapped onto the structure of autoinhibited Grp1 with the color code used for the boxes in Figure 8A.

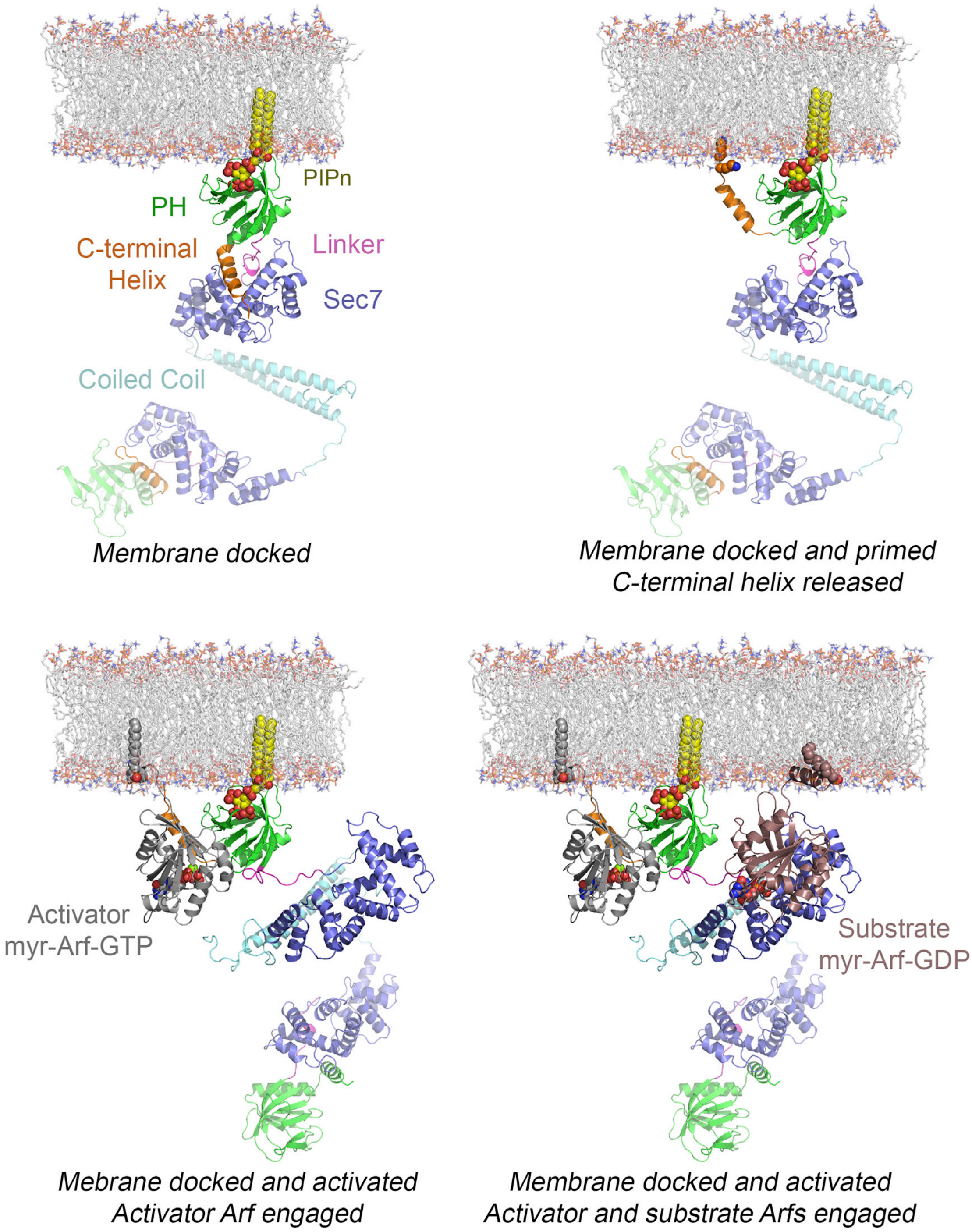

Figure 9. Model for Membrane Recruitment and Allosteric Activation of Cytohesins

Membrane recruitment and allosteric activation of cytohesins is depicted as a series of putative intermediates consistent with observations presented here and elsewhere. The intermediates are based on the most frequent model in the six-state MultiFoXS ensemble for $Grp1_{14\text{-}399}$ (upper left, see Figure 2F) or composites of that model and the most frequent MultiFoXS model for the Grp1-Arf6 fusion where linker residues 252–259 are flexible (Malaby et al., 2018) (other panels). The PH domain was docked with PIP_3 in a model phospholipid bilayer based on the bound head group and residues implicated in membrane partitioning as described previously (Malaby et al., 2013). Activator myr-Arf6-GTP is shown in the orientation observed in the allosteric site complex. Substrate myr-Arf1-GDP was acquired by superposition with the Sec7 domain in the ARNO complex with NΔ17Arf1-GDP (Renault et al., 2003). Myristoylated N-terminal helices were modeled in arbitrary configurations consistent with membrane partitioning. The POPC bilayer membrane was derived from the coordinates of a molecular dynamics simulation (Heller et al., 1993).

second subunit, which together may account for the positive feedback effect mediated by Arf-GTP at the surface of liposomes (Stalder et al., 2011). In the context of this model, conformational dynamics influenced by intra/intermolecular interactions generate multiple conformations for each of the intermediates and contribute to transitions between them.

Together, the analysis of ARNO and Grp1 indicates a structural dynamic pathway of cytohesin dimers in which the central CC and Sec7 domain interface modulates the positions of the lipid-binding sites in the dimer and membranes/phosphoinositides promote conformational changes that prime cytohesins for activation. SAXS modeling and HDX-MS are not expected to capture specific intramolecular interactions or how they are remodeled during the activation process, and further experiments are required to elucidate the structural details. The approach implemented here may be applicable to cytohesin heterodimers as well as other macromolecular complexes with conformationally dynamic states.

STAR★METHODS

Detailed methods are provided in the online version of this paper and include the following:

- KEY RESOURCES TABLE
- LEAD CONTACT AND MATERIALS AVAILABILITY
- EXPERIMENTAL MODEL AND SUBJECT DETAILS
- METHOD DETAILS
  - Constructs, Expression and Purification
  - SEC-SAXS Data Collection and Processing
  - Basic SAXS Analyses and Ab Initio Modeling
  - Rigid Body and Ensemble Modeling
  - Electron Microscopy Sample Preparation and Negative Staining
  - Image Processing, Particle Picking and 2D Classification
  - 2D Heterogeneity Analysis
  - 3D Volume Reconstruction and Refinement
  - Comparison of 2D Class Averages and 3D Volumes with Atomic Resolution Models
  - Liposomes
  - Nucleotide Exchange Kinetics
  - DLS Experiments
  - HDX-MS Experiments
  - Software Resources
- QUANTIFICATION AND STATISTICAL ANALYSIS
  - SAXS Profiles
  - Comparison with Class Averages and Volumes
  - Nucleotide Exchange Kinetics
- DATA AND CODE AVAILABILITY
  - Data and Model Depositions
  - Software
  - Shell Scripts
  - Python Scripts
  - Pipelines

SUPPLEMENTAL INFORMATION

Supplemental Information can be found online at https://doi.org/10.1016/j.str.2019.09.007.

ACKNOWLEDGMENTS

In memory of Pierre Chardin, who pioneered the field of small GTPases. We thank Sagar Kathuria for help with SAXS data collection, Gregory Hendricks, Lara Stritmatter, Chen Xu, and Roger Craig at the UMASS EM core facility for technical support during EM data collection, and Steven Ludtke at Baylor College of Medicine for expert advice on EM data processing and analysis with EMAN2. The research on Grp1 was supported by grant NIH GM056324 (to D.G.L.) and used resources of the Advanced Photon Source, a U.S. Department of Energy (DOE) Office of Science User Facility operated for the DOE Office of Science by Argonne National Laboratory under contract no. DE-AC02-06CH11357. This project was supported by grant 9 P41 GM103622 from the National Institute of General Medical Sciences of the NIH. The content is solely the responsibility of the authors and does not necessarily reflect the official views of the National Institute of General Medical Sciences or the NIH. We thank the teams at the SWING (Synchrotron SOLEIL, Gif-sur-Yvette, France) and BM29 (ESRF, Grenoble, France) beamlines for time allocation and expert advice. The work on ARNO was supported by grants from the Institut National Du Cancer (INCa 2014-160), from the Fondation pour la Recherche Médicale (DEQ20150331694) and from the Agence Nationale de la Recherche (14-CE09-0028) to J.C. and from the European Biophysical Societies' Association to A.N.

AUTHOR CONTRIBUTIONS

Conceptualization, S.D., A.W.M., A.N., O.B., J.C., and D.G.L.; Methodology, S.D., A.W.M., A.N., S.C., T.C.I., O.B., M.S., J.C., and D.G.L.; Investigation, S.D., A.W.M., A.N., W.Z., S.M., and S.C.; Software, S.D., A.W.M., and D.G.L.; Validation, S.D., A.W.M., A.N., M.S., S.D., J.C., and D.G.L.; Formal Analysis, S.D., A.W.M., A.N., S.C., and D.G.L.; Resources, M.Z., S.C., and T.C.I.; Writing – Original Draft, S.D., A.W.M., J.C., and D.G.L.; Writing – Review & Editing, S.D., A.W.M., A.N., S.C., T.C.I., O.B., J.C., and D.G.L.; Visualization, S.D., A.W.M., and D.G.L.; Supervision, T.C.I., O.B., J.C., and D.G.L.; Funding Acquisition, T.C.I., J.C., and D.G.L.

DECLARATION OF INTERESTS

The authors declare no competing interests.

Received: June 25, 2019
Revised: September 3, 2019
Accepted: September 16, 2019
Published: October 7, 2019

REFERENCES

Aizel, K., Biou, V., Navaza, J., Duarte, L.V., Campanacci, V., Cherfils, J., and Zeghouf, M. (2013). Integrated conformational and lipid-sensing regulation of endosomal ArfGEF BRAG2. PLoS Biol. *11*, e1001652.

Antonny, B., Beraud-Dufour, S., Chardin, P., and Chabre, M. (1997). N-terminal hydrophobic residues of the G-protein ADP-ribosylation factor-1 insert into membrane phospholipids upon GDP to GTP exchange. Biochemistry *36*, 4675–4684.

Benabdi, S., Peurois, F., Nawrotek, A., Chikireddy, J., Caneque, T., Yamori, T., Shiina, I., Ohashi, Y., Dan, S., Rodriguez, R., et al. (2017). Family-wide analysis of the inhibition of Arf guanine nucleotide exchange factors with small molecules: evidence of unique inhibitory profiles. Biochemistry *56*, 5125–5133.

Beraud-Dufour, S., Robineau, S., Chardin, P., Paris, S., Chabre, M., Cherfils, J., and Antonny, B. (1998). A glutamic finger in the guanine nucleotide exchange factor ARNO displaces Mg^{2+} and the beta-phosphate to destabilize GDP on ARF1. EMBO J. *17*, 3651–3659.

Berman, H.M., Bhat, T.N., Bourne, P.E., Feng, Z., Gilliland, G., Weissig, H., and Westbrook, J. (2000). The Protein Data Bank and the challenge of structural genomics. Nat. Struct. Biol. *7* (*Suppl*), 957–959.

Booth, D.S., Avila-Sakar, A., and Cheng, Y. (2011). Visualizing proteins and macromolecular complexes by negative stain EM: from grid preparation to image acquisition. J. Vis. Exp. https://doi.org/10.3791/3227.

Carter, L., Kim, S.J., Schneidman-Duhovny, D., Stohr, J., Poncet-Montange, G., Weiss, T.M., Tsuruta, H., Prusiner, S.B., and Sali, A. (2015). Prion protein-antibody complexes characterized by chromatography-coupled small-angle X-ray scattering. Biophys. J. *109*, 793–805.

Casanova, J.E. (2007). Regulation of Arf activation: the Sec7 family of guanine nucleotide exchange factors. Traffic *8*, 1476–1485.

Chardin, P., Paris, S., Antonny, B., Robineau, S., Beraud-Dufour, S., Jackson, C.L., and Chabre, M. (1996). A human exchange factor for ARF contains Sec7- and pleckstrin-homology domains. Nature *384*, 481–484.

Chen, H.C., Ziemba, B.P., Landgraf, K.E., Corbin, J.A., and Falke, J.J. (2012). Membrane docking geometry of GRP1 PH domain bound to a target lipid bilayer: an EPR site-directed spin-labeling and relaxation study. PLoS One *7*, e33640.

Cherfils, J., Menetrey, J., Mathieu, M., Le Bras, G., Robineau, S., Beraud-Dufour, S., Antonny, B., and Chardin, P. (1998). Structure of the Sec7 domain of the Arf exchange factor ARNO. Nature *392*, 101–105.

Cherfils, J., and Zeghouf, M. (2013). Regulation of small GTPases by GEFs, GAPs, and GDIs. Physiol. Rev. *93*, 269–309.

Cohen, L.A., Honda, A., Varnai, P., Brown, F.D., Balla, T., and Donaldson, J.G. (2007). Active Arf6 recruits ARNO/cytohesin GEFs to the PM by binding their PH domains. Mol. Biol. Cell *18*, 2244–2253.

Cronin, T.C., DiNitto, J.P., Czech, M.P., and Lambright, D.G. (2004). Structural determinants of phosphoinositide selectivity in splice variants of Grp1 family PH domains. EMBO J. *23*, 3711–3720.

Deleage, G., Blanchet, C., and Geourjon, C. (1997). Protein structure prediction. Implications for the biologist. Biochimie *79*, 681–686.

DiNitto, J.P., Delprato, A., Gabe Lee, M.T., Cronin, T.C., Huang, S., Guilherme, A., Czech, M.P., and Lambright, D.G. (2007). Structural basis and mechanism of autoregulation in 3-phosphoinositide-dependent Grp1 family Arf GTPase exchange factors. Mol. Cell *28*, 569–583.

DiNitto, J.P., and Lambright, D.G. (2006). Membrane and juxtamembrane targeting by PH and PTB domains. Biochim. Biophys. Acta *1761*, 850–867.

DiNitto, J.P., Lee, M.T., Malaby, A.W., and Lambright, D.G. (2010). Specificity and membrane partitioning of Grsp1 signaling complexes with Grp1 family Arf exchange factors. Biochemistry *49*, 6083–6092.

Donaldson, J.G., and Honda, A. (2005). Localization and function of Arf family GTPases. Biochem. Soc. Trans. *33*, 639–642.

Donaldson, J.G., and Jackson, C.L. (2011). ARF family G proteins and their regulators: roles in membrane transport, development and disease. Nat. Rev. Mol. Cell Biol. *12*, 362–375.

Durand, D., Vives, C., Cannella, D., Perez, J., Pebay-Peyroula, E., Vachette, P., and Fieschi, F. (2010). NADPH oxidase activator p67(phox) behaves in solution as a multidomain protein with semi-flexible linkers. J. Struct. Biol. *169*, 45–53.

Ferguson, K.M., Kavran, J.M., Sankaran, V.G., Fournier, E., Isakoff, S.J., Skolnik, E.Y., and Lemmon, M.A. (2000). Structural basis for discrimination of 3-phosphoinositides by pleckstrin homology domains. Mol. Cell *6*, 373–384.

Franco, M., Chardin, P., Chabre, M., and Paris, S. (1993). Myristoylation is not required for GTP-dependent binding of ADP-ribosylation factor ARF1 to phospholipids. J. Biol. Chem. *268*, 24531–24534.

Franco, M., Chardin, P., Chabre, M., and Paris, S. (1995). Myristoylation of ADP-ribosylation factor 1 facilitates nucleotide exchange at physiological Mg2+ levels. J. Biol. Chem. *270*, 1337–1341.

Franke, D., Petoukhov, M.V., Konarev, P.V., Panjkovich, A., Tuukkanen, A., Mertens, H.D.T., Kikhney, A.G., Hajizadeh, N.R., Franklin, J.M., Jeffries, C.M., et al. (2017). ATSAS 2.8: a comprehensive data analysis suite for small-angle scattering from macromolecular solutions. J. Appl. Crystallogr. *50*, 1212–1225.

Franke, D., and Svergun, D.I. (2009). DAMMIF, a program for rapid ab-initio shape determination in small-angle scattering. J. Appl. Crystallogr. *42*, 342–346.

Frishman, D., and Argos, P. (1996). Incorporation of non-local interactions in protein secondary structure prediction from the amino acid sequence. Protein Eng. *9*, 133–142.

Fuss, B., Becker, T., Zinke, I., and Hoch, M. (2006). The cytohesin Steppke is essential for insulin signalling in *Drosophila*. Nature *444*, 945–948.

Garzón, J.I., Kovacs, J., Abagyan, R., and Chacon, P. (2007). ADP_EM: fast exhaustive multi-resolution docking for high-throughput coverage. Bioinformatics *23*, 427–433.

Goldberg, J. (1998). Structural basis for activation of ARF GTPase: mechanisms of guanine nucleotide exchange and GTP-myristoyl switching. Cell *95*, 237–248.

Guermeur, Y., Geourjon, C., Gallinari, P., and Deleage, G. (1999). Improved performance in protein secondary structure prediction by inhomogeneous score combination. Bioinformatics *15*, 413–421.

Hafner, M., Schmitz, A., Grune, I., Srivatsan, S.G., Paul, B., Kolanus, W., Quast, T., Kremmer, E., Bauer, I., and Famulok, M. (2006). Inhibition of cytohesins by SecinH3 leads to hepatic insulin resistance. Nature *444*, 941–944.

Hashimoto, S., Onodera, Y., Hashimoto, A., Tanaka, M., Hamaguchi, M., Yamada, A., and Sabe, H. (2004). Requirement for Arf6 in breast cancer invasive activities. Proc. Natl. Acad. Sci. U S A *101*, 6647–6652.

Heller, H., Schaefer, M., and Schulten, K. (1993). Molecular dynamics simulation of a bilayer of 200 lipids in the gel and in the liquid crystal phase. J. Phys. Chem. *97*, 8343–8360.

Hickman, F.E., Stanley, E.M., and Carter, B.D. (2018). Neurotrophin responsiveness of sympathetic neurons is regulated by rapid mobilization of the p75 receptor to the cell surface through TrkA activation of Arf6. J. Neurosci. *38*, 5606–5619.

Hiester, K.G., and Santy, L.C. (2013). The cytohesin coiled-coil domain interacts with threonine 276 to control membrane association. PLoS One *8*, e82084.

Ito, A., Fukaya, M., Saegusa, S., Kobayashi, E., Sugawara, T., Hara, Y., Yamauchi, J., Okamoto, H., and Sakagami, H. (2018). Pallidin is a novel interacting protein for cytohesin-2 and regulates the early endosomal pathway and dendritic formation in neurons. J. Neurochem. *147*, 153–177.

Karandur, D., Nawrotek, A., Kuriyan, J., and Cherfils, J. (2017). Multiple interactions between an Arf/GEF complex and charged lipids determine activation kinetics on the membrane. Proc. Natl. Acad. Sci. U S A *114*, 11416–11421.

Kavran, J.M., Klein, D.E., Lee, A., Falasca, M., Isakoff, S.J., Skolnik, E.Y., and Lemmon, M.A. (1998). Specificity and promiscuity in phosphoinositide binding by pleckstrin homology domains. J. Biol. Chem. *273*, 30497–30508.

Klarlund, J.K., Guilherme, A., Holik, J.J., Virbasius, J.V., Chawla, A., and Czech, M.P. (1997). Signaling by phosphoinositide-3,4,5-trisphosphate through proteins containing pleckstrin and Sec7 homology domains. Science *275*, 1927–1930.

Klarlund, J.K., Holik, J., Chawla, A., Park, J.G., Buxton, J., and Czech, M.P. (2001). Signaling complexes of the FERM domain-containing protein GRSP1 bound to ARF exchange factor GRP1. J. Biol. Chem. *276*, 40065–40070.

Klarlund, J.K., Tsiaras, W., Holik, J.J., Chawla, A., and Czech, M.P. (2000). Distinct polyphosphoinositide binding selectivities for pleckstrin homology domains of GRP1-like proteins based on diglycine versus triglycine motifs. J. Biol. Chem. *275*, 32816–32821.

Kolanus, W., Nagel, W., Schiller, B., Zeitlmann, L., Godar, S., Stockinger, H., and Seed, B. (1996). Alpha L beta 2 integrin/LFA-1 binding to ICAM-1 induced by cytohesin-1, a cytoplasmic regulatory molecule. Cell *86*, 233–242.

Konarev, P.V., Volkov, V.V., Sokolova, A.V., Koch, M.H.J., and Svergun, D.I. (2003). PRIMUS: a Windows PC-based system for small-angle scattering data analysis. J. Appl. Crystallogr. *36*, 1277–1282.

Kozin, M.B., and Svergun, D.I. (2001). Automated matching of high- and low-resolution structural models. J. Appl. Crystallogr. *34*, 33–41.

Kremer, J.R., Mastronarde, D.N., and McIntosh, J.R. (1996). Computer visualization of three-dimensional image data using IMOD. J. Struct. Biol. *116*, 71–76.

Lai, C.L., Srivastava, A., Pilling, C., Chase, A.R., Falke, J.J., and Voth, G.A. (2013). Molecular mechanism of membrane binding of the GRP1 PH domain. J. Mol. Biol. *425*, 3073–3090.

Lawson, C.L., Patwardhan, A., Baker, M.L., Hryc, C., Garcia, E.S., Hudson, B.P., Lagerstedt, I., Ludtke, S.J., Pintilie, G., Sala, R., et al. (2016). EMDataBank unified data resource for 3DEM. Nucleic Acids Res. *44*, D396–D403.

Lemmon, M.A. (2004). Pleckstrin homology domains: not just for phosphoinositides. Biochem. Soc. Trans. *32*, 707–711.

Li, J., Malaby, A.W., Famulok, M., Sabe, H., Lambright, D.G., and Hsu, V.W. (2012). Grp1 plays a key role in linking insulin signaling to glut4 recycling. Dev. Cell *22*, 1286–1298.

Lietzke, S.E., Bose, S., Cronin, T., Klarlund, J., Chawla, A., Czech, M.P., and Lambright, D.G. (2000). Structural basis of 3-phosphoinositide recognition by pleckstrin homology domains. Mol. Cell *6*, 385–394.

Liu, Y., Kahn, R.A., and Prestegard, J.H. (2009). Structure and membrane interaction of myristoylated ARF1. Structure *17*, 79–87.

Liu, Y., Kahn, R.A., and Prestegard, J.H. (2010). Dynamic structure of membrane-anchored Arf*GTP. Nat. Struct. Mol. Biol. *17*, 876–881.

Lupas, A., Van Dyke, M., and Stock, J. (1991). Predicting coiled coils from protein sequences. Science *252*, 1162–1164.

Malaby, A.W., Chakravrthy, S., Irving, T.C., Kathuria, S.V., Osman, B., and Lambright, D.G. (2015). Methods for analysis of size-exclusion chromatography-small angle X-ray scattering and reconstruction of protein scattering. J. Appl. Crystallogr. *48* (Pt 4), 1102–1113.

Malaby, A.W., Das, S., Chakravarthy, S., Irving, T.C., Bilsel, O., and Lambright, D.G. (2018). Structural dynamics control allosteric activation of cytohesin family Arf GTPase exchange factors. Structure *26*, 106–117.e6.

Malaby, A.W., van den Berg, B., and Lambright, D.G. (2013). Structural basis for membrane recruitment and allosteric activation of cytohesin family Arf GTPase exchange factors. Proc. Natl. Acad. Sci. U S A *110*, 14213–14218.

Mansour, M., Lee, S.Y., and Pohajdak, B. (2002). The N-terminal coiled coil domain of the cytohesin/ARNO family of guanine nucleotide exchange factors interacts with the scaffolding protein CASP. J. Biol. Chem. *277*, 32302–32309.

Mohanan, V., Nakata, T., Desch, A.N., Levesque, C., Boroughs, A., Guzman, G., Cao, Z., Creasey, E., Yao, J., Boucher, G., et al. (2018). C1orf106 is a colitis risk gene that regulates stability of epithelial adherens junctions. Science *359*, 1161–1166.

Morin, A., and Sliz, P. (2013). Structural biology computing: lessons for the biomedical research sciences. Biopolymers *99*, 809–816.

Muralidharan-Chari, V., Clancy, J., Plou, C., Romao, M., Chavrier, P., Raposo, G., and D'Souza-Schorey, C. (2009). ARF6-regulated shedding of tumor cell-derived plasma membrane microvesicles. Curr. Biol. *19*, 1875–1885.

Nagel, W., Schilcher, P., Zeitlmann, L., and Kolanus, W. (1998). The PH domain and the polybasic C domain of cytohesin-1 cooperate specifically in plasma membrane association and cellular function. Mol. Biol. Cell *9*, 1981–1994.

Nawrotek, A., Zeghouf, M., and Cherfils, J. (2016). Allosteric regulation of Arf GTPases and their GEFs at the membrane interface. Small GTPases *7*, 283–296.

Nie, Z., Hirsch, D.S., and Randazzo, P.A. (2003). Arf and its many interactors. Curr. Opin. Cell Biol. *15*, 396–404.

Ogasawara, M., Kim, S.C., Adamik, R., Togawa, A., Ferrans, V.J., Takeda, K., Kirby, M., Moss, J., and Vaughan, M. (2000). Similarities in function and gene structure of cytohesin-4 and cytohesin-1, guanine nucleotide-exchange proteins for ADP-ribosylation factors. J. Biol. Chem. *275*, 3221–3230.

Pasqualato, S., Menetrey, J., Franco, M., and Cherfils, J. (2001). The structural GDP/GTP cycle of human Arf6. EMBO Rep. *2*, 234–238.

Pasqualato, S., Renault, L., and Cherfils, J. (2002). Arf, Arl, Arp and Sar proteins: a family of GTP-binding proteins with a structural device for 'front-back' communication. EMBO Rep. *3*, 1035–1041.

Petoukhov, M.V., Franke, D., Shkumatov, A.V., Tria, G., Kikhney, A.G., Gajda, M., Gorba, C., Mertens, H.D.T., Konarev, P.V., and Svergun, D.I. (2012). New developments in the ATSAS program package for small-angle scattering data analysis. J. Appl. Crystallogr. *45*, 342–350.

Pettersen, E.F., Goddard, T.D., Huang, C.C., Couch, G.S., Greenblatt, D.M., Meng, E.C., and Ferrin, T.E. (2004). UCSF Chimera—a visualization system for exploratory research and analysis. J. Comput. Chem. *25*, 1605–1612.

Peurois, F., Veyron, S., Ferrandez, Y., Ladid, I., Benabdi, S., Zeghouf, M., Peyroche, G., and Cherfils, J. (2017). Characterization of the activation of small GTPases by their GEFs on membranes using artificial membrane tethering. Biochem. J. *474*, 1259–1272.

Rafiq, N.B., Lieu, Z.Z., Jiang, T., Yu, C.H., Matsudaira, P., Jones, G.E., and Bershadsky, A.D. (2017). Podosome assembly is controlled by the GTPase ARF1 and its nucleotide exchange factor ARNO. J. Cell Biol. *216*, 181–197.

Randazzo, P.A., Terui, T., Sturch, S., Fales, H.M., Ferrige, A.G., and Kahn, R.A. (1995). The myristoylated amino terminus of ADP-ribosylation factor 1 is a phospholipid- and GTP-sensitive switch. J. Biol. Chem. *270*, 14809–14815.

Ratcliffe, C.D.H., Siddiqui, N., Coelho, P.P., Laterreur, N., Cookey, T.N., Sonenberg, N., and Park, M. (2018). HGF-induced migration depends on the PI(3,4,5)P3-binding microexon-spliced variant of the Arf6 exchange factor cytohesin-1. J. Cell Biol. *218*, 285–298.

Raveh, B., Enosh, A., Schueler-Furman, O., and Halperin, D. (2009). Rapid sampling of molecular motions with prior information constraints. PLoS Comput. Biol. *5*, e1000295.

Renault, L., Guibert, B., and Cherfils, J. (2003). Structural snapshots of the mechanism and inhibition of a guanine nucleotide exchange factor. Nature *426*, 525–530.

Rosenthal, P.B., and Henderson, R. (2003). Optimal determination of particle orientation, absolute hand, and contrast loss in single-particle electron cryomicroscopy. J. Mol. Biol. *333*, 721–745.

Rost, B., and Sander, C. (1993). Prediction of protein secondary structure at better than 70% accuracy. J. Mol. Biol. *232*, 584–599.

Russel, D., Lasker, K., Webb, B., Velázquez-Muriel, J., Tjioe, E., Schneidman-Duhovny, D., Peterson, B., and Sali, A. (2012). Putting the pieces together: integrative modeling platform software for structure determination of macromolecular assemblies. PLoS Biol. *10*, e1001244.

Schneidman-Duhovny, D., Hammel, M., Tainer, J.A., and Sali, A. (2013). Accurate SAXS profile computation and its assessment by contrast variation experiments. Biophys. J. *105*, 962–974.

Silva, J.C., Gorenstein, M.V., Li, G.Z., Vissers, J.P., and Geromanos, S.J. (2006). Absolute quantification of proteins by LCMSE: a virtue of parallel MS acquisition. Mol. Cell Proteomics *5*, 144–156.

Skilling, J., and Bryan, S.K. (1984). Maximum entropy image reconstruction: general algorithm. Mon. Not. R. Astr. Soc. *211*, 111–124.

Stalder, D., Barelli, H., Gautier, R., Macia, E., Jackson, C.L., and Antonny, B. (2011). Kinetic studies of the Arf activator Arno on model membranes in the presence of Arf effectors suggest control by a positive feedback loop. J. Biol. Chem. *286*, 3873–3883.

Svergun, D. (1992). Determination of the regularization parameter in indirect-transform methods using perceptual criteria. J. Appl. Crystallogr. *25*, 495–503.

Svergun, D.I., Petoukhov, M.V., and Koch, M.H. (2001). Determination of domain structure of proteins from X-ray solution scattering. Biophys. J. *80*, 2946–2953.

Sztul, E., Chen, P.W., Casanova, J.E., Cherfils, J., Dacks, J.B., Lambright, D.G., Lee, F.S., Randazzo, P.A., Santy, L.C., Schurmann, A., et al. (2019). ARF GTPases and their GEFs and GAPs: concepts and challenges. Mol. Biol. Cell *30*, 1249–1271.

Tang, G., Peng, L., Baldwin, P.R., Mann, D.S., Jiang, W., Rees, I., and Ludtke, S.J. (2007). EMAN2: an extensible image processing suite for electron microscopy. J. Struct. Biol. *157*, 38–46.

Valentini, E., Kikhney, A.G., Previtali, G., Jeffries, C.M., and Svergun, D.I. (2015). SASBDB, a repository for biological small-angle scattering data. Nucleic Acids Res. *43*, D357–D363.

Volkov, V.V., and Svergun, D.I. (2003). Uniqueness of ab initio shape determination in small-angle scattering. J. Appl. Crystallogr. *36*, 860–864.

Webb, B., and Sali, A. (2014). Comparative protein structure modeling using MODELLER. Curr. Protoc. Bioinformatics *47*, 5 6 1–32.

Wood, C.W., Bruning, M., Ibarra, A.A., Bartlett, G.J., Thomson, A.R., Sessions, R.B., Brady, R.L., and Woolfson, D.N. (2014). CCBuilder: an interactive web-based tool for building, designing and assessing coiled-coil protein assemblies. Bioinformatics *30*, 3029–3035.

STAR★METHODS

KEY RESOURCES TABLE

REAGENT or RESOURCE	SOURCE	IDENTIFIER
**Bacterial and Virus Strains**		
BL21(DE3) Competent Cells	Novagen	Cat#69450
XL-10 Gold Ultracompetent Cells	Agilent	Cat#200314
**Chemicals, Peptides, and Recombinant Proteins**		
Inositol 1,3,4,5 tetrakis-phosphate, Potassium Salt (IP4)	Cell Signals	Cat#803
Uranyl Formate	EM Sciences	Cat#22450
phosphatidylcholine (PC)	Avanti	Cat#840053C
phosphatidylethanolamine (PE),	Avanti	Cat#840022C
Phosphatidylserine (PS)	Avanti	Cat#840032C
cholesterol	Sigma	Cat#C8667
NBD-PE	Avanti	Cat#810144C
phosphatidylinositol-4,5-triphosphate (PIP2)	Avanti	Cat#850185P
**Critical Commercial Assays**		
Wizard Plus Miniprep DNA Purification Kit	Promega	Cat#A7510
Wizard SV Gel and PCR Cleanup Kit	Promega	Cat#A9281
**Deposited Data**		
Grp1 63-399 + IP4	DiNitto et al., 2007	PDB: 2R09
Cytohesin-2; ARF nucleotide-binding site opener, ARNO truncation mutant	This paper	SASBDB: SASDEV9 https://www.sasbdb.org
Cytohesin-2; ARF nucleotide-binding site opener, ARNO	This paper	SASBDB: SASDEW9 https://www.sasbdb.org
Grp1 14-399 + IP4 SAXS with DAMMIF and GASBOR models	This paper	SASBDB: SASDG64 https://www.sasbdb.org
Grp1 14-399 + IP4 SAXS with antiparallel CORAL and MultiFoXS models	This paper	SASBDB: SASDG94 https://www.sasbdb.org
Grp1 14-399 + IP4 SAXS with parallel CORAL and MultiFoXS models	This paper	SASBDB: SASDGA4 https://www.sasbdb.org
Grp1 14-390 + IP4 SAXS with DAMMIF and GASBOR models	This paper	SASBDB: SASDG74 https://www.sasbdb.org
Grp1 14-390 + IP4 SAXS with antiparallel CORAL and MultiFoXS models	This paper	SASBDB: SASDGB4 https://www.sasbdb.org
Grp1 14-390 + IP4 SAXS with parallel CORAL and MultiFoXS models	This paper	SASBDB: SASDGC4 https://www.sasbdb.org
ARNO 2-400 + IP4 SAXS with DAMMIF, GASBOR and antiparallel CORAL models	This paper	SASBDB: SASDG84 https://www.sasbdb.org
Grp1 14-399 + IP4 NS-EM Volume 1 with best antiparallel model	This paper	EMDB: EMD-20628<br>PDB: 6U3E http://www.emdatabank.org
Grp1 14-399 + IP4 NS-EM Volume 2 with best antiparallel model	This paper	EMDB: EMD-20629<br>PDB: 6U3G http://www.emdatabank.org
**Recombinant DNA**		
Plasmid: Modified pET15 (pDL2)	DiNitto et al., 2007	N/A
Mouse Grp1 14-399 in pDL2	DiNitto et al., 2007	N/A
Mouse ARNO 2-400 (diglycine variant) in pDL2	DiNitto et al., 2007	N/A
Human FL ARNO 3G pET-8c	Antonny et al., 1997	N/A

(*Continued on next page*)

Continued

REAGENT or RESOURCE	SOURCE	IDENTIFIER
Human ARNO 3-299 pET-8c	Antonny et al., 1997	N/A
Arf1 pET-3c	Franco et al., 1993	N/A
Software and Algorithms		
ADP_EM	Garzón et al., 2007	http://chaconlab.org/hybrid4em/adp-em
ATSAS	Petoukhov et al., 2012	www.embl-hamburg.de/biosaxs/software.html
CHIMERA	Pettersen et al., 2004	https://www.cgl.ucsf.edu/chimera/download.html
CORAL	Petoukhov et al., 2012	www.embl-hamburg.de/biosaxs/software.html
DAMAVER (DAMSEL, DAMSUP, DAMAVER and DAMFILT)	Volkov and Svergun, 2003	www.embl-hamburg.de/biosaxs/software.html
DAMMIF	Franke and Svergun, 2009	www.embl-hamburg.de/biosaxs/software.html
DELA	Malaby et al., 2015	DOI: 10.1107/S1600576715010420
EMAN2	Tang et al. (2007)	http://blake.bcm.edu/emanwiki/EMAN2
FoXS	Schneidman-Duhovny et al., 2013	https://integrativemodeling.org
GASBOR	Svergun et al., 2001	www.embl-hamburg.de/biosaxs/software.html
GNOM	Svergun (1992)	www.embl-hamburg.de/biosaxs/software.html
IMOD	Kremer et al. (1996)	http://bio3d.colorado.edu/imod/
IMP	Russel et al., 2012	https://integrativemodeling.org
MODELLER	Webb and Sali, 2014	https://salilab.org/modeller/
MultiFoXS	Carter et al., 2015	https://integrativemodeling.org
PRIMUS	Konarev et al., 2003	www.embl-hamburg.de/biosaxs/software.html
PyMol	SBGRID	https://pymol.org
RRT_SAMPLE	Raveh et al., 2009	https://integrativemodeling.org
SBGRID	Morin and Sliz, 2013	https://sbgrid.org
SUPCOMB	Kozin and Svergun, 2001	www.embl-hamburg.de/biosaxs/software.html
dammif.sh	Malaby et al., 2018	N/A
e2classvsproj.py	EMAN2	http://blake.bcm.edu/emanwiki/EMAN2
e2classesvsprojs.py	Malaby et al., 2018	N/A
e2classesvsprojs_best_scores.py	Malaby et al., 2018	N/A
e2classesvsprojs_extract_best.py	Malaby et al., 2018	N/A
e2classesvsprojs_generate_best_list.py	Malaby et al., 2018	N/A
e2pdb2mrc.py	EMAN2	http://blake.bcm.edu/emanwiki/EMAN2
e2pdbs2mrcs.py	Malaby et al., 2018	N/A
e2classesvsprojs_pipeline.txt	Malaby et al., 2018	N/A
extract_models.sh	This paper	N/A
extract_rg.sh	This paper	N/A
filenames_rg.py	This paper	N/A
foxs.sh	Malaby et al., 2018	N/A
foxs_component_summation_resample.sh	This paper	N/A
foxs_component_summation.py	This paper	N/A
foxs_resample.py	This paper	N/A
gasbor.sh	Malaby et al., 2018	N/A
histogram.py	This paper	N/A
histogram_fraction.py	This paper	N/A
multifoxs_filenames.py	This paper	N/A
multifoxs_mem_pipeline.txt	This paper	N/A
multifoxs_pipeline.txt	Malaby et al., 2018	N/A
Other		
HiTrap Q HP	GE Healthcare Life Sciences	Cat#17-1154-01
HiTrap SP HP	GE Healthcare Life Sciences	Cat#17115201

(*Continued on next page*)

Continued		
REAGENT or RESOURCE	SOURCE	IDENTIFIER
His-Trap HP	GE Healthcare Life Sciences	Cat#17-5248-02
Gilder Copper grids, 400 Mesh	Ted Pella	Cat#G400
Half Area 96 Well Microplate	Corning	Cat#3679
HiLoad Superdex 75 PG 16/60	GE Healthcare Life Sciences	Cat#28989333
HiLoad Superdex 200 PG 16/60	GE Healthcare Life Sciences	Cat#28989335
Superdex 200 Increase 5/150	GE Healthcare Life Sciences	Cat#28990945

LEAD CONTACT AND MATERIALS AVAILABILITY

Further information and requests for resources and reagents should be directed to and will be fulfilled by the Lead Contact, David Lambright (David.Lambright@umassmed.edu). This study did not generate new unique reagents.

EXPERIMENTAL MODEL AND SUBJECT DETAILS

Constructs of the diglycine splice variants of mouse Grp1 and ARNO with N-terminal 6xHis tags were purified after heterologous expression in the bacterial strain BL21(DE3).

METHOD DETAILS

Constructs, Expression and Purification

Constructs corresponding to the diglycine variants of Grp1 and $ARNO_{2\text{-}400}$ were amplified using Vent polymerase, digested with BamHI/SalI, and ligated into modified pET15b vectors that incorporate an N-terminal his tag (MGHHHHHHGS) (DiNitto et al., 2007). BL21(DE3) cells (Novagen) transformed with the plasmids were grown in 2xYT supplemented with 100 mg/L ampicillin to OD_{600} 0.2-0.4 and induced with 50 μM IPTG for 14-18 hrs at 18°C. Cells pellets were resuspended in buffer (50 mM Tris, pH 8.0, 150 mM NaCl, 2 mM $MgCl_2$, 0.1% 2-mercaptoethanol) and incubated with 0.1 mM PMSF, 0.2 mg/ml lysozyme, and 0.01 mg/ml protease free DNAse I (Worthington). Lysates were sonicated, centrifuged at 30,000×g for 1 hr with 0.5% Triton X-100 and purified over Ni-NTA followed by ion exchange with HiTrap Q, and gel filtration on Superdex-200 (GE Healthcare).

Myristoylated Arf1 (myrArf1) was co-expressed in *Escherichia coli* with yeast N-myristoyl transferase (NMT) and purified as described previously (Beraud-Dufour et al., 1998). Full-length ARNO carrying a 3G sequence in the membrane-binding site ($ARNO_{FL}$), which binds $PI(4,5)P_2$ and $PI(3,4,5)P_3$ equally (Klarlund et al., 2000) and a construct truncated for the N-terminal CC domain (residues 50-400; $ARNO\Delta^{Nt}$) were cloned into pET-8c vector (kind gift of Bruno Antonny, CNRS, Sophia-Antipolis, France) and overexpressed in *Escherichia coli*. Untagged $ARNO\Delta^{Nt}$ was purified as described previously (Peurois et al., 2017). Expression of $ARNO_{FL}$, which carries a N-terminal 7-His tag, was induced for 3h at 37°C by addition of 0.5 mM of IPTG. Cell pellets were resuspended in 20 mM $NaPO_4$ pH 7.4, 500 mM NaCl, 2 mM betamercaptoethanol and 10 mM imidazole, and then disrupted using a French press. The cleared lysate supernatant was first purified by a Ni-NTA affinity chromatography step (HisTrap FF, GE Healthcare) and then submitted to a Superdex 200 16/600 column (GE Healthcare) equilibrated with 20 mM Tris pH 7.5, 250 mM NaCl and 2 mM betamercaptoethanol. Proteins were more than 95% pure as judged by SDS-PAGE analysis.

SEC-SAXS Data Collection and Processing

SEC-SAXS data sets for Grp1 constructs and $ARNO_{2\text{-}400}$ were collected at the BioCAT Sector 18-ID beamline at the Argonne National Laboratory Advanced Photon Source. Samples were incubated with a 1.2 molar excess of inositol 1,3,4,5-tetrakis phosphate (IP_4) for 1-5 hrs, concentrated to 10-20 mg/ml and injected onto 3 ml Superdex-200 Increase columns (GE Healthcare) equilibrated with 20 mM Tris, pH 8.0, 150 mM NaCl, 2 mM $MgCl_2$, 0.1% 2-mercaptoethanol, 5% glycerol, and 1 μM IP_4. Column outlets were connected to the flow cell and SAXS data sets acquired with 1 s exposures at 5 s intervals during elution. Raw SAXS images were radially averaged on a log scale over the q range 0.00621-0.333 $Å^{-1}$, normalized by the incident beam intensity, and further processed to reconstruct scattering profiles for the protein by buffer subtraction with or without automatic determination of an optional scaling constant or by singular value decomposition and linear combination (SVD-LC) as described (Malaby et al., 2015). The SVD-LC profiles typically had higher signal-to-noise, fewer subtraction artifacts, and were used for subsequent analyses. SEC-SAXS data sets for $Grp1_{63\text{-}399}$ and $Grp1_{63\text{-}390}$ were collected and processed as described previously (Malaby et al., 2018).

$ARNO\Delta^{Nt}$ data were collected using an inline HPLC-coupled SAXS instrument (SWING beamline, SOLEIL Synchrotron, France). 350 μg $ARNO\Delta^{Nt}$ was injected in a 40 μL volume (8 mg/ml) into a size exclusion chromatography column (Bio SEC-3 300 Å, Agilent Technologies, Inc.) equilibrated with elution buffer (20 mM Tris pH 8.0, 150 mM NaCl and 1 mM DTT), prior to the SAXS data acquisition. The buffer scattering signal was recorded for the first 90 images, then 240 images for the sample. SAXS images were processed with the FOXTROT suite (SOLEIL synchrotron, SWING beamline) to generate individual curves. Data intensity and quality

was evaluated by plotting the I(0) and R_G as a function of frames. Curves from consecutive images showing high intensity I(0) and similar R_G were averaged. $ARNO_{FL}$ data were collected at BM29 beamline, ESRF, France. Images were recorded throughout the HPLC elution process using the Bio Sec300 column. 600 μg in 60 μL volume was injected. Data reduction to absolute unit, subtraction and averaging was done with the EDNA pipeline implemented in the ISPyB software (ESRF BM29 beamline).

Basic SAXS Analyses and Ab Initio Modeling

For the diglycine variants of Grp1 and $ARNO_{2\text{-}400}$, Guinier analyses and dimensionless Kratky plots (Durand et al., 2010) were calculated in DELA (Malaby et al., 2015). P(r) distributions were calculated using GNOM (Svergun, 1992) in PRIMUS (Konarev et al., 2003) and MEM with a sine prior in DELA (Malaby et al., 2015, 2018). *Ab initio* bead envelopes were calculated using DAMMIF (Franke and Svergun, 2009) and GASBOR (Svergun et al., 2001). Typically, 100 bead envelopes were averaged/filtered in groups of 10 using DAMAVER (Volkov and Svergun, 2003) and the process repeated on the averaged/filtered models to generate the final models, which were aligned with atomic coordinates using SUPCOMB (Kozin and Svergun, 2001).

For the triglycine variants of ARNO, SAXS data analyses were performed with the ATSAS 2.8.3 package (Franke et al., 2017). Radii of gyration (R_G) were evaluated by Guinier Wizard using the data within the range of Guinier approximation sR_G<1.3 and by Distance Distribution Wizard, both of which are modules of the PRIMUS program. The maximum distance D_{max} was estimated with PRIMUS and refined by trial and error with GNOM. The distance distribution functions P(r) were calculated with GNOM. The dimensionless Kratky plot was calculated by plotting $(qR_G)^2I(q)/I(0)$ against qR_G. Molecular weights were estimated by Primus Molecular Weight Wizard using different algorithms. The fit between scattering amplitude calculated for the crystal structure of autoinhibited $Grp1\Delta^{Nt}$ (residues 63-399) and the SAXS curve of $ARNO\Delta^{Nt}$ was calculated with CRYSOL. *Ab initio* envelopes were calculated with GASBOR and DAMMIN over the q range 0.0025-0.5 $Å^{-1}$ for $ARNO_{FL}$ and 0.01-0.600 $Å^{-1}$ for $ARNO\Delta^{Nt}$. P2 symmetry was imposed for ARNO. The resulting models were further compared using SUPCOMB and clustered with DAMCLUST. The consensus of the calculated models was represented by the lowest Normalized Spatial Discrepancy (NSD), which was determined by DAMSEL. The comparison of models and superposition was performed with SUPCOMB.

Rigid Body and Ensemble Modeling

Models for the autoinhibited and active cores were derived from the crystal structure of $Grp1_{63\text{-}399}$ (PDB: 2R09) or the most frequent model in the minimal three state MultiFoXS ensemble for $Grp1_{63\text{-}390}$, respectively. CC models were generated with CCBuilder (Wood et al., 2014). The CC-Sec7 domain linker and missing terminal residues were built with MODELLER (Webb and Sali, 2014) in CHIMERA (Pettersen et al., 2004). Rigid body modeling was performed with CORAL (Petoukhov et al., 2012). For ensemble and MEM analyses, pools of 10,000 models for each chain/topology were generated with RRT_SAMPLE (Raveh et al., 2009). The head group was represented as atoms in glycine residues that retained the chemical information in the last column of the PDB file required to specify the correct scattering form factors. Components for partial scattering profiles were calculated with FoXS (Schneidman-Duhovny et al., 2013) and minimal best fitting ensembles determined using MultiFoXS (Carter et al., 2015). For MEM distributions, scattering profiles were calculated by summation of the components generated by FoXS, with coefficients for the best-fitting MultiFoXS ensemble, as described (Schneidman-Duhovny et al., 2013) and resampled with linear interpolation to match the q sampling of the experimental profile. MEM distributions were calculated with an unbiased prior in DELA (Malaby et al., 2015) as described (Skilling and Bryan, 1984).

Electron Microscopy Sample Preparation and Negative Staining

$Grp1_{14\text{-}399}$ was incubated with IP_4 for 2 hrs prior to concentration and size exclusion chromatography on a Superdex-200 column equilibrated with 20 mM Tris, pH 8.0, 150 mM NaCl, 0.1% 2-mercaptoethanol, and 1 μM IP_4. Protein from the peak fraction was immediately diluted, applied to glow discharged carbon coated Gilder copper 400 mesh grids (Ted Pella), incubated for 1 min, rinsed with deionized water, and stained with 0.75% (w/v) uranyl formate (EM Sciences) as described (Booth et al., 2011). Images were acquired on a Philips CM120 electron microscope operated at 120 kV using a TVIPS 2k x 2k CCD (TemCam-F224HD) camera with a nominal magnification of 28,000, corresponding to a calibrated pixel size of 6.5 Å at the specimen level. A total of 500 micrographs were collected with a nominal defocus range of -1.2 to -3.2 μm and a low dose of ~30 electrons/Å.

Image Processing, Particle Picking and 2D Classification

Images were processed with EMAN2 (Tang et al., 2007) after X-ray removal with IMOD (Kremer et al., 1996). Approximately 10,000 particles were manually picked with a box size of 80×80 pixels. Following contrast transfer function (CTF) fitting and preprocessing of extracted images, particle sets were built. One hundred 2D class averages were generated by unsupervised reference-free classification. After discarding poor quality classes, 53 classes comprising 6504 particles remained.

2D Heterogeneity Analysis

Heterogeneity within 2D classes was analyzed using the 2D heterogeneity module in the 3D refinement section of EMAN2. Particles for similar classes were grouped to generate new sets, which were reclassified by reference free class averaging with the center set to the center of mass.

3D Volume Reconstruction and Refinement

Initial models were built for two particle sets consisting of 20 (Volume 1) or 15 (Volume 2) classes selected on the basis of qualitative similarity in the overall size and shape of the best fitting model projections for each class. Final 3D refinement with full CTF correction against the starting models was carried out by the gold-standard procedure in EMAN2 without imposed symmetry (i.e. C1). The resolution of the final 3D reconstructions was conservatively estimated to be 53 Å based on a Fourier shell correlation (FSC) cut-off of 0.5 (Rosenthal and Henderson, 2003). The refined 3D volumes were validated by EMAN2 validation methods.

Comparison of 2D Class Averages and 3D Volumes with Atomic Resolution Models

Atomic resolution models were converted to 40 Å resolution volumes with the e2pdb2mrc.py and volume projections compared with 2D class averages at 10° increments using e2classsvsproj.py. Two python scripts (e2pdbs2mrcs.py and e2classesvsprojs_best_scores.py) were previously developed to automate these steps and rank order models based on best scoring projections for each class (Malaby et al., 2018). For comparison with 3D volumes, automated rigid body docking of models at a resolution of 40 Å was performed with ADP_EM (Garzón et al., 2007). Models and volumes were visualized in Chimera (Pettersen et al., 2004).

Liposomes

All lipids were obtained from Avanti Polar Lipids. Liposomes were prepared as described previously (Aizel et al., 2013; Stalder et al., 2011) in a buffer containing 50 mM HEPES pH 7.4 and 120 mM potassium acetate. All liposomes contained 37.9% phosphatidylcholine (PC), 20% phosphatidylethanolamine (PE), 20% phosphatidylserine (PS), 20% cholesterol, 0.1 % NBD-PE and 2% phosphatidylinositol-4,5-triphosphate (PIP_2) and were extruded through a 0.2 μm filter (Whatman). For kinetics assays, NBD-PE was omitted and PC was adjusted to 38%.

Nucleotide Exchange Kinetics

Kinetics of activation of myristoylated Arf1 were monitored at 37°C by tryptophan fluorescence (emission/excitation wavelengths of 292/340 nm) in 50 mM HEPES pH 7.4, 120 mM potassium acetate, 1 mM $MgCl_2$ and 1 mM DTT (HKM buffer). 100 μM of liposomes were incubated for 2 minutes at 37°C, before the addition of ARNO constructs at different concentrations and 0.4 μM myristoylated Arf1. Nucleotide exchange was initiated by addition of 150 μM GTP.

DLS Experiments

Dynamic Light Scattering (DLS) experiments were performed on a DynaPro NanoStarTM instrument (Wyatt Technology). 1 mM of liposomes were incubated without or with 3 μM of the indicated protein at room temperature prior to analysis by DLS as described previously (Benabdi et al., 2017).

HDX-MS Experiments

$ARNO_{FL}$ was diluted in HKM buffer to 1 μM and incubated for 10 min with or without 100 μM of liposomes prior addition of D_2O. Deuterium exchange reactions were initiated by diluting the protein in D_2O (99.8% D_2O ACROS, Sigma, UK) in 25 mM HEPES pH 7.5, 125 mM potassium acetate, 1 mM TCEP to give a final D_2O percentage of ~95%. Deuterium labelling was carried out at 23°C (unless otherwise stated) at five time points: 0.3 (3 seconds on ice), 3, 30, 300 and 3000 seconds. The labelling reaction was quenched by the addition of chilled 2.4% v/v formic acid in 2M guanidinium hydrochloride and immediately frozen in liquid nitrogen. Samples were stored at -80°C prior to analysis. Each experiment was performed in triplicate.

The quenched protein samples were rapidly thawed and subjected to proteolytic cleavage with pepsin followed by reversed phase HPLC separation. Briefly, the protein was passed through an Enzymate BEH immobilized pepsin column, 2.1 x 30 mm, 5 μm (Waters, UK) at 200 μL/min for 2 min, the peptic peptides were trapped and desalted on a 2.1 x 5 mm C18 trap column (Acquity BEH C18 Van-guard pre-column, 1.7 μm, Waters, UK). Trapped peptides were subsequently eluted over 11 min using a 3-43% gradient of acetonitrile in 0.1% v/v formic acid at 40 μL/min. Peptides were separated on a reverse phase column (Acquity UPLC BEH C18 column 1.7 μm, 100 mm x 1 mm (Waters, UK) and detected on a SYNAPT G2-Si HDMS mass spectrometer (Waters, UK) over a m/z of 300 to 2000, with the standard electrospray ionization (ESI) source with lock mass calibration using [Glu1]-fibrino peptide B (50 fmol/μL). The mass spectrometer was operated at a source temperature of 80°C and a spray voltage of 2.6 kV. Spectra were collected in positive ion mode.

Peptide identification was performed by MSe (Silva et al., 2006) using an identical gradient of increasing acetonitrile in 0.1% v/v formic acid over 11 min. The resulting MSe data were analyzed using Protein Lynx Global Server software (Waters, UK) with an MS tolerance of 5 ppm.

Mass analysis of the peptide centroids was performed using the DynamX HDX data analysis software 3.0 (Waters, UK). Only peptides with a score > 6.4 were considered. The first round of analysis and identification was performed automatically by the DynamX software, however, all peptides (deuterated and non-deuterated) were manually verified at every time point for the correct charge state, presence of overlapping peptides, and correct retention time. Deuterium incorporation was not corrected for back-exchange and represents relative, rather than absolute changes in deuterium levels. Changes in H/D amide exchange in any peptide may be due to a single amide or a number of amides within that peptide.

Software Resources

Software available through the SBGRID Consortium was used for supported applications (Morin and Sliz, 2013).

QUANTIFICATION AND STATISTICAL ANALYSIS

SAXS Profiles

Errors for SAXS profiles reconstructed by SVD-LC were estimated as the root mean squared deviation of the residuals for the fit with a maximum entropy model for the discretized inverse pair-distribution transformation

$$I(q) = 4\pi \sum P(r)\ \sin(qr)/qr$$

calculated on a real space grid of 1 Å over the range from 0.01 Å to an upper limit approximately 10-20% larger than D_{max}. The informational entropy was calculated using a sine function on the interval 0-π radians as the prior distribution. The χ^2 values reported here thus reflect the quality of fits with *ab initio*, rigid body and ensemble models compared to the nearly ideal best fit attainable with the maximum entropy inverse pair-distribution model. This approach for estimating errors avoids non-trivial and likely inaccurate error propagation associated with SVD-LC reconstruction of SAXS profiles.

Comparison with Class Averages and Volumes

Correlation coefficients and scoring functions for comparison of 2D class averages and 3D volumes with projections and volumes derived from atomic coordinates are presented as calculated by the software applications described in the Method Details and references therein.

Nucleotide Exchange Kinetics

All experiments were performed in duplicate, and means of two independent experiments are given ± the standard deviation (s.d.). k_{obs} were determined from monoexponential fits and k_{cat}/k_M were calculated by linear regression of kobs values as a function of GEF concentration as described in (Aizel et al., 2013).

DATA AND CODE AVAILABILITY

Data and Model Depositions

SAXS profiles, P(r) distributions, fits and models have been deposited with the Small Angle Scattering Biological Data Bank (Valentini et al., 2015) under the accession codes SASBDB: SASDEV9 (Cytohesin-2; ARF nucleotide-binding site opener, ARNO truncation mutant), SASBDB: SASDEW9 (Cytohesin-2; ARF nucleotide-binding site opener, ARNO), SASBDB: SASDG64 (Grp1 14-399 + IP4 SAXS with DAMMIF and GASBOR models), SASBDB: SASDG94 (Grp1 14-399 + IP4 SAXS with antiparallel CORAL and MultiFoXS models), SASBDB: SASDGA4 (Grp1 14-399 + IP4 SAXS with parallel CORAL and MultiFoXS models), SASBDB: SASDG74 (Grp1 14-390 + IP4 SAXS with DAMMIF and GASBOR models), SASBDB: SASDGB4 (Grp1 14-390 + IP4 SAXS with antiparallel CORAL and MultiFoXS models), SASBDB: SASDGC4 (Grp1 14-390 + IP4 SAXS with parallel CORAL and MultiFoXS models), SASBDB: SASDG84 (ARNO 2-400 + IP4 SAXS with DAMMIF, GASBOR and antiparallel CORAL models). EM envelopes have been deposited with the EM Data Bank (Lawson et al., 2016) under the accession codes EMDB: EMD-20628 (Grp1 14-399 + IP4 NS-EM Volume 1 with best antiparallel model) and EMDB: EMD-20629 (Grp1 14-399 + IP4 NS-EM Volume 2 with best antiparallel model). The best-fitting MultiFoXS models selected by ADP_EM have been deposited with the Protein Data Bank (Berman et al., 2000) under the accession codes PDB: 6U3E (Grp1 14-399 + IP4 NS-EM Volume 1 with best antiparallel model) and PDB: 6U3G (Grp1 14-399 + IP4 NS-EM Volume 2 with best antiparallel model). Accession codes are also included in Key Resources Table. Other data and models are available on request to the Lead Contact.

Software

The Mac OSX application DELA and associated Python scripts for processing and analysis of SEC-SAXS data sets and SAXS profiles have been described previously (Malaby et al., 2015). Python scripts (.py), bash shell scripts (.sh), and "pipelines" (_pipeline.txt) for SAXS and EM analyses described below can be downloaded as a zip file (Data S1), which also includes the application bundle and associated Python scripts for DELA. This version of DELA supports calculation of MEM distributions using model profiles derived from MultiFoXS pools. Although the scripts and pipelines are distributed as Open Source (https://opensource.org), the command line tools, programs or source code executed by these automation scripts are subject to the licensing terms of the relevant packages.

Shell Scripts

calculate_extract_rg.sh

Automates calculation and extraction of Rg values using the IMP program rg.

dammif.sh

Automates generation of ab initio bead models with DAMMIF, systematic pairwise alignment and selection with DAMSEL, alignment against the most representative bead model with DAMSUP, 'averaging' with DAMAVER, filtering with DAMFILT, and generation of an input file for DAMMIN with DAMSTART.

extract_models.sh
Automates extraction of individual models from multi model pdb files.
foxs.sh
Automates calculation of SAXS profiles using the command line version FoXS. Can be run in parallel batches.
foxs_component_summation_resample.sh
Automates summation of FoXS partial profiles and resampling to match data q values.
gasbor.sh
Equivalent to dammif.sh except that generation of ab initio bead models is done with GASBOR.

Python Scripts
e2pdbs2mrcs.py
Automates generation of volumes from atomic coordinates using the EMAN2 python script e2pdb2mrc.py. Can be run in parallel batches.
e2classesvsprojs.py
Automates comparison of class averages with volume projections using the EMAN2 python script e2classvsproj.py. Can be run in parallel batches.
e2classesvsprojs_best_scores.py
Identifies the best score and volume projection for each class average as well as the overall best score and volume projection for all class averages using the output of e2classesvsprojs.py.
e2classesvsprojs_extract_best.py
Extracts the best scoring coordinate files and corresponding image stacks using the output of e2classesvsprojs_best_scores.py.
e2classesvsprojs_generate_best_list.py
Generates a list of the images for the best scoring volume projection versus class average comparisons using the output of e2classesvsprojs_best_scores.py. The resulting list in "fast LST format" can be used as input for compilation of the images into an image stack in EMAN2.
extract_rg.py
Extracts Rg values embedded in a text file containing output generated by the IMP program rg.
filenames_rg.py
Combines filenames from one file with Rg values from another.
foxs_component_summation.py
Sums FoXS partial profiles using c1 and c2 constants from MultiFoXS.
foxs_resample.py
Resamples a FoXS profile to match q values from a reference profile using linear interpolation.
histogram_fractions.py
Generates a histogram of values with corresponding fractions after sorting in ascending order.
histogram.py
Generates a histogram of values after sorting in ascending order.
multifoxs_filenames.py
Generates a file containing the filenames for input to the command line version of multi_foxs.

Pipelines
The following "pipelines" are intended to illustrate the sequence of command line tools and scripts. Although they can be converted to a fully automated shell script if desired, we prefer to run the instructions individually to allow the output at each step to be monitored for quality control.
e2classesvsprojs_pipeline.txt
Example "pipeline" illustrating sequence of command line instructions used for comparison of 2D class averages with volume projections calculated from a pool of models generated by RRT_SAMPLE.
multifoxs_pipeline.txt
Example "pipeline" illustrating the sequence of command line instructions used for Multi_FoXS model generation, profile calculation and analysis with the IMP command line tools RRT_SAMPLE, foxs, and multi_foxs.
multifoxs_mem_pipeline.txt
Example "pipeline" illustrating the sequence of command line instructions used to prepare Multi_FoXS output for MEM in DELA. This "pipeline" requires partial profiles from FoXS (with -p option) and uses constants (c1 and c2) from MultiFoXS. The required partial profiles and constant values are available after the multifoxs_pipeline.txt "pipeline" completes.

Structure, Volume *27*

Supplemental Information

Structural Organization and Dynamics of Homodimeric Cytohesin Family Arf GTPase Exchange Factors in Solution and on Membranes

Sanchaita Das, Andrew W. Malaby, Agata Nawrotek, Wenhua Zhang, Mahel Zeghouf, Sarah Maslen, Mark Skehel, Srinivas Chakravarthy, Thomas C. Irving, Osman Bilsel, Jacqueline Cherfils, and David G. Lambright

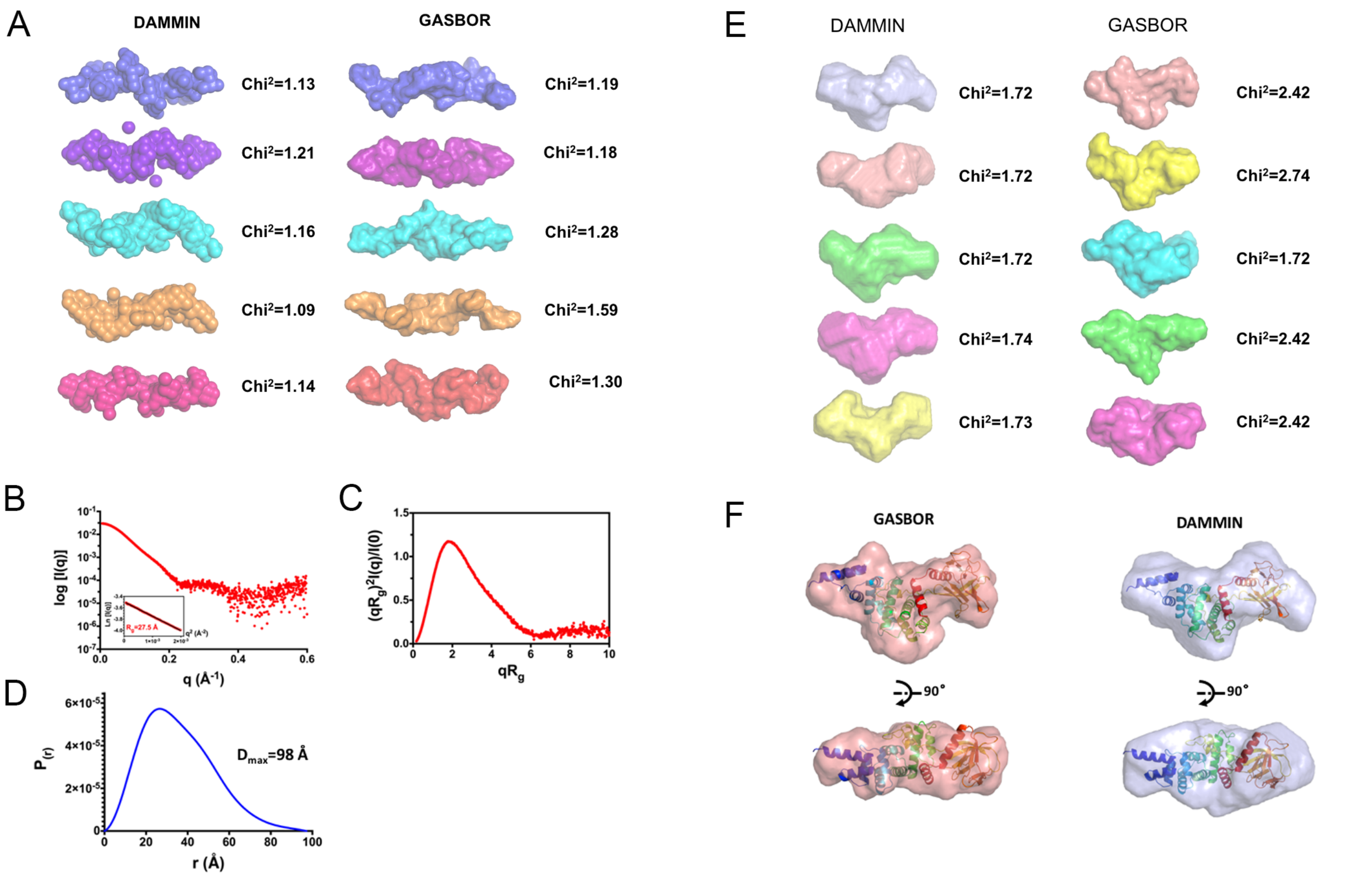

Figure S1. SAXS analyses of ARNOΔ^{Nt} and ARNO$_{FL}$. Related to Figure 1
(A) SAXS profile of ARNOΔ^{Nt}. The insert shows the Guinier plot ($R_g \times q_{max}$ = 1.22). (B) The dimensionless Kratky plot analysis shows a fully globular protein. (C) The P(r) plot analysis gives an estimated D_{max} of 98 Å. (D) Envelopes calculated with GASBOR and DAMMIN. (E) Fit of autoinhibited GRP1 structure in a representative ARNOΔ^{Nt} envelope. (F) Additional envelopes of ARNO$_{FL}$ calculated with GASBOR and DAMMIN. The Chi2 values are indicated.

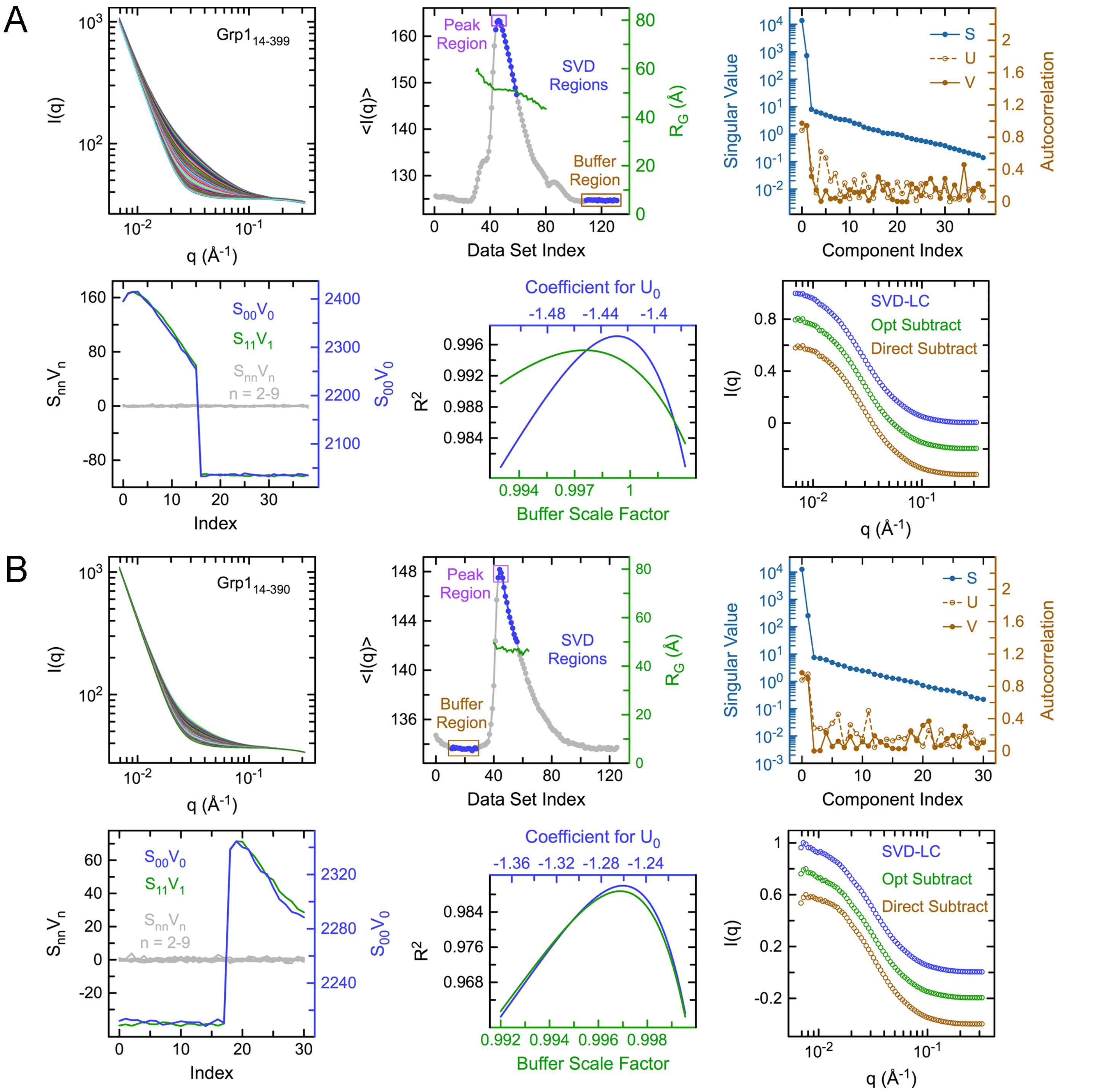

Figure S2. SEC-SAXS Analysis and Reconstruction of Protein Scattering for Dimeric Grp1 Constructs with IP_4. Related to Figure 1

(A) Autoinhibited $Grp1_{14\text{-}399}$. (B) Fully active $Grp1_{14\text{-}390}$. (A and B) *Upper left*, SAXS profiles after radial averaging and normalization. *Upper middle*, mean scattering during elution with regions used for buffer subtraction or SVD indicated. Also shown are R_G values from Guinier analysis of buffer subtracted scattering profiles. *Upper right*, singular values (diagonal elements of the diagonal matrix **S**) and autocorrelations for the rank ordered components (columns of the matrix **U**) and corresponding variable coefficients (columns of the symmetric matrix **V**) after singular value decomposition (SVD; $\mathbf{A} = \mathbf{U} \bullet \mathbf{S} \bullet \mathbf{V}^T$) of the SAXS profiles (columns of the matrix **A**). *Lower left*, Singular value-weighted columns of **V**, where n is the component index and the x-axis corresponds to the index of the SAXS data sets used for SVD. *Lower middle*, analysis of linearity in the Guinier region as a function of the buffer scale factor for optimized buffer subtraction or the U_0 coefficient for linear combination of the two most significant SVD components (SVD-LC). *Lower right*, comparison of methods for reconstruction of the protein scattering profile as $c * U_0 + U_1$ (SVD-LC), <I(q) peak region> - c * <I(q) buffer region(s)> (Opt Subtract), or <I(q) peak region> - <I(q) buffer region(s)> (Direct Subtract).

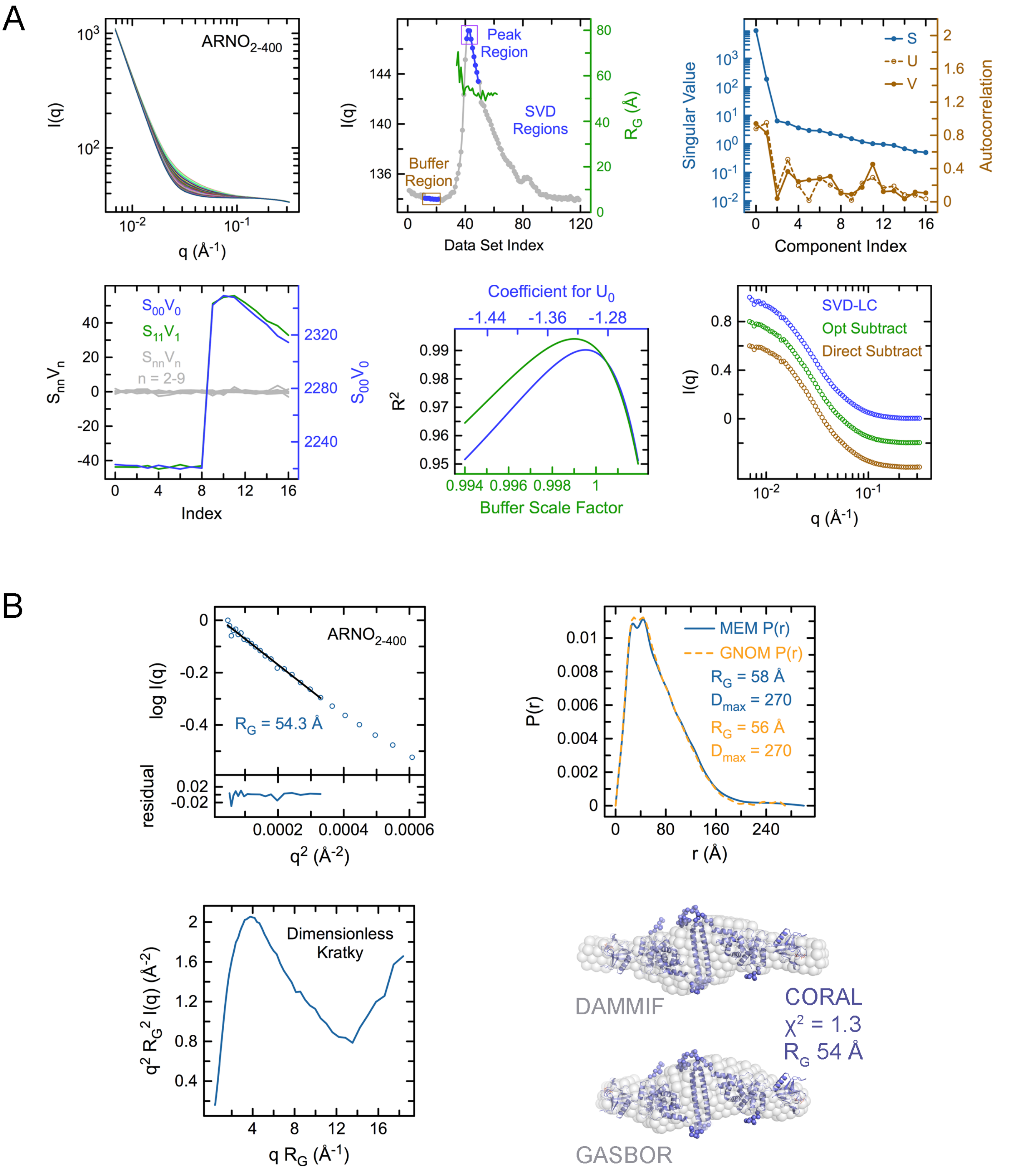

Figure S3. SEC-SAXS Analysis for Homodimeric ARNO$_{2\text{-}400}$ with IP$_4$. Related to Figure 2

(A) SEC-SAXS and reconstruction of protein scattering profiles for homodimeric ARNO$_{2\text{-}400}$ bound to IP$_4$. See Figure S2 legend for description of panels. (B) Basic SAXS analyses and *ab initio* models. *Upper left*, Guinier plot and fit. *Upper right*, MEM and GNOM P(r) distributions. *Lower left*, dimensionless Kratky plot. *Lower right*, *ab initio* models calculated with DAMMIF and GASBOR. Also shown is the rigid body CORAL model based on the Grp1 autoinhibited core and antiparallel coiled coil.

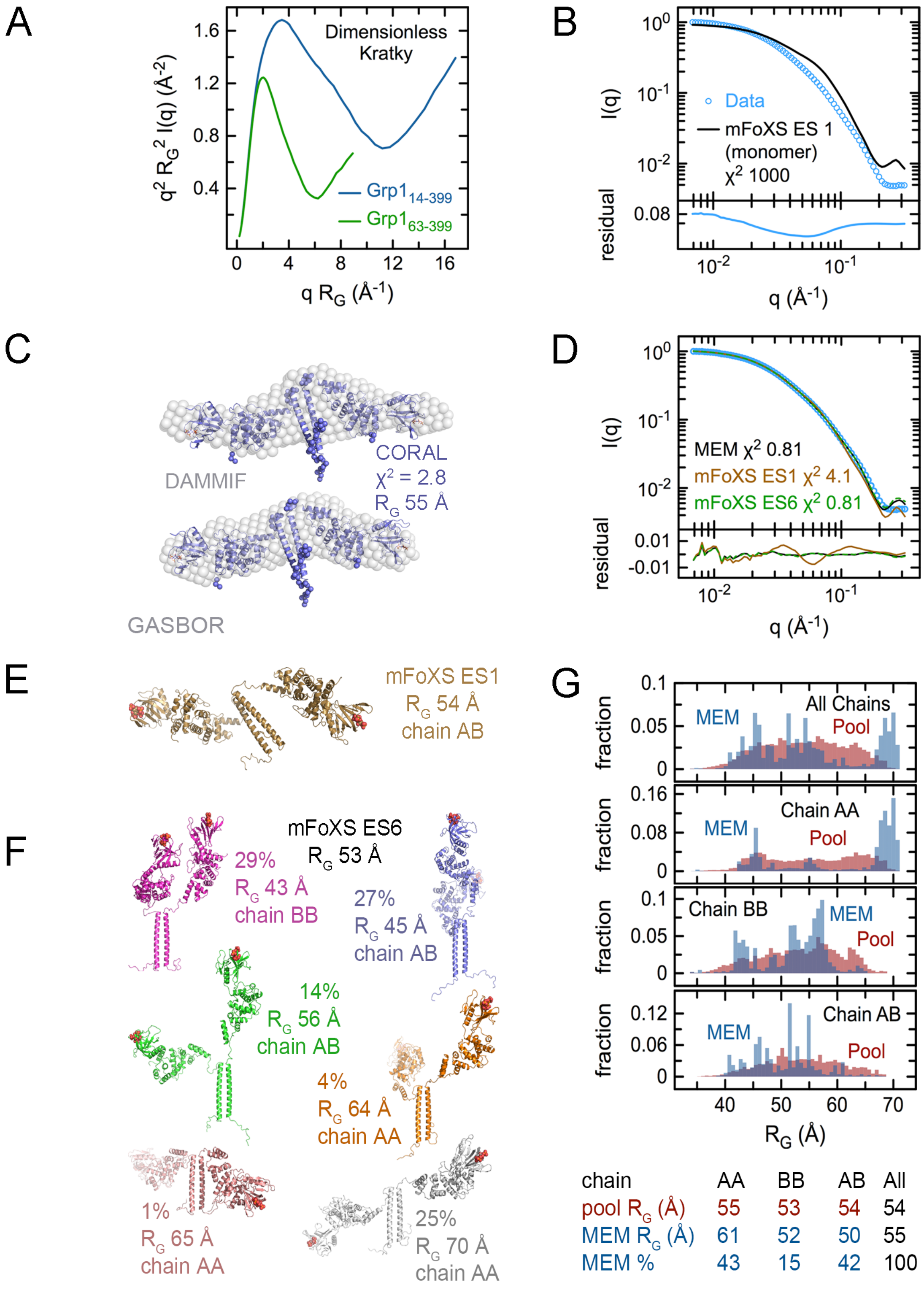

Figure S4. SAXS Analyses of Autoinhibited Grp1 Dimers. Related to Figure 2
(A) Dimensionless Kratky plot for autoinhibited Grp1 constructs with or without the CC. (B) Experimental SAXS profile and best-fitting single model MultiFoXS profile for a monomer pool. No multistate ensembles were identified. (C) *Ab initio* models calculated with DAMMIF or GASBOR and aligned with the rigid body CORAL model for the parallel CC dimer. (D) Comparison of the experimental SAXS profile with the profiles for the best-fitting single model (ES1) and multiple model (ES6) MultiFoXS ensembles as well as the all model MEM distribution for the parallel CC dimer. (E) Best-fitting single state MultiFoXS model (ES1) for the parallel CC dimer. (F) Models for the best-fitting MultiFoXS ensemble (ES6) for the parallel CC dimer with percentages and R_G values. The overall R_G for the ensemble was calculated as the fraction-weighted mean of the individual R_G values. (G) Fraction-weighted histograms of R_G values for the MEM distribution and pool for the parallel CC dimer. Fraction-weighted mean R_G values and percentages are tabulated below.

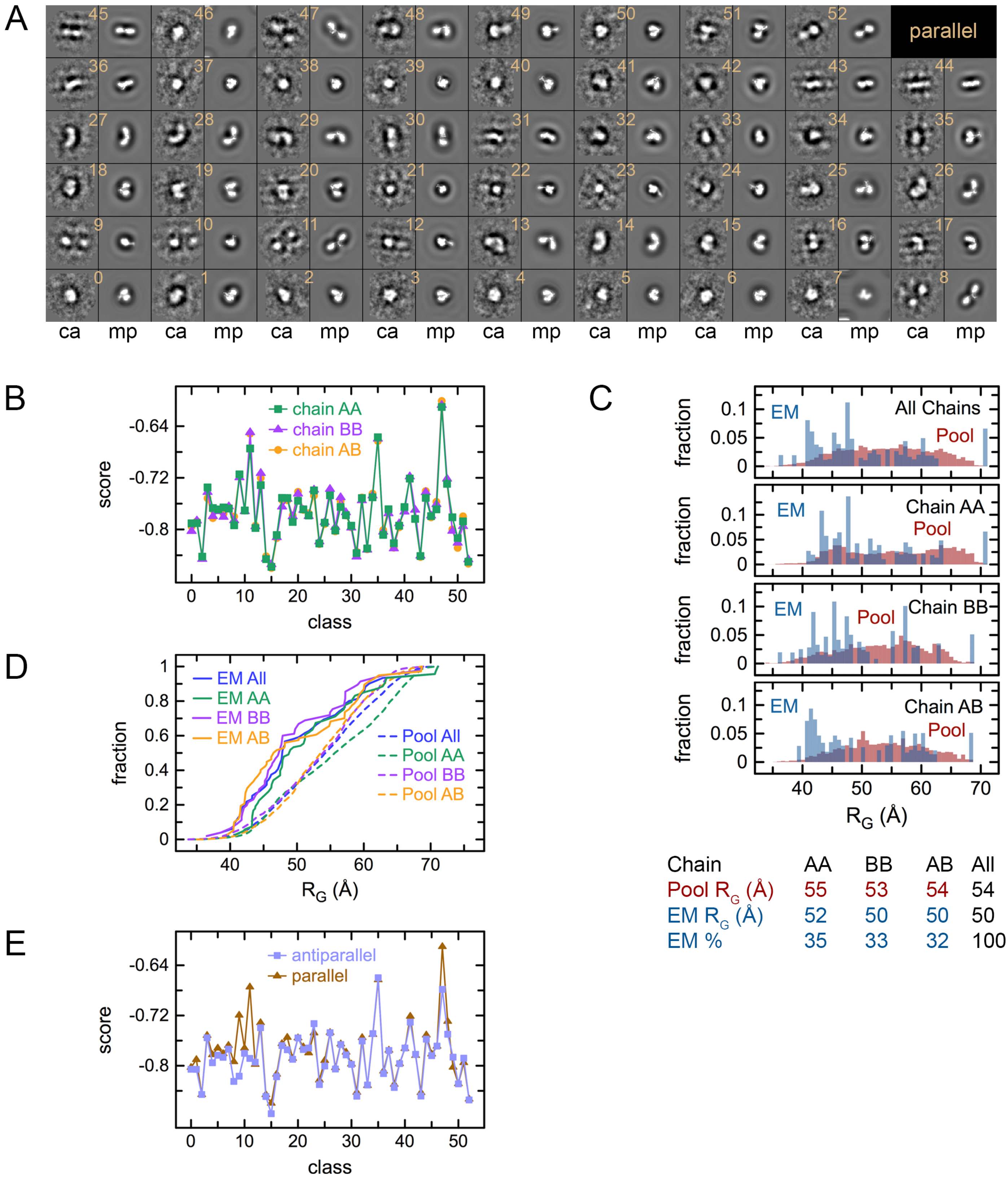

Chain	AA	BB	AB	All
Pool R_G (Å)	55	53	54	54
EM R_G (Å)	52	50	50	50
EM %	35	33	32	100

Figure S5. Projection Matching Analysis with Parallel MultiFoXS Models. Related to Figure 4
(A) Class averages compared with the best scoring 3D volume projection from the models in the MultiFoXS pools. (B) Scores for comparison of class averages with the best scoring 3D volume projection from the parallel models in the MultiFoXS pools. (C) Histograms of R_G values for the best scoring model for each class average.(D) Cumulative distribution of R_G values for the best scoring model for each class average.
(E) Comparison of scores for parallel and parallel CC topologies.

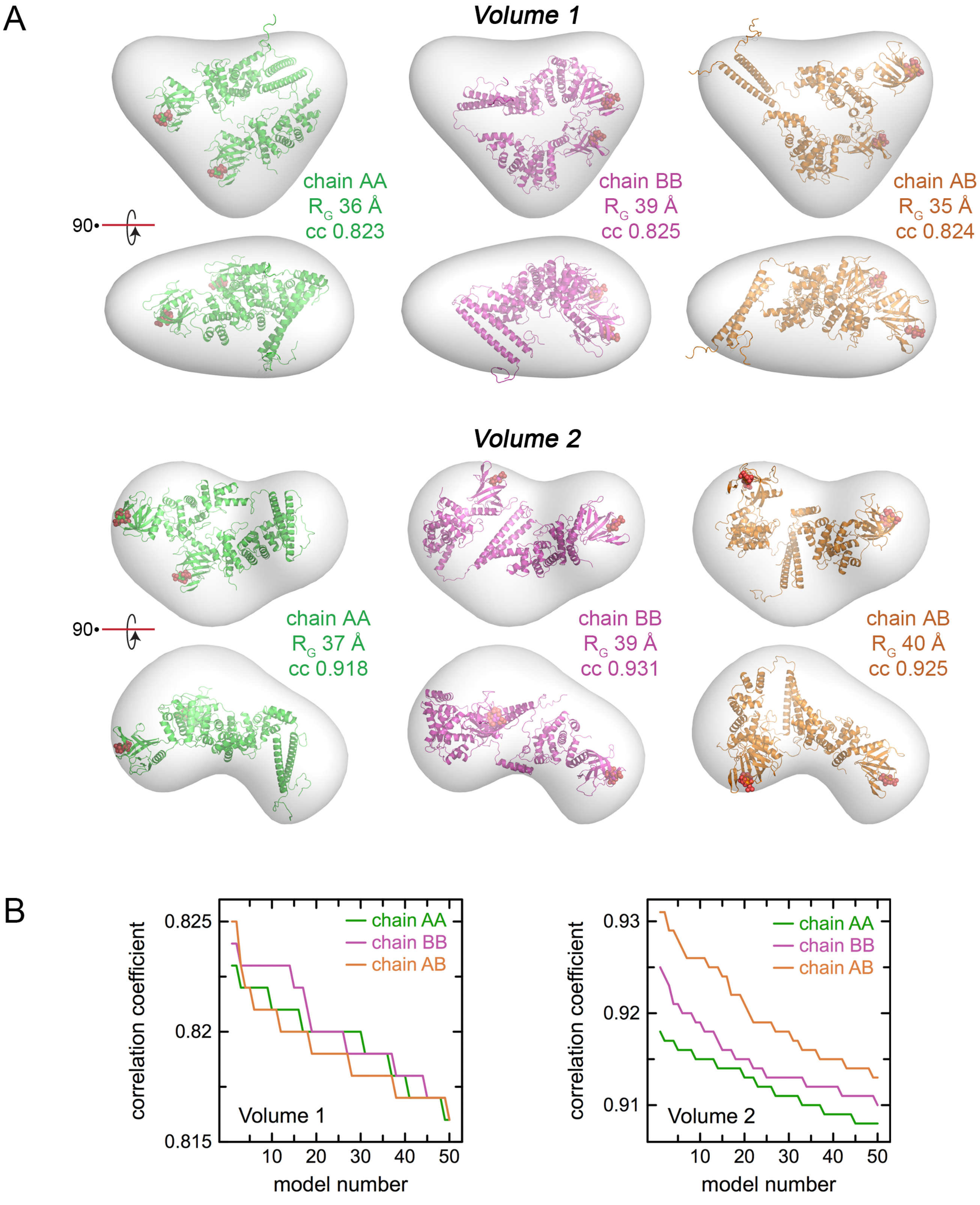

Figure S6. 3D Reconstructions and Best Fitting Parallel MultiFoXS Models. Related to Figure 5
(A) Comparison of the best fitting MultiFoXS models with the volumes from 3D reconstruction and refinement of the class sets indicated in Figure 4C. (B) Correlation coefficients for the 50 best-fitting models from the comparison of each volume with the MultiFoXS pools.

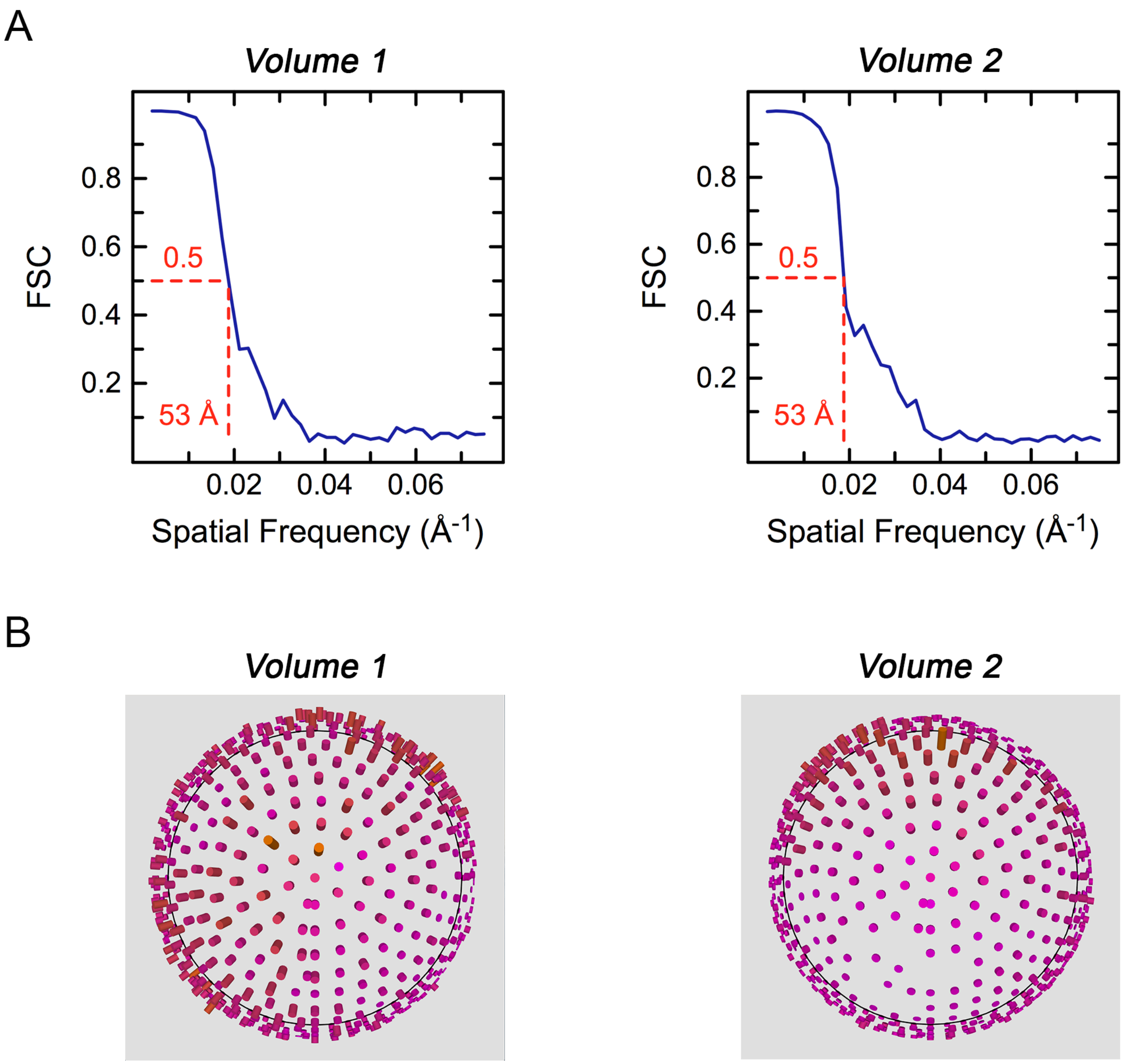

Figure S7. Assessment of 3D Reconstructions. Related to Figure 5
(A) Resolution estimation for 3D reconstructions. (B) Euler angle distribution of particles used to for 3D reconstructions.

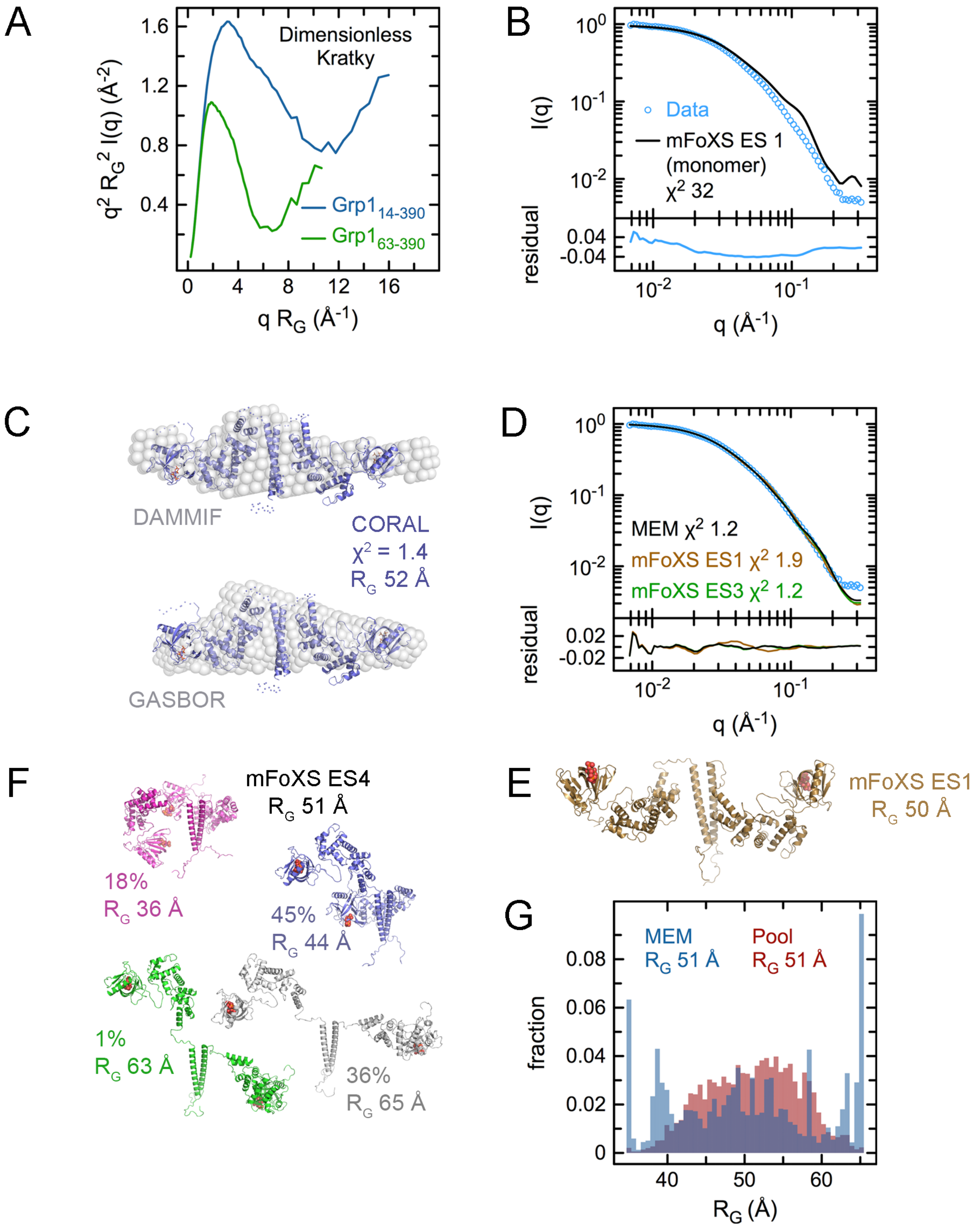

Figure S8. SAXS Analyses of Fully Active Grp1$_{13\text{-}390}$ Dimers. Related to Figure 6
(A) Dimensionless Kratky plot for autoinhibited Grp1 constructs with and without the CC. (B) Comparison of the experimental SAXS profile with the best-fitting single model MultiFoXS profile for a monomer pool. No multistate ensembles were identified. (C) *Ab initio* models calculated with DAMMIF or GASBOR and aligned with the rigid body CORAL model for the parallel CC dimer. (D) Comparison of the experimental SAXS profile with the profiles for the best-fitting single model (ES1) and multiple model (ES4) MultiFoXS ensembles as well as the all model MEM distribution for the parallel CC dimer. (E) Best-fitting single state MultiFoXS model (ES1) for the parallel CC dimer. (F) Models for the best-fitting MultiFoXS ensemble (ES4) for the parallel CC dimer with percentages and R_G values. The overall R_G for the ensemble was calculated as the fraction-weighted mean of the individuals R_G values. (G) Fraction-weighted histograms of R_G values for the MEM distribution and pool for the parallel CC dimer. Fraction-weighted mean R_G values and percent fractions are tabulated below.

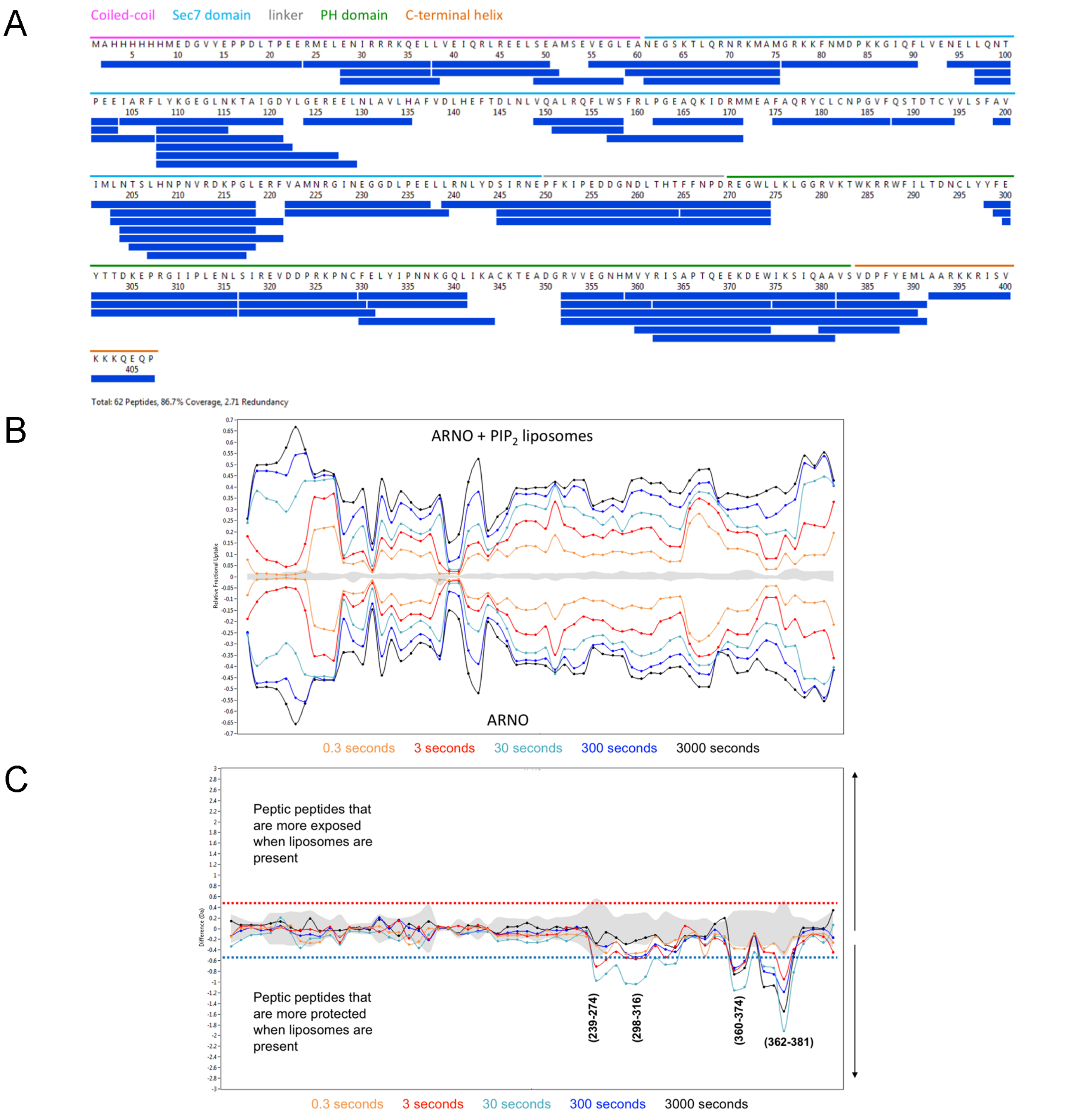

Figure S9. HDX-MS analysis of the $ARNO_{FL}$ interaction with PIP_2 containing liposomes. Related to Figure 8

(A) Peptic peptide coverage of $ARNO_{FL}$ with domains indicated above the amino acid sequence. (B) Butterfly or mirror plot displayed in DynamX 3.0 (Waters, UK) showing the incorporation of deuterium at successive time points indicated by the different colors. Relative fractional uptake is shown on the y-axis,without correction for back-exchange, while the experimental error is reported as a grey bar on the x-axis. (C) Difference plot obtained by subtracting the incorporation of deuterium in the absence of PIP_2 containing liposomes to the one obtained in the presence of PIP_2 containing liposomes. Experimental error is reported in grey, while different colors represent different D_2O incubation times as above. More exposed and protected regions are located, respectively, on the higher and lower part of the graph. Peptide aa numbers from highly protected regions of $ARNO_{FL}$ are shown. ±0.5 Dalton difference is indicated with red/blue dotted lines, which represent a 98% confidence limit, so that peptides above or below these lines can be considered significantly changed.

Table S1. Statistics of SEC-SAXS analysis of ARNOΔ^{Nt} and ARNO^{FL}. Related to Figure 1

		**ARNOΔ^{Nt}**		**ARNO^{FL}**	
Instrument		SOLEIL synchrotron SWING beamline		ESRF BM29	
Detector		PCCD170170 Aviex Detector		Pilatus 1M detector	
Beam geometry		Pinhole		Pinhole	
Wavelength (Å)		1.033		0.99	
q range (Å^{-1})		0.01–0.600		0.0025-0.5	
Exposure time (s)		1.5 per frame		1	
Temperature (°C)		15		15	
**Structural Parameters**					
From Guinier fit	I_0 (cm^{-1})	0.03±2e-05		43.99±6.7e-02	
	R_g (Å)	27.46±0.16		47.9	
From $P_{(r)}$	I_0 (cm^{-1})	0.03±1.4e-05		45.01	
	Volume(Å^3)	63000		14614	
	R_g (Å)	27.7		50.9	
	D_{max} (Å)	98		200	
**Molar Mass Determination**					
Molar mass (kDa) from sequence		39.9		93.2 (homodimer)	
Molar mass (kDa) from Qp		42		102	
Molecular mass (kDa) from MoW		40		89	
Molecular mass (kDa) from Vc		41		89	
**Model Evaluation**					
		Average Fit	Spatial Discrepancy	Average Fit	Spatial Discrepancy
GASBOR (n=5)		Chi^2=2.42±0.18	NSD=1.046±0.045	Chi^2=1.3±0.19	NSD=1.72±0.04
DAMMIN (n=5)		Chi^2=1.72±0.006	NSD=0.631±0.027	Chi^2=1.14±0.05	NSD=0.84±0.05

Table S2. Statistics of SEC-SAXS analysis of Grp1 and ARNO constructs with IP_4. Related to Figures 2 and 6

		**Grp1$_{14\text{-}399}$**	**Grp1$_{14\text{-}390}$**	**ARNO$_{2\text{-}400}$**
Instrument		APS BioCAT 18-ID	APS BioCAT 18-ID	APS BioCAT 18-ID
Detector		MAR 165 CCD	MAR 165 CCD	MAR 165 CCD
Beam geometry		Pinhole	Pinhole	Pinhole
Wavelength (Å)		1.033	1.033	1.033
q range (Å^{-1})		0.0062–0.333	0.0062-0.333	0.0062-0.333
Exposure time (s)		1 per frame	1 per frame	1 per frame
Temperature (°C)		20	20	20
**Structural Parameters**				
From Guinier fit	I_0 (cm^{-1})	1.05±1e-3	1.02±2e-3	1.03±2e-3
	R_g (Å)	54.56±0.17	50.61±0.4	53.03±0.36
From $P_{(r)}$	I_0 (cm^{-1})	1.06±2e-3	1.03±5e-3	1.04±4e-3
	Volume(Å^3)	194000	168000	180000
	R_g (Å)	57.32±0.6	54.27±1.4	55.98±1.3
	D_{max} (Å)	260	257	270
**Molar Mass Determination**				
Molar mass (kDa) from sequence		93.6 (homodimer)	91.2 (homodimer)	95.2 (homodimer)
Molar mass (kDa) from Qp		135	118	127
Molecular mass (kDa) from MoW		97	86	95
Molecular mass (kDa) from Vc		86	76	82
Molecular mass (kDa) from Bayes Inference		94	86	91

**Model Evaluation**						
	Average χ^2	Normalized Spatial Discrepancy	Average χ^2	Normalized Spatial Discrepancy	Average χ^2	Normalized Spatial Discrepancy
GASBOR (n=100)	4.68±0.13	0.604±0.015	1.96±0.033	0.631±0.025	2.18±1.20	0.606±0.022
DAMMIF (n=100)	1.22±0.003	0.570±0.011	0.591±0.015	0.84±0.05	1.19±0.04	0.609±0.017
