# Supplementary material for: Structural Organization and Dynamics of Homodimeric Cytohesin Family Arf GTPase Exchange Factors in Solution and on Membranes
Source: Structure. 2019 Dec 3;27(12):1782–1797.e7. doi: 10.1016/j.str.2019.09.007 (PMC6948192; doi:10.1016/j.str.2019.09.007)
Supplement: Data S1. Software, Related to the STAR Methods [file mmc3.zip › Software/Dela App Scripts/Dela.app/Contents/Resources/Dela.help/Contents/Resources/English.lproj/pgs/axes.html]

xml version="1.0" encoding="utf-8"?


Plot axes panel


Home

Contents


# Plot Axes panel

## Plots axes

Plots typically have two primary axes (X and Y), two secondary axes (X2 and Y2) and, if residuals are displayed, three additional axes (R, R2, and R3). The R and R2 axes refer to the vertical residuals axes located just below the Y and Y2 axes. The R3 axis refers to the horizontal axis above the residuals display area.

### To access the plot panel:

• ***double left-click on an axis*** - opens the Axes paenl to the double left-clicked axis

or

• ***left-click Axes in document tool bar*** - opens Axes panel

or

• ***choose Axes... from the application's Plot menu*** - opens Axes panel

### To select a plot axis:

• ***select an axis from the Plot Axes panel*** - if *All* is selected, changes will be applied to all axes

or

• ***double left-click on an axis***

## Metrics

Basic properties of axes can be controlled from the Metrics view of the Plot Axes panel.

### Axis Range, Scale and Appearance

• ***Auto*** - minimum and maximum values for the axis automatically determined from the range of the data in the plot

• ***Fixed*** - the range for the axis is set to the values supplied in the minimum and maximum fields

• ***Display Axis*** - displays/hides the axis

• ***Log Scale*** - toggles between linear and log scales

• ***Color Well*** - sets the axis color

### Tic Values and Appearance

• ***Display menu*** - selects tic display options (none, inside, outside or both)

• ***Color Well*** - sets the color for major and minor tics

• ***First Value*** - sets the initial tic value automatically (auto) or to the *real value* supplied in the field (fixed)

• ***Increment*** - sets the tic increment automatically (auto) or to the *real value* supplied in the field (fixed)

• ***Exponent*** - sets the exponent automatically (auto) or to the *integer value* supplied in the field (fixed)

• ***Major tic length*** - the *real value* for the length of the major tics in pixels

• ***Minor tic length*** - the *real value* for the length of the minor tic in pixels

• ***# minor tics*** - an integer value that specifies the number of minor tics

## Stroke

The drawing (stroking) of an axis can be controlled from the Stroke view of the Plot Axes panel.

### Axis / Major Tics / Minor Tics

• ***Type*** - determines the display type (none, continuous, or dashed)

• ***Join*** - determines how consecutive line segments are joined (miter, round, or bevel).

\* ***Cap*** - determines the shape at the end of a line segment (butt, square, or round)

• ***Color Well*** - sets the color

• ***Width*** - the *real value* of the width in pixels

• ***Miter Limit*** - the *real value* of the miter limit in pixels (limit for the length of the miter joint between line segments)

• ***Opacity*** - the *real value* of the opacity (i.e., the alpha component) ranging from 0 (100% transparent) to 1 (0% transparent)

## Shadow

The display and appearance of semi-transparent 'shadows' can be controlled from the Shadow view of the Plot Axes panel.

### Axis / Major Tics / Minor Tics

• Type - determines whether shadows are displayed

• Blur - the *real value* for the blurring factor

• Color Well - sets the shadow color

• Offset X - the *real value* of the X offset from the object casting the shadow

• Offset Y - the *real value* of the Y offset from the object casting the shadow

• Opacity - the *real value* of the opacity (i.e., the alpha component) ranging from 0 (100% transparent) to 1 (0% transparent)
