# Supplementary material for: Structural Organization and Dynamics of Homodimeric Cytohesin Family Arf GTPase Exchange Factors in Solution and on Membranes
Source: Structure. 2019 Dec 3;27(12):1782–1797.e7. doi: 10.1016/j.str.2019.09.007 (PMC6948192; doi:10.1016/j.str.2019.09.007)
Supplement: Data S1. Software, Related to the STAR Methods [file mmc3.zip › Software/Dela App Scripts/Dela.app/Contents/Resources/Dela.help/Contents/Resources/English.lproj/pgs/binding_kinetics.html]

xml version="1.0" encoding="utf-8"?


Binding Kinetics


Home

Contents


# Binding Kinetics

## Model function

Y (X; ***P***) = ***P4*** + ***P5***\*X + ***conditional term***

where

### if X < ***P0*** (pre-association phase)

***conditional term*** = 0;

### else if X < ***P1*** (association phase)

***conditional term*** = ***P2*** + ***P6*** \* fbeq \* (1 - exp(-kobs \* (X - ***P0***)))

### else (dissociation phase)

***conditional term*** = P6 \* fbeq \* (1 - exp(-kobs \* (***P1*** - ***P0***))) \* exp(-***P8*** \* (X - ***P1***))

fbeq = ***P3*** / (Kd + ***P3***)

kobs = ***P7*** \* ***P3*** + ***P8***

Kd = ***P8*** / ***P7***

## Parameters

### Non-term parameters

***P0*** - association phase start time

***P1*** - dissociation phase start time

***P2*** - association phase bulk signal

***P3*** - total concentration of the variable component

***P4*** - constant

***P5*** - slope

***P6*** - amplitude

***P7*** - association rate constant (ka)

***P8*** - dissociation rate constant (kd)

### Term parameters

***None***

For more general information about models, see Model Functions.
