# Supplementary material for: Structural Organization and Dynamics of Homodimeric Cytohesin Family Arf GTPase Exchange Factors in Solution and on Membranes
Source: Structure. 2019 Dec 3;27(12):1782–1797.e7. doi: 10.1016/j.str.2019.09.007 (PMC6948192; doi:10.1016/j.str.2019.09.007)
Supplement: Data S1. Software, Related to the STAR Methods [file mmc3.zip › Software/Dela App Scripts/Dela.app/Contents/Resources/Dela.help/Contents/Resources/English.lproj/pgs/catalytic_efficiencies.html]

xml version="1.0" encoding="utf-8"?


Catalytic Efficiencies sheet


Home

Contents


# Catalytic Efficiencies (kcat/Km) sheet

## Calculating catalytic efficiencies

The Catalytic Efficiencies (kcat/Km) sheet facillitates calculation of the catalytic efficiency of an enzyme from a plot of the observed rate (kobs or initial rate) vs. the concentration of subtrate or enzyme, depending on which was varied in the experiment.

## Data source

Catalytic efficiencies can be calculated for any number of data objects.

### To specify data objects for calculation of initial rates:

1. Highlight or select data for processing in the Document Contents, Data List or Sheet Data List

2. Choose the desired data source using the ***Data from*** the popip menu

For more general information about models, see Model Functions.
