# Supplementary material for: Structural Organization and Dynamics of Homodimeric Cytohesin Family Arf GTPase Exchange Factors in Solution and on Membranes
Source: Structure. 2019 Dec 3;27(12):1782–1797.e7. doi: 10.1016/j.str.2019.09.007 (PMC6948192; doi:10.1016/j.str.2019.09.007)
Supplement: Data S1. Software, Related to the STAR Methods [file mmc3.zip › Software/Dela App Scripts/Dela.app/Contents/Resources/Dela.help/Contents/Resources/English.lproj/pgs/contents.html]

xml version="1.0" encoding="utf-8"?


Contents


Home

Contents


# Contents

## General

Overview

Document window

## Import and Export

Importing data

Exporting to text files

Exporting plots as images

## Graphics

Creating and updating plots

Working with plots

Plot Axes panel

Plot Symbols panel

## Data Processing

Process panel

Interpret script panel

Interpreter

Scripts

Python scripts

SAXS Python scripts

ITC panel

SVD panel

## Enzyme Kinetics

Initials Rates sheet

Catalytic Efficiencies sheet

## Models

Model Selection

Model functions

Fit Options

## Maximum Likelihood

Viewing, editing and fitting parameters

Set Parameters panel

## Creating and Setting New Data

New Data

New Instrument Response

New Model Data

## Statistics and Error Estimation

Statistics

Estimate Errors
