# Supplementary material for: Structural Organization and Dynamics of Homodimeric Cytohesin Family Arf GTPase Exchange Factors in Solution and on Membranes
Source: Structure. 2019 Dec 3;27(12):1782–1797.e7. doi: 10.1016/j.str.2019.09.007 (PMC6948192; doi:10.1016/j.str.2019.09.007)
Supplement: Data S1. Software, Related to the STAR Methods [file mmc3.zip › Software/Dela App Scripts/Dela.app/Contents/Resources/Dela.help/Contents/Resources/English.lproj/pgs/data_object.html]

xml version="1.0" encoding="utf-8"?


Data Object


Home

Contents


# Data Object

## Model function

Y (X; ***P***) = ***P0*** + ***P1***\*X + ***P2***\*Y\_DataI2(X) [ + ... + ***Pn*** \* Y\_DataIn(X) ]

## Parameters

### Non-term parameters

***P0*** - constant

***P1*** - slope

### Term parameters

***P2 ... Pn-1*** - amplitude

***DataI2 ... DataIn*** - data objects in model data list. There should be at least as many model data objects as term parameters. Excess model data objects are ignored. Terms without model data objects are treated as data with Y values equal to zero.

This function uses general linear least squares for estimation the constant, slope, and amplitude parameters.

For more general information about models, see Model Functions.
