# Supplementary material for: Structural Organization and Dynamics of Homodimeric Cytohesin Family Arf GTPase Exchange Factors in Solution and on Membranes
Source: Structure. 2019 Dec 3;27(12):1782–1797.e7. doi: 10.1016/j.str.2019.09.007 (PMC6948192; doi:10.1016/j.str.2019.09.007)
Supplement: Data S1. Software, Related to the STAR Methods [file mmc3.zip › Software/Dela App Scripts/Dela.app/Contents/Resources/Dela.help/Contents/Resources/English.lproj/pgs/document_window.html]

xml version="1.0" encoding="utf-8"?


Document window


Home

Contents


# Document window

## Layout

The main document window includes a contents view for selection of data, sheets, and plots. Items selected in the contents view become the default target(s) for actions including processing and plotting. The most recently selected item is displayed in the main view. Click on disclosure triangles in the contents view to toggle item visibility. In some cases, it may be necessary to click on a view to activate it for keyboard input.

## Views

### Contents view

The contents view is hierarchically organized with four parent items: All Data, Sheets, Plots and Groups.

• ***All Data*** - list of all data objects in the document. Data are stored in objects with arrays for x, y, and error values. Every data object has a unique identifying ‘Label’ of the form Data#, where # is an integer automatically assigned when the data objected is created.

• ***Sheets*** - list of all data sheets in the document. Unlike typical spread sheets, however, data objects may be shared between different sheets. Data objects can be visualized/edited in tabular from, either individually or collectively at the level of a sheet.

• ***Plots*** - list of all plots in the document.

• ***Groups*** - list of all groups in the document. Data, plots, and sheets can be organized into groups of related content.

### Main view

Content for items selected in the contents view is displayed in the main view.

### Utility view

Various utilities for data procressing, scripts, plotting, fitting, etc.

### Tool bar

Buttons and menus for accesing program functionallity.

### Format bar

Various formatting controls that depend on the type of content displayed in the main view.

### Info strip

Information related to the content displayed in the main view.
