# Supplementary material for: Structural Organization and Dynamics of Homodimeric Cytohesin Family Arf GTPase Exchange Factors in Solution and on Membranes
Source: Structure. 2019 Dec 3;27(12):1782–1797.e7. doi: 10.1016/j.str.2019.09.007 (PMC6948192; doi:10.1016/j.str.2019.09.007)
Supplement: Data S1. Software, Related to the STAR Methods [file mmc3.zip › Software/Dela App Scripts/Dela.app/Contents/Resources/Dela.help/Contents/Resources/English.lproj/pgs/estimate_errors.html]

xml version="1.0" encoding="utf-8"?


Estimate errors


Home

Contents


# Estimate errors

## Estimating errors

For every X Y pair in a data object there is an associated Y error that is either imported with the data or otherwise automatically estimated when the data are imported/created. The default algorithm estimates a single uniform error value for all Y values by averaged the root mean squared deviation of a series of linear fits for a sliding window with a width representing a small fraction of the data. The width of the sliding window is at least three points but typically larger. The default error estimation may work reasonably well as a crude approximation. A variety of other options described below are also available and may provide better estimates. None of these estimates should be taken literally as there is error in the estimation of the errors. Nevertheless, the best strategy for error estimation depends on the details of the particular data set(s), including prior processing and/or modelling.

### To estimate errors for data object(s):

1. In the document contents, select the data objects, sheets, and/or plots with the data for error estimation.

2. Click ***Statistics→Estimate Errors***  from the document toolbar.

3. Change the data source if necessary, modify the options as desired, and click  ***Estimate Errors***.

## Options

The following options are presented when ***Statistics→Estimate Errors*** is selected from the document toolbar.

### Data from

Selects the intended data source.

• ***Estimate using residuals check box*** - if checked and one or more models are available for the data, errors will be estimated using the residuals for currently selected model.

### X range

Specifies the X value range for error estimation.

• ***Entire range*** - extends over all data points.

• ***Obtain from plot selection regions*** - extends over data points within the X range(s) of one or more plot selection regions.

• ***Enter minimum and maximum*** - extends over the range defined by the values in the minimum X and maximum X fields.

### Error Estimation Options

Specifies the options for error estimation.

• ***RMSD*** - estimate errors as the root mean squared deviation.

• ***Running RMSD***  - estimate errors as the RMSD from a series of linear fits with a sliding window. The size in points of the sliding window is automatically determined if Auto window size is checked or otherwise taken from the adjacent field.

• ***Value*** - errors are set to the value in the adjacent field.

• ***Polynomial*** - errors are estimated as the root mean squared deviation from a polynomial fit with a sliding window. The size in points of the sliding window is taken from the adjacent field. The order of the polynomial (i.e. the highest exponent in the polynomial series) is taken from the order field.

• ***Non-uniform*** - if checked, non-uniform errors are calculated as an average over the estimated errors in a sliding window with a size in points taken from the adjacent field. Otherwise, a uniform error value will be calculated as an average over all points. Applies only to the Running RMSD and Polynomial options.
