# Supplementary material for: Structural Organization and Dynamics of Homodimeric Cytohesin Family Arf GTPase Exchange Factors in Solution and on Membranes
Source: Structure. 2019 Dec 3;27(12):1782–1797.e7. doi: 10.1016/j.str.2019.09.007 (PMC6948192; doi:10.1016/j.str.2019.09.007)
Supplement: Data S1. Software, Related to the STAR Methods [file mmc3.zip › Software/Dela App Scripts/Dela.app/Contents/Resources/Dela.help/Contents/Resources/English.lproj/pgs/exponential_decay.html]

xml version="1.0" encoding="utf-8"?


Exponential Decay


Home

Contents


# Exponential Decay

## Model function

Y (X; ***P***) = ***P0*** + ***P1***\*X + ***P2***\*exp(-***P3***\*X) [ + ... + ***Pn-1*** \* exp(-***Pn***\*X) ]

## Parameters

### Non-term parameters

***P0*** - constant

***P1*** - slope

### Term parameters

***P2 ... Pn-1*** - amplitude

***P3 ... Pn*** - decay constant

For more general information about models, see Model Functions.
