# Supplementary material for: Structural Organization and Dynamics of Homodimeric Cytohesin Family Arf GTPase Exchange Factors in Solution and on Membranes
Source: Structure. 2019 Dec 3;27(12):1782–1797.e7. doi: 10.1016/j.str.2019.09.007 (PMC6948192; doi:10.1016/j.str.2019.09.007)
Supplement: Data S1. Software, Related to the STAR Methods [file mmc3.zip › Software/Dela App Scripts/Dela.app/Contents/Resources/Dela.help/Contents/Resources/English.lproj/pgs/export.html]

xml version="1.0" encoding="utf-8"?


Exporting Data


Home

Contents


# Exporting to text files or pasteboard

## Export Methods

Data, model or residuals can be exported to standard ASCII text files or to the clipboard. Parameters associated with model functions can exported to the pasteboard.

### Exporting by File Navigation

1. Highlight the data objects of interest in the Documents Contents view.

2. Select ***Export*** from the document toolbar or ***Export ASCII...*** from the Export sub-menu of the application's File menu.

3. Select a directory for the exported files and click ***Open***.

4. Specify the target, column format, file name options, labels labels to be included, base name and/or tag as required and click ***Export***.

### Exporting by Pasting from the Pasteboard

1. Highlight the data objects of interest in the Documents Contents view.

2. Select the ***Data List*** or a specific sheet from the ***Document Contents***.

3. Type ***<commmand v>*** or select ***Paste*** from the application's ***Edit*** menu.

4. Specify the column format and labels if any and click ***Import***.

## Target

The target for export can correspond to the data, model, residuals or instrument response from any of the available sources listed in the right hand menu. If the target is model or residuals, the values associated with the current model will be exported. The current model can selected using the Model button in the document toolbar or the Select... item in the application's Model menu. All values will be written to a single file unless ***Export as individual files*** is checked.

## Column Format

• ***X Y*** - first column of numbers is X values; second column of numbers is Y values

• ***X Y Error*** - same as X Y format but includes a third column of numbers for errors

• ***Y*** - first column of numbers is Y values

• ***Y Error*** - same as Y format but includes a second column of numbers for errors

• ***X Y1 Y2*** ... - tab deliminated spread sheet; first column is X values; subsequent columns are Y values

## File Name Options

One of the following options must be selected:

• ***Use existing files names*** - filenames will correspond exactly to the filename shown in the Data List.

• ***Insert tag before extension of existing file names*** - the tag supplied in the Tag field will be inserted before the file extension of existing file names.

• ***Use base name with incremental index*** - filenames will contain the base name supplied in the Base name field, an incremental index and a .txt extension.

• ***Use base name with buffer index*** - filenames will contain the base name supplied in the Base name field, the buffer index and a .txt extension.

• ***Use base name as file name*** - the filename will correspond to the basename supplied in the Base name field

***Any existing files with the same name will be overwritten.***

## Labels

Any combination of the following descriptive labels can be included:

• ***Data label*** - describes individual data (e.g. data from on well of a microplate)

• ***Column label*** - describes the X and Y columns (e.g. time, concentration, intensity, etc)

• ***Row labels*** - describes individual data pairs

• ***Single row label for all columns*** - a single row label derived from the first highlighted data buffer is written at the beginning of each line.

Since labels are optional, the number of label lines can range from none to two. The first label line will contain the Data label if included or the Column label otherwise. The second label line is restricted to column labels. Row labels will preceed the X values (X and Y Error formats) or the Y values (Y, Y Error, and X Y1 Y2 ... formats).

## Files, Data Types and Contents

The exporter writes standard ASCII characters to text files or the paste board. Label and value fields are tab deliminted. Each line is terminated with a carriage return.
