# Supplementary material for: Structural Organization and Dynamics of Homodimeric Cytohesin Family Arf GTPase Exchange Factors in Solution and on Membranes
Source: Structure. 2019 Dec 3;27(12):1782–1797.e7. doi: 10.1016/j.str.2019.09.007 (PMC6948192; doi:10.1016/j.str.2019.09.007)
Supplement: Data S1. Software, Related to the STAR Methods [file mmc3.zip › Software/Dela App Scripts/Dela.app/Contents/Resources/Dela.help/Contents/Resources/English.lproj/pgs/export_image.html]

xml version="1.0" encoding="utf-8"?


Exporting Images


Home

Contents


# Exporting plots as TIFF images

## Method

To save a plot as a TIFF (Tagged Image Format File) image:

1. Highlight the plot of interest in the Document Contents view.

2. Select ***Export Image*** from the document toolbar or ***Export Image...*** from the Export sub-menu application's File menu.

3. Enter a base name for the image file, select the directory, select the image file format, and click ***Save***.

4. Specify the resolution, color depth, and opacity and click ***Export***.

## Resolution

Common image resolutions in dots (i.e. pixels) per inch are listed in the menu. Other resolutions can be specified by selecting Other from the menu and entering the desired value in the Resolution field.

## Color Depth

By default, TIFF images are generated with a color depth of eight bits per channel corresponding to "Millions of Colors". This is currently the only option.

## Opacity

Determines whether the alpha channel is included.

## Trimming

If Trim is checked, the image is trimmed to removed background pixels in the plot margins.

## Color Channels

TIFF images are generated with color channels (red, blue and green or RGB) and an optional alpha channel per pixel in the order RGB[A] RGB[A] ... Color components are not pre-multiplied by the alpha value.
