# Supplementary material for: Structural Organization and Dynamics of Homodimeric Cytohesin Family Arf GTPase Exchange Factors in Solution and on Membranes
Source: Structure. 2019 Dec 3;27(12):1782–1797.e7. doi: 10.1016/j.str.2019.09.007 (PMC6948192; doi:10.1016/j.str.2019.09.007)
Supplement: Data S1. Software, Related to the STAR Methods [file mmc3.zip › Software/Dela App Scripts/Dela.app/Contents/Resources/Dela.help/Contents/Resources/English.lproj/pgs/fit_options.html]

xml version="1.0" encoding="utf-8"?


Fit Options


Home

Contents


# Fit Options

## Fit Options

Control parameters for fitting algorithms can be changed in Fit Options panel.

### To access the Fit Options panel:

• ***left-click Fit Options in document tool bar***

or

• ***choose Fit Options... from the application's Model menu***

## Marquardt Control Options

### Iteration Limit

The maximum number of cycles for the Marquardt algorithm.

### Iterations after convergence

The number of cycles that must be completed in which the changes in the merit function value between successive cycles are less than the fractional tolerance specified for convergence.

## Simplex Control Options

### Iteration Limit

The maximum number of cycles for the Simplex algorithm.

### Iterations after convergence

The number of cycles that must be completed in which the changes in the merit function value between successive cycles are less than the fractional tolerance specified for convergence.

### Intial fractional variation

The fraction of the original parameter value that will be added or substracted to generate the intial verticies of the Simplex object.

### Maximum Simplex iterations

The maximum number of Simplex interations per cycle.

## Convergence Criteria

### Fractional tolerance

The fractional difference in the merit function value between cycles required to establish convergence.
