# Supplementary material for: Structural Organization and Dynamics of Homodimeric Cytohesin Family Arf GTPase Exchange Factors in Solution and on Membranes
Source: Structure. 2019 Dec 3;27(12):1782–1797.e7. doi: 10.1016/j.str.2019.09.007 (PMC6948192; doi:10.1016/j.str.2019.09.007)
Supplement: Data S1. Software, Related to the STAR Methods [file mmc3.zip › Software/Dela App Scripts/Dela.app/Contents/Resources/Dela.help/Contents/Resources/English.lproj/pgs/higer_order_gaussian.html]

xml version="1.0" encoding="utf-8"?


Higher Order Gaussian


Home

Contents


# Higher Order Gaussian

## Model function

Y (X; ***P***) = ***P0*** + ***P1***\*X + ***P2*** / (sqrt(2π)\****P4***) \* exp(-(X - ***P3***)^2 / (2\****P4***^2) - P5 \* (X - ***P3***)^3 - P6 \* (X - ***P3***)^4) [ + ... + ***Pn-4*** \* exp(-(X - ***Pn******-3***)^2 / (2\****Pn******-2***^2) - ***Pn-1***\*(X - ***Pn******-3***)^3 + ***Pn*** \* (X - ***Pn******-3***)^4) ]

## Parameters

### Non-term parameters

***P0*** - constant

***P1*** - slope

### Term parameters

***P2 ... Pn-4*** - amplitude

***P3 ... Pn-3*** - mean

***P4 ... Pn-2*** - standard deviation

***P5 ... Pn-1*** - B (weight for cubic term)

***P6 ... Pn*** - C (weight for quartic term)

For more general information about models, see Model Functions.
