# Supplementary material for: Structural Organization and Dynamics of Homodimeric Cytohesin Family Arf GTPase Exchange Factors in Solution and on Membranes
Source: Structure. 2019 Dec 3;27(12):1782–1797.e7. doi: 10.1016/j.str.2019.09.007 (PMC6948192; doi:10.1016/j.str.2019.09.007)
Supplement: Data S1. Software, Related to the STAR Methods [file mmc3.zip › Software/Dela App Scripts/Dela.app/Contents/Resources/Dela.help/Contents/Resources/English.lproj/pgs/import.html]

xml version="1.0" encoding="UTF-8"?


Importing Data


Home

Contents


# Importing data

## Import Methods

Dela provides several equivalent methods for importing data from files or from the pasteboard.

### Importing by File Navigation

1. Select ***Import*** from the document toolbar or from the application's File menu.

2. Select one or more files for import and click ***Open***.

3. Specify the column format and labels if any and click ***Import***.

### Importing by File Drag and Drop

1. Select the ***Data List*** or a specific sheet from the ***Document Contents***.

2. Drag one or more files from the Finder onto the right hand view area.

3. Specify the column format and labels if any and click ***Import***.

### Importing from the Pasteboard

1. Select the ***Data List*** or a specific sheet from the ***Document Contents***.

2. Type ***<commmand v>*** or select ***Paste*** from the application's ***Edit*** menu.

3. Specify the column format and labels if any and click ***Import***.

## Data Type

Selecting a Data Type from the popup menu sets default import options. It is not necessary to select a data type.

## Header

Header information is optional and may include any of the following descriptive labels:

• ***data label***s - a single line with tab delimited descriptions of the individual data sets

• ***column labels*** - a single line with tab delimited descriptions of the X, Y and error columns (e.g. time, concentration, intensity, etc)

• ***row labels*** - tab delimited description of individual rows

Data, column and row labels can be imported or ignored. By default, head and trailer lines with any text content in addition to numeric data and delimiters will be ignored. Lines with row labels will not be imported unless *Has row labels* is checked.

Any number of lines preceding optional data and/or column labels can be skipped.

## Multiple files

When importing multiple files at the same time, the files can be imported into a single sheet for all files or one sheet for each file.

## Missing X values

For column formats without X values, the missing X value are reconstructed using the values in the *Initial value* and *Increment* fields.

## Columns

The importer parsers lines for numeric values in free format columns seperated by one or more delimiters. The following collumn formats are available:

• ***X ...***  - columns of X values

• ***Y*** ... - columns of Y values

• ***Error*** ... - columns of errors

• ***X Y*** ... - columns of X values followed by Y values

• ***X Error*** ...- columns of X values followed by errors

• ***Y Error*** ...- columns of Y values followed by errors

• ***X Y Error*** ... - columns of X values followed by Y values followed by errors

• ***X Y1 Y2 ...*** - single column of X values followed by columns of Y values

• ***ITC*** - ITC data from Microcal text files.

For the Y ... and Y Error ... formats, the X values are reconstructed from initial value and increment under Missing X Values.

The number of columns is automatically determined from the most frequently occuring number of numeric values in all lines of the input source.

Lines are parsed for numeric values, which are interpreted according to the specified column format. If the number columns exceeds the the number of column format specifiers (e.g. X Y and X Y E have two and three format specifiers, respectively), the columns are interpreted as a sequence of data sets with a shared column format (e.g. X1 Y1 X2 Y2 ... or X1 Y1 E1 X2 Y2 E2 ..., where the numbers indicate the data set).

## Default descriptions

If not included in the file or pasteboard content and Ignore is not selected, data labels and column labels are taken from the designated text boxes.

Since labels are optional, the number of label lines can range from none to two. The first label line can contain Data or Column labels. The second label line is restricted to column labels. Row labels must preceed the X values (X and Y Error formats) or the Y values (Y, Y Error, and X Y1 Y2 ... formats).

Sheet and data descriptions can be acquired from file names.

## Delimiters

By default, files or pasteboard content are assumed to be delimited by a Tab, Space or Comma. More or less restrictive sets of allowed delimiters can be selection from the popup menu. Custom delimiter sets can be defined by selecting *Characters from text on right* from the popup menu and provided one or more unicode characters in the field on the right.

Extra spaces before or after delimiters are ignored. A delimiter after the last numeric value in a line is not required and ignored if present. Delimiters not preceded by numeric characters and spaces are interpreted as missing values that default to zero for X and Y values and one for errors. Data, column and row labels are assumed to be tab delimited.

## Text types and contents

The importer recognizes ASCII or Unicode characters from text files or pasteboard contents as a byte stream of ***UTF-8 encoded unicode characters***, which includes the basic Latin, decimal, punctuation, and control characters encoded by the ASCII character set. Files or pasteboard data can contain optional label lines, followed by any number of data lines (in practice limited by available memory). Labels should be tab deliminted. Deliminators separating data columns are tab, space(s), or comma by default. Lines are definded by termination with newline (U+000A, \n or LF), return (U+000D, \r or CR), both (U+000DU+000A,\r\n or CRLF) or unicode line (U+2028) or paragraph (U+2029) separators. Lines containing non-numeric characters that are not either delimiters or terminators are ignored.
