# Supplementary material for: Structural Organization and Dynamics of Homodimeric Cytohesin Family Arf GTPase Exchange Factors in Solution and on Membranes
Source: Structure. 2019 Dec 3;27(12):1782–1797.e7. doi: 10.1016/j.str.2019.09.007 (PMC6948192; doi:10.1016/j.str.2019.09.007)
Supplement: Data S1. Software, Related to the STAR Methods [file mmc3.zip › Software/Dela App Scripts/Dela.app/Contents/Resources/Dela.help/Contents/Resources/English.lproj/pgs/initial_velocities.html]

xml version="1.0" encoding="utf-8"?


Initial Rate sheet


Home

Contents


# Initial Rate sheet

## Calculating initial rates

The Initial Rate sheet facillitates calculation of intial rates for enzyme kinetic data. Before calutating initial rates, the data should be masked to include only data points within the initial linear region.

## Data source

Initial rates can be calculated for any number of data objects.

### To specify data objects for calculation of initial rates:

1. Highlight or select data for processing in the Document Contents, Data List or Sheet Data List

2. Choose the desired data source using the ***Data from*** the popip menu

## Concentrations

The concentrations of the variable component (substrate or enzyme) should be included in the table provide. Use the + and - buttons to add or delete concentrations as needed. The number and order of concentrations should match the number and order of data objects for which initial rates will be calculated.

## Conversion factor

The y values of each data object will be multiplied by the conversion factor provided in the ***Multiply y values*** by field. The conversion factor allows conversion from observation units (e.g. fluorescence, absorbance, counts, ...) to concentration units (e.g. M, mM, ...).
