# Supplementary material for: Structural Organization and Dynamics of Homodimeric Cytohesin Family Arf GTPase Exchange Factors in Solution and on Membranes
Source: Structure. 2019 Dec 3;27(12):1782–1797.e7. doi: 10.1016/j.str.2019.09.007 (PMC6948192; doi:10.1016/j.str.2019.09.007)
Supplement: Data S1. Software, Related to the STAR Methods [file mmc3.zip › Software/Dela App Scripts/Dela.app/Contents/Resources/Dela.help/Contents/Resources/English.lproj/pgs/interpret_script.html]

xml version="1.0" encoding="utf-8"?


Interpreter


Home

Contents


# Interpret script

## Script interpretation

The Interpret script panel provides an interface for interpretation of scripts contained in plain text files containing 8 bit characters extended from ASCII via UTF-8 encoding. Dela scripts have file names ending in a .dela extension. Scripts can be selected from a menu of imported scripts or loaded from a file path. See Interpreter for descriptions of the syntax and available internal functions.

## Script

### Script menu

Allows selection of imported scripts or an option to get scripts from the file path in the Script file field.

### Import button

Iniates an open file navigation dialog to select script files, which are copied to the pre-defined script directory in the users path. Truncated verions of the file names lacking extensions are added to the script menu.

### Delete button

Deletes the currently selected script with the name shown in the script menu. Only the imported copy of the original script is deleted.

## Script file

Specifies an optional path to a plain text script file, which will be used if "Get from script file path" is displayed in the Script menu.

### Script file field

Absolute path to a script file.

### Browse button

Iniates an open file navigation dialog to select a script file.

## Log file

Specifies an optional path for a rich text file for script generated log information. If no log file is specified, the log information can be saved after script interpretation via the Save Log button.

### Log file field

Absolute path path for file.

### Browse button

Iniates an save file navigation dialog to select a path and name for the log file.

## Log

Script generated log information, which can be optionally edited and/or saved. Error messages, if any, are highlighted in red. The interpreter attempts to identify the type of error and trace the location in the script at which the error occured. However, the error may indirectly result from an eariler problem that was not detected.

### Log text view

Text view updated with script generated log information.

### Save Log button

Iniates an save file navigation dialog to select a path and name for the log file.

## Interpret button

Loads and interprets the script selected in the Script menu or specified in the Script file field. For scripts acting on document objects, selection of items in the document interface may be required prior to script execution.
