# Supplementary material for: Structural Organization and Dynamics of Homodimeric Cytohesin Family Arf GTPase Exchange Factors in Solution and on Membranes
Source: Structure. 2019 Dec 3;27(12):1782–1797.e7. doi: 10.1016/j.str.2019.09.007 (PMC6948192; doi:10.1016/j.str.2019.09.007)
Supplement: Data S1. Software, Related to the STAR Methods [file mmc3.zip › Software/Dela App Scripts/Dela.app/Contents/Resources/Dela.help/Contents/Resources/English.lproj/pgs/interpreter.html]

xml version="1.0" encoding="utf-8"?


Interpreter


Home

Contents


# Interpreter

## The Interpreter

Dela uses an interpreter to preform data transformations and other calculations that require evaluation of mathematical expressions. The interpreter is in essence a recursive descent parser that interprets programs with a C-like syntax described briefly below. When a new Dela document is created or opened, an interpreter for that document is invoked with a defult program that creates various internal variables and then awaits subsequent instructions. What is actually passed to the interpreter is a block of instructions that can range from a single regular expression to a complex algorithm involving multiple expressions, logic elements and loops. The terminating semi-colon is optional for a single regular expression but required for multiple expression blocks.

Scripts consisting of complete programs stored in plain text files can also be interpreted using the Interpret script panel.

## Syntax

### Character and string constants

Charater constants are enclosed by apostrophes ('...') and contain a single ASCII character. String constants are enclosed by quotes ("...") and contain C-like strings extended to 8 bits via UTF-8 encoding. Recognized characteres include the following:

**0-9**

**A-Z**

**a-z**

**\b**, back

**\f**, forward

**\n**, newline

**\r**, return

**\t**, tab

**"**, double quote

**\'**, apostrophe

**\\**, backslash

**\v**,

**\a**,

**\?**,

### Integer and floating point constants

**Integer consants** have the form [+ or -]d, where d is any sequence of digits (0-9)

**Floating point constants** have the form [+ or -][d].[d][e or E][+ or -][d], where d is any sequence of digits (0-9) and e or E are the standared base 10 exponentiation symbols

### Variables, variable types, and arrays

All ***variables*** are by definition arrays, which contain one or more elements of the following types:

Characters specified by the ketyword ***char***

Integers (32 bit for OSX 10.6.x, 64 bit otherwise) defined by the keyword ***int***

Double precission floating point numbers (64 bit) defined by the keyword ***double***

### Operators

***\*,*** multiplication

***/***, division

***+***, addition or unary plus or string concatenation

***-***, subtraction or unary minus

^, power operator (raises the left side value to the power of the right side value)

***=***, assigns the value of the right hands side to the left hand side

!, logical NOT (0 becomes 1, other values become 0)

### Logical operators

***==***, equal to

***!=***, not equal to

***>***, greater than

***<***, less than

***>=***, greater than or equal to

***<=***, less than or equal to

***&&***, logical AND

***||***, logical OR

### Delimiters

***{}***, braces enclose a block of code

***()***, paraentheses group operations in regular expressions or enclose an argument list for functions

***[]***, square brackets enclose an index or expression to calculate an index of an array element

***;***, semi-colons terminate terminate expression

***" "***, double quotes enclosing a C string

***' '***, apostrophes (single quotes) enclosing characters

**,** , comma separates elements of a list.

### Character and string constants

**digits**

**A-Z**

**a-z**

**\b**, back

**\f**, forward

**\n**, newline

**\r**, return

**\t**, tab

**"**, double quote

**\'**, apostrophe

**\\**, backslash

**\v**,

**\a**,

**\?**,

"...", double quotes enclose C-like strings (i.e., C style strings extended to 8 bits via UTF-8 encoding)

### Keywords

***char***, declares a local C character

***int***, declares an local integer

***double***, declares a local double precession floating point number

***gchar***, declares a global C charater

***gint***, declares a global integer

***gdouble***, declares a global double precession floating point number

***release***, removes a previously decleared variable from the local or global stack and free associated memory

***return***, returns control from a function

***if***, iniates evaluation of a conditional expressoin

***else***, executed when evalution of an if conditional expression returns false

***while***, loop executed while condition expression is true

***do***, loop executed in conjunction with a termining while

***for***, loop executed with an initiallization statement; conditional expression for termination; and expression evaluated on each iteration

***for*** <value> ***in*** <array>, loop executed sequentially over all values in an array; the types of the variables <value> and <array> must match.

## Internal functions

Internal as well as externally defined functions have the general form

return\_type function\_name (*arg1, arg2, ...*)

where arg1, arg2, ... are arguments (variables or constants) supplied to the function and return\_type indicates that type of the value returned by the function. Functions with non-void return types are typically used in assignment statements; however, the returned value can be ignored. Functions without arguments must nevertheless include the parentheses. In some cases, the order and type of arguments is fixed. In other cases, the order and/or type of arguments is variable. Multiple arguments must be serparated by commas.

The core library of internal functions are listed below. Additional functions are available for use in scripts. In many cases, the return types depend on the type of the corresponding argument.

### Array functions

*double* **mean(*array [, first\_index, last\_index]*)**

Returns the mean of an ***array*** as a double value. The optional integer arguments first\_index and last\_index restrict the range of the calculation.

*type\_of\_array* **min(*array [, first\_index, last\_index]*)**

Returns the minimum of a numerical ***array*** as a value with the type of the array. The optional integer arguments first\_index and last\_index restrict the range of the calculation.

*type\_of\_array* **max(*array [, first\_index, last\_index]*)**

Returns the maximum of a numerical ***array*** as a value with the type of the array. The optional integer arguments first\_index and last\_index restrict the range of the calculation.

*type\_of\_array* **initial(*array [, first\_index, last\_index]*)**

Returns the initial value of a numerical ***array*** as a value with the type of the array. The optional integer arguments first\_index and last\_index restrict the range of the calculation.

*type\_of\_array* **final(*array [, first\_index, last\_index]*)**

Returns the final value of a numerical ***array*** as a value with the type of the array. The optional integer arguments first\_index and last\_index restrict the range of the calculation.

*double* **mid(*array [, first\_index, last\_index]*)**

Returns the midpoint value of a numerical ***array*** as a double value. The optional integer arguments first\_index and last\_index restrict the range of the calculation.

*type\_of\_array* **maxminusmin(*array [, first\_index, last\_index]*)**

Returns the difference between the maximum and minimum values of a numerical ***array*** as a value with the type of the array. The optional integer arguments first\_index and last\_index restrict the range of the calculation.

*type\_of\_array* **finalminusinitial(*array [, first\_index, last\_index]*)**

Returns the difference between the final and initial values of a numerical ***array*** as a value with the type of the array. The optional integer arguments first\_index and last\_index restrict the range of the calculation.

*double* **slope(*x\_array, y\_array [, first\_index, last\_index]*)**

Returns the linear least squares slope after fitting the slope and intercept of a numerical ***x\_array*** and ***y\_array*** as a double value. The optional integer arguments first\_index and last\_index restrict the range of the calculation.

*double* **xhalfdy(*x\_array, y\_array [, first\_index, last\_index]*)**

Returns the x value in a numberical ***x\_array*** that corresponds to the half delta y value in a numerical ***y\_array*** as a double value. The optional integer arguments first\_index and last\_index restrict the range of the calculation.

*double* **yhalfdy(*y\_array [, first\_index, last\_index]*)**

Returns the y value corresponding to the half delta y value in a numerical ***y\_array*** as a double value. The optional integer arguments first\_index and last\_index restrict the range of the calculation.

*double* **xooedy(*x\_array, y\_array [, first\_index, last\_index]*)**

Returns the x value in a numberical ***x\_array*** that corresponds to the 1/e delta y value in a numerical ***y\_array*** as a double value. The optional integer arguments first\_index and last\_index restrict the range of the calculation.

*double* **yooedy(*y\_array [, first\_index, last\_index]*)**

Returns the y value corresponding to the 1/e delta y value in a numerical ***y\_array*** as a double value. The optional integer arguments first\_index and last\_index restrict the range of the calculation.

*type\_of\_x\_array*  **xymax(*x\_array, y\_array [, first\_index, last\_index]*)**

Returns the x value in a numberical ***x\_array*** that corresponds to the maximum y value in a numerical ***y\_array*** as a value with the type determined by the *x\_array* type. The optional integer arguments first\_index and last\_index restrict the range of the calculation.

*type\_of\_x\_array* **xymin(*x\_array, y\_array [, first\_index, last\_index]*)**

Returns the x value in a numberical ***x\_array*** that corresponds to the minimum y value in a numerical ***y\_array*** as a value with the type determined by the *x\_array* type. The optional integer arguments first\_index and last\_index restrict the range of the calculation.

*int* **indexymax(*y\_array [, first\_index, last\_index]*)**

Returns the index of the maximum y value in a numerical ***y\_array*** as type double. The optional integer arguments first\_index and last\_index restrict the range of the calculation.

*int* **indexymin(*array [, first\_index, last\_index]*)**

Returns the index of the maximum y value in a numerical ***y\_array*** as type double. The optional integer arguments first\_index and last\_index restrict the range of the calculation.

*double* **sum(*array [, first\_index, last\_index]*)**

Returns the sum of an ***array*** as a double value. The optional integer arguments first\_index and last\_index restrict the range of the calculation.

*int* **subfirst(*array [, first\_index, last\_index]*)**

Subtracts the first value of a numerical ***array*** from all elements in the array. The optional integer arguments first\_index and last\_index restrict the range of the calculation. The index of the array element containing the first value is returned.

*int* **submin(*array [, first\_index, last\_index]*)**

Subtracts the minimum value of a numerical ***array*** from all elements in the array. The optional integer arguments first\_index and last\_index restrict the range of the calculation. The index of the array element containing the minimum value is returned.

*int* **divmax(*array [, first\_index, last\_index]*)**

Divides all elements of a numerical ***array*** by the maximum value in ***array***. The optional integer arguments first\_index and last\_index restrict the range of the calculation. The index of the array element containing the maximum value is returned.

*int* **divmin(*array [, first\_index, last\_index]*)**

Divides all elements of a numerical ***array*** by the minimum value in ***array***. The optional integer arguments first\_index and last\_index restrict the range of the calculation. The index of the array element containing the minimum value is returned.

**rsum(*array [, first\_index, last\_index]*)**

Calculates the running sum (integral) of a numerical ***array***. The optional integer arguments first\_index and last\_index restrict the range of the calculation.

**rdiff(*array [, first\_index, last\_index]*)**

Calculates the running difference (derivative) of a numerical ***array***. The optional integer arguments first\_index and last\_index restrict the range of the calculation.

### Logarithmic and exponential functions

*double* **log(*arg*)**

Returns the natrual logarithm of ***arg***.

*double* **log10(*arg*)**

Returns the base 10 logarithm of ***arg***, which must be a numerical constant or numerical variable.

*double* **exp(*arg*)**

Returns e raised to the power of ***arg***, which must be a numerical constant or numerical variable.

*double* **pow(*arg1, arg2*)**

Returns ***arg1*** raised to the power of ***arg2***, both of which must be a numerical constants or numerical variables.

*double* **sqrt(*arg*)**

Returns the square root of ***arg***, which must be a numerical constant or numerical variable.

### Trigonometric functions

*double* **sin(*arg*)**

Returns the sine of ***arg***, which must be a numerical constant or numerical variable. The return value has the type of ***arg***.

*double* **cos(*arg*)**

Returns the cosine of ***arg***, which must be a numerical constant or numerical variable. The return value has the type of ***arg***.

*double* **tan(*arg*)**

Returns the tangent of ***arg***, which must be a numerical constant or numerical variable. The return value has the type of ***arg***.

*double* **asin(*arg*)**

Returns the arcsine of ***arg***, which must be a numerical constant or numerical variable. The return value has the type of ***arg***.

*double* **acos(*arg*)**

Returns the arccosine of ***arg***, which must be a numerical constant or numerical variable. The return value has the type of ***arg***.

*double* **atan(*arg*)**

Returns the arctangent of ***arg***, which must be a numerical constant or numerical variable. The return value has the type of ***arg***.

*double* **sinh(*arg*)**

Returns the hyperbolic sine of ***arg***, which must be a numerical constant or numerical variable. The return value has the type of ***arg***.

*double* **cosh(*arg*)**

Returns the hyperbolic cosine of ***arg***, which must be a numerical constant or numerical variable. The return value has the type of ***arg***.

*double* **tanh(*arg*)**

Returns the hyperbolic tangent of ***arg***, which must be a numerical constant or numerical variable. The return value has the type of ***arg***.

### Statistical functions

*type\_depends\_on\_keyword* **statistic(*key*, *arg*)**

where ***arg*** is an array of type int or double and ***key*** is one of the following strings:

"sample size"

"index of minimum"

"index of maximum"

"minimum"

"maximum"

"extent"

"sum"

"mean"

"median"

"standard deviation"

"standard error"

"variance"

"skewness"

"kurtosis"

"first moment"

"second moment"

"third moment"

"fourth moment"

"first cumulant"

"second cumulant"

"third cumulant"

"forth cumulant"

### Other functions

*type\_of\_arg* **abs(*arg*)**

Returns the absolute value of ***arg***, which must be a numerical constant or numerical variable. The return value has the type of ***arg***.

*double* **ran(seed)**

Returns a random number between 0.0 and 1.0. Different integer values of ***seed*** produce different sequences of random number.
