# Supplementary material for: Structural Organization and Dynamics of Homodimeric Cytohesin Family Arf GTPase Exchange Factors in Solution and on Membranes
Source: Structure. 2019 Dec 3;27(12):1782–1797.e7. doi: 10.1016/j.str.2019.09.007 (PMC6948192; doi:10.1016/j.str.2019.09.007)
Supplement: Data S1. Software, Related to the STAR Methods [file mmc3.zip › Software/Dela App Scripts/Dela.app/Contents/Resources/Dela.help/Contents/Resources/English.lproj/pgs/itc.html]

xml version="1.0" encoding="utf-8"?


ITC


Home

Contents


# ITC

## Model function

Y (X; ***P***) = ***P3*** + q2 + ***P6*** / ***P7*** \* (q1 + q2) / 2 - q1

where

q1 = 0.5 \* ***P0*** \* mt1 \* ***P2*** \* ***P7*** \* (b1 - sqrt(b1^2 - 4 \* ac1))

b1 = 1.0 + xt1 / (***P0*** \* mt1) + ***P1*** / (***P0*** \* mt1)

ac1 = xt1 / (***P0*** \* mt1)

mt1 = ***P4*** \* (1.0 - 0.5 \* (x - 1.0) \* ***P6*** / ***P7***)) / (1.0 + 0.5 \* (x - 1.0) \* ***P6*** / ***P7***))

xt1 = ***P5*** \* (1.0 - 0.5 \* (x - 1.0) \* ***P6*** / ***P7***)) \* (x - 1.0) \* ***P6*** / ***P7***)

q2 = 0.5 \* ***P0*** \* mt2 \* ***P2*** \* ***P7*** \* (b2 - sqrt(b2^2 - 4 \* ac2))

b2 = 1 + xt2 / (***P0*** \* mt2) + ***P1*** / (***P0*** \* mt2)

ac2 = xt / (***P0*** \* mt2)

mt2 = ***P4*** \* (1.0 - 0.5 \* x \* ***P6*** / ***P7***) / (1.0 + 0.5 \* x \* ***P6*** / ***P7***)

xt2 = ***P5*** \* (1.0 - 0.5 \* x \* ***P6*** / ***P7***) \* x \* ***P6*** / ***P7***

## Parameters

### Non-term parameters

***P0*** - Stoichiometry (n)

***P1*** - Dissociation constant (Kd)

***P2*** - Binding enthalpy (ΔH)

***P3*** - Heat of dilution/mixing

***P4*** - Concentration of the fixed component

***P5*** - Concentration of the variable component

***P6*** - Injection volume

***P7*** - Initial total volume

### Term parameters

***None***

For more general information about models, see Model Functions.
