# Supplementary material for: Structural Organization and Dynamics of Homodimeric Cytohesin Family Arf GTPase Exchange Factors in Solution and on Membranes
Source: Structure. 2019 Dec 3;27(12):1782–1797.e7. doi: 10.1016/j.str.2019.09.007 (PMC6948192; doi:10.1016/j.str.2019.09.007)
Supplement: Data S1. Software, Related to the STAR Methods [file mmc3.zip › Software/Dela App Scripts/Dela.app/Contents/Resources/Dela.help/Contents/Resources/English.lproj/pgs/model_functions.html]

xml version="1.0" encoding="utf-8"?


Model functions


Home

Contents


# Model functions

## General form

Working model functions in Dela are constructed from a master list of model function templates and have the following general form:

y (x, ***P***) = f(x, ***Pnt***) + ∑ ***Pta*** f(x, ***Ptf***)

where

***P*** represents the set of all parameters;

***Pnt*** represents the set of parameters not included in the summation ('non-term parameters');

***Pta*** represents the set of term amplitude parameters in the summation;

***Ptf*** represents the set of term function parameters in the summation.

Every working model funciton has exactly one copy of the non-term parameters (***Pnt***) and at least one copy of the term parameters (***Pta*** and ***Ptf***). Working model functions with two or more terms simply include additional copies of the term parameters. Note that most but not all model functions have term parameters.

## Model functions

### Binding kinetics

Binding kinetics

### Distribution / Line Shape

Gaussian

Higher Order Gaussian

Log Normal

### Enzyme Kinetics

Michaelis-Menten

Pseudo First Order Michaelis-Menten

Pseudo First Order Michaelis-Menten Catalytic Efficiency

### Equilibrium Binding

Hill Binding

Langmuir Binding

Quadratic Binding

ITC

### Exponential

Exponential Decay

### Linear and Polynomial

Line

Polynomial

### Linear Combination

Data Object
