# Supplementary material for: Structural Organization and Dynamics of Homodimeric Cytohesin Family Arf GTPase Exchange Factors in Solution and on Membranes
Source: Structure. 2019 Dec 3;27(12):1782–1797.e7. doi: 10.1016/j.str.2019.09.007 (PMC6948192; doi:10.1016/j.str.2019.09.007)
Supplement: Data S1. Software, Related to the STAR Methods [file mmc3.zip › Software/Dela App Scripts/Dela.app/Contents/Resources/Dela.help/Contents/Resources/English.lproj/pgs/new_data.html]

xml version="1.0" encoding="utf-8"?


New Data


Home

Contents


# Creating new data

## Creating new data

New data can be created as one or more independent data objects or as a series of related data objects. X values can be generated using regular expressions as a funciton of the previous x value. Y values can generated as a function of the x values and an iterator corresponding to the index of the data object. Gaussian noise can added to the Y values if desired.

### To create a new data object:

1. Click ***New Data→New Data*** from the document toolbar.

2. Modify options and click  ***Create***.

A new sheet with the new data objects will appear in the document's contents.

## New data options

The following options are presented when ***New Data→New Data*** is selected from the document toolbar.

### Description and Labels

UTF-8 compatible unicode descriptions and labels.

• ***Sheet*** - description for the sheet.

• ***Data*** - description for the data object(s).

• ***X*** - description for the x values (e.g. "time (s)").

• ***Y*** - description for the y values (e.g. "intensity (a.u.)")

### Dimensions

The size and number of data objects.

• ***# points*** - number of points in each data object.

• ***# data objects*** - number of data objects.

### X Values

Calcution of x values using the DELA interpreter.

• ***Expression for initial value*** - a constant or regular expression of i for the initial X value (i.e. the X value with index 0). Here, i is index of the data object. In the example above i ranges from 0 to 4 since the number of data objects is five and DELA counts from zero.

• ***Relationship to previous value*** - a regular expression of x and i for calculation of the current X value (with index N) from the previous X value (with index N - 1). Here, x refers to the previous x values and i to the index of the data object in the series of data object. In the example above, the X value is simply

### Y Values

Calcution of y values using the DELA interpreter.

• ***Relationship to X values*** - a regular expression of x and i for calculation of the Y values. Here, x refers to the X value with the same index as the Y value in the corresponding data arrays and i refers to the index of the data object in the series of data objects. In the example above, i is used to create a series of data objects with Y values calculated from a expression for an exponetial decay where the rate constants increase by a factor of two for each new data object.

### Errors

Errors and optional addition of Gaussian noise.

• ***Standard Deviation*** - the standard deviation of the errors. Also used for generation of Gaussian noise.

• ***Add Gaussian noise***  - if selected, Gaussian noise distributed about a mean of zero will be generated with the random number seed and standard deviation proivded will be added to the Y values.

• ***Seed*** - an integer value for the random number generator. Different seed values result in a distinct though reproducible random number series. How random and Guaissian? Who knows. Probably good enough for simulating the effect of noise on routine scientific data analyses but not recommend for encryption or other data critical applications.
