# Supplementary material for: Structural Organization and Dynamics of Homodimeric Cytohesin Family Arf GTPase Exchange Factors in Solution and on Membranes
Source: Structure. 2019 Dec 3;27(12):1782–1797.e7. doi: 10.1016/j.str.2019.09.007 (PMC6948192; doi:10.1016/j.str.2019.09.007)
Supplement: Data S1. Software, Related to the STAR Methods [file mmc3.zip › Software/Dela App Scripts/Dela.app/Contents/Resources/Dela.help/Contents/Resources/English.lproj/pgs/new_instrument_response.html]

xml version="1.0" encoding="utf-8"?


New instrument response


Home

Contents


# New instrument response

## Setting the instrument response

Some experiments are modeled with functions that require an instrument response. An instrument response can be imported as a data object and then set as the designated instrument response for any data object(s). Multiple data objects may share a common instrument response; however, each data object has only one designated instrument reponse at any given time.

### To set a new instrument response for data object(s):

1. In the document contents, select the data objects, sheets, and/or plots with the data for which the instrument response will be set.

2. Click ***New Data→New Instrument Response*** from the document toolbar.

3. Change the data source if necessary, provide the Data label for the instrument response and click  ***Apply***.

## New instrument response options

The following options are presented when ***New Data→New Instrument Response*** is selected from the document toolbar.

### Data from

Selects the intended data source.

### New instrument response

Specifies the instrument response data object.

• ***Label of new instrument response*** - the Data label (e.g. Data4) of the instrument response.
