# Supplementary material for: Structural Organization and Dynamics of Homodimeric Cytohesin Family Arf GTPase Exchange Factors in Solution and on Membranes
Source: Structure. 2019 Dec 3;27(12):1782–1797.e7. doi: 10.1016/j.str.2019.09.007 (PMC6948192; doi:10.1016/j.str.2019.09.007)
Supplement: Data S1. Software, Related to the STAR Methods [file mmc3.zip › Software/Dela App Scripts/Dela.app/Contents/Resources/Dela.help/Contents/Resources/English.lproj/pgs/new_model_data.html]

xml version="1.0" encoding="utf-8"?


New model data


Home

Contents


# New model data

## Setting the model data

Some data can represented as a function (e.g. a linear combination) of other data objects (see Data Object model), which may include theoretical models and/or experimental data. Each data objects can have a set of one or more model data objects that may be shared with other data objects; however, only one set of model data objects can be designated for each data object at any given time. Model data objects are also required to have the same number of points as the data object to be represented by the model data.

### To set the model data object(s) for data object(s):

1. In the document contents, select the data objects, sheets, and/or plots with the data for which the model data will be set.

2. Click ***New Data→New Model Data***  from the document toolbar.

3. Change the data source if necessary, provide the Data label, sheet index, or plot index for the instrument response and click  ***Apply***.

## New model data options

The following options are presented when ***New Data→New Model Data*** is selected from the document toolbar.

### Data from

Selects the intended data source.

### New model data

Specifies the model data object(s).

• ***New model data popup menu*** - selects the option for specificying the new model data: Data with label, Data from sheet with index, or Data from plot with index.

• ***Data Label, Sheet Index, or Plot Index field*** - the Data label (e.g. Data4), sheet index (in this example 2) or plot index for the model data. The index for a sheet or plot corresponds to the index in the document contents.
