# Supplementary material for: Structural Organization and Dynamics of Homodimeric Cytohesin Family Arf GTPase Exchange Factors in Solution and on Membranes
Source: Structure. 2019 Dec 3;27(12):1782–1797.e7. doi: 10.1016/j.str.2019.09.007 (PMC6948192; doi:10.1016/j.str.2019.09.007)
Supplement: Data S1. Software, Related to the STAR Methods [file mmc3.zip › Software/Dela App Scripts/Dela.app/Contents/Resources/Dela.help/Contents/Resources/English.lproj/pgs/new_plot.html]

xml version="1.0" encoding="utf-8"?


New Data


Home

Contents


# Creating and updating plots

## Creating new plots

New plots can be created from any combination of data in the Data List, Sheets, or existing plots.

### To create a new plot:

1. Select or highlight data of interest in the Data List or a Sheet or highlight data, sheets or plots in the Document Contents..

2. Click ***New Plot*** from the document toolbar or from the application's Plot menu.

3. Change the data source if necessary and click  ***New Plot***.

If one or more data buffers are highlighted/selected, a single plot will be created that displays all of the highlighted/selected data. If multiple sheets are highlighted (but nothing else), one plot will be created for each sheet.

## Updating plots

Plots are automatically updated following most changes that affect displayed data or graphics attributes. Left clicking within the view region of a plot or highlighting a plot in the Document Contents causes the plot to be updated.
