# Supplementary material for: Structural Organization and Dynamics of Homodimeric Cytohesin Family Arf GTPase Exchange Factors in Solution and on Membranes
Source: Structure. 2019 Dec 3;27(12):1782–1797.e7. doi: 10.1016/j.str.2019.09.007 (PMC6948192; doi:10.1016/j.str.2019.09.007)
Supplement: Data S1. Software, Related to the STAR Methods [file mmc3.zip › Software/Dela App Scripts/Dela.app/Contents/Resources/Dela.help/Contents/Resources/English.lproj/pgs/overview.html]

xml version="1.0" encoding="utf-8"?


Overview


Home

Contents


# Overview

## Description

DELA is a document-based Intel Mac OSX application with a variety of tools for data import/export, graphical visualization/analysis, processing, local and global maximum likelihood fitting, and other functions. Documents can be saved, data can be imported/exported in a variety of text formats, and publication quality plots can be exported in common image formats.

## Extensibility

The basic application is extensible via an embedded Python interpreter, which includes an extensive API consisting of multiple automatically defined modules that facilitate data transfer to/from the Python side and control of application functionality within Python scripts.

## Data storage

Data are stored in objects with arrays for x, y, and error values. Every data object has a unique identifying ‘Label’ of the form Data#, where # is an integer automatically assigned when the data objected is created.

## Organization

Data objects can be organized in ‘sheets’. Unlike typical spread sheets, however, data objects may be shared between different sheets. Data objects can be visualized/edited in tabular from, either individually or collectively at the level of a sheet. Data, plots, and sheets can be further organized into groups of related content.

## Recommended

A three button mouse with a scroll wheel is recommended but not essential. A right mouse button click, for example, equivalent to a control-click.
