# Supplementary material for: Structural Organization and Dynamics of Homodimeric Cytohesin Family Arf GTPase Exchange Factors in Solution and on Membranes
Source: Structure. 2019 Dec 3;27(12):1782–1797.e7. doi: 10.1016/j.str.2019.09.007 (PMC6948192; doi:10.1016/j.str.2019.09.007)
Supplement: Data S1. Software, Related to the STAR Methods [file mmc3.zip › Software/Dela App Scripts/Dela.app/Contents/Resources/Dela.help/Contents/Resources/English.lproj/pgs/parameters.html]

xml version="1.0" encoding="utf-8"?


Parameters panel


Home

Contents


# Parameters panel

## Parameter editing, estimation and fitting

The Parameters panel is an integrated interface for viewing, editting, estimating, and fitting model function parameters. It is designed to be used in conjunction with a highlighted plot containing the data to be modelled.

## Data and model menus

The data popup menu lists all of the data objects contained in the data list of the current highlighted plot in addition to a Global Paraemters object. The Model popup menu lists all of the models associated with the data object or Global Parameters object shown in the Data popup menu.

### To view the parameters for a model function associated with a given data object:

1. Choose the data of interest from the Data menu

2. Choose the model of interest from the Model menu

## Parameters table

The current parameter values and related properties as well as switches that control the behavoir of parameters during fitting are displayed in the the Parameters table. The table diplays parameters associated with the Data and models objects shown in the Data and and Model menus.

## Action Buttons

### Estimate Parameters

Rough estimation of parameter values. For some models, certain parameter values such as concentrations may need to be entered first.

### Set Parameters

Opens the Set Parameters panel, which can be used to quickly enter parameter values associated with different data objects in cases where the values are related by a mathematical expression. The value for the first data object in the series should be entered first.

### Store Parameters

Stores a copy of all values and settings in the Parameters table for all data objects listed in the data menu. Applies only to the currently selected model. Note that any previously stored values will be replaced with the current values.

### Recall Parameters

Recalls the most recently stored copy of all values and settings in the Parameters table for all data objects listed in the data menu. Applies only to the currently selected model.

### Fit Marquardt

Initiates maximum likelihood parameter estimation using Levenburg-Marquardt algorithm. This non-linear fitting algorithm is fast but may not work well for complex or global models; however, it may still be useful in such cases provide an improved starting point for Simplex fitting.

### Fit Simplex

Initiates maximum likelihood parameter estimation using the Simpelx algorithm. Though relatively slow, this non-linear fitting algorithm can get out of local minima. This is the recommended algorithm for gloal fits.

### Save parameters

Save a copy of the values and settings in the parameters table to a text file.

### Parameters -> Data

Create a new sheet with data objects containing the current parameter values for all data objects listed in the data menu.

### Copy Parameters

Copy the parameters values and settings in the parameters table for all data objects listed in the data menu.

### Fit Options

Opens the Fit Options sheet to view and/or edit settings that control fitting by the Marquardt and Simplex algorithms.

### Calculate Model

Uses the current paramter values to calculate the model for all data objects listed in the data menu. Useful for updating the model after manual editting of parameter values or after using Set Parameters.

### Value -> Target

Copies the current parameters values to the target values. Which values are copied depends on which rows are selected in the parameters table.

### Abort Fit

Aborts fitting after completion of the current iteration.

## Control Buttons

### Local/Global

Determines whether parameters selected as global in the parameters table are fit as local or global parameters.

### Apply to all

Determines whether edits in the parameters table are copy to all data objects in the data menu or apply only to the currently selected data object.

## Text Fields

### Fit progress

Updated after completion of each overall iteration of the Marquardt or Simplex algorithms. Note that the time required for completion of a given iteration can vary substantially.

### Fit status

Updated after completion of fitting by the Marquardt or Simplex algorithms. The displayed merit function value corresponds to the negative log of the likelihood function, which reduces to chi squarred if no parameters are restrained to their target values (i.e. if none of the ML boxes are checked).

### ML Weight

Overall weight applied to increase or decrease the contribution of the target restraints to the maximum likelihood function. For example, setting the ML weight to 2 would be equivalent to mulitiply all of the individual target errors by 2.
