# Supplementary material for: Structural Organization and Dynamics of Homodimeric Cytohesin Family Arf GTPase Exchange Factors in Solution and on Membranes
Source: Structure. 2019 Dec 3;27(12):1782–1797.e7. doi: 10.1016/j.str.2019.09.007 (PMC6948192; doi:10.1016/j.str.2019.09.007)
Supplement: Data S1. Software, Related to the STAR Methods [file mmc3.zip › Software/Dela App Scripts/Dela.app/Contents/Resources/Dela.help/Contents/Resources/English.lproj/pgs/pfo_michaelis_menten.html]

xml version="1.0" encoding="utf-8"?


Pseudo First Order Michaelis-Menten


Home

Contents


# Pseudo First Order Michaelis-Menten

## Model function

Y (X; ***P***) = **P4** \* (X + ***P8***) + ***P6*** \* (1 - exp(-***P7*** \* (X + ***P8***))) + ec \* ***P2*** \* (***P1*** + (***P0*** - ***P1***) \* exp(-kobs \* (X + ***P8***)))

where

kobs = ***P3*** + ec \* ***P9*** \* ***P11*** / (***P10*** + ***P11***) [ + ... + ec \* ***Pn-2*** \* ***Pn*** / (***Pn-1*** + ***Pn***) ]

ec = 100 / (100 - P5 \* (X + ***P8***))

## Parameters

### Non-term parameters

***P0*** - Initial conversion factor (Signal units / unit [substrate])

***P1*** - Final conversion factor (Signal units / unit [substrate])

***P2*** - Initial substrate concentration

***P3*** - Intrinsic rate constant

***P4*** - Baseline slope (Signal units / second)

***P5*** - Evaporation rate (% volume decrease / second)

***P6*** - Baseline amplitdue

***P7*** - Baseline rate constant (1/seconds)

***P8*** - time offset (seconds)

### Term parameters

***P9 ... Pn-2*** - kcat

***P10 ... Pn-1*** - km

***P11 ... Pn-1*** - total enzyme concentration

For more general information about models, see Model Functions.
