# Supplementary material for: Structural Organization and Dynamics of Homodimeric Cytohesin Family Arf GTPase Exchange Factors in Solution and on Membranes
Source: Structure. 2019 Dec 3;27(12):1782–1797.e7. doi: 10.1016/j.str.2019.09.007 (PMC6948192; doi:10.1016/j.str.2019.09.007)
Supplement: Data S1. Software, Related to the STAR Methods [file mmc3.zip › Software/Dela App Scripts/Dela.app/Contents/Resources/Dela.help/Contents/Resources/English.lproj/pgs/plot.html]

xml version="1.0" encoding="utf-8"?


Working with plots


Home

Contents


# Working with plots

## Plots objects

Plots display a variety of different objects that can be selected and in many cases altered. Left-clicking selects an objects. Double left-clicking initiiates object-specific actions.

### Selectable objects and double left-click actions:

• ***axes*** - double left-click opens Axes paenl

• ***data points*** - double left-click opens Symbols panel

• ***text labels*** - double left-click initiates text editing

• ***ruler*** - double left-click opens Plot Metrics panel

## Scale and translation

The plot view can be scaled and translated using simple mouse or key actions. Scaling affects the plot view but does not change the dimensions of the plot relative to the printed page and has no affect on export of TIFF images.

### Mouse actions:

• ***scroll wheel*** - scales both the view and page (i.e. zooms in and out)

• ***left-drag on background*** - translates the view but not the page

## Resizing axes

The dimensions of a plot can be changed by left-dragging in the selection handles of selected axes or via the Plot Metrics panel.

## Right mouse button Menu

The right mouse button menu faciilitates addition of labels, display of plot objects, changes to the Scale and Translation, resizing, masking or removing data, and also provides access to the Plot Metrics and Model Properties panels. Many of the menu items have equivalent key board short cuts.

## Labels

There are four varieties text objects: the title, axis labels, tic labels and plot labels. The title, axis labels and tic labels can optionally displayed. Editing of tic labels is restricted to character attributes (e.g. font, font size, color, etc) and affects all of the tic labes for a given axes. The location of labels can be adjusted by left-dragging with the mouse or using the arrow keys. To add a new label, position the cursor at the desired location and choose ***Add Label*** from the right mouse button menu.
