# Supplementary material for: Structural Organization and Dynamics of Homodimeric Cytohesin Family Arf GTPase Exchange Factors in Solution and on Membranes
Source: Structure. 2019 Dec 3;27(12):1782–1797.e7. doi: 10.1016/j.str.2019.09.007 (PMC6948192; doi:10.1016/j.str.2019.09.007)
Supplement: Data S1. Software, Related to the STAR Methods [file mmc3.zip › Software/Dela App Scripts/Dela.app/Contents/Resources/Dela.help/Contents/Resources/English.lproj/pgs/polynomial.html]

xml version="1.0" encoding="utf-8"?


Polynomial


Home

Contents


# Polynomial

## Model function

Y (X; ***P***) = ***P0*** + ***P2***\*(X - ***P1***) [ + ***P3*** \* (X - ***P1***)^2 + ***P4*** \* (X - ***P1***)^3 + ... + ***Pn*** \* (X - ***P1***)^n-1 ]

## Parameters

### Non-term parameters

***P0*** - constant

***P1*** - X offset

### Term parameters

***P2*** - amplitude of linear term

***P3*** - amplitde of quadratic term

***P4*** - amplitide of cubic term

***Pn*** - amplitide of nth order term
