# Supplementary material for: Structural Organization and Dynamics of Homodimeric Cytohesin Family Arf GTPase Exchange Factors in Solution and on Membranes
Source: Structure. 2019 Dec 3;27(12):1782–1797.e7. doi: 10.1016/j.str.2019.09.007 (PMC6948192; doi:10.1016/j.str.2019.09.007)
Supplement: Data S1. Software, Related to the STAR Methods [file mmc3.zip › Software/Dela App Scripts/Dela.app/Contents/Resources/Dela.help/Contents/Resources/English.lproj/pgs/process.html]

xml version="1.0" encoding="utf-8"?


Process panel


Home

Contents


# Process panel

## Data Processing

The Process panel facillitates numerical transformation of data through various arithmetic operations, functions, and interpreted mathematical expressions. More elaborate proessing, analysis, or alogrithmic manipulation is facillitated through a seperate interface for script interpretation.

## Target

Numerical transformations can be applied to the x values, y values, or errors associated with selected or highlighted data objects. The processor defines a variable (t) that can be assigned to the target of interest.

### To specify the target for data processing:

1. Highlight or select data for processing in the Document Contents, Data List or Sheet Data List

2. Choose a target from the left menu

3. Choose the data source from the right menu

## Action

Left clicking the Process button initiates the numerical transformation defined by the menu selections and field values. Actions are grouped into general classes including Arithmetic operations, Exponential functions, Trigonometric functions, Other functions, and Interpret f(t).

### To specify an action for data processing:

1. Choose a general action class

2. Choose a specific action from the relevant popup menu

3. Enter a value for the constant (Arithmetic operations and Exponential functions) or the desired function of the target variable t.

### Arithmetic operations

• ***t + constant*** - addition of a constant to the target variable

• ***constant - t*** - substraction of the target variable from a constant

• ***t \* constant*** - multiplication of the target variable by a constant

• ***t / constant*** - division of the target variable by a constant

• ***constant / t*** - division of a constant by the target variable

The value of constant must be supplied in the field to the right of the Arithmetic operations popup menu.

### Exponential functions

• ***log(t)*** - natural logarithm of the target variable

• ***log10(t)*** - base 10 logarithm of the target variable

• ***exp(t)*** - e raised to the power of the target variable

• ***10^t*** - 10 raised to the power of the target variable

• ***sqrt(t)*** - square root of the target variable

• ***t^constant*** - the target variable raised to the power of a constant

• ***contstant^t*** - a constant raised to the power of the target variable

### Trigonometric functions

• ***sin(t)*** - sine of the target variable

• ***cos(t)*** - cosine of the target variable

• ***tan(t)*** - tangent of the target variable

• ***asin(t)*** - arcsine of the target variable

• ***acos(t)*** - arccosine of the target variable

• ***atan(t)*** - arctangent of the target variable

• ***sinh(t)*** - hyperbolic sine of the target variable

• ***cosh(t)*** - hyperbolic cosine of the target variable

• ***tanh(t)*** - hyperbolic tangent of the target variable

### Other functions

• ***substract first value*** - subtract the value of the first element in the target variable array

• ***substract last value*** - subtract the value of the last element in the target variable array

• ***subtract minimum value*** - subtract the minimum value in the target variable array

• ***subtract maximum value*** - subtract the maximum value in the target variable array

• ***divide by first value*** - divide by the first value in the target variable array

• ***divide by last value*** - divide by the last value in the target variable array

• ***divide by minimum value*** - divide by the minimum value in the target variable array

• ***divide by maximum value*** - divide by the maximum value in the target variable array

• ***integrate*** - sum of all values in the target variable array

• ***differentiate*** - simple numerical derivative calculated for each element of the target variable array

### Interpret f(t)

Functions are interpretted via a recursive descent parsar with a C-like syntax. The interpreter understands regular mathematical expressions that are functions of the target variable (t) and/or other internal variables as noted below. The Interpret f(t) functionallity is designed for convenient processing of data using a single mathematical expression.

**The following variables and shorthand notation can be used in addition to *t* to refer to values associated with data points:**

• ***i*** - data object iterator

• ***n*** - data array index iterator

• ***x*** - x value of the data point

• ***y*** - y value of the data point

• ***e*** - error values of the data

• ***d#*** - value of the target for the data object with the identifying integer #

• ***d#x*** - x value of the data point for the data object with the identifying integer #

• ***d#y*** - y value of the data point for the data object with the identifying integer #

• ***d#e*** - error value of the data point for the data object with the identifying integer #

• ***d#.x*** - x value of the data point for the data object with the identifying integer #

• ***d#.y*** - y value of the data point for the data object with the identifying integer #

• ***d#.e*** - error value of the data point for the data object with the identifying integer #

**Specific data points can be referred to as follows:**

• ***d#x[#]***

• ***d#y[#]***

• ***d#e[#]***

• ***d#.x[#]***

• ***d#.y[#]***

• ***d#.e[#]***

• ***Data#.x[#]***

• ***Data#.y[#]***

• ***Data#.e[#]***

The # symbol following *Data* or the shorthand notation *d* refers to the unique indentifying integer in the data label (e.g. # = 0 for Data0, # = 1 for Data1, ...). The # symbol in square brackets (i.e. [#}) refers to the array index, which ranges from 0 to the array size - 1 (e.g. if the array size is 100, the index can be any integer from 0 to 99). Array indices less than or greater than the array limits are replaced by the lower limit or upper limit, respectively.
