# Supplementary material for: Structural Organization and Dynamics of Homodimeric Cytohesin Family Arf GTPase Exchange Factors in Solution and on Membranes
Source: Structure. 2019 Dec 3;27(12):1782–1797.e7. doi: 10.1016/j.str.2019.09.007 (PMC6948192; doi:10.1016/j.str.2019.09.007)
Supplement: Data S1. Software, Related to the STAR Methods [file mmc3.zip › Software/Dela App Scripts/Dela.app/Contents/Resources/Dela.help/Contents/Resources/English.lproj/pgs/process_itc.html]

xml version="1.0" encoding="utf-8"?


Process ITC sheet


Home

Contents


# Process ITC sheet

## Processing ITC data

The Process ITC sheet facillitates baseline correction and integration of isothermal titration calorimetry data.

## Data source

Any number of data objects can be baseline corrected and integrated, provided the injection start times and intervals are identical.

### To specify data objects for data processing:

1. Highlight or select data for processing in the Document Contents, Data List or Sheet Data List

2. Choose the desired data source using the ***Data from*** the popip menu

## Injections, Baseline Correction and Integration

### Injections

• ***Start of first injection*** - should correspond to the beginning of the first injection in seconds. Note that a small "pre-injection" is not considered an injection.

• ***Injection interval*** - the time interval in seconds between the start of one injection and the start of the next injection

### Baseline Correction

• ***Averaging window*** - the time window in seconds preceding the start of each injection to be used for calculating the baseline slope during an injection. The averaging window should be large enough for a reasonable estimate of the average baseline value and small enough to exclude peaks.

### Integration

• ***Integration window*** - the time window in seconds used for peak integration. Should be large enough to include full peaks. Smaller integration windows may improve signal-to-noise without loss of data content, provided the window spans the entire peak. The appropriate window size is best judged by inspecting strong peaks with high signal-to-noise.
