# Supplementary material for: Structural Organization and Dynamics of Homodimeric Cytohesin Family Arf GTPase Exchange Factors in Solution and on Membranes
Source: Structure. 2019 Dec 3;27(12):1782–1797.e7. doi: 10.1016/j.str.2019.09.007 (PMC6948192; doi:10.1016/j.str.2019.09.007)
Supplement: Data S1. Software, Related to the STAR Methods [file mmc3.zip › Software/Dela App Scripts/Dela.app/Contents/Resources/Dela.help/Contents/Resources/English.lproj/pgs/process_svd.html]

xml version="1.0" encoding="utf-8"?


SVD sheet


Home

Contents


# SVD sheet

## Singular value decomposition

The SVD sheet facillitaties singular value decomposition (SVD) of a matrix with columns derived from the y-value arrays of two or more data objects. If the original matrix of data objects is defined as '**A**', then

**A** = **U** • **S** • **V**^T^

where **U** is matrix of orthonormal basis components, **S** is a diagonal matrix of singular values representing the overall contribution or weight of each correponsing column of **U**, and **V**^T^ is the transpose of an orthonormal matrix with columns representing the variation of each corresponding column of **U** over the range of columns in **A**.

## Data from

The data source must include two or more data objects having an identical number of points and sharing a common set of x-values.

### To specify data objects for data processing:

1. Highlight or select data for SVD in the Document Contents, Data List or Sheet Data List

2. Choose the desired data source using the ***Data from*** the popip menu

## Column Options

• Save all columns - all columns of the resulting U, S, and V matrices will be saved as new data objects

• Save most significant - the N columns of the U, S, and V matrices with the largest singular values will saved a new data objects. The value of N is specified in the ***# to save*** field.

## Reconstruction Options

• No reconstruction - SVD is performed without reconstruction.

• Reconstruct with # significant values - reconstructs a filtered approximation to the original matrix using the specified number of rank ordered components (i.e the components with the most significant singular values).

• Reconstruct with significant singular values threshould - reconstructs a filtered approximation to the original matrix using the components with singular values above the specified threshold.

• Reconstruct remainder with # significant singular values - reconstructs the remainder matrix with all components not included in the top # of significant values. This is done by zeroing the singular values for the top # of components.

• Reconstruct remainder with significant singular value threshold - same as the previous option but using the specified threshold for the singular values.

## Results

A sheet containing:

• The most significant columns of in rank order (U1, U2, ...)

• The corresponding columns of V premultiplied by the correspond singular values (S11V1, S22V2, ...)

• The rank ordered singular values - 'significant' values should standout above the remaining 'noise', which typically decays monotonically on a logrithmic scale unless the noise is not strictly radom or is unevenly distributed.

• The autocorrelation of each column of U - values range from 0 (uncorrelated) to 1 (perfectly correlated).

• The autocorrelation of each column of V - values range from 0 (uncorrelated) to 1 (perfectly correlated).
