# Supplementary material for: Structural Organization and Dynamics of Homodimeric Cytohesin Family Arf GTPase Exchange Factors in Solution and on Membranes
Source: Structure. 2019 Dec 3;27(12):1782–1797.e7. doi: 10.1016/j.str.2019.09.007 (PMC6948192; doi:10.1016/j.str.2019.09.007)
Supplement: Data S1. Software, Related to the STAR Methods [file mmc3.zip › Software/Dela App Scripts/Dela.app/Contents/Resources/Dela.help/Contents/Resources/English.lproj/pgs/python_module_DelaAxis.html]

xml version="1.0" encoding="utf-8"?


Python Module DelaAxis


Home

Top
Contents


# DelaAxis python module

## Overview

The DelaAxis module provides methods related to plot axes.

### Getting and setting display options

display

displayLabel

displayTics

displayTicLabels

setDisplay

setDisplayLabel

setDisplayTics

setDisplayTicLabels

### Getting and setting tic parameters

minorTicCount

setMinorTicCount

### Getting and setting scale options

autoScale

logScale

setAutoScale

setLogScale

### Getting and setting limits

minimum

maximum

setMinimum

setMaximum

## Methods

### autoScale

Returns the auto scale for a plot axis.

> ##### Usage
>
> *integer* DelaAxis.autoScale(*Plot\_index, Axis\_id\_string*)
>
> ##### Arguments
>
> *Plot\_index* is an integer value corresponding to the index of a plot in the document contents.
>
> *Axis\_id\_string* is a unicode or C-style (ASCII) string corresponding to the axis. Recognized axis id strings are: "x", "x2", "y", "y2", "r", and "r2" or the uppercase equivalents.
>
> ##### Return value
>
> An integer value corresponding to auto scale option if successful or -1 if a problem was encountered.

### display

Returns the display option for a plot axis.

> ##### Usage
>
> *integer* DelaAxis.display(*Plot\_index, Axis\_id\_string*)
>
> ##### Arguments
>
> *Plot\_index* is an integer value corresponding to the index of a plot in the document contents.
>
> *Axis\_id\_string* is a unicode or C-style (ASCII) string corresponding to the axis. Recognized axis id strings are: "x", "x2", "y", "y2", "r", and "r2" or the uppercase equivalents.
>
> ##### Return value
>
> An integer value corresponding to the display option if successful or -1 if a problem was encountered.

### displayLabel

Returns the display label option for a plot axis.

> ##### Usage
>
> *integer* DelaAxis.displayLabel(*Plot\_index, Axis\_id\_string*)
>
> ##### Arguments
>
> *Plot\_index* is an integer value corresponding to the index of a plot in the document contents.
>
> *Axis\_id\_string* is a unicode or C-style (ASCII) string corresponding to the axis. Recognized axis id strings are: "x", "x2", "y", "y2", "r", and "r2" or the uppercase equivalents.
>
> ##### Return value
>
> An integer value corresponding to the display label option if successful or -1 if a problem was encountered.

### displayTics

Returns the display tics option for a plot axis.

> ##### Usage
>
> *integer* DelaAxis.displayTics(*Plot\_index, Axis\_id\_string*)
>
> ##### Arguments
>
> *Plot\_index* is an integer value corresponding to the index of a plot in the document contents.
>
> *Axis\_id\_string* is a unicode or C-style (ASCII) string corresponding to the axis. Recognized axis id strings are: "x", "x2", "y", "y2", "r", and "r2" or the uppercase equivalents.
>
> ##### Return value
>
> An integer value corresponding to the display tics option if successful or -1 if a problem was encountered.

### displayTicLabels

Returns the display tic labels option for a plot axis.

> ##### Usage
>
> *integer* DelaAxis.displayTicLabels(*Plot\_index, Axis\_id\_string*)
>
> ##### Arguments
>
> *Plot\_index* is an integer value corresponding to the index of a plot in the document contents.
>
> *Axis\_id\_string* is a unicode or C-style (ASCII) string corresponding to the axis. Recognized axis id strings are: "x", "x2", "y", "y2", "r", and "r2" or the uppercase equivalents.
>
> ##### Return value
>
> An integer value corresponding to the display tic labels option if successful or -1 if a problem was encountered.

### logScale

Returns the log scale for a plot axis.

> ##### Usage
>
> *integer* DelaAxis.logScale(*Plot\_index, Axis\_id\_string*)
>
> ##### Arguments
>
> *Plot\_index* is an integer value corresponding to the index of a plot in the document contents.
>
> *Axis\_id\_string* is a unicode or C-style (ASCII) string corresponding to the axis. Recognized axis id strings are: "x", "x2", "y", "y2", "r", and "r2" or the uppercase equivalents.
>
> ##### Return value
>
> An integer value corresponding to log scale option if successful or -1 if a problem was encountered.

### maximum

Returns the maximum for a plot axis.

> ##### Usage
>
> *integer* DelaAxis.maximum(*Plot\_index, Axis\_id\_string*)
>
> ##### Arguments
>
> *Plot\_index* is an integer value corresponding to the index of a plot in the document contents.
>
> *Axis\_id\_string* is a unicode or C-style (ASCII) string corresponding to the axis. Recognized axis id strings are: "x", "x2", "y", "y2", "r", and "r2" or the uppercase equivalents.
>
> ##### Return value
>
> A double value corresponding to the axis maximum if successful or -1.0 if a problem was encountered.

### minimum

Returns the minimum for a plot axis.

> ##### Usage
>
> *integer* DelaAxis.minimum(*Plot\_index, Axis\_id\_string*)
>
> ##### Arguments
>
> *Plot\_index* is an integer value corresponding to the index of a plot in the document contents.
>
> *Axis\_id\_string* is a unicode or C-style (ASCII) string corresponding to the axis. Recognized axis id strings are: "x", "x2", "y", "y2", "r", and "r2" or the uppercase equivalents.
>
> ##### Return value
>
> A double value corresponding to the axis minimum if successful or -1.0 if a problem was encountered.

### minorTicCount

Returns the number of minor tics for a plot axis.

> ##### Usage
>
> *integer* DelaAxis.minorTicCount(*Plot\_index, Axis\_id\_string*)
>
> ##### Arguments
>
> *Plot\_index* is an integer value corresponding to the index of a plot in the document contents.
>
> *Axis\_id\_string* is a unicode or C-style (ASCII) string corresponding to the axis. Recognized axis id strings are: "x", "x2", "y", "y2", "r", and "r2" or the uppercase equivalents.
>
> ##### Return value
>
> An integer value corresponding to the number of minor tics if successful or -1 if a problem was encountered.

### setAutoScale

Sets the auto scale option for a plot axis.

> ##### Usage
>
> *integer* DelaAxis.setAutoScale(*Plot\_index, Axis\_id\_string, Scale\_option*)
>
> ##### Arguments
>
> *Plot\_index* is an integer value corresponding to the index of a plot in the document contents.
>
> *Axis\_id\_string* is a unicode or C-style (ASCII) string corresponding to the axis. Recognized axis id strings are: "x", "x2", "y", "y2", "r", and "r2" or the uppercase equivalents.
>
> *Scale\_option* is an integer value corresponding to the auto scale option. Recognized values are 0 (off) and 1 (on).
>
> ##### Return value
>
> The return value is 0 if successful or -1 if a problem was encountered.

### setDisplay

Sets the display option for a plot axis.

> ##### Usage
>
> *integer* DelaAxis.setDisplay(*Plot\_index, Axis\_id\_string, Display\_option*)
>
> ##### Arguments
>
> *Plot\_index* is an integer value corresponding to the index of a plot in the document contents.
>
> *Axis\_id\_string* is a unicode or C-style (ASCII) string corresponding to the axis. Recognized axis id strings are: "x", "x2", "y", "y2", "r", and "r2" or the uppercase equivalents.
>
> *Display\_option* is an integer value corresponding to the display option. Recognized values are 0 (hide) and 1 (display).
>
> ##### Return value
>
> The return value is 0 if successful or -1 if a problem was encountered.

### setDisplayLabel

Sets the display label option for a plot axis.

> ##### Usage
>
> *integer* DelaAxis.setDisplayLabel(*Plot\_index, Axis\_id\_string, Display\_option*)
>
> ##### Arguments
>
> *Plot\_index* is an integer value corresponding to the index of a plot in the document contents.
>
> *Axis\_id\_string* is a unicode or C-style (ASCII) string corresponding to the axis. Recognized axis id strings are: "x", "x2", "y", "y2", "r", and "r2" or the uppercase equivalents.
>
> *Display\_option* is an integer value corresponding to the display label option. Recognized values are 0 (hide) and 1 (display).
>
> ##### Return value
>
> The return value is 0 if successful or -1 if a problem was encountered.

### setDisplayTics

Sets the display tics option for a plot axis.

> ##### Usage
>
> *integer* DelaAxis.setDisplayTics(*Plot\_index, Axis\_id\_string, Display\_option*)
>
> ##### Arguments
>
> *Plot\_index* is an integer value corresponding to the index of a plot in the document contents.
>
> *Axis\_id\_string* is a unicode or C-style (ASCII) string corresponding to the axis. Recognized axis id strings are: "x", "x2", "y", "y2", "r", and "r2" or the uppercase equivalents.
>
> *Display\_option* is an integer value corresponding to the display tics option. Recognized values are 0 (hide) and 1 (display).
>
> ##### Return value
>
> The return value is 0 if successful or -1 if a problem was encountered.

### setDisplayTicLabels

Sets the display tics option for a plot axis.

> ##### Usage
>
> *integer* DelaAxis.setDisplayTicLabels(*Plot\_index, Axis\_id\_string, Display\_option*)
>
> ##### Arguments
>
> *Plot\_index* is an integer value corresponding to the index of a plot in the document contents.
>
> *Axis\_id\_string* is a unicode or C-style (ASCII) string corresponding to the axis. Recognized axis id strings are: "x", "x2", "y", "y2", "r", and "r2" or the uppercase equivalents.
>
> *Display\_option* is an integer value corresponding to the display tic labels option. Recognized values are 0 (hide), 1 (display inside), 2 (display outside), and 3 (display inside and outside).
>
> ##### Return value
>
> The return value is 0 if successful or -1 if a problem was encountered.

### setLogScale

Sets the log scale option for a plot axis.

> ##### Usage
>
> *integer* DelaAxis.setLogScale(*Plot\_index, Axis\_id\_string, Scale\_option*)
>
> ##### Arguments
>
> *Plot\_index* is an integer value corresponding to the index of a plot in the document contents.
>
> *Axis\_id\_string* is a unicode or C-style (ASCII) string corresponding to the axis. Recognized axis id strings are: "x", "x2", "y", "y2", "r", and "r2" or the uppercase equivalents.
>
> *Scale\_option* is an integer value corresponding to the log scale option. Recognized values are 0 (off) and 1 (on).
>
> ##### Return value
>
> The return value is 0 if successful or -1 if a problem was encountered.

### setMaximum

Sets the maximum for a plot axis.

> ##### Usage
>
> *integer* DelaAxis.setMaximum(*Plot\_index, Axis\_id\_string, Maximum*)
>
> ##### Arguments
>
> *Plot\_index* is an integer value corresponding to the index of a plot in the document contents.
>
> *Axis\_id\_string* is a unicode or C-style (ASCII) string corresponding to the axis. Recognized axis id strings are: "x", "x2", "y", "y2", "r", and "r2" or the uppercase equivalents.
>
> *Maximum* is a double value corresponding to the axis maximum.
>
> ##### Return value
>
> The return value is 0 if successful or -1 if a problem was encountered.

### setMinimum

Sets the minimum for a plot axis.

> ##### Usage
>
> *integer* DelaAxis.setMinimum(*Plot\_index, Axis\_id\_string, Minimum*)
>
> ##### Arguments
>
> *Plot\_index* is an integer value corresponding to the index of a plot in the document contents.
>
> *Axis\_id\_string* is a unicode or C-style (ASCII) string corresponding to the axis. Recognized axis id strings are: "x", "x2", "y", "y2", "r", and "r2" or the uppercase equivalents.
>
> *Minimum* is a double value corresponding to the axis minimum.
>
> ##### Return value
>
> The return value is 0 if successful or -1 if a problem was encountered.

### setMinorTicCount

Sets the number of minor tics for a plot axis.

> ##### Usage
>
> *integer* DelaAxis.setMinorTicCount(*Plot\_index, Axis\_id\_string, Tic\_count*)
>
> ##### Arguments
>
> *Plot\_index* is an integer value corresponding to the index of a plot in the document contents.
>
> *Axis\_id\_string* is a unicode or C-style (ASCII) string corresponding to the axis. Recognized axis id strings are: "x", "x2", "y", "y2", "r", and "r2" or the uppercase equivalents.
>
> *Tic\_count* is an integer value corresponding to the number of minor tics. Values less than 0 are ignored.
>
> ##### Return value
>
> The return value is 0 if successful or -1 if a problem was encountered.
