# Supplementary material for: Structural Organization and Dynamics of Homodimeric Cytohesin Family Arf GTPase Exchange Factors in Solution and on Membranes
Source: Structure. 2019 Dec 3;27(12):1782–1797.e7. doi: 10.1016/j.str.2019.09.007 (PMC6948192; doi:10.1016/j.str.2019.09.007)
Supplement: Data S1. Software, Related to the STAR Methods [file mmc3.zip › Software/Dela App Scripts/Dela.app/Contents/Resources/Dela.help/Contents/Resources/English.lproj/pgs/python_module_DelaData.html]

xml version="1.0" encoding="utf-8"?


Python Module DelaData


Home

Top
Contents


# DelaData python module

## Overview

The DelaData module provides methods related to data objects.

### Getting the number of elements in the data arrays

size

### Getting the unique identifying data label

label

### Getting unicde text descriptions

description

attributedDescription

xDescription

yDescription

errorDescription

rowDescription

### Getting data array values

xValues

yValues

errorValues

maskValues

### Setting unicode text descriptions

setDescription

setAttributedDescription

setXDescription

setYDescription

setErrorDescription

setRowDescription

### Setting data array values

setXValues

setYValues

setErrorValues

setMaskValues

### Creating new data objects

newData

### Creating a new linear model

newLinearModel

### Getting the instrument response

instrumentResponseIndex

### Setting the instrument response

setInstrumentResponse

### Getting the model data indices

modelDataIndices

### Setting the model data

setModelData

## Methods

### attributedDescription

Returns the attributed description of a data object.

> ##### Usage
>
> *unicode* DelaData.attributedDescription(*Data\_index\_or\_data\_label*)
>
> ##### Argument
>
> *Data\_index\_or\_data\_label* is either the index of the data object in the All Data list or the label of the data object.
>
> ##### Return value
>
> A *unicode* string corresponding to the text content of the attributed description if successful or an empty NULL-terminated unicode string if a problem was encountered.

### description

Returns the description of a data object.

> ##### Usage
>
> *unicode* DelaData.description(*Data\_index\_or\_data\_label*)
>
> ##### Argument
>
> *Data\_index\_or\_data\_label* is either the index of the data object in the All Data list or the label of the data object.
>
> ##### Return value
>
> A *unicode* string corresponding to the description if successful or an empty NULL-terminated unicode string if a problem was encountered.

### errorDescription

Returns the description of the error values in a data object.

> ##### Usage
>
> *unicode* DelaData.errorDescription(*Data\_index\_or\_data\_label*)
>
> ##### Argument
>
> *Data\_index\_or\_data\_label* is either the index of the data object in the All Data list or the label of the data object.
>
> ##### Return value
>
> A *unicode* string corresponding to error description if successful or an empty NULL-terminated unicode string if a problem was encountered.

### errorValues

Returns the y error values of a data object.

> ##### Usage
>
> *list* DelaData.errorValues(*Data\_index\_or\_data\_label*)
>
> ##### Argument
>
> *Data\_index\_or\_data\_label* is either the index of the data object in the All Data list or the label of the data object.
>
> ##### Return value
>
> A list of double values corresponding to the error values or [-1.0] if a problem was encountered.

### instrumentResponseIndex

Returns the index of the instrument response data object in the All data list.

> ##### Usage
>
> *unicode* DelaData.instrumentResponseIndex(*Data\_index\_or\_data\_label*)
>
> ##### Argument
>
> *Data\_index\_or\_data\_label* is either the index of the data object in the All Data list or the label of the data object.
>
> ##### Return value
>
> An integer value corresponding to the index of the instrument response if successful or -1 if a problem was encountered.

### label

Returns the label of a data object.

> ##### Usage
>
> *unicode* DelaData.label(*Data\_index\_or\_data\_label*)
>
> ##### Argument
>
> *Data\_index\_or\_data\_label* is either the index of the data object in the All Data list or the label of the data object.
>
> ##### Return value
>
> A *unicode* string corresponding to the label if successful or an empty NULL-terminated unicode string if a problem was encountered.

### maskValues

Returns the binary mask values of a data object.

> ##### Usage
>
> *list* DelaData.maskValues(*Data\_index\_or\_data\_label*)
>
> ##### Argument
>
> *Data\_index\_or\_data\_label* is either the index of the data object in the All Data list or the label of the data object.
>
> ##### Return value
>
> A list of integer values corresponding to the mask values if successful or [-1] if a problem was encountered.

### modelDataIndices

Returns the indices of a model data objects in the All Data list.

> ##### Usage
>
> *list* DelaData.maskValues(*Data\_index\_or\_data\_label*)
>
> ##### Argument
>
> *Data\_index\_or\_data\_label* is either the index of the data object in the All Data list or the label of the data object.
>
> ##### Return value
>
> A list of integer values corresponding to the model data indices if successful or [-1] if a problem was encountered.

### newData

Creates one or more new data objects.

> ##### Usage
>
> *integer* DelaData.newData(*Array\_size, Description, Number\_data\_objects, Sheet\_description*)
>
> ##### Arguments
>
> *Array\_size* is a required integer value that specifies the number of data points in the new data object(s).
>
> *Description* is either a unicode or C-style (ASCII) string. It is required if two or more arguments are supplied but can be omitted otherwise. *Description* is pre-processed for attribute tags.
>
> *Number\_data\_objects* is an integer value corresponding to the number of data objects to be created (must be > 0). It is required if three or more arguments are supplied.
>
> *Sheet\_description* is an optional argument that can be a unicode or C-style (ASCII) string corresponding to the sheet description. *Sheet\_description* is pre-processed for attribute tags.
>
> ##### Return value
>
> The return value is 0 if successful or -1 if a problem was encountered.
>
> ##### Discussion
>
> A new sheet is created and the new data object(s) are added to the new sheet and the All Data list. The x and y values are initialized to 0.0, the error valuesto 1.0, and the mask values to 0.

### newLinearModel

Creates a new linear model for a data object.

> ##### Usage
>
> *integer* DelaData.newLinearModel(*Data\_index\_or\_data\_label*, *Slope, Intercept, X\_minimum, X\_maximum*)
>
> ##### Arguments
>
> *Data\_index\_or\_data\_label* is either the index of the data object in the All Data list or the label of the data object.
>
> *Slope* is a double value corresponding to the slope.
>
> *Intercept* is a double value corresponding to the intercept.
>
> *X\_minimum* and *X\_maximum* are double values corresponding to the x limits for the linear model. These argument are optional; however, both must either be omitted or supplied.
>
> ##### Return value
>
> The return value is 0 if successful or -1 if a problem was encountered.
>
> ##### Discussion
>
> The model will not be automatically displayed in plots.

### rowDescription

Returns the description of a row corresponding to the index of a data point in a data object.

> ##### Usage
>
> *unicode* DelaData.rowDescription(*Data\_index\_or\_data\_label, Row\_index*)
>
> ##### Arguments
>
> *Data\_index\_or\_data\_label* is either the index of the data object in the All Data list or the label of the data object.
>
> *Row\_index* is the index of a data point in the data object.
>
> ##### Return value
>
> A *unicode* string corresponding to the row description if successful or an empty NULL-terminated unicode string if a problem was encountered.

### setAttributedDescription

Sets the text content (and attributes via attribute tags) of the attributed description of a data object.

> ##### Usage
>
> *integer* DelaData.setAttributedDescription(*Data\_index\_or\_data\_label*, *Description*)
>
> ##### Arguments
>
> *Data\_index\_or\_data\_label* is either the index of the data object in the All Data list or the label of the data object.
>
> *Description* is a unicode or C-style (ASCII) string corresponding to the new description. *Description* is pre-processed for attribute tags.
>
> ##### Return value
>
> The return value is 0 if successful or -1 if a problem was encountered.

### setDescription

Sets the description of a data object.

> ##### Usage
>
> *integer* DelaData.setDescription(*Data\_index\_or\_data\_label*, *Description*)
>
> ##### Arguments
>
> *Data\_index\_or\_data\_label* is either the index of the data object in the All Data list or the label of the data object.
>
> *Description* is a unicode or C-style (ASCII) string corresponding to the new description. *Description* is pre-processed for attribute tags.
>
> ##### Return value
>
> The return value is 0 if successful or -1 if a problem was encountered.

### setErrorDescription

Sets the description of the error values in a data object.

> ##### Usage
>
> *integer* DelaData.setErrorDescription(*Data\_index\_or\_data\_label*, *Description*)
>
> ##### Arguments
>
> *Data\_index\_or\_data\_label* is either the index of the data object in the All Data list or the label of the data object.
>
> *Description* is a unicode or C-style (ASCII) string corresponding to the new description for the error values. *Description* is pre-processed for attribute tags.
>
> ##### Return value
>
> The return value is 0 if successful or -1 if a problem was encountered.

### setErrorValues

Sets the error values in a data object.

> ##### Usage
>
> *integer* DelaData.setErrorValues(*Data\_index\_or\_data\_label*, *Values*)
>
> ##### Arguments
>
> *Data\_index\_or\_data\_label* is either the index of the data object in the All Data list or the label of the data object.
>
> *Values* is a list of double values corresponding to the new error values.
>
> ##### Return value
>
> The return value is 0 if successful or -1 if a problem was encountered.
>
> ##### Discussion
>
> If the number of elements in *Values* differs from the array size of the data object, memory is reallocated to the number of elements in *Values*.

### setInstrumentResponse

Sets the instrument response of a data object.

> ##### Usage
>
> *integer* DelaData.setInstrumentResponse(*Data\_index\_or\_data\_label*, *IR\_Data\_index\_or\_data\_label*)
>
> ##### Arguments
>
> *Data\_index\_or\_data\_label* is either the index of the data object in the All Data list or the label of the data object.
>
> *IR\_Data\_index\_or\_data\_label* is either the index of the instrument response data object in the All Data list or the label of the instrument response data object.
>
> ##### Return value
>
> The return value is 0 if successful or -1 if a problem was encountered.
>
> ##### Discussion
>
> Replaces any existing instrument response.

### setMaskValues

Sets the mask values in a data object.

> ##### Usage
>
> *integer* DelaData.setMaskValues(*Data\_index\_or\_data\_label*, *Values*)
>
> ##### Arguments
>
> *Data\_index\_or\_data\_label* is either the index of the data object in the All Data list or the label of the data object.
>
> *Values* is a list of integer values corresponding to the new mask values.
>
> ##### Return value
>
> The return value is 0 if successful or -1 if a problem was encountered.
>
> ##### Discussion
>
> The elements of Values are converted as 0 if 0 and 1 otherwise. If the number of elements in *Values* differs from the array size of the data object, memory is reallocated to the number of elements in *Values*.

### setModelData

Sets the model data objects.

> ##### Usage
>
> *integer* DelaData.setModelData(*Data\_index\_or\_data\_label*, *Model\_data\_indices*)
>
> ##### Arguments
>
> *Data\_index\_or\_data\_label* is either the index of the data object in the All Data list or the label of the data object.
>
> *Model\_data\_indices* is a list of integer values corresponding to the indices of the model data in the All Data list.
>
> ##### Return value
>
> The return value is 0 if successful or -1 if a problem was encountered.
>
> ##### Discussion
>
> Replaces the existing list of model data objects.

### setRowDescription

Sets the description of a row corresponding a data point in a data object.

> ##### Usage
>
> *integer* DelaData.setRowDescription(*Data\_index\_or\_data\_label*, *Row\_index, Description*)
>
> ##### Arguments
>
> *Data\_index\_or\_data\_label* is either the index of the data object in the All Data list or the label of the data object.
>
> *Row\_index* is the index of a data point in the data object.
>
> *Description* is a unicode or C-style (ASCII) string corresponding to the new description for the error values. *Description* is pre-processed for attribute tags.
>
> ##### Return value
>
> The return value is 0 if successful or -1 if a problem was encountered.

### setXDescription

Sets the description of the x values in a data object.

> ##### Usage
>
> *integer* DelaData.setXDescription(*Data\_index\_or\_data\_label*, *Description*)
>
> ##### Arguments
>
> *Data\_index\_or\_data\_label* is either the index of the data object in the All Data list or the label of the data object.
>
> *Description* is a unicode or C-style (ASCII) string corresponding to the new description for the x values. *Description* is pre-processed for attribute tags.
>
> ##### Return value
>
> The return value is 0 if successful or -1 if a problem was encountered.

### setXValues

Sets the x values in a data object.

> ##### Usage
>
> *integer* DelaData.setXValues(*Data\_index\_or\_data\_label*, *Values*)
>
> ##### Arguments
>
> *Data\_index\_or\_data\_label* is either the index of the data object in the All Data list or the label of the data object.
>
> *Values* is a list of double values corresponding to the new x values.
>
> ##### Return value
>
> The return value is 0 if successful or -1 if a problem was encountered.
>
> ##### Discussion
>
> If the number of elements in *Values* differs from the array size of the data object, memory is reallocated to the number of elements in *Values*.

### setYDescription

Sets the description of the y values in a data object.

> ##### Usage
>
> *integer* DelaData.setYDescription(*Data\_index\_or\_data\_label*, *Description*)
>
> ##### Arguments
>
> *Data\_index\_or\_data\_label* is either the index of the data object in the All Data list or the label of the data object.
>
> *Description* is a unicode or C-style (ASCII) string corresponding to the new description for the y values. *Description* is pre-processed for attribute tags.
>
> ##### Return value
>
> The return value is 0 if successful or -1 if a problem was encountered.

### setYValues

Sets the y values in a data object.

> ##### Usage
>
> *integer* DelaData.setYValues(*Data\_index\_or\_data\_label*, *Values*)
>
> ##### Arguments
>
> *Data\_index\_or\_data\_label* is either the index of the data object in the All Data list or the label of the data object.
>
> *Values* is a list of double values corresponding to the new y values.
>
> ##### Return value
>
> The return value is 0 if successful or -1 if a problem was encountered.
>
> ##### Discussion
>
> If the number of elements in *Values* differs from the array size of the data object, memory is reallocated to the number of elements in *Values*.

### size

Returns the array size of a data object.

> ##### Usage
>
> *integer* DelaData.size(*Data\_index\_or\_data\_label*)
>
> ##### Argument
>
> *Data\_index\_or\_data\_label* is either the index of the data object in the All Data list or the label of the data object.
>
> ##### Return value
>
> An integer value corresponding to the size if successful or -1 if a problem was encountered.

### xDescription

Returns the description of the x values in a data object.

> ##### Usage
>
> *unicode* DelaData.xDescription(*Data\_index\_or\_data\_label*)
>
> ##### Argument
>
> *Data\_index\_or\_data\_label* is either the index of the data object in the All Data list or the label of the data object.
>
> ##### Return value
>
> A *unicode* string corresponding to the x description if successful or an empty NULL-terminated unicode string if a problem was encountered.

### xValues

Returns the x values of a data object.

> ##### Usage
>
> *list* DelaData.xValues(*Data\_index\_or\_data\_label*)
>
> ##### Argument
>
> *Data\_index\_or\_data\_label* is either the index of the data object in the All Data list or the label of the data object.
>
> ##### Return value
>
> A list of double values corresponding to the x values or [-1.0] if a problem was encountered.

### yDescription

Returns the description of the y values in a data object.

> ##### Usage
>
> *unicode* DelaData.yDescription(*Data\_index\_or\_data\_label*)
>
> ##### Argument
>
> *Data\_index\_or\_data\_label* is either the index of the data object in the All Data list or the label of the data object.
>
> ##### Return value
>
> A *unicode* string corresponding to y description if successful or an empty NULL-terminated unicode string if a problem was encountered.

### yValues

Returns the y values of a data object.

> ##### Usage
>
> *list* DelaData.yValues(*Data\_index\_or\_data\_label*)
>
> ##### Argument
>
> *Data\_index\_or\_data\_label* is either the index of the data object in the All Data list or the label of the data object.
>
> ##### Return value
>
> A list of double values corresponding to the y values or [-1.0] if a problem was encountered.
