# Supplementary material for: Structural Organization and Dynamics of Homodimeric Cytohesin Family Arf GTPase Exchange Factors in Solution and on Membranes
Source: Structure. 2019 Dec 3;27(12):1782–1797.e7. doi: 10.1016/j.str.2019.09.007 (PMC6948192; doi:10.1016/j.str.2019.09.007)
Supplement: Data S1. Software, Related to the STAR Methods [file mmc3.zip › Software/Dela App Scripts/Dela.app/Contents/Resources/Dela.help/Contents/Resources/English.lproj/pgs/python_module_DelaDataSymbolFill.html]

xml version="1.0" encoding="utf-8"?


Python Module DelaDataSymbolFill


Home

Top
Contents


# DelaDataSymbolFill python module

## Overview

The DelaDataSymbolFill module provides methods related to the fill of a plot symbol.

### Getting and setting fill properties

type

color

setType

setColor

## Methods

### color

Returns the RGBA color elements of the color for the fill of a plot symbol.

> ##### Usage
>
> *integer* DelaDataSymbolFill.color(*Plot\_index, Data\_index\_or\_data\_label*)
>
> ##### Arguments
>
> *Plot\_index* is an integer value corresponding to the index of a plot in the document contents.
>
> *Data\_index\_or\_data\_label* is either the index of the data object in the All Data list or the label of the data object.
>
> ##### Return value
>
> A list of double values corresponding to the RGBA color elements or -1.0 if a problem was encountered. The color element list contains four double values: [red, green, blue, alpha]. The values range from 0.0-1.0.

### type

Returns the type option for the fill of a plot symbol.

> ##### Usage
>
> *integer* DelaDataSymbolFill.type(*Plot\_index, Data\_index\_or\_data\_label*)
>
> ##### Arguments
>
> *Plot\_index* is an integer value corresponding to the index of a plot in the document contents.
>
> *Data\_index\_or\_data\_label* is either the index of the data object in the All Data list or the label of the data object.
>
> ##### Return value
>
> An integer value corresponding to type option if successful or -1 if a problem was encountered.

### setColor

Sets the RGBA color elements of the color for the fill of a plot symbol.

> ##### Usage
>
> *integer* DelaDataSymbolFill.setColor(*Plot\_index, Data\_index\_or\_data\_label, RGBA\_components*)
>
> ##### Arguments
>
> *Plot\_index* is an integer value corresponding to the index of a plot in the document contents.
>
> *Data\_index\_or\_data\_label* is either the index of the data object in the All Data list or the label of the data object.
>
> *RGBA\_components* is a list of double values corresponding to RGBA color elements. The color element list should contain four double values: [red, green, blue, alpha]. The values range from 0.0-1.0.
>
> ##### Return value
>
> The return value is 0 if successful or -1 if a problem was encountered.

### setType

Sets the type option for the fill of a plot symbol.

> ##### Usage
>
> *integer* DelaDataSymbolFill.setType(*Plot\_index, Data\_index\_or\_data\_label, Option*)
>
> ##### Arguments
>
> *Plot\_index* is an integer value corresponding to the index of a plot in the document contents.
>
> *Data\_index\_or\_data\_label* is either the index of the data object in the All Data list or the label of the data object.
>
> *Option* is an integer value corresponding to the type option. Recognized values are 0 (none), 1 (continuous), and 2 (dash).
>
> ##### Return value
>
> The return value is 0 if successful or -1 if a problem was encountered.
