# Supplementary material for: Structural Organization and Dynamics of Homodimeric Cytohesin Family Arf GTPase Exchange Factors in Solution and on Membranes
Source: Structure. 2019 Dec 3;27(12):1782–1797.e7. doi: 10.1016/j.str.2019.09.007 (PMC6948192; doi:10.1016/j.str.2019.09.007)
Supplement: Data S1. Software, Related to the STAR Methods [file mmc3.zip › Software/Dela App Scripts/Dela.app/Contents/Resources/Dela.help/Contents/Resources/English.lproj/pgs/python_module_DelaDocument.html]

xml version="1.0" encoding="utf-8"?


Python Module DelaDocument


Home

Top
Contents


# DelaDocument python module

## Overview

The DelaDocument module provides methods related to document properties and user selections.

### Getting object counts

dataCount

sheetCount

plotCount

### Getting the index of a current object

currentSheetIndex

currentPlotIndex

### Getting indices of objects that are highlighted in the document contents

highlightedDataIndices

highlightedSheetIndices

highlightedPlotIndices

highlightedContentsDataIndices

### Getting indices of data objects associated with other objects

highlightedAllDataIndices

highlightedSheetsDataIndices

highlightedPlotsDataIndices

checkedAllDataIndices

allDataIndices

editDataIndices

### Selecting objects

selectPlot

### Actions

updateViews

## Methods

### allDataIndices

Returns a list of the indices of the data in the All Data list.

> ##### Usage
>
> *list* DelaDocument.allDataIndices()
>
> ##### Return value
>
> A list of integer values corresponding to the indices if successful or [-1] if a problem was encountered.

### checkedAllDataIndices

Returns a list of the indices of the checked data in the All Data list.

> ##### Usage
>
> *list* DelaDocument.checkedAllDataIndices()
>
> ##### Return value
>
> A list of integer values corresponding to the indices if successful or [-1] if a problem was encountered.

### currentPlotIndex

Returns the index of the most recently selected plot.

> ##### Usage
>
> *integer* DelaDocument.currentPlotIndex()
>
> ##### Return value
>
> An integer value corresponding to the index if successful or -1 if a problem was encountered.

### currentSheetIndex

Returns the index of the most recently selected sheet.

> ##### Usage
>
> *integer* DelaDocument.currentSheetIndex()
>
> ##### Return value
>
> An integer value corresponding to the index if successful or -1 if a problem was encountered.

### dataCount

Returns the total number of data objects.

> ##### Usage
>
> *integer* DelaDocument.dataCount()
>
> ##### Return value
>
> An integer value corresponding to the number of data objects if successful or -1 if a problem was encountered.

### editDataIndices

Returns a list of the indices of the currently edited data.

> ##### Usage
>
> *list* DelaDocument.editDataIndices()
>
> ##### Return value
>
> A list of integer values corresponding to the indices if successful or [-1] if a problem was encountered.

### highlightedAllDataIndices

Returns a list of the indices of the highlighted data in the All Data list.

> ##### Usage
>
> *list* DelaDocument.highlightedAllDataIndices()
>
> ##### Return value
>
> A list of integer values corresponding to the indices if successful or [-1] if a problem was encountered.

### highlightedContentsDataIndices

Returns a list of the indices of the data in the highlighted objects (data, sheets, and plots) in the document contents.

> ##### Usage
>
> *list* DelaDocument.highlightedContentsDataIndices()
>
> ##### Return value
>
> A list of integer values corresponding to the indices if successful or [-1] if a problem was encountered.

### highlightedDataIndices

Returns a list of the indices of the highlighted data in the document contents.

> ##### Usage
>
> *list* DelaDocument.highlightedDataIndices()
>
> ##### Return value
>
> A list of integer values corresponding to the indices if successful or [-1] if a problem was encountered.

### highlightedPlotIndices

Returns a list of the indices of the highlighted plots in the document contents.

> ##### Usage
>
> *list* DelaDocument.highlightedPlotIndices()
>
> ##### Return value
>
> A list of integer values corresponding to the indices if successful or [-1] if a problem was encountered.

### highlightedPlotsDataIndices

Returns a list of the indices of the data in the highlighted plots in the document contents.

> ##### Usage
>
> *list* DelaDocument.highlightedPlotsDataIndices()
>
> ##### Return value
>
> A list of integer values corresponding to the indices if successful or [-1] if a problem was encountered.

### highlightedSheetIndices

Returns a list of the indices of the highlighted sheets in the document contents.

> ##### Usage
>
> *list* DelaDocument.highlightedSheetIndices()
>
> ##### Return value
>
> A list of integer values corresponding to indices if successful or [-1] if a problem was encountered.

### highlightedSheetsDataIndices

Returns a list of the indices of the data in the highlighted sheets in the document contents.

> ##### Usage
>
> *list* DelaDocument.highlightedSheetsDataIndices()
>
> ##### Return value
>
> A list of integer values corresponding to the indices if successful or [-1] if a problem was encountered.

### plotCount

Returns the total number of plots.

> ##### Usage
>
> *integer* DelaDocument.plotCount()
>
> ##### Return value
>
> An integer value corresponding to the number of plots if successful or -1 if a problem was encountered.

### selectPlot

Selects a plot in the document contents.

> ##### Usage
>
> *integer* DelaDocument.selectPlot(*Plot\_index*)
>
> ##### Argument
>
> *Plot\_index* is an integer value corresponding to the index of a plot in the document contents.
>
> ##### Return value
>
> The return value is 0 if successful or -1 if a problem was encountered.

### sheetCount

Returns the total number of sheets.

> ##### Usage
>
> *integer* DelaDocument.sheetCount()
>
> ##### Return value
>
> An integer value corresponding to the number of sheets if successful or -1 if a problem was encountered.

### updateViews

Updates the document views to reflect changes resulting from script execution.

> ##### Usage
>
> *integer* DelaDocument.updateViews()
>
> ##### Return value
>
> The return value is 0 if successful or -1 if a problem was encountered.
