# Supplementary material for: Structural Organization and Dynamics of Homodimeric Cytohesin Family Arf GTPase Exchange Factors in Solution and on Membranes
Source: Structure. 2019 Dec 3;27(12):1782–1797.e7. doi: 10.1016/j.str.2019.09.007 (PMC6948192; doi:10.1016/j.str.2019.09.007)
Supplement: Data S1. Software, Related to the STAR Methods [file mmc3.zip › Software/Dela App Scripts/Dela.app/Contents/Resources/Dela.help/Contents/Resources/English.lproj/pgs/python_module_DelaError.html]

xml version="1.0" encoding="utf-8"?


Python Module DelaError


Home

Top
Contents


# DelaError python module

## Overview

The DelaError module provides methods related to the script execution errors.

### Displaying an error message

write

### Terminating script execution

quit

## Methods

### quit

Terminates script execution and optionally displays an error message in bold red font.

> ##### Usage
>
> *integer* DelaError.quit(*Error\_message*)
>
> ##### Argument
>
> *Error\_message* is an optional unicode or C-style (ASCII) string.
>
> ##### Return value
>
> The return value is moot since script execution is terminated.
>
> ##### Discussion
>
> *String* is preprocessed for embedded tags specifying unicode characters, fonts, character attributes, or paragraph styles.

### write

Displays an error message to the user interface in bold red font.

> ##### Usage
>
> *integer* DelaError.write(*Error\_message*)
>
> ##### Argument
>
> *Error\_message* is a unicode or C-style (ASCII) string.
>
> ##### Return value
>
> The return value is 0 if successful or -1 if a problem was encountered.
>
> ##### **Discussion**
>
> *String* is preprocessed for embedded tags specifying unicode characters, fonts, character attributes, or paragraph styles. This method is used by the embedded python interpretor for messages to stdout.
