# Supplementary material for: Structural Organization and Dynamics of Homodimeric Cytohesin Family Arf GTPase Exchange Factors in Solution and on Membranes
Source: Structure. 2019 Dec 3;27(12):1782–1797.e7. doi: 10.1016/j.str.2019.09.007 (PMC6948192; doi:10.1016/j.str.2019.09.007)
Supplement: Data S1. Software, Related to the STAR Methods [file mmc3.zip › Software/Dela App Scripts/Dela.app/Contents/Resources/Dela.help/Contents/Resources/English.lproj/pgs/python_module_DelaMajorTics.html]

xml version="1.0" encoding="utf-8"?


Python Module DelaMajorTics


Home

Top
Contents


# DelaMajorTics python module

## Overview

The DelaMajorTics module provides methods related to the major tics of plot axes.

### Getting and setting display options

display

displayLabels

labelSkipCount

setDisplay

setDisplayLabels

setLabelSkipCount

### Getting and setting label offsets

labelOffset

setLabelOffset

### Getting and setting auto control parameters

autoFirstValue

autoIncrement

autoExponent

setAutoFirstValue

setAutoIncrement

setAutoExponent

### Getting and setting manual paramaters

firstValue

increment

exponent

length

setFirstValue

setIncrement

setExponent

setLength

## Methods

### autoExponent

Returns the auto exponent option for the major tics of a plot axis.

> ##### Usage
>
> *integer* DelaMajorTics.autoExponent(*Plot\_index, Axis\_id\_string*)
>
> ##### Arguments
>
> *Plot\_index* is an integer value corresponding to the index of a plot in the document contents.
>
> *Axis\_id\_string* is a unicode or C-style (ASCII) string corresponding to the axis. Recognized axis id strings are: "x", "x2", "y", "y2", "r", and "r2" or the uppercase equivalents.
>
> ##### Return value
>
> An integer value corresponding to auto exponent option if successful or -1 if a problem was encountered.

### autoFirstValue

Returns the auto first value option for the major tics of a plot axis.

> ##### Usage
>
> *integer* DelaMajorTics.autoFirstValue(*Plot\_index, Axis\_id\_string*)
>
> ##### Arguments
>
> *Plot\_index* is an integer value corresponding to the index of a plot in the document contents.
>
> *Axis\_id\_string* is a unicode or C-style (ASCII) string corresponding to the axis. Recognized axis id strings are: "x", "x2", "y", "y2", "r", and "r2" or the uppercase equivalents.
>
> ##### Return value
>
> An integer value corresponding to auto first value option if successful or -1 if a problem was encountered.

### autoIncrement

Returns the auto increment option for the major tics of a plot axis.

> ##### Usage
>
> *integer* DelaMajorTics.autoIncrement(*Plot\_index, Axis\_id\_string*)
>
> ##### Arguments
>
> *Plot\_index* is an integer value corresponding to the index of a plot in the document contents.
>
> *Axis\_id\_string* is a unicode or C-style (ASCII) string corresponding to the axis. Recognized axis id strings are: "x", "x2", "y", "y2", "r", and "r2" or the uppercase equivalents.
>
> ##### Return value
>
> An integer value corresponding to auto increment option if successful or -1 if a problem was encountered.

### display

Returns the display option for the major tics of a plot axis.

> ##### Usage
>
> *integer* DelaMajorTics.display(*Plot\_index, Axis\_id\_string*)
>
> ##### Arguments
>
> *Plot\_index* is an integer value corresponding to the index of a plot in the document contents.
>
> *Axis\_id\_string* is a unicode or C-style (ASCII) string corresponding to the axis. Recognized axis id strings are: "x", "x2", "y", "y2", "r", and "r2" or the uppercase equivalents.
>
> ##### Return value
>
> An integer value corresponding to the display option if successful or -1 if a problem was encountered.

### displayLabels

Returns the display labels option for the major tics of a plot axis.

> ##### Usage
>
> *integer* DelaMajorTics.displayLabels(*Plot\_index, Axis\_id\_string*)
>
> ##### Arguments
>
> *Plot\_index* is an integer value corresponding to the index of a plot in the document contents.
>
> *Axis\_id\_string* is a unicode or C-style (ASCII) string corresponding to the axis. Recognized axis id strings are: "x", "x2", "y", "y2", "r", and "r2" or the uppercase equivalents.
>
> ##### Return value
>
> An integer value corresponding to the display labels option if successful or -1 if a problem was encountered.

### exponent

Returns the exponent for the major tics of a plot axis.

> ##### Usage
>
> *integer* DelaMajorTics.exponent(*Plot\_index, Axis\_id\_string*)
>
> ##### Arguments
>
> *Plot\_index* is an integer value corresponding to the index of a plot in the document contents.
>
> *Axis\_id\_string* is a unicode or C-style (ASCII) string corresponding to the axis. Recognized axis id strings are: "x", "x2", "y", "y2", "r", and "r2" or the uppercase equivalents.
>
> ##### Return value
>
> A double value corresponding to the exponent if successful or -1 if a problem was encountered.

### firstValue

Returns the first value for the major tics of a plot axis.

> ##### Usage
>
> *integer* DelaMajorTics.firstValue(*Plot\_index, Axis\_id\_string*)
>
> ##### Arguments
>
> *Plot\_index* is an integer value corresponding to the index of a plot in the document contents.
>
> *Axis\_id\_string* is a unicode or C-style (ASCII) string corresponding to the axis. Recognized axis id strings are: "x", "x2", "y", "y2", "r", and "r2" or the uppercase equivalents.
>
> ##### Return value
>
> A double value corresponding to the first value if successful or -1 if a problem was encountered.

### increment

Returns the increment for the major tics of a plot axis.

> ##### Usage
>
> *integer* DelaMajorTics.increment(*Plot\_index, Axis\_id\_string*)
>
> ##### Arguments
>
> *Plot\_index* is an integer value corresponding to the index of a plot in the document contents.
>
> *Axis\_id\_string* is a unicode or C-style (ASCII) string corresponding to the axis. Recognized axis id strings are: "x", "x2", "y", "y2", "r", and "r2" or the uppercase equivalents.
>
> ##### Return value
>
> A double value corresponding to the increment if successful or -1 if a problem was encountered.

### labelSkipCount

Returns the label skip count for the major tics of a plot axis.

> ##### Usage
>
> *integer* DelaMajorTics.labelSkipCount(*Plot\_index, Axis\_id\_string*)
>
> ##### Arguments
>
> *Plot\_index* is an integer value corresponding to the index of a plot in the document contents.
>
> *Axis\_id\_string* is a unicode or C-style (ASCII) string corresponding to the axis. Recognized axis id strings are: "x", "x2", "y", "y2", "r", and "r2" or the uppercase equivalents.
>
> ##### Return value
>
> An integer value corresponding to the label skip count if successful or -1 if a problem was encountered.

### labelOffset

Returns the label offset for the major tics of a plot axis.

> ##### Usage
>
> *integer* DelaMajorTics.labelOffset(*Plot\_index, Axis\_id\_string*)
>
> ##### Arguments
>
> *Plot\_index* is an integer value corresponding to the index of a plot in the document contents.
>
> *Axis\_id\_string* is a unicode or C-style (ASCII) string corresponding to the axis. Recognized axis id strings are: "x", "x2", "y", "y2", "r", and "r2" or the uppercase equivalents.
>
> ##### Return value
>
> A list of double values corresponding to the x and y values of the label offset if successful or [-1.0] if a problem was encountered. Items 0 and 1 contain the x and y values of the label offset, respectively.

### length

Returns the length for the major tics of a plot axis.

> ##### Usage
>
> *integer* DelaMajorTics.length(*Plot\_index, Axis\_id\_string*)
>
> ##### Arguments
>
> *Plot\_index* is an integer value corresponding to the index of a plot in the document contents.
>
> *Axis\_id\_string* is a unicode or C-style (ASCII) string corresponding to the axis. Recognized axis id strings are: "x", "x2", "y", "y2", "r", and "r2" or the uppercase equivalents.
>
> ##### Return value
>
> A double value corresponding to the length if successful or -1 if a problem was encountered.

### setAutoExponent

Sets the auto exponent option for the major tics of a plot axis.

> ##### Usage
>
> *integer* DelaMajorTics.setAutoExponent(*Plot\_index, Axis\_id\_string, Option*)
>
> ##### Arguments
>
> *Plot\_index* is an integer value corresponding to the index of a plot in the document contents.
>
> *Axis\_id\_string* is a unicode or C-style (ASCII) string corresponding to the axis. Recognized axis id strings are: "x", "x2", "y", "y2", "r", and "r2" or the uppercase equivalents.
>
> *Option* is an integer value corresponding to the auto exponent option. Recognized values are 0 (off) and 1 (on).
>
> ##### Return value
>
> The return value is 0 if successful or -1 if a problem was encountered.

### setAutoFirstValue

Sets the auto first value option for the major tics of a plot axis.

> ##### Usage
>
> *integer* DelaMajorTics.setAutoFirstValue(*Plot\_index, Axis\_id\_string, Option*)
>
> ##### Arguments
>
> *Plot\_index* is an integer value corresponding to the index of a plot in the document contents.
>
> *Axis\_id\_string* is a unicode or C-style (ASCII) string corresponding to the axis. Recognized axis id strings are: "x", "x2", "y", "y2", "r", and "r2" or the uppercase equivalents.
>
> *Option* is an integer value corresponding to the auto first value option. Recognized values are 0 (off) and 1 (on).
>
> ##### Return value
>
> The return value is 0 if successful or -1 if a problem was encountered.

### setAutoIncrement

Sets the auto increment option for the major tics of a plot axis.

> ##### Usage
>
> *integer* DelaMajorTics.setAutoIncrement(*Plot\_index, Axis\_id\_string, Option*)
>
> ##### Arguments
>
> *Plot\_index* is an integer value corresponding to the index of a plot in the document contents.
>
> *Axis\_id\_string* is a unicode or C-style (ASCII) string corresponding to the axis. Recognized axis id strings are: "x", "x2", "y", "y2", "r", and "r2" or the uppercase equivalents.
>
> *Option* is an integer value corresponding to the auto increment option. Recognized values are 0 (off) and 1 (on).
>
> ##### Return value
>
> The return value is 0 if successful or -1 if a problem was encountered.

### setDisplay

Sets the display option the major tics of a plot axis.

> ##### Usage
>
> *integer* DelaMajorTics.setDisplay(*Plot\_index, Axis\_id\_string, Option*)
>
> ##### Arguments
>
> *Plot\_index* is an integer value corresponding to the index of a plot in the document contents.
>
> *Axis\_id\_string* is a unicode or C-style (ASCII) string corresponding to the axis. Recognized axis id strings are: "x", "x2", "y", "y2", "r", and "r2" or the uppercase equivalents.
>
> *Option* is an integer value corresponding to the display option. Recognized values are 0 (hide) and 1 (display).
>
> ##### Return value
>
> The return value is 0 if successful or -1 if a problem was encountered.

### setDisplayLabels

Sets the display labels option the major tics of a plot axis.

> ##### Usage
>
> *integer* DelaMajorTics.setDisplayLabels(*Plot\_index, Axis\_id\_string, Option*)
>
> ##### Arguments
>
> *Plot\_index* is an integer value corresponding to the index of a plot in the document contents.
>
> *Axis\_id\_string* is a unicode or C-style (ASCII) string corresponding to the axis. Recognized axis id strings are: "x", "x2", "y", "y2", "r", and "r2" or the uppercase equivalents.
>
> *Option* is an integer value corresponding to the display labels option. Recognized values are 0 (hide) and 1 (display).
>
> ##### Return value
>
> The return value is 0 if successful or -1 if a problem was encountered.

### setExponent

Sets the exponent for the major tics of a plot axis.

> ##### Usage
>
> *integer* DelaMajorTics.setExponent(*Plot\_index, Axis\_id\_string, Value*)
>
> ##### Arguments
>
> *Plot\_index* is an integer value corresponding to the index of a plot in the document contents.
>
> *Axis\_id\_string* is a unicode or C-style (ASCII) string corresponding to the axis. Recognized axis id strings are: "x", "x2", "y", "y2", "r", and "r2" or the uppercase equivalents.
>
> *Value* is a double value corresponding to the exponent.
>
> ##### Return value
>
> The return value is 0 if successful or -1 if a problem was encountered.

### setFirstValue

Sets the first value for the major tics of a plot axis.

> ##### Usage
>
> *integer* DelaMajorTics.setFirstValue(*Plot\_index, Axis\_id\_string, Value*)
>
> ##### Arguments
>
> *Plot\_index* is an integer value corresponding to the index of a plot in the document contents.
>
> *Axis\_id\_string* is a unicode or C-style (ASCII) string corresponding to the axis. Recognized axis id strings are: "x", "x2", "y", "y2", "r", and "r2" or the uppercase equivalents.
>
> *Value* is a double value corresponding to the first value.
>
> ##### Return value
>
> The return value is 0 if successful or -1 if a problem was encountered.

### setIncrement

Sets the increment for the major tics of a plot axis.

> ##### Usage
>
> *integer* DelaMajorTics.setIncrement(*Plot\_index, Axis\_id\_string, Value*)
>
> ##### Arguments
>
> *Plot\_index* is an integer value corresponding to the index of a plot in the document contents.
>
> *Axis\_id\_string* is a unicode or C-style (ASCII) string corresponding to the axis. Recognized axis id strings are: "x", "x2", "y", "y2", "r", and "r2" or the uppercase equivalents.
>
> *Value* is a double value corresponding to the increment.
>
> ##### Return value
>
> The return value is 0 if successful or -1 if a problem was encountered.

### setLabelSkipCount

Sets the label skip count for the major tics of a plot axis.

> ##### Usage
>
> *integer* DelaMajorTics.setLabelSkipCount(*Plot\_index, Axis\_id\_string, Count*)
>
> ##### Arguments
>
> *Plot\_index* is an integer value corresponding to the index of a plot in the document contents.
>
> *Axis\_id\_string* is a unicode or C-style (ASCII) string corresponding to the axis. Recognized axis id strings are: "x", "x2", "y", "y2", "r", and "r2" or the uppercase equivalents.
>
> *Count* is an integer value corresponding to the label skip count. Recognized values are greater than or equal to 0.
>
> ##### Return value
>
> The return value is 0 if successful or -1 if a problem was encountered.

### setLabelOffset

Sets the label offset for the major tics of a plot axis.

> ##### Usage
>
> *integer* DelaMajorTics.setLabelOffset(*Plot\_index, Axis\_id\_string, Offset*)
>
> ##### Arguments
>
> *Plot\_index* is an integer value corresponding to the index of a plot in the document contents.
>
> *Axis\_id\_string* is a unicode or C-style (ASCII) string corresponding to the axis. Recognized axis id strings are: "x", "x2", "y", "y2", "r", and "r2" or the uppercase equivalents.
>
> *Offset* is a list of doubles values corresponding to x and y values of the label offset. Items 0 and 1 of the list should contain the x and y values, respectively.
>
> ##### Return value
>
> The return value is 0 if successful or -1 if a problem was encountered.

### setLength

Sets the length for the major tics of a plot axis.

> ##### Usage
>
> *integer* DelaMajorTics.setLength(*Plot\_index, Axis\_id\_string, Value*)
>
> ##### Arguments
>
> *Plot\_index* is an integer value corresponding to the index of a plot in the document contents.
>
> *Axis\_id\_string* is a unicode or C-style (ASCII) string corresponding to the axis. Recognized axis id strings are: "x", "x2", "y", "y2", "r", and "r2" or the uppercase equivalents.
>
> *Value* is a double value corresponding to the length in pixels.
>
> ##### Return value
>
> The return value is 0 if successful or -1 if a problem was encountered.
