# Supplementary material for: Structural Organization and Dynamics of Homodimeric Cytohesin Family Arf GTPase Exchange Factors in Solution and on Membranes
Source: Structure. 2019 Dec 3;27(12):1782–1797.e7. doi: 10.1016/j.str.2019.09.007 (PMC6948192; doi:10.1016/j.str.2019.09.007)
Supplement: Data S1. Software, Related to the STAR Methods [file mmc3.zip › Software/Dela App Scripts/Dela.app/Contents/Resources/Dela.help/Contents/Resources/English.lproj/pgs/python_module_DelaMaximumLikelihood.html]

xml version="1.0" encoding="utf-8"?


Python Module DelaMaximumLikelihood


Home

Top
Contents


# DelaML python module

## Overview

The DelaML module provides methods related to maximum likelihood analyses.

### Fitting a model

linearFit

## Methods

### linearFit

Returns the results of a linear least squares to x and y data pairs.

> ##### Usage
>
> *linear\_model\_list* DelaML.linearFit(*X\_values, Y\_values*)
>
> ##### Arguments
>
> *X\_values* and *Y\_values* are lists of double values corresponding to x and y data pairs, respectively.
>
> ##### Return value
>
> A list containing the slope, intercept, and R-squared values for the linear least squares fit to the x and y data pairs if successful or [-1] if a problem was encountered.
