# Supplementary material for: Structural Organization and Dynamics of Homodimeric Cytohesin Family Arf GTPase Exchange Factors in Solution and on Membranes
Source: Structure. 2019 Dec 3;27(12):1782–1797.e7. doi: 10.1016/j.str.2019.09.007 (PMC6948192; doi:10.1016/j.str.2019.09.007)
Supplement: Data S1. Software, Related to the STAR Methods [file mmc3.zip › Software/Dela App Scripts/Dela.app/Contents/Resources/Dela.help/Contents/Resources/English.lproj/pgs/python_module_DelaMinorTics.html]

xml version="1.0" encoding="utf-8"?


Python Module DelaMinorTics


Home

Top
Contents


# DelaMinorTics python module

## Overview

The DelaMinorTics module provides methods related to the minor tics of plot axes.

### Getting and setting the count

count

setCount

### Getting and setting the length

length

setLength

## Methods

### count

Returns the count for the minor tics of a plot axis.

> ##### Usage
>
> *integer* DelaMinorTics.count(*Plot\_index, Axis\_id\_string*)
>
> ##### Arguments
>
> *Plot\_index* is an integer value corresponding to the index of a plot in the document contents.
>
> *Axis\_id\_string* is a unicode or C-style (ASCII) string corresponding to the axis. Recognized axis id strings are: "x", "x2", "y", "y2", "r", and "r2" or the uppercase equivalents.
>
> ##### Return value
>
> An integer value corresponding to the count if successful or -1 if a problem was encountered.

### length

Returns the length for the minor tics of a plot axis.

> ##### Usage
>
> *integer* DelaMinorTics.length(*Plot\_index, Axis\_id\_string*)
>
> ##### Arguments
>
> *Plot\_index* is an integer value corresponding to the index of a plot in the document contents.
>
> *Axis\_id\_string* is a unicode or C-style (ASCII) string corresponding to the axis. Recognized axis id strings are: "x", "x2", "y", "y2", "r", and "r2" or the uppercase equivalents.
>
> ##### Return value
>
> A double value corresponding to the length if successful or -1 if a problem was encountered.

### setCount

Sets the count for the minor tics of a plot axis.

> ##### Usage
>
> *integer* DelaMinorTics.setCount(*Plot\_index, Axis\_id\_string, Count*)
>
> ##### Arguments
>
> *Plot\_index* is an integer value corresponding to the index of a plot in the document contents.
>
> *Axis\_id\_string* is a unicode or C-style (ASCII) string corresponding to the axis. Recognized axis id strings are: "x", "x2", "y", "y2", "r", and "r2" or the uppercase equivalents.
>
> *Count* is an integer value corresponding to the count. A value greater than or equal to 0 is expectecd.
>
> ##### Return value
>
> The return value is 0 if successful or -1 if a problem was encountered.

### setLength

Sets the length for the minor tics of a plot axis.

> ##### Usage
>
> *integer* DelaMinorTics.setLength(*Plot\_index, Axis\_id\_string, Value*)
>
> ##### Arguments
>
> *Plot\_index* is an integer value corresponding to the index of a plot in the document contents.
>
> *Axis\_id\_string* is a unicode or C-style (ASCII) string corresponding to the axis. Recognized axis id strings are: "x", "x2", "y", "y2", "r", and "r2" or the uppercase equivalents.
>
> *Value* is a double value corresponding to the length in pixels.
>
> ##### Return value
>
> The return value is 0 if successful or -1 if a problem was encountered.
