# Supplementary material for: Structural Organization and Dynamics of Homodimeric Cytohesin Family Arf GTPase Exchange Factors in Solution and on Membranes
Source: Structure. 2019 Dec 3;27(12):1782–1797.e7. doi: 10.1016/j.str.2019.09.007 (PMC6948192; doi:10.1016/j.str.2019.09.007)
Supplement: Data S1. Software, Related to the STAR Methods [file mmc3.zip › Software/Dela App Scripts/Dela.app/Contents/Resources/Dela.help/Contents/Resources/English.lproj/pgs/python_module_DelaModelLineStroke.html]

xml version="1.0" encoding="utf-8"?


Python Module DelaModelLineStroke


Home

Top
Contents


# DelaModelLineStroke python module

## Overview

The DelaModelLineStroke module provides methods related to the stroke of a plot model line.

### Getting and setting stroke properties

type

join

cap

miterLimit

width

color

setType

setJoin

setCap

setMiterLimit

setWidth

setColor

## Methods

### cap

Returns the cap option for the stroke of a plot model line.

> ##### Usage
>
> *integer* DelaModelLineStroke.cap(*Plot\_index, Data\_index\_or\_data\_label, Model\_index*)
>
> ##### Arguments
>
> *Plot\_index* is an integer value corresponding to the index of a plot in the document contents.
>
> *Data\_index\_or\_data\_label* is either the index of the data object in the All Data list or the label of the data object.
>
> *Model\_index* is the index of a model in the data object.
>
> ##### Return value
>
> An integer value corresponding to cap option if successful or -1 if a problem was encountered.

### color

Returns the RGBA color elements of the color for the stroke of a plot model line.

> ##### Usage
>
> *integer* DelaModelLineStroke.color(*Plot\_index, Data\_index\_or\_data\_label, Model\_index*)
>
> ##### Arguments
>
> *Plot\_index* is an integer value corresponding to the index of a plot in the document contents.
>
> *Data\_index\_or\_data\_label* is either the index of the data object in the All Data list or the label of the data object.
>
> *Model\_index* is the index of a model in the data object.
>
> ##### Return value
>
> A list of double values corresponding to the RGBA color elements or -1.0 if a problem was encountered. The color element list contains four double values: [red, green, blue, alpha]. The values range from 0.0-1.0.

### join

Returns the join option for the stroke of a plot model line.

> ##### Usage
>
> *integer* DelaModelLineStroke.join(*Plot\_index, Data\_index\_or\_data\_label**, Model\_index*)
>
> ##### Arguments
>
> *Plot\_index* is an integer value corresponding to the index of a plot in the document contents.
>
> *Data\_index\_or\_data\_label* is either the index of the data object in the All Data list or the label of the data object.
>
> *Model\_index* is the index of a model in the data object.
>
> ##### Return value
>
> An integer value corresponding to join option if successful or -1 if a problem was encountered.

### miterLimit

Returns the miter limit for the stroke of a plot model line.

> ##### Usage
>
> *integer* DelaModelLineStroke.miterLimit(*Plot\_index, Data\_index\_or\_data\_label**, Model\_index*)
>
> ##### Arguments
>
> *Plot\_index* is an integer value corresponding to the index of a plot in the document contents.
>
> *Data\_index\_or\_data\_label* is either the index of the data object in the All Data list or the label of the data object.
>
> *Model\_index* is the index of a model in the data object.
>
> ##### Return value
>
> A double value corresponding to the miter limit if successful or -1.0 if a problem was encountered.

### type

Returns the type option for the stroke of a plot model line.

> ##### Usage
>
> *integer* DelaModelLineStroke.type(*Plot\_index, Data\_index\_or\_data\_label**, Model\_index*)
>
> ##### Arguments
>
> *Plot\_index* is an integer value corresponding to the index of a plot in the document contents.
>
> *Data\_index\_or\_data\_label* is either the index of the data object in the All Data list or the label of the data object.
>
> *Model\_index* is the index of a model in the data object.
>
> ##### Return value
>
> An integer value corresponding to type option if successful or -1 if a problem was encountered.

### width

Returns the width for the stroke of a plot model line.

> ##### Usage
>
> *integer* DelaModelLineStroke.width(*Plot\_index, Data\_index\_or\_data\_label, Model\_index*)
>
> ##### Arguments
>
> *Plot\_index* is an integer value corresponding to the index of a plot in the document contents.
>
> *Data\_index\_or\_data\_label* is either the index of the data object in the All Data list or the label of the data object.
>
> *Model\_index* is the index of a model in the data object.
>
> ##### Return value
>
> A double value corresponding to the width if successful or -1.0 if a problem was encountered.

### setCap

Sets the cap option for the stroke of a plot model line.

> ##### Usage
>
> *integer* DelaModelLineStroke.setCap(*Plot\_index, Data\_index\_or\_data\_label, Model\_index, Option*)
>
> ##### Arguments
>
> *Plot\_index* is an integer value corresponding to the index of a plot in the document contents.
>
> *Data\_index\_or\_data\_label* is either the index of the data object in the All Data list or the label of the data object.
>
> *Model\_index* is the index of a model in the data object.
>
> *Option* is an integer value corresponding to the cap option. Recognized values are 0 (butt), 1 (round), and 2 (square).
>
> ##### Return value
>
> The return value is 0 if successful or -1 if a problem was encountered.

### setColor

Sets the RGBA color elements of the color for the stroke of a plot model line.

> ##### Usage
>
> *integer* DelaModelLineStroke.setColor(*Plot\_index, Data\_index\_or\_data\_label, Model\_index, RGBA\_components*)
>
> ##### Arguments
>
> *Plot\_index* is an integer value corresponding to the index of a plot in the document contents.
>
> *Data\_index\_or\_data\_label* is either the index of the data object in the All Data list or the label of the data object.
>
> *Model\_index* is the index of a model in the data object.
>
> *RGBA\_components* is a list of double values corresponding to RGBA color elements. The color element list should contain four double values: [red, green, blue, alpha]. The values range from 0.0-1.0.
>
> ##### Return value
>
> The return value is 0 if successful or -1 if a problem was encountered.

### setJoin

Sets the join option for the stroke of a plot model line.

> ##### Usage
>
> *integer* DelaModelLineStroke.setJoin(*Plot\_index, Data\_index\_or\_data\_label, Model\_index, Option*)
>
> ##### Arguments
>
> *Plot\_index* is an integer value corresponding to the index of a plot in the document contents.
>
> *Data\_index\_or\_data\_label* is either the index of the data object in the All Data list or the label of the data object.
>
> *Model\_index* is the index of a model in the data object.
>
> *Option* is an integer value corresponding to the join option. Recognized values are 0 (miter; uses miter limit), 1 (round), and 2 (bevel).
>
> ##### Return value
>
> The return value is 0 if successful or -1 if a problem was encountered.

### setMiterLimit

Sets the miter limit for the stroke of a plot model line.

> ##### Usage
>
> *integer* DelaModelLineStroke.setMiterLimit(*Plot\_index, Data\_index\_or\_data\_label, Model\_index, Limit*)
>
> ##### Arguments
>
> *Plot\_index* is an integer value corresponding to the index of a plot in the document contents.
>
> *Data\_index\_or\_data\_label* is either the index of the data object in the All Data list or the label of the data object.
>
> *Model\_index* is the index of a model in the data object.
>
> *Limit* is a double value corresponding to the miter limit.
>
> ##### Return value
>
> The return value is 0 if successful or -1 if a problem was encountered.

### setType

Sets the type option for the stroke of a plot model line.

> ##### Usage
>
> *integer* DelaModelLineStroke.setType(*Plot\_index, Data\_index\_or\_data\_label, Model\_index, Option*)
>
> ##### Arguments
>
> *Plot\_index* is an integer value corresponding to the index of a plot in the document contents.
>
> *Data\_index\_or\_data\_label* is either the index of the data object in the All Data list or the label of the data object.
>
> *Model\_index* is the index of a model in the data object.
>
> *Option* is an integer value corresponding to the type option. Recognized values are 0 (none), 1 (continuous), and 2 (dash).
>
> ##### Return value
>
> The return value is 0 if successful or -1 if a problem was encountered.

### setWidth

Sets the width for the stroke of a plot model line.

> ##### Usage
>
> *integer* DelaModelLineStroke.setWidth(*Plot\_index, Data\_index\_or\_data\_label, Model\_index, Width*)
>
> ##### Arguments
>
> *Plot\_index* is an integer value corresponding to the index of a plot in the document contents.
>
> *Data\_index\_or\_data\_label* is either the index of the data object in the All Data list or the label of the data object.
>
> *Model\_index* is the index of a model in the data object.
>
> *Width* is a double value corresponding to the width.
>
> ##### Return value
>
> The return value is 0 if successful or -1 if a problem was encountered.
