# Supplementary material for: Structural Organization and Dynamics of Homodimeric Cytohesin Family Arf GTPase Exchange Factors in Solution and on Membranes
Source: Structure. 2019 Dec 3;27(12):1782–1797.e7. doi: 10.1016/j.str.2019.09.007 (PMC6948192; doi:10.1016/j.str.2019.09.007)
Supplement: Data S1. Software, Related to the STAR Methods [file mmc3.zip › Software/Dela App Scripts/Dela.app/Contents/Resources/Dela.help/Contents/Resources/English.lproj/pgs/python_module_DelaPlot.html]

xml version="1.0" encoding="utf-8"?


Python Module DelaPlot


Home

Top
Contents


# DelaPlot python module

## Overview

The DelaPlot module provides methods related to plots.

### Getting counts for plot objects

dataCount

labelCount

### Getting and setting unicode text for plot objects

title

attributedDescription

xAxisLabel

yAxisLabel

rAxisLabel

label

setTitle

setAttributedDescription

setXAxisLabel

setYAxisLabel

setRAxisLabel

setLabel

### Getting indices for plot objects

dataIndices

selectedDataIndices

displayedDataIndices

### Getting and setting display options

displayModel

displayResiduals

setDisplayModel

setDisplayResiduals

### Getting and setting alternate axes options

alternateAxes

alternateAxesData

setAlternateAxes

setAlternateAxesData

### Getting and setting axis limits

xAxisMinimum

xAxisMaximum

yAxisMinimum

yAxisMaximum

rAxisMinimum

rAxisMaximum

setXAxisMinimum

setXAxisMaximum

setYAxisMinimum

setYAxisMaximum

setRAxisMinimum

setRAxisMaximum

setXAxisLimits

setYAxisLimits

setRAxisLimits

### Getting and setting selection rectangle limits

selectionRectangleXMin

selectionRectangleXMax

selectionRectangleYMin

selectionRectangleYMax

setSelectionRectangleXMin

setSelectionRectangleXMax

setSelectionRectangleYMin

setSelectionRectangleYMax

### Getting and setting colors

labelColor

dataColor

dataSymbolStrokeColor

dataSymbolFillColor

dataLineStrokeColor

setLabelColor

setDataColor

setDataSymbolStrokeColor

setDataSymbolFillColor

setDataLineStrokeColor

### Creating a new plot

newPlot

### Creating a new label

newLabel

## Methods

### alternateAxes

Returns the alternate axes option for a plot.

> ##### Usage
>
> *integer* DelaPlot.alternateAxes(*Plot\_index*)
>
> ##### Argument
>
> *Plot\_index* is an integer value corresponding to the index of a plot in the document contents.
>
> ##### Return value
>
> An integer value corresponding to the alternate axes option if successful or -1 if a problem was encountered. Recognized values are 0 (no alternates axes), 1 (alternate X axis), 2 (alternate Y axis), or 3 (alternate X and Y axes).

### alternateAxesData

Returns the option to include a data object in the alternate axes list  in a plot.

> ##### Usage
>
> *integer* DelaPlot.alternateAxesData(*Plot\_index, Data\_index*)
>
> ##### Argument
>
> *Plot\_index* is an integer value corresponding to the index of a plot in the document contents.
>
> *Data\_index* is an integer value corresponding to the All Data index of a data object in a plot.
>
> ##### Return value
>
> An integer value corresponding to the alternate axes data option if successful or -1 if a problem was encountered. Recognized values are 0 (not included) or 1 (included).

### attributedDescription

Returns the attributed description of a plot.

> ##### Usage
>
> *unicode* DelaPlot.attributedDescription(*Plot\_index*)
>
> ##### Argument
>
> *Plot\_index* is an integer value corresponding to the index of a plot in the document contents.
>
> ##### Return value
>
> A *unicode* string corresponding to the text content of the attributed description if successful or an empty NULL-terminated unicode string if a problem was encountered.

### dataColor

Returns the RGBA color elements of the color of a data object in a plot.

> ##### Usage
>
> *list* DelaPlot.dataColor(*Plot\_index, Data\_index*)
>
> ##### Arguments
>
> *Plot\_index* is an integer value corresponding to the index of a plot in the document contents.
>
> *Data\_index* is an integer value corresponding to the All Data index of a data object in a plot.
>
> ##### Return value
>
> A list of double values corresponding to the RGBA color elements or -1.0 if a problem was encountered. The color element list contains four double values: [red, green, blue, alpha]. The values range from 0.0-1.0.
>
> ##### Discussion
>
> The returned color corresponds to the symbol stroke if displayed, or (if not) the symbol fill if displayed, or (if not) the line stroke if display, or (if not) the default data symbol stroke.

### dataCount

Returns the number of data objects in a plot.

> ##### Usage
>
> *linteger* DelaPlot.dataCount(*Plot\_index*)
>
> ##### Argument
>
> *Plot\_index* is an integer value corresponding to the index of a plot in the document contents.
>
> ##### Return value
>
> An integer value corresponding to the number of data object if successful or -1 if a problem was encountered.

### dataIndices

Returns the All Data indices of the data objects in a plot.

> ##### Usage
>
> *list* DelaPlot.dataIndices(*Plot\_index*)
>
> ##### Argument
>
> *Plot\_index* is an integer value corresponding to the index of a plot in the document contents.
>
> ##### Return value
>
> A list of integer values corresponding to the All Data indicies of the data objects if successful or -1 if a problem was encountered.

### dataLineStrokeColor

Returns the RGBA color elements of the line stroke of a data object in a plot.

> ##### Usage
>
> *list* DelaPlot.dataLineStrokeColor(*Plot\_index, Data\_index*)
>
> ##### Arguments
>
> *Plot\_index* is an integer value corresponding to the index of a plot in the document contents.
>
> *Data\_index* is an integer value corresponding to the All Data index of a data object in a plot.
>
> ##### Return value
>
> A list of double values corresponding to the RGBA color elements or -1.0 if a problem was encountered. The color element list contains four double values: [red, green, blue, alpha]. The values range from 0.0-1.0.

### dataSymbolFillColor

Returns the RGBA color elements of the symbol fill of a data object in a plot.

> ##### Usage
>
> *list* DelaPlotdataSymbolFillColor(*Plot\_index, Data\_index*)
>
> ##### Arguments
>
> *Plot\_index* is an integer value corresponding to the index of a plot in the document contents.
>
> *Data\_index* is an integer value corresponding to the All Data index of a data object in a plot.
>
> ##### Return value
>
> A list of double values corresponding to the RGBA color elements or -1.0 if a problem was encountered. The color element list contains four double values: [red, green, blue, alpha]. The values range from 0.0-1.0.

### dataSymbolStrokeColor

Returns the RGBA color elements of the symbol stroke of a data object in a plot.

> ##### Usage
>
> *list* DelaPlot.dataSymbolStrokeColor(*Plot\_index, Data\_index*)
>
> ##### Arguments
>
> *Plot\_index* is an integer value corresponding to the index of a plot in the document contents.
>
> *Data\_index* is an integer value corresponding to the All Data index of a data object in a plot.
>
> ##### Return value
>
> A list of double values corresponding to the RGBA color elements or -1.0 if a problem was encountered. The color element list contains four double values: [red, green, blue, alpha]. The values range from 0.0-1.0.

### displayedDataIndices

Returns the All Data indices of the displayed data objects in a plot.

> ##### Usage
>
> *list* DelaPlot.displayedDataIndices(*Plot\_index*)
>
> ##### Argument
>
> *Plot\_index* is an integer value corresponding to the index of a plot in the document contents.
>
> ##### Return value
>
> A list of integer values corresponding to the All Data indicies of the data objects if successful or -1 if a problem was encountered.

### displayModel

Returns the display model setting for a plot.

> ##### Usage
>
> *integer* DelaPlot.displayModel(*Plot\_index*)
>
> ##### Argument
>
> *Plot\_index* is an integer value corresponding to the index of a plot in the document contents.
>
> ##### Return value
>
> An integer value corresponding to the display model setting if successful or -1 if a problem was encountered.

### displayResiduals

Returns the display residuals setting for a plot.

##### Usage

*integer* DelaPlot.displayResiduals(*Plot\_index*)

> ##### Argument
>
> *Plot\_index* is an integer value corresponding to the index of a plot in the document contents.
>
> ##### Return value
>
> An integer value corresponding to the display residuals setting if successful or -1 if a problem was encountered.

### label

Returns a label in a plot.

> ##### Usage
>
> *unicode* DelaPlot.label(*Plot\_index, Label\_index*)
>
> ##### Arguments
>
> *Plot\_index* is an integer value corresponding to the index of a plot in the document contents.
>
> *Label\_index* is an integer value corresponding to the index of a label in the plot.
>
> ##### Return value
>
> A unicode string corresponding to the label if successful or an empty NULL-terminated unicode string if a problem was encountered.

### labelColor

Returns the RGBA color elements of a label in a plot.

> ##### Usage
>
> *list* DelaPlot.labelColor(*Plot\_index, Label\_index*)
>
> ##### Arguments
>
> *Plot\_index* is an integer value corresponding to the index of a plot in the document contents.
>
> *Label\_index* is an integer value corresponding to the index of a label in a plot.
>
> ##### Return value
>
> A list of double values corresponding to the RGBA color elements or -1.0 if a problem was encountered. The color element list contains four double values: [red, green, blue, alpha]. The values range from 0.0-1.0.

### labelCount

Returns the number of labels in a plot.

> ##### Usage
>
> *integer* DelaPlot.labelCount(*Plot\_index*)
>
> ##### Argument
>
> *Plot\_index* is an integer value corresponding to the index of a plot in the document contents.
>
> ##### Return value
>
> An integer value corresponding to the number of labels if successful or -1 if a problem was encountered.

### newLabel

Creates a new label for a plot.

> ##### Usage
>
> *integer* DelaPlot.newLabel(*Plot\_index, Label*)
>
> ##### Arguments
>
> *Plot\_index* is an integer value corresponding to the index of a plot in the document contents.
>
> *Label* is a unicode or C-style (ASCII) string corresponding to the new label.
>
> ##### Return value
>
> The return value is 0  if successful or -1 if a problem was encountered.

### newPlot

Creates a new plot.

##### Usage

*integer* DelaPlot.newPlot(*Data\_objects*)

> ##### Arguments
>
> *Data\_objects* is an optional list of integer values corresponding to the indices of data objects in the All Data list.
>
> ##### Return value
>
> The return value is 0 if successful or -1 if a problem was encountered.
>
> ##### Discussion
>
> The plot is append to the plots in the document contents; however, it will not be visible until the document views are updated.

### rAxisLabel

Returns the residuals axis label of a plot.

> ##### Usage
>
> *unicode* DelaPlot.rAxisLabel(*Plot\_index*)
>
> ##### Argument
>
> *Plot\_index* is an integer value corresponding to the index of a plot in the document contents.
>
> ##### Return value
>
> A unicode string corresponding to the title if successful or an empty NULL-terminated unicode string if a problem was encountered.

### rAxisMaximum

Returns the residuals axis maximum of a plot.

> ##### Usage
>
> *double* DelaPlot.rAxisMaximum(*Plot\_index*)
>
> ##### Argument
>
> *Plot\_index* is an integer value corresponding to the index of a plot in the document contents.
>
> ##### Return value
>
> A double value corresponding to the residuals axis maximum if successful or -1 if a problem was encountered.

### rAxisMinimum

Returns the residuals axis minimum of a plot.

> ##### Usage
>
> *double* DelaPlot.rAxisMinimum(*Plot\_index*)
>
> ##### Argument
>
> *Plot\_index* is an integer value corresponding to the index of a plot in the document contents.
>
> ##### Return value
>
> A double value corresponding to the residuals axis minimum if successful or -1 if a problem was encountered.

### selectedDataIndices

Returns the All Data indices of the selected data objects in a plot.

> ##### Usage
>
> *list* DelaPlot.selectedDataIndices(*Plot\_index*)
>
> ##### Argument
>
> *Plot\_index* is an integer value corresponding to the index of a plot in the document contents.
>
> ##### Return value
>
> A list of integer values corresponding to the All Data indices of the data objects if successful or -1 if a problem was encountered.

### selectionRectangleXMax

Returns the selection rectangle x maximum of a plot.

> ##### Usage
>
> *double* DelaPlot.selectionRectangleXMax(*Plot\_index*)
>
> ##### Argument
>
> *Plot\_index* is an integer value corresponding to the index of a plot in the document contents.
>
> ##### Return value
>
> A double value corresponding to the selection rectangle x maximum if successful or -1 if a problem was encountered.

### selectionRectangleXMin

Returns the selection rectangle x minimum of a plot.

> ##### Usage
>
> *double* DelaPlot.selectionRectangleXMin(*Plot\_index*)
>
> ##### Argument
>
> *Plot\_index* is an integer value corresponding to the index of a plot in the document contents.
>
> ##### Return value
>
> A double value corresponding to the selection rectangle x minimum if successful or -1 if a problem was encountered.

### selectionRectangleYMax

Returns the selection rectangle y maximum of a plot.

> ##### Usage
>
> *double* DelaPlot.selectionRectangleYMax(*Plot\_index*)
>
> ##### Argument
>
> *Plot\_index* is an integer value corresponding to the index of a plot in the document contents.
>
> ##### Return value
>
> A double value corresponding to the selection rectangle y maximum if successful or -1 if a problem was encountered.

### selectionRectangleYMin

Returns the selection rectangle y minimum of a plot.

> ##### Usage
>
> *double* DelaPlot.selectionRectangleYMin(*Plot\_index*)
>
> ##### Argument
>
> *Plot\_index* is an integer value corresponding to the index of a plot in the document contents.
>
> ##### Return value
>
> A double value corresponding to the selection rectangle y minimum if successful or -1 if a problem was encountered.

### setAlternateAxes

Sets alternate axes option in a plot.

> ##### Usage
>
> *integer* DelaPlot.setAlternateAxes(*Plot\_index, Value*)
>
> ##### Arguments
>
> *Plot\_index* is an integer value corresponding to the index of a plot in the document contents.
>
> *Value* is an integer value corresponding to 0 (no alternate axes), 1 (alternate X axis), 2 (alternate Y axis), or 3 (alternate X and Y axes).
>
> ##### Return value
>
> The return value is 0  if successful or -1 if a problem was encountered.

### setAlternateAxesData

Sets the option to include data in the alternate axes list in a plot.

> ##### Usage
>
> *integer* DelaPlot.setAlternateAxesData(*Plot\_index, Data\_index, Value*)
>
> ##### Arguments
>
> *Plot\_index* is an integer value corresponding to the index of a plot in the document contents.
>
> *Data\_index* is an integer value corresponding to the All Data index of a data object in a plot.
>
> *Value* is an integer value corresponding to 0 (the data object is not included) or 1 (the data object is included).
>
> ##### Return value
>
> The return value is 0  if successful or -1 if a problem was encountered.

### setAttributedDescription

Sets the text content (and attributes via attribute tags) of the attributed description of a plot.

> ##### Usage
>
> *integer* DelaData.setAttributedDescription(*Data\_index\_or\_data\_label*, *Description*)
>
> ##### Arguments
>
> *Plot\_index* is an integer value corresponding to the index of a plot in the document contents.
>
> *Description* is a unicode or C-style (ASCII) string corresponding to the new description. *Description* is pre-processed for attribute tags.
>
> ##### Return value
>
> The return value is 0 if successful or -1 if a problem was encountered.

### setDataColor

Sets the color of a data object in a plot.

> ##### Usage
>
> *integer* DelaPlot.setDataColor(*Plot\_index, Data\_index, RGBA\_components*)
>
> ##### Arguments
>
> *Plot\_index* is an integer value corresponding to the index of a plot in the document contents.
>
> *Data\_index* is an integer value corresponding to the All Data index of a data object in a plot.
>
> *RGBA\_components* is a list of double values corresponding to RGBA color elements. The color element list should contain four double values: [red, green, blue, alpha]. The values range from 0.0-1.0.
>
> ##### Return value
>
> The return value is 0  if successful or -1 if a problem was encountered.

### setDataLineStrokeColor

Sets the line stroke color of a data object in a plot.

> ##### Usage
>
> *integer* DelaPlot.setDataLineStrokeColor(*Plot\_index, Data\_index, RGBA\_components*)
>
> ##### Arguments
>
> *Plot\_index* is an integer value corresponding to the index of a plot in the document contents.
>
> *Data\_index* is an integer value corresponding to the All Data index of a data object in a plot.
>
> *RGBA\_components* is a list of double values corresponding to RGBA color elements. The color element list should contain four double values: [red, green, blue, alpha]. The values range from 0.0-1.0.
>
> ##### Return value
>
> The return value is 0  if successful or -1 if a problem was encountered.

### setDataSymbolFillColor

Sets the symbol fill color of a data object in a plot.

> ##### Usage
>
> *integer* DelaPlot.setDataSymbolFillColor(*Plot\_index, Data\_index, RGBA\_components*)
>
> ##### Arguments
>
> *Plot\_index* is an integer value corresponding to the index of a plot in the document contents.
>
> *Data\_index* is an integer value corresponding to the All Data index of a data object in a plot.
>
> *RGBA\_components* is a list of double values corresponding to RGBA color elements. The color element list should contain four double values: [red, green, blue, alpha]. The values range from 0.0-1.0.
>
> ##### Return value
>
> The return value is 0  if successful or -1 if a problem was encountered.

### setDataSymbolStrokeColor

Sets the symbol stroke color of a data object in a plot.

> ##### Usage
>
> *integer* DelaPlot.setDataSymbolStrokeColor(*Plot\_index, Data\_index, RGBA\_components*)
>
> ##### Arguments
>
> *Plot\_index* is an integer value corresponding to the index of a plot in the document contents.
>
> *Data\_index* is an integer value corresponding to the All Data index of a data object in a plot.
>
> *RGBA\_components* is a list of double values corresponding to RGBA color elements. The color element list should contain four double values: [red, green, blue, alpha]. The values range from 0.0-1.0.
>
> ##### Return value
>
> The return value is 0  if successful or -1 if a problem was encountered.

### setDisplayModel

Sets display model setting in a plot.

> ##### Usage
>
> *integer* DelaPlot.setDisplayModel(*Plot\_index, Value*)
>
> ##### Arguments
>
> *Plot\_index* is an integer value corresponding to the index of a plot in the document contents.
>
> *Value* is an integer value corresponding to new display model setting (0 to hide; 1 to display).
>
> ##### Return value
>
> The return value is 0  if successful or -1 if a problem was encountered.

### setDisplayResiduals

Sets display residuals setting in a plot.

> ##### Usage
>
> *integer* DelaPlot.setDisplayResiduals(*Plot\_index, integer\_value*)
>
> ##### Arguments
>
> *Plot\_index* is an integer value corresponding to the index of a plot in the document contents.
>
> *Value* is an integer value corresponding to new display residuals setting (0 to hide; 1 to display).
>
> ##### Return value
>
> The return value is 0  if successful or -1 if a problem was encountered.

### setLabel

Sets a label of in a plot.

> ##### Usage
>
> *integer* DelaPlot.setLabel(*Plot\_index, Label\_index, Label*)
>
> ##### Arguments
>
> *Plot\_index* is an integer value corresponding to the index of a plot in the document contents.
>
> *Label\_index* is an integer value corresponding to the index of a label in the plot.
>
> *Label* is a unicode or C-style (ASCII) string corresponding to the new label.
>
> ##### Return value
>
> The return value is 0  if successful or -1 if a problem was encountered.

### setLabelColor

Sets the color of the label in a plot.

> ##### Usage
>
> *integer* DelaPlot.setLabeColorl(*Plot\_index, Label\_index, RGBA\_components*)
>
> ##### Arguments
>
> *Plot\_index* is an integer value corresponding to the index of a plot in the document contents.
>
> *Label\_index* is an integer value corresponding to the index of a label in a plot.
>
> *RGBA\_components* is a list of double values corresponding to RGBA color elements. The color element list should contain four double values: [red, green, blue, alpha]. The values range from 0.0-1.0.
>
> ##### Return value
>
> The return value is 0  if successful or -1 if a problem was encountered.

### setRAxisLabel

Sets the residuals axis label of a plot.

> ##### Usage
>
> *integer* DelaPlot.setRAxisLabel(*Plot\_index, R\_axis\_label*)
>
> ##### Arguments
>
> *Plot\_index* is an integer value corresponding to the index of a plot in the document contents.
>
> *R\_axis\_label* is a unicode or C-style (ASCII) string corresponding to the new residuals axis label.
>
> ##### Return value
>
> The return value is 0  if successful or -1 if a problem was encountered.

### setRAxisLimits

Sets the residuals axis minimum and maximum of a plot.

> ##### Usage
>
> *integer* DelaPlot.setRAxisLimits(*Plot\_index, Min\_value, Max\_value*)
>
> ##### Arguments
>
> *Plot\_index* is an integer value corresponding to the index of a plot in the document contents.
>
> *Min\_value and Max\_value* are double values corresponding to the new minimum and maximum.
>
> ##### Return value
>
> The return value is 0  if successful or -1 if a problem was encountered.

### setRAxisMaximum

Sets the residuals axis maximum of a plot.

> ##### Usage
>
> *integer* DelaPlot.setRAxisMaximum(*Plot\_index, double\_value*)
>
> ##### Arguments
>
> *Plot\_index* is an integer value corresponding to the index of a plot in the document contents.
>
> *Value* is a double value corresponding to new residuals axis maximum.
>
> ##### Return value
>
> The return value is 0  if successful or -1 if a problem was encountered.

### setRAxisMinimum

Sets the residuals axis minimum of a plot.

> ##### Usage
>
> *integer* DelaPlot.setRAxisMinimum(*Plot\_index, double\_value*)
>
> ##### Arguments
>
> *Plot\_index* is an integer value corresponding to the index of a plot in the document contents.
>
> *Value* is a double value corresponding to new residuals axis minimum.
>
> ##### Return value
>
> The return value is 0  if successful or -1 if a problem was encountered.

### setSelectionRectangleXMax

Sets the selection rectangle x maximum of a plot.

> ##### Usage
>
> *integer* DelaPlot.setSelectionRectangleXMax(*Plot\_index, Value*)
>
> ##### Arguments
>
> *Plot\_index* is an integer value corresponding to the index of a plot in the document contents.
>
> *Value* is a double value corresponding to new selection rectangle x maximum.
>
> ##### Return value
>
> The return value is 0  if successful or -1 if a problem was encountered.

### setSelectionRectangleXMin

Sets the selection rectangle x minimum of a plot.

> ##### Usage
>
> *integer* DelaPlot.setSelectionRectangleXMin(*Plot\_index, Value*)
>
> ##### Arguments
>
> *Plot\_index* is an integer value corresponding to the index of a plot in the document contents.
>
> *Value* is a double value corresponding to new selection rectangle x minimum.
>
> ##### Return value
>
> The return value is 0  if successful or -1 if a problem was encountered.

### setSelectionRectangleYMax

Sets the selection rectangle y maximum of a plot.

> ##### Usage
>
> *integer* DelaPlot.setSelectionRectangleYMax(*Plot\_index, Value*)
>
> ##### Arguments
>
> *Plot\_index* is an integer value corresponding to the index of a plot in the document contents.
>
> *Value* is a double value corresponding to new selection rectangle y maximum.
>
> ##### Return value
>
> The return value is 0  if successful or -1 if a problem was encountered.

### setSelectionRectangleYMin

Sets the selection rectangle y minimum of a plot.

> ##### Usage
>
> *integer* DelaPlot.setSelectionRectangleYMin(*Plot\_index, Value*)
>
> ##### Arguments
>
> *Plot\_index* is an integer value corresponding to the index of a plot in the document contents.
>
> *Value* is a double value corresponding to new selection rectangle y minimum.
>
> ##### Return value
>
> The return value is 0  if successful or -1 if a problem was encountered.

### setTitle

Sets the title of a plot.

> ##### Usage
>
> *integer* DelaPlot.setTitle(*Plot\_index, Title*)
>
> ##### Arguments
>
> *Plot\_index* is an integer value corresponding to the index of a plot in the document contents.
>
> *Title* is a unicode or C-style (ASCII) string corresponding to the new title.
>
> ##### Return value
>
> The return value is 0  if successful or -1 if a problem was encountered.

### setXAxisLabel

Sets the x axis label of a plot.

> ##### Usage
>
> *integer* DelaPlot.setXAxisLabel(*Plot\_index, X\_axis\_label*)
>
> ##### Arguments
>
> *Plot\_index* is an integer value corresponding to the index of a plot in the document contents.
>
> *X\_axis\_label* is a unicode or C-style (ASCII) string corresponding to the new x axis label.
>
> ##### Return value
>
> The return value is 0  if successful or -1 if a problem was encountered.

### setXAxisLimits

Sets the x axis minimum and maximum of a plot.

> ##### Usage
>
> *integer* DelaPlot.setXAxisLimits(*Plot\_index, Min\_Max*)
>
> ##### Arguments
>
> *Plot\_index* is an integer value corresponding to the index of a plot in the document contents.
>
> *Min\_value and Max\_value* are double values corresponding to the new minimum and maximum.
>
> ##### Return value
>
> The return value is 0  if successful or -1 if a problem was encountered.

### setXAxisMaximum

Sets the x axis maximum of a plot.

> ##### Usage
>
> *integer* DelaPlot.setXAxisMaximum(*Plot\_index, double\_value*)
>
> ##### Arguments
>
> *Plot\_index* is an integer value corresponding to the index of a plot in the document contents.
>
> *Value* is a double value corresponding to new x axis maximum.
>
> ##### Return value
>
> The return value is 0  if successful or -1 if a problem was encountered.

### setXAxisMinimum

Sets the x axis minimum of a plot.

> ##### Usage
>
> *integer* DelaPlot.setXAxisMinimum(*Plot\_index, Value*)
>
> ##### Arguments
>
> *Plot\_index* is an integer value corresponding to the index of a plot in the document contents.
>
> *Value* is a double value corresponding to new x axis minimum.
>
> ##### Return value
>
> The return value is 0  if successful or -1 if a problem was encountered.

### setYAxisLabel

Sets the y axis label of a plot.

> ##### Usage
>
> *integer* DelaPlot.setYAxisLabel(*Plot\_index, Y\_axis\_label*)
>
> ##### Arguments
>
> *Plot\_index* is an integer value corresponding to the index of a plot in the document contents.
>
> *Y\_axis\_label* is a unicode or C-style (ASCII) string corresponding to the new y axis label.
>
> ##### Return value
>
> The return value is 0  if successful or -1 if a problem was encountered.

### setYAxisLimits

Sets the y axis minimum and maximum of a plot.

> ##### Usage
>
> *integer* DelaPlot.setYAxisLimits(*Plot\_index, Min\_Max*)
>
> ##### Arguments
>
> *Plot\_index* is an integer value corresponding to the index of a plot in the document contents.
>
> *Min\_value and Max\_value* are double values corresponding to the new minimum and maximum.
>
> ##### Return value
>
> The return value is 0  if successful or -1 if a problem was encountered.

### setYAxisMaximum

Sets the y axis maximum of a plot.

> ##### Usage
>
> *integer* DelaPlot.setYAxisMaximum(*Plot\_index, double\_value*)
>
> ##### Arguments
>
> *Plot\_index* is an integer value corresponding to the index of a plot in the document contents.
>
> *Value* is a double value corresponding to new y axis maximum.
>
> ##### Return value
>
> The return value is 0  if successful or -1 if a problem was encountered.

### setYAxisMinimum

Sets the y axis minimum of a plot.

> ##### Usage
>
> *integer* DelaPlot.setYAxisMinimum(*Plot\_index, double\_value*)
>
> ##### Arguments
>
> *Plot\_index* is an integer value corresponding to the index of a plot in the document contents.
>
> *Value* is a double value corresponding to new y axis minimum.
>
> ##### Return value
>
> The return value is 0  if successful or -1 if a problem was encountered.

### title

Returns the title of the plot.

> ##### Usage
>
> *unicode* DelaPlot.title(*Plot\_index*)
>
> ##### Argument
>
> *Plot\_index* is an integer value corresponding to the index of a plot in the document contents.
>
> ##### Return value
>
> A unicode string corresponding to the title if successful or an empty NULL-terminated unicode string if a problem was encountered.

### xAxisLabel

Returns the x axis label of a plot.

> ##### Usage
>
> *unicode* DelaPlot.xAxisLabel(*Plot\_index*)
>
> ##### Argument
>
> *Plot\_index* is an integer value corresponding to the index of a plot in the document contents.
>
> ##### Return value
>
> A unicode string corresponding to the title if successful or an empty NULL-terminated unicode string if a problem was encountered.

### xAxisMaximum

Returns the x axis maximum of a plot.

> ##### Usage
>
> *double* DelaPlot.xAxisMaximum(*Plot\_index*)
>
> ##### Argument
>
> *Plot\_index* is an integer value corresponding to the index of a plot in the document contents.
>
> ##### Return value
>
> A double value corresponding to the x axis maximum if successful or -1 if a problem was encountered.

### xAxisMinimum

Returns the x axis minimum of a plot.

> ##### Usage
>
> *double* DelaPlot.xAxisMinimum(*Plot\_index*)
>
> ##### Argument
>
> *Plot\_index* is an integer value corresponding to the index of a plot in the document contents.
>
> ##### Return value
>
> A double value corresponding to the x axis minimum if successful or -1 if a problem was encountered.

### yAxisLabel

Returns the y axis label of a plot.

> ##### Usage
>
> *unicode* DelaPlot.yAxisLabel(*Plot\_index*)
>
> ##### Argument
>
> *Plot\_index* is an integer value corresponding to the index of a plot in the document contents.
>
> ##### Return value
>
> A unicode string corresponding to the title if successful or an empty NULL-terminated unicode string if a problem was encountered.

### yAxisMaximum

Returns the y axis maximum of a plot.

> ##### Usage
>
> *double* DelaPlot.yAxisMaximum(*Plot\_index*)
>
> ##### Argument
>
> *Plot\_index* is an integer value corresponding to the index of a plot in the document contents.
>
> ##### Return value
>
> A double value corresponding to the y axis maximum if successful or -1 if a problem was encountered.

### yAxisMinimum

Returns the y axis minimum of a plot.

> ##### Usage
>
> *double* DelaPlot.yAxisMinimum(*Plot\_index*)
>
> ##### Argument
>
> *Plot\_index* is an integer value corresponding to the index of a plot in the document contents.
>
> ##### Return value
>
> A double value corresponding to the y axis minimum if successful or -1 if a problem was encountered.
