# Supplementary material for: Structural Organization and Dynamics of Homodimeric Cytohesin Family Arf GTPase Exchange Factors in Solution and on Membranes
Source: Structure. 2019 Dec 3;27(12):1782–1797.e7. doi: 10.1016/j.str.2019.09.007 (PMC6948192; doi:10.1016/j.str.2019.09.007)
Supplement: Data S1. Software, Related to the STAR Methods [file mmc3.zip › Software/Dela App Scripts/Dela.app/Contents/Resources/Dela.help/Contents/Resources/English.lproj/pgs/python_module_DelaPlotMetrics.html]

xml version="1.0" encoding="utf-8"?


Python Module DelaPlotMetrics


Home

Top
Contents


# DelaPlotMetrics python module

## Overview

The DelaPlotMetrics module provides methods related to plot metrics.

### Getting and setting axis lengths

xAxisLength

yAxisLength

setXAxisLength

setYAxisLength

### Getting and setting the residual axis fractions

residualAxisFraction

setResidualAxisFraction

## Methods

### xAxisLength

Returns the x axis length of a plot.

> ##### Usage
>
> *double* DelaPlotMetrics.xAxisLength(*Plot\_index, Units*)
>
> ##### Arguments
>
> *Plot\_index* is an integer value corresponding to the index of a plot in the document contents.
>
> *Units* is a unicode or C-style (ASCII) string corresponding to the desired units. Recognized units are "cm" or u"cm" (centimeters), "in" or u"in" (inches), and "px" or u"px" (pixels). Inches are assumed if *Units* is omitted.
>
> ##### Return value
>
> A double value corresponding to the x axis length in the specified units if successful or -1 if a problem was encountered.

### yAxisLength

Returns the y axis length of a plot.

> ##### Usage
>
> *double* DelaPlotMetrics.yAxisLength(*Plot\_index*)
>
> ##### Arguments
>
> *Plot\_index* is an integer value corresponding to the index of a plot in the document contents.
>
> *Units* is a unicode or C-style (ASCII) string corresponding to the desired units. Recognized units are "cm" or u"cm" (centimeters), "in" or u"in" (inches), and "px" or u"px" (pixels). Inches are assumed if *Units* is omitted.
>
> ##### Return value
>
> A double value corresponding to the y axis length in the specified units if successful or -1 if a problem was encountered.

### residualAxisFraction

Returns the residual axis fraction of a plot.

> ##### Usage
>
> *double* DelaPlotMetrics.residualAxisFraction(*Plot\_index*)
>
> ##### Argument
>
> *Plot\_index* is an integer value corresponding to the index of a plot in the document contents.
>
> ##### Return value
>
> A double value corresponding to the residual axis fraction if successful or -1 if a problem was encountered.

### setXAxisLength

Sets the x axis length of a plot.

> ##### Usage
>
> *integer* DelaPlotMetrics.setXAxisLength(*Plot\_index, Units, Value*)
>
> ##### Arguments
>
> *Plot\_index* is an integer value corresponding to the index of a plot in the document contents.
>
> *Units* is a unicode or C-style (ASCII) string corresponding to the desired units. Recognized units are "cm" or u"cm" (centimeters), "in" or u"in" (inches), and "px" or u"px" (pixels). Inches are assumed if *Units* is omitted.
>
> *Value* is a double value corresponding to x axis length in the specified units.
>
> ##### Return value
>
> The return value is 0  if successful or -1 if a problem was encountered.

### setYAxisLength

Sets the y axis length of a plot.

> ##### Usage
>
> *integer* DelaPlotMetrics.setYAxisLength(*Plot\_index, Units, Value*)
>
> ##### Arguments
>
> *Plot\_index* is an integer value corresponding to the index of a plot in the document contents.
>
> *Units* is a unicode or C-style (ASCII) string corresponding to the desired units. Recognized units are "cm" or u"cm" (centimeters), "in" or u"in" (inches), and "px" or u"px" (pixels). Inches are assumed if *Units* is omitted.
>
> *Value* is a double value corresponding to y axis length in the specified units.
>
> ##### Return value
>
> The return value is 0  if successful or -1 if a problem was encountered.

### setResidualAxisFraction

Sets the residual axis fraction of a plot.

> ##### Usage
>
> *integer* DelaPlotMetrics.setResidualAxisFraction(*Plot\_index, Value*)
>
> ##### Arguments
>
> *Plot\_index* is an integer value corresponding to the index of a plot in the document contents.
>
> *Value* is a double value corresponding to the residual axis fraction.
>
> ##### Return value
>
> The return value is 0  if successful or -1 if a problem was encountered.
