# Supplementary material for: Structural Organization and Dynamics of Homodimeric Cytohesin Family Arf GTPase Exchange Factors in Solution and on Membranes
Source: Structure. 2019 Dec 3;27(12):1782–1797.e7. doi: 10.1016/j.str.2019.09.007 (PMC6948192; doi:10.1016/j.str.2019.09.007)
Supplement: Data S1. Software, Related to the STAR Methods [file mmc3.zip › Software/Dela App Scripts/Dela.app/Contents/Resources/Dela.help/Contents/Resources/English.lproj/pgs/python_module_DelaSheet.html]

xml version="1.0" encoding="utf-8"?


Python Module DelaSheet


Home

Top
Contents


# DelaSheet python module

## Overview

The DelaSheet module provides methods related to sheets.

### Getting the number of data objects in a sheet

dataCount

### Getting unicode text descriptions for a sheet

description

attributedDescription

### Getting indices for data objects in a sheet

dataIndices

highlightedDataIndices

checkedDataIndices

### Setting unicode text descriptions for a sheet

setDescription

setAttributedDescription

### Creating a new sheet

newSheet

## Methods

### attributedDescription

Returns the attributed description of a sheet.

> ##### Usage
>
> *unicode* DelaSheet.attributedDescription(*Sheet\_index*)
>
> ##### Argument
>
> *Sheet\_index* is an integer value corresponding to the index of a sheet in the document contents.
>
> ##### Return value
>
> A *unicode* string corresponding to the text content of the attributed description if successful or an empty NULL-terminated unicode string if a problem was encountered.

### checkedDataIndices

Returns a list of the indices of the checked data objects in a sheet.

> ##### Usage
>
> *list* DelaSheet.checkedDataIndices(*Sheet\_index*)
>
> ##### Argument
>
> *Sheet\_index* is an integer value corresponding to the index of a sheet in the document contents.
>
> ##### Return value
>
> A list of integer values corresponding to the indices or [-1] if a problem was encountered.

### dataCount

Returns the total number of data objects a sheet.

> ##### Usage
>
> *integer* DelaSheet.dataCount(*Sheet\_index*)
>
> ##### Argument
>
> *Sheet\_index* is an integer value corresponding to the index of a sheet in the document contents.
>
> ##### Return value
>
> An integer value corresponding to the number of data objects if successful or -1 if a problem was encountered.

### dataIndices

Returns a list of the indices of the data objects in a sheet.

> ##### Usage
>
> *list* DelaSheet.dataIndices(*Sheet\_index*)
>
> ##### Argument
>
> *Sheet\_index* is an integer value corresponding to the index of a sheet in the document contents.
>
> ##### Return value
>
> A list of integer values corresponding to the indices or [-1] if a problem was encountered.

### description

Returns the description of a sheet.

> ##### Usage
>
> *unicode* DelaSheet.description(*sheet\_index*)
>
> ##### Argument
>
> *Sheet\_index* is an integer value corresponding to the index of a sheet in the document contents.
>
> ##### Return value
>
> A *unicode* string containing the description or an empty NULL-terminated *unicode* string if a problem was encountered.

### highlightedDataIndices

Returns a list of the indices of the highlighted data objects in a sheet.

> ##### Usage
>
> *list* DelaSheet.highlightedDataIndices(*Sheet\_index*)
>
> ##### Argument
>
> *Sheet\_index* is an integer value corresponding to the index of a sheet in the document contents.
>
> ##### Return value
>
> A list of integer values corresponding to the indices or [-1] if a problem was encountered.

### newSheet

Creates a new sheet.

> ##### Usage
>
> *integer*  DelaSheet.newSheet(*Data\_objects, Description*)
>
> ##### Arguments
>
> *Data\_objects* is an optional list of integer values corresponding to the indices of data objects in the All Data list.
>
> *Description* is an optional unicode or C-style (ASCII) string corresponding to the description for the new sheet. *Description* is pre-processed for attribute tags.
>
> ##### Return value
>
> The return value is 0 if successful or -1 if a problem was encountered.

### setAttributedDescription

Sets the text content (and attributes via attribute tags) of the attributed description of a sheet.

> ##### Usage
>
> *integer* DelaSheet.setAttributedDescription(*Sheet\_index*, *Description*)
>
> ##### Arguments
>
> *Sheet\_index* is an integer value corresponding to the index of a sheet in the document contents.
>
> *Description* is a unicode or C-style (ASCII) string corresponding to the new description. *Description* is pre-processed for attribute tags.
>
> ##### Return value
>
> The return value is 0 if successful or -1 if a problem was encountered.

### setDescription

Sets the description of a sheet.

> ##### Usage
>
> *integer* DelaSheet.setDescription(*Sheet\_index, Description*)
>
> ##### Arguments
>
> *Sheet\_index* is an integer value corresponding to the index of a sheet in the document contents.
>
> *Description* is a unicode or C-style (ASCII) string corresponding to the new description. *Description* is pre-processed for attribute tags.
>
> ##### Return value
>
> The return value is 0 if successful or -1 if a problem was encountered.
