# Supplementary material for: Structural Organization and Dynamics of Homodimeric Cytohesin Family Arf GTPase Exchange Factors in Solution and on Membranes
Source: Structure. 2019 Dec 3;27(12):1782–1797.e7. doi: 10.1016/j.str.2019.09.007 (PMC6948192; doi:10.1016/j.str.2019.09.007)
Supplement: Data S1. Software, Related to the STAR Methods [file mmc3.zip › Software/Dela App Scripts/Dela.app/Contents/Resources/Dela.help/Contents/Resources/English.lproj/pgs/python_module_DelaStatistics.html]

xml version="1.0" encoding="utf-8"?


Python Module DelaStatistics


Home

Top
Contents


# DelaStatistics python module

## Overview

The DelaStatistics module provide methods related to statistical analyses.

### Getting the number of elements

size

### Getting the bounds

minimum

maximum

extent

### Getting the sum, mean, and median

sum

mean

median

### Getting the variance, standard deviation, and standard error

variance

standardDeviation

standardError

### Getting the skewness and kurtosis

skewness

kurtosis

### Getting the moments and cumulants

momemt

cumulant

### Getting Gaussian noise

gaussianNoise

## Methods

### cumulant

Returns the Nth cumulant of an array.

> ##### Usage
>
> *integer* DelaStatistics.cumulant(*Data\_array, Mask\_array, N*)
>
> ##### Arguments
>
> *Data\_array* is a list of double values for the analysis.
>
> *Mask\_array* is an optional list of integer values corresponding to a binary mask for the values in *Data\_array*.
>
> *N* is an integer value corresponding to the order (e.g. first = 1, second = 2, third = 3, fourth = 4).
>
> ##### Return value
>
> A double value containing the Nth cumulant if successful or -1 if a problem was encountered.

### extent

Returns the extent (maximum - minimum) of an array.

> ##### Usage
>
> *integer* DelaStatistics.extent(*Data\_array, Mask\_array*)
>
> ##### Arguments
>
> *Data\_array* is a list of double values for the analysis.
>
> *Mask\_array* is an optional list of integer values corresponding to a binary mask for the values in *Data\_array*.
>
> ##### Return value
>
> A double value containing the extent if successful or -1 if a problem was encountered.

### gaussianNoise

Returns the next value in a sequence of random Gaussian noise.

> ##### Usage
>
> *integer* DelaStatistics.gaussianNoise(*Seed*)
>
> ##### Arguments
>
> *Seed* is an optional integer that determines the particular random sequence of Gaussian noise values. If not supplied, *Seed* defaults to 34527968. If *Seed* is negative, the random Gaussion noise generator will be reinitialized
>
> ##### Return value
>
> A double value containing the next value in the Gaussian noise series if successful or 0.0 if a problem was encountered.

### kurtosis

Returns the kurtosis of an array.

> ##### Usage
>
> *integer* DelaStatistics.kurtosis(*Data\_array, Mask\_array*)
>
> ##### Arguments
>
> *Data\_array* is a list of double values for the analysis.
>
> *Mask\_array* is an optional list of integer values corresponding to a binary mask for the values in *Data\_array*.
>
> ##### Return value
>
> A double value containing the kurtosis if successful or -1 if a problem was encountered.

### maximum

Returns the maximum of an array.

> ##### Usage
>
> *integer* DelaStatistics.maximum(*Data\_array, Mask\_array*)
>
> ##### Arguments
>
> *Data\_array* is a list of double values for the analysis.
>
> *Mask\_array* is an optional list of integer values corresponding to a binary mask for the values in *Data\_array*.
>
> ##### Return value
>
> A double value containing the maximum if successful or -1 if a problem was encountered.

### mean

Returns the mean of an array.

> ##### Usage
>
> *integer* DelaStatistics.mean(*Data\_array, Mask\_array*)
>
> ##### Arguments
>
> *Data\_array* is a list of double values for the analysis.
>
> *Mask\_array* is an optional list of integer values corresponding to a binary mask for the values in *Data\_array*.
>
> ##### Return value
>
> A double value containing the mean if successful or -1 if a problem was encountered.

### median

Returns the mean of an array.

> ##### Usage
>
> *integer* DelaStatistics.median(*Data\_array, Mask\_array*)
>
> ##### Arguments
>
> *Data\_array* is a list of double values for the analysis.
>
> *Mask\_array* is an optional list of integer values corresponding to a binary mask for the values in *Data\_array*.
>
> ##### Return value
>
> A double value containing the median if successful or -1 if a problem was encountered.

### minimum

Returns the minimum of an array.

> ##### Usage
>
> *integer* DelaStatistics.minimum(*Data\_array, Mask\_array*)
>
> ##### Arguments
>
> *Data\_array* is a list of double values for the analysis.
>
> *Mask\_array* is an optional list of integer values corresponding to a binary mask for the values in *Data\_array*.
>
> ##### Return value
>
> A double value containing the minimum if successful or -1 if a problem was encountered.

### momemt

Returns the Nth moment of an array.

> ##### Usage
>
> *integer* DelaStatistics.momemt(*Data\_array, Mask\_array, N*)
>
> ##### Arguments
>
> *Data\_array* is a list of double values for the analysis.
>
> *Mask\_array* is an optional list of integer values corresponding to a binary mask for the values in *Data\_array*.
>
> *N* is an integer value corresponding to the order (e.g. first = 1, second = 2, third = 3, fourth = 4).
>
> ##### Return value
>
> A double value containing the Nth moment if successful or -1 if a problem was encountered.

### size

Returns the size of an array.

> ##### Usage
>
> *integer* DelaStatistics.size(*Data\_array, Mask\_array*)
>
> ##### Arguments
>
> *Data\_array* is a list of double values for the analysis.
>
> *Mask\_array* is an optional list of integer values corresponding to a binary mask for the values in *Data\_array*.
>
> ##### Return value
>
> An integer value containing the size if successful or -1 if a problem was encountered.

### skewness

Returns the skewness of an array.

> ##### Usage
>
> *integer* DelaStatistics.skewness(*Data\_array, Mask\_array*)
>
> ##### Arguments
>
> *Data\_array* is a list of double values for the analysis.
>
> *Mask\_array* is an optional list of integer values corresponding to a binary mask for the values in *Data\_array*.
>
> ##### Return value
>
> A double value containing the skewness if successful or -1 if a problem was encountered.

### standardDeviation

Returns the standard deviation of an array.

> ##### Usage
>
> *integer* DelaStatistics.standardDeviation(*Data\_array, Mask\_array*)
>
> ##### Arguments
>
> *Data\_array* is a list of double values for the analysis.
>
> *Mask\_array* is an optional list of integer values corresponding to a binary mask for the values in *Data\_array*.
>
> ##### Return value
>
> A double value containing the standard deviation if successful or -1 if a problem was encountered.

### standardError

Returns the standard error of an array.

> ##### Usage
>
> *integer* DelaStatistics.standardError(*Data\_array, Mask\_array*)
>
> ##### Arguments
>
> *Data\_array* is a list of double values for the analysis.
>
> *Mask\_array* is an optional list of integer values corresponding to a binary mask for the values in *Data\_array*.
>
> ##### Return value
>
> A double value containing the standard error if successful or -1 if a problem was encountered.

### sum

Returns the sum of an array.

> ##### Usage
>
> *integer* DelaStatistics.sum(*Data\_array, Mask\_array*)
>
> ##### Arguments
>
> *Data\_array* is a list of double values for the analysis.
>
> *Mask\_array* is an optional list of integer values corresponding to a binary mask for the values in *Data\_array*.
>
> ##### Return value
>
> A double value containing the sum if successful or -1 if a problem was encountered.

### variance

Returns the variance of an array.

> ##### Usage
>
> *integer* DelaStatistics.variance(*Data\_array, Mask\_array*)
>
> ##### Arguments
>
> *Data\_array* is a list of double values for the analysis.
>
> *Mask\_array* is an optional list of integer values corresponding to a binary mask for the values in *Data\_array*.
>
> ##### Return value
>
> A double value containing the variance if successful or -1 if a problem was encountered.
