# Supplementary material for: Structural Organization and Dynamics of Homodimeric Cytohesin Family Arf GTPase Exchange Factors in Solution and on Membranes
Source: Structure. 2019 Dec 3;27(12):1782–1797.e7. doi: 10.1016/j.str.2019.09.007 (PMC6948192; doi:10.1016/j.str.2019.09.007)
Supplement: Data S1. Software, Related to the STAR Methods [file mmc3.zip › Software/Dela App Scripts/Dela.app/Contents/Resources/Dela.help/Contents/Resources/English.lproj/pgs/python_module_DelaUI.html]

xml version="1.0" encoding="utf-8"?


Python Module DelaUI


Home

Top
Contents


# DelaUI python module

## Overview

The DelaUI module provides methods related to interaction with the user interface in the Interpret script panel.

### Displaying mesages to the user interface

displayString

### Getting input from the user interface

getString

getInteger

getDouble

### Getting files paths

getOpenFilePath

getSaveFilePath

scriptPath

### Creating and displaying sheets

beginSheet

displaySheet

### Appending controls to a sheet

appendCheckButton

appendHeader

appendPopUp

appendTextField

### Appending and selecting menu items

appendPopUpItem

selectPopUpItem

### Getting values from controls in a sheet

checkButtonValue

popUpSelectedItem

textFieldDouble

textFieldInteger

textFieldString

### Setting tool tips for controls in a sheet

setTitleToolTip

setToolTip

## Methods

### appendCheckButton

Appends a check button to a sheet previously created with beginSheet.

> ##### Usage
>
> *integer* DelaUI.appendCheckButton(*prompt\_string*, *state*)
>
> ##### Arguments
>
> *prompt\_string* is a unicode or C-style (ASCII) string.
>
> *state* is an integer value corresponding to the state of the check button, which will be unchecked if state is 0 and checked if state is 1.
>
> ##### Return value
>
> An integer value representing an identifier for the control if successful or -1.0 if a problem was encountered.

### appendHeader

Appends a header field to a sheet previously created with beginSheet.

> ##### Usage
>
> *integer* DelaUI.appendHeader(*header\_string*)
>
> ##### Arguments
>
> *header\_string* is a unicode or C-style (ASCII) string.
>
> ##### Return value
>
> An integer value representing an identifier for the control if successful or -1.0 if a problem was encountered.

### appendPopUp

Appends a pop up button to a sheet previously created with beginSheet.

> ##### Usage
>
> *integer* DelaUI.appendPopUp(*prompt\_string*)
>
> ##### Arguments
>
> *prompt\_string* is a unicode or C-style (ASCII) string.
>
> ##### Return value
>
> An integer value representing an identifier for the control if successful or -1.0 if a problem was encountered.
>
> ##### Discussion
>
> The pop up button is created with an empty menu. Menu items can be added with appendPopUpItem.

### appendPopUpItem

Appends an item to the menu of a pop up button previously created with appendPopUp.

> ##### Usage
>
> *integer* DelaUI.appendPopUpItem(*identifier*, *prompt\_string, item\_string*)
>
> ##### Arguments
>
> *identifier* is the unique integer identifier returned by appendPopUp.
>
> *prompt\_string* is a unicode or C-style (ASCII) string.
>
> *item\_string* is a unicode or C-style (ASCII) string for the menu item.
>
> ##### Return value
>
> The return value is 0 if successful or -1 if a problem was encountered.

### appendTextField

Appends a text field to a sheet previously created with beginSheet.

> ##### Usage
>
> *integer* DelaUI.appendTextField(*prompt\_string, text\_field\_value*)
>
> ##### Arguments
>
> *prompt\_string* is a unicode or C-style (ASCII) string.
>
> *text\_field\_value* is an integer, double, unicode string, or C-style (ASCII) string value.
>
> ##### Return value
>
> An integer value representing an identifier for the control if successful or -1.0 if a problem was encountered.

### beginSheet

Creates a new sheet that can subsequently be displayed to the user interface with *displaySheet*.

> ##### Usage
>
> *integer* DelaUI.beginSheet()
>
> ##### Return value
>
> The return value is 0 if successful or -1 if a problem was encountered.
>
> ##### Discussion
>
> The newly created sheet replaces any sheet previously created with beginSheet. The sheet existis for the duration of script execution unless replaced by a subsequent call to beginSheet.

### checkButtonValue

Returns the state value of a check button previously created with appendCheckButton.

> ##### Usage
>
> *integer* DelaUI.checkButtonValue(*identifier*)
>
> ##### Arguments
>
> *identifier* is the unique integer identifier returned by appendCheckButton.
>
> ##### Return value
>
> An integer value representing the state of the check button, which will 0 if unchecked, 1 if checked, or -1 if a problem was encountered.

### displaySheet

Displays a sheet previously created with beginSheet.

> ##### Usage
>
> *integer* DelaUI.displaySheet(*title\_string*)
>
> ##### Argument
>
> *title\_string* is a unicode or C-style (ASCII) string that will be displayed as a title for the sheet.
>
> ##### Return value
>
> The return value is 0 if successful or -1 if a problem was encountered.
>
> **Discussion**
>
> Script execution is paused until the user responds.

### displayString

Displays a string to the user interface.

> ##### Usage
>
> *integer* DelaUI.displayString(*String*)
>
> ##### Argument
>
> *String* is a unicode or C-style (ASCII) string.
>
> ##### Return value
>
> The return value is 0 if successful or -1 if a problem was encountered.
>
> **Discussion**
>
> Script execution is paused until the user responds.

### getDouble

Gets a double value from the user interface.

> ##### Usage
>
> *double* DelaUI.getDouble(*querry\_string, default\_double\_value*)
>
> ##### Arguments
>
> Arguments are a *querry\_string* and a *default\_double\_value*.
>
> ##### Return value
>
> A double value if successful or -1.0 if a problem was encountered.
>
> **Discussion**
>
> Script execution is paused until the user responds.

### getInteger

Gets an integer value from the user interface.

> ##### Usage
>
> *integer* DelaUI.getInteger(*querry\_string, default\_integer\_value*)
>
> ##### Arguments
>
> Arguments are a unicode or C-style (ASCII) *querry\_string* and a *default\_integer\_value*.
>
> ##### Return value
>
> An integer value if successful or -1 if a problem was encountered.
>
> **Discussion**
>
> Script execution is paused until the user responds.

### getString

Gets a *unicode* string from the user interface.

> ##### Usage
>
> *unicode* DelaUI.getString(*prompt\_string, *default\_string**)
>
> ##### Arguments
>
> *prompt\_string* and *default\_string* are unicode or C-style (ASCII) strings.
>
> ##### Return value
>
> A unicode string if successful or an empty NULL-terminated unicode string if a problem was encountered.

### getOpenFilePath

Gets an open file path from the user interface.

> ##### Usage
>
> *unicode* DelaUI.getOpenFilePath(*prompt\_string, default\_file\_path*)
>
> ##### Arguments
>
> *prompt\_string* and *default\_file\_path* are unicode or C-style (ASCII) strings.
>
> ##### Return value
>
> A unicode string containing the file path if successful or an empty NULL-terminated unicode string if a problem was encountered.
>
> **Discussion**
>
> Initiates an open file navigation dialog displaying the *prompt\_string*. Script execution is paused until the user responds.

### getSaveFilePath

Gets a save file path from the user interface.

> ##### Usage
>
> *unicode* DelaUI.getSaveFilePath(*prompt\_string, default\_file\_path*)
>
> ##### Arguments
>
> *prompt\_string* and *default\_file\_path* are unicode or C-style (ASCII) strings.
>
> ##### Return value
>
> A unicode string containing the file path if successful or an empty NULL-terminated unicode string if a problem was encountered.
>
> **Discussion**
>
> Initiates a save file navigation dialog displaying the *prompt\_string*. Script execution is paused until the user responds.

### popUpSelectedItem

Returns the selected item in the menu of a pop up button previously created with appendPopUp.

> ##### Usage
>
> *integer* DelaUI.popUpSelectedItem(*identifier*)
>
> ##### Arguments
>
> *identifier* is the unique integer identifier returned by appendPopUp.
>
> ##### Return value
>
> An integer value representing the index of the selected menu item or -1 if a problem was encountered. The index of the first menu item is 0.

### scriptPath

Returns the script path from the user interface.

> ##### Usage
>
> *unicode* DelaUI.scriptPath()
>
> ##### Return value
>
> A unicode string containing the script path if successful or an empty NULL-terminated unicode string if a problem was encountered.

### selectPopUpItem

Selects and displays an item in the menu of a pop up button previously created with appendPopUp.

> ##### Usage
>
> *integer* DelaUI.selectPopUpItem(*identifier*, *item\_index*)
>
> ##### Arguments
>
> *identifier* is the unique integer identifier returned by appendPopUp.
>
> *item\_index* is an integer value corresponding to the index of the item in the menu. The index of the first item is 0.
>
> ##### Return value
>
> The return value is 0 if successful or -1 if a problem was encountered.

### setTitleToolTip

Sets the tool tip string for the title of a sheet.

> ##### Usage
>
> *integer* DelaUI.setTitleToolTip(*tool\_tip\_string*)
>
> ##### Argument
>
> *tool\_tip\_string* is a unicode or C-style (ASCII) string.
>
> ##### Return value
>
> The return value is 0 if successful or -1 if a problem was encountered.

### setToolTip

Sets the tool tip string for a control in a sheet.

> ##### Usage
>
> *integer* DelaUI.setToolTip(*identifier*, *tool\_tip\_string*)
>
> ##### Arguments
>
> *identifier* is the unique integer identifier returned by the append method for the control.
>
> *tool\_tip\_string* is a unicode or C-style (ASCII) string.
>
> ##### Return value
>
> The return value is 0 if successful or -1 if a problem was encountered.

### textFieldDouble

Returns a double value after conversion of the contents of a text field previously created with appendTextField.

> ##### Usage
>
> *double* DelaUI.textFieldDouble(*identifier*)
>
> ##### Arguments
>
> *identifier* is the unique integer identifier returned by appendTextField.
>
> ##### Return value
>
> A double value resulting from conversion of the text field contents, or -1.0 if a problem was encountered.

### textFieldInteger

Returns an integer value after conversion of the contents of a text field previously created with appendTextField.

> ##### Usage
>
> *integer* DelaUI.textFieldInteger(*identifier*)
>
> ##### Arguments
>
> *identifier* is the unique integer identifier returned by appendTextField.
>
> ##### Return value
>
> An integer value resulting from conversion of the text field contents, or -1.0 if a problem was encountered.

### textFieldString

Returns a unicode string corresponding to the contents of a text field previously created with appendTextField.

> ##### Usage
>
> *unicode* DelaUI.textFieldString(*identifier*)
>
> ##### Arguments
>
> *identifier* is the unique integer identifier returned by appendTextField.
>
> ##### Return value
>
> A unicode string value corresponding to the text field contents, or an empy NULL-terminated unicode string if a problem was encountered.
