# Supplementary material for: Structural Organization and Dynamics of Homodimeric Cytohesin Family Arf GTPase Exchange Factors in Solution and on Membranes
Source: Structure. 2019 Dec 3;27(12):1782–1797.e7. doi: 10.1016/j.str.2019.09.007 (PMC6948192; doi:10.1016/j.str.2019.09.007)
Supplement: Data S1. Software, Related to the STAR Methods [file mmc3.zip › Software/Dela App Scripts/Dela.app/Contents/Resources/Dela.help/Contents/Resources/English.lproj/pgs/python_saxs_scripts.html]

xml version="1.0" encoding="utf-8"?


Python SAXS scripts


Home

Top
Contents


# Python scripts for SAXS data processing and analysis

## Overview

The scripts described below utilize python modules and methods provided by the python interpreter embedded in DELA.  One or more plots, sheets, or data objects may need to be selected in the document contents prior to script execution.  Some scripts require that a particular data object (e.g. buffer scattering) be identified by its data label (e.g. Data123).

## Available scripts

### Plot I vs. q

SAXS\_IvsQ\_plot.py - *Plot I(q) vs. q*

### Scattering profiles (e.g. SAXS chromatograms)

SAXS\_Mean\_Scattering\_Profile.py - *Plot mean scattering vs. data index*   
SAXS\_Total\_Scattering\_Profile.py - *Plot total scattering vs. data index*

### Beam intensity normalization

SAXS\_Normalization.py - *Normalize scattering data*   
SAXS\_Normalization\_exploration.py - *Explore normalization options*   
SAXS\_Normalization\_exploration\_weights.py - *Explore normalization options*   
SAXS\_Undo\_Normalization.py - *Undo normalization of scattering data*

### Plot data with indices bounded by selection rectangle

SAXS\_Plot\_Data\_With\_Indices\_In\_Selection\_Regions.py- *Plot data with indices in selection regions*

### Buffer subtraction

SAXS\_Buffer\_Subtraction.py - *Buffer subtraction with optional buffer scaling*   
SAXS\_Buffer\_Subtraction\_Guinier\_Analysis.py - *Guinier optimized buffer subtraction*

### Plot data with optimal signal to noise

SAXS\_Plot\_Data\_With\_Optimal\_StoN.py - *Plot data with optimal S/N*   
SAXS\_Plot\_Data\_With\_SR\_Indices\_Optimal\_StoN\_SR.py - *Plot data with optimal S/N in SR region*

### Reconstruction of macromolecular scattering

SAXS\_Linear\_Combination\_Guinier\_Analysis.py - *Guinier analysis of coefficients for linear combination*

### Transform plots

SAXS\_Guinier\_Plot.py - *Guinier transform and plot*  
SAXS\_Kratky\_Plot.py - *Kratky transform and plot*  
SAXS\_Log\_Log\_Plot.py - *Log y vs. log x plot*  
SAXS\_Porod\_Plot.py - *Porod transform and plot*

### Guinier plots, fits and profiles

SAXS\_Guinier\_Fit.py - *Guinier fit with options*  
SAXS\_Guinier\_Fit\_with\_defaults.py - *Guinier fit with default options*  
SAXS\_Guinier\_Plot\_Fit.py - *Guinier transform, plot and fit*  
SAXS\_Guinier\_Profile.py - *Profile of Rg vs. data index*

### Simulation

SAXS\_SEC-SAXS\_simulation.py - *Simulation of SEC-SAXS* data sets.

## Descriptions

### SAXS\_Buffer\_Subtraction.py

Buffer subtraction with optional buffer scaling.

> ##### Input
>
> selected plot(s), sheet(s), or data object(s) with sample scattering data
>
> data label for buffer scattering
>
> ##### Output
>
> plot with new buffer subtracted data
>
> sheet with new buffer subtracted data

### SAXS\_Buffer\_Subtraction\_Guinier\_Analysis.py

Guinier optimized buffer subtraction with buffer scaling determined by linearity of the Guinier region.

> ##### Input
>
> selected plot(s), sheet(s), or data object(s) with sample scattering data
>
> data label for the buffer (defaults to last data object)
>
> ##### Output
>
> plot of R-squared vs. scaling constant for buffer
>
> plot with new buffer subtracted data
>
> sheet with new buffer subtracted data

### SAXS\_Guinier\_Fit.py

Guinier fit with options.

> ##### Input
>
> selected plot with Guinier transformed data
>
> ##### Output
>
> fitted linear least squares model(s)

### SAXS\_Guinier\_Fit\_with\_defaults.py

Guinier fit with default options. Same as SAXS\_Guinier\_Fit.py script except that the default options are used without presenting a sheet to the user interface.

> ##### Input
>
> selected plot with Guinier transformed data
>
> ##### Output
>
> fitted linear least squares model(s)

### SAXS\_Guinier\_Plot.py

Guinier transform and plot.

> ##### Input
>
> selected plot(s), sheet(s), or data object(s) with buffer subtracted scattering data
>
> ##### Output
>
> Guinier plot(s)
>
> sheet with Guinier transformed data

### SAXS\_Guinier\_Plot\_Fit.py

Guinier transform, plot and fit.

> ##### Input
>
> selected plot(s), sheet(s), or data object(s) with buffer subtracted scattering data
>
> ##### Output
>
> Guinier plot(s) and fitted linear least squares model
>
> sheet with Guinier transformed data object(s)

### SAXS\_Guinier\_Profile.py

Profile of Rg vs. data index.

> ##### Input
>
> selected plot(s), sheet(s), or data object(s) with buffer subtracted scattering data
>
> ##### Output
>
> sheet with Guinier profile with Rg, I0, q x Rg, and R2
>
> ##### Discussion
>
> It may be helpful to plot the Rg profile with the mean or total scattering profile using alternate axes

### SAXS\_IvsQ\_Plot.py

Plot of I(q) as a function of q.

> ##### Input
>
> selected plot(s), sheet(s), or data object(s) with scattering data
>
> ##### Output
>
> I vs. q plot(s)

### SAXS\_Kratky\_Plot.py

Kratky transform and plot.

> ##### Input
>
> selected plot(s), sheet(s), or data object(s) with buffer subtracted scattering data
>
> ##### Output
>
> Kratky plot(s)
>
> sheet with Kratky transformed data

### SAXS\_Linear\_Combination\_Guinier\_Analysis.py

Reconstruct macromolecular scattering by Guinier analysis of coefficients for linear combination of the U0 and U1 basis components from SVD of normalized scattering data.

> ##### Input
>
> selected data objects representing U0 and U1 from SVD of normalized scattering data
>
> ##### Output
>
> plot of R-squared vs. linear coefficient for U0
>
> plot with linear combination of U0 and U1 using optimal coefficient for U0
>
> sheet with linear combination of U0 and U1 using optimal coefficient for U0

### SAXS\_Log\_Log\_Plot.py

Plot of log y as a function of log x.

> ##### Input
>
> selected plot(s), sheet(s), or data object(s) with buffer subtracted scattering data
>
> ##### Output
>
> log(I) vs. log(q) plot(s)
>
> sheet with log(I) and log(q) transformed data

### **SAXS\_Mean\_Scattering\_Profile.py**

Plot mean scattering vs. data index.

> ##### Input
>
> selected plot(s), sheet(s), or data object(s) with scattering data
>
> ##### Output
>
> mean scattering vs. data index plot(s)

### SAXS\_Normalization.py

Normalize scattering data.

> ##### Input
>
> selected scattering data
>
> data label for incident beam intensity (I0); defaults to last data with description matching "I0"
>
> data label transmitted beam intensity (I1); defaults to last data with description matching "I1"
>
> ##### Output
>
> normalized scattering data; original data are replaced
>
> optional plot of total scattering intensity for each normalized data object

### SAXS\_Normalization\_exploration.py

Explore normalization options.

> ##### Input
>
> selected plot with total or mean scattering of buffer subtracted data
>
> data label for incident beam intensity (I0); defaults to last data with description matching "I0"
>
> data label transmitted beam intensity (I1); defaults to last data with description matching "I1"
>
> ##### Output
>
> plot(s) with normalized total scattering vs. data index
>
> sheet(s) with with normalized total scattering vs. data index

### SAXS\_Normalization\_exploration\_weights.py

Explore of normalization options with I0 and I1 weights incremented before constant weights. Otherwise, identical to the SAXS\_Normalization\_exploration script.

> ##### Input
>
> selected plot with total or mean scattering of buffer subtracted data
>
> data label for incident beam intensity (I0); defaults to last data with description matching "I0"
>
> data label transmitted beam intensity (I1); defaults to last data with description matching "I1"
>
> ##### Output
>
> plots with normalized total scattering vs. data index
>
> sheets with with normalized total scattering vs. data index

### SAXS\_Plot\_Data\_With\_Indices\_In\_Selection\_Regions.py

Plot of data with indices in one or more selection regions.

> ##### Input
>
> plot with total or mean scattering profile, where the x values are indices of corresponding to scattering profiles; the bounds for the region(s) of interest should be set before running the script.
>
> ##### Output
>
> plot(s) of scattering data with indices defined by selection rectangle

### SAXS\_Plot\_Data\_With\_Optimal\_StoN.py

Plot data with optimal S/N.

> ##### Input
>
> plot with buffer subtracted scattering data
>
> ##### Output
>
> plot(s) of buffer subtracted scattering data with optimal signal-to-noise when averaged

### SAXS\_Plot\_Data\_With\_SR\_Indices\_Optimal\_StoN\_SR.py

Plot data with optimal S/N in SR region.

> ##### Input
>
> plot with total or mean scattering of buffer subtracted data; region of interest should be bound by the selection rectangle
>
> ##### Output
>
> plot(s) of buffer subtracted scattering data with optimal signal-to-noise when averaged

### SAXS\_Porod\_Plot.py

Porod transform and plot.

> ##### Input
>
> selected plot(s), sheet(s), or data object(s) with buffer subtracted scattering data
>
> ##### Output
>
> Porod plot(s)
>
> sheet with Porod transformed data

### SAXS\_SEC-SAXS\_simulation.py

Simulation of SEC-SAXS from sample/buffer scattering components and concentration profiles

> ##### Input
>
> Will need to provide indices for sheets with: 1) one or more macromolecular scattering profiles; 2) the corresponding concentration profiles in the same order as the macromolecular scattering profiles; and 3) scattering profiles for the buffer and optional cell in that order.
>
> ##### Output
>
> Sheet and plot with simulated SEC-SAXS data sets.

### SAXS\_Total\_Scattering\_Profile.py

Plot total scattering vs. data index.

> ##### Input
>
> selected plot(s), sheet(s), or data object(s) with scattering data
>
> ##### Output
>
> mean scattering vs. data index plot(s)

### SAXS\_Undo\_Normalization.py

Undo normalization of scattering data previously normalized by I0 or I1.

> ##### Input
>
> selected scattering data normalized by either I0 or I1
>
> data label for incident beam intensity (I0); defaults to last data with description matching "I0"
>
> data label transmitted beam intensity (I1); defaults to last data with description matching "I1"
>
> ##### Output
>
> un-normalized scattering data; original data are replaced
>
> optional plot of total scattering intensity for each un-normalized data object

## Importing, plotting and normalizing scattering data

### Importing SAXS data

(modify as required by data format)

> 1. Drag selected data files or click on import in the file menu
>
> 2. Select X-ray Scattering in the Data Type menu
>
> 3. Click import

### Importing beam intensities

(modify as required by data format)

> 1. Drag selected data files or click on import in the file menu
>
> 2. Generic Sheet in the Data Type menu
>
> 3. Select has column labels under header
>
> 4. Select has row labels and Ignore under header
>
> 5. Select Y... under columns
>
> 6. Change default descriptions as desired
>
> 7. Click import

### Plotting the scattering data

(modify as required by data format)

> Select the sheet with the scattering data and run the SAXS\_IvsQ\_Plot.py script.

### Undoing normalization

> If the data were previously normalized by dividing by either I0 or I1, undo the normalization using the SAXS\_Undo\_Normalization.py script. By default, this script will generate a total scattering profile (e.g. SAXS chromatogram) using the un-normalized scattering data.

### Exploring normalization options

> 1. Since the relevant scripts typically generate multiple sheets and plots with different combinations of I0 and I1 weights and a constant to be subtracted, it is recommended that normalization exploration be done using a copy of the original DELA document file.
>
> 2. Select the plot of the un-normalized total or mean scattering profile.
>
> 3. Run the SAXS\_Normalization\_exploration.py (or SAXS\_Normalization\_exploration\_weights.py) script with different options to determine what works best. Typically, dividing by the product of I0 and I1 is better than dividing by either alone. In some cases, even better results can be obtained using a weighted average of I0 and I1, but this typically requires evaluating different combinations of the weights and constant.

### Normalizing the scattering data

> Select the plot with the un-normalized scattering data in the original document and run the SAXS\_Normalization.py script using the best option, weights, and/or constant determined in the exploration step. By default, the script generates a total scattering profile for the normalized data.

## Buffer subtraction and Rg profile

### Selecting, plotting, and averaging of peak and buffer regions

> 1. Select the plot of the total scattering profile for the normalized data.
>
> 2. Using the selection rectangle, encompass the point(s) representing the maximum of the peak of interest and run the SAXS\_Plot\_Data\_With\_Indices\_In\_Selection\_Regions.py script.
>
> 3. Select the new plot with the scattering data representing the peak of interest and choose Average from the Transform menu.
>
> 4. Re-select the plot of the total scattering profile for the normalized data.
>
> 5. Using the selection rectangle with the SAXS\_Plot\_Data\_With\_Indices\_In\_Selection\_Regions.py script, encompass one or more regions representing buffer.
>
> 6. Select the new plot with the scattering data representing the buffer and choose Average from the Transform menu.

### Subtracting the buffer scattering

> Select the sheet (or plot) with the averaged peak data and run the SAXS\_Buffer\_Subtraction.py or SAXS\_Buffer\_Subtraction\_Guinier\_Analysis.py script. The former script is useful for direct buffer subtraction without optimization of the buffer scaling constant (although a scaling constant can be supplied or calculated). The latter script is useful when automated determination of an optimal buffer scaling constant based on linearity of the Guinier region is desired.

### Generating an RG profile

> 1. Select the plot with all of the normalized scattering data and run the SAXS\_Buffer\_Subtraction.py script. If necessary, undo the script and re-run with a different scaling constant.
>
> 2. Select the plot with the buffer subtracted scattering data and run the SAXS\_Guinier\_Profile.py script.
>
> 3. Plot the RG profile in the newly generated sheet with total or mean scattering profile for the normalized data using alternative Y axes.

## Singular value decomposition and linear combination

### Singular Value Decomposition

SVD can be done on any matrix of two or more data objects having an identical number of points. It can used as a purely diagnostic tool, as a noise filter, and/or to construct a set of orthonormal basis components for linear combination.

> 1. Select a plot or sheet containing normalized scattering data, which may correspond to all or part of the experiment (e.g. the peak of interest in a SAXS chromatogram), and choose Singular Value Decomposition from the Calculate menu. The output will be a sheet with: i) the N (= 10 by default) most significant columns of U; ii) the N most significant columns of V multiplied by the corresponding singular values; iii) the N most significant singular values; and iv) the auto-correlations of the N most significant columns of U and V.
>
> 2. Inspect the singular values and auto-correlations. For this purpose it is useful to plot the singular values on a log Y scale separately from the auto-correlations, which can be plotted together or separately on a linear scale. Noise components will typically have monotonically decreasing singular values and low auto-correlations (e.g. < 0.3). Signal components of interest will typically have singular values substantially larger than the noise components and high auto-correlations (e.g. > 0.7). For a single homogenous macromolecular species in a uniform buffer, two significant components are expected. The presence of additional significant components is indicative of additional macromolecular species, non-uniform buffer, or systematic errors with the experiment (e.g. radiation damage). It may be possible to reduce the number of significant components to two, or otherwise reduce the contribution of the additional significant components, by restricting the range of data using the SAXS\_Plot\_Data\_With\_Indices\_In\_Selection\_Regions.py script.

### Reconstruction of macromolecular scattering by linear combination

> 1. Identify a set of scattering data objects for which SVD results in only two significant components apart from noise (i.e. the basis components that span the space represented by a single macromolecular species in a uniform buffer). An RG profile as described above may be useful for selecting candidate regions using the SAXS\_Plot\_Data\_With\_Indices\_In\_Selection\_Regions.py script. In contrast to buffer subtraction, it is not necessary to identify or even include pure buffer scattering, as long as there sufficient variation in macromolecular vs. buffer scattering. In addition, inclusion of more scattering data will increase signal-to-noise and consequently the quality of the reconstruction, provided the requirement for only two significant components is satisfied.
>
> 2. Select the U0 and U1 data objects in the singular value decomposition of interest and run the SAXS\_Linear\_Combination\_Guinier\_Analysis.py script. The output includes: i) the reconstructed macromolecular scattering corresponding to the linear combination that maximizes the linearity of the Guinier region as judged by R2 merit statistic; and ii) a plot of R2 as a function of the linear coefficient for U0. Using a q × RG cutoff of < 1.3 is relatively robust; however, a more stringent cutoff (e.g. q × RG < 1.0) may improve accuracy. The R2 vs. U0 coefficient plot should be smoothly varying with a well defined maximum. If not, the reconstruction should be considered suspect.
