# Supplementary material for: Structural Organization and Dynamics of Homodimeric Cytohesin Family Arf GTPase Exchange Factors in Solution and on Membranes
Source: Structure. 2019 Dec 3;27(12):1782–1797.e7. doi: 10.1016/j.str.2019.09.007 (PMC6948192; doi:10.1016/j.str.2019.09.007)
Supplement: Data S1. Software, Related to the STAR Methods [file mmc3.zip › Software/Dela App Scripts/Dela.app/Contents/Resources/Dela.help/Contents/Resources/English.lproj/pgs/python_scripts.html]

xml version="1.0" encoding="utf-8"?


Python scripts


Home

Top
Contents


# Python scripts

## Python scripts

DELA provides an embedded interpreter for python scripts, which can be executed from the Interpret script panel.

## Python script files

The interpreter recognizes python script file names that end with the extension .py (case sensitive). Python script files can be executed or imported from any accessible path. Direct execution via a specified file path is convenient for testing, scripts that are rarely executed, or scripts requiring frequent modification. Imported scripts are copied to a location in the user's default directory path and made available to all documents via the Script menu in the Interpret script panel.

Several DELA Python scripts are provided with the application distribution, including scripts for processing and analysis of SAXS data.

## DELA python modules

Dela supports python scripting through the set of modules described below. These modules are defined when the embedded interpreter is launched and do not need to be imported within the script. Return type and required arguments are listed for each module method. For some methods, arguments are automatically recognized by type and/or how many are supplied.

### General form

All DELA python modules begin with 'Dela' (to distinguish Dela modules froms other modules that might otherwise have the same name) and have the follwing general form:

> *return\_value* **DelaModuleName.methodName**(*argument1*, *argument2*, ...)

where the number of argumements, their type, and the type of the return values depends on the particular method.

### Argument and return types

Unless otherwise noted, DELA module methods accept arguments and return values that have one of the following types:

> *an interger* number
>
> *a double* precission floating point number
>
> *a unicode* *string*
>
> a C-style (ASCII) *string*
>
> a list containg a sequence of values with one of the above types

Methods involving strings typically return unicode strings and accept either unicode or C-style (ASCII) strings as arguments. In addition, strings supplied as arguments to module methods are preprocessed for embedded tags specifying unicode characters, fonts, character attributes, or paragraph styles, whenever those string will be dsiplayed in a context (e.g. plots or the script log view) where string attribute and formating information is relevent.

## Available modules

### User interface and script log view

DelaUI

DelaLog

### Document

DelaDocument

### Data objects

DelaData

### Sheets

DelaSheet

### Plots

DelaPlot

DelaPlotMetrics

### Plot axes

DelaAxis

DelaMajorTics

DelaMinorTics

DelaAxisStroke

### Plot data symbols and line

DelaDataSymbol

DelaDataSymbolStroke

DelaDataSymbolFill

DelaDataLineStroke

### Plot model symbols and line

DelaModelSymbol

DelaModelSymbolStroke

DelaModelSymbolFill

DelaModelLineStroke

### Statistics and Maximum Likelihood

DelaStatistics

DelaML

### Errors

DelaError
