# Supplementary material for: Structural Organization and Dynamics of Homodimeric Cytohesin Family Arf GTPase Exchange Factors in Solution and on Membranes
Source: Structure. 2019 Dec 3;27(12):1782–1797.e7. doi: 10.1016/j.str.2019.09.007 (PMC6948192; doi:10.1016/j.str.2019.09.007)
Supplement: Data S1. Software, Related to the STAR Methods [file mmc3.zip › Software/Dela App Scripts/Dela.app/Contents/Resources/Dela.help/Contents/Resources/English.lproj/pgs/quadratic_binding.html]

xml version="1.0" encoding="utf-8"?


Quadratic Binding


Home

Contents


# Quadratic Binding

## Model function

Y (X; ***P***) = ***P0*** + ***P1***\*X + ***P2*** \* (b - sqrt(b^2 - 4 \* ac) / 2) [ + ... + ***Pn-2*** \* (bn - sqrt(bn^2 - 4 \* acn) / 2) ]

where

b = 1 + x / (***P4*** \* ***P2***) + ***P5*** / (***P4*** \* ***P2***)

ac = x / (***P4*** \* ***P2***)

bn = 1 + x / (***Pn-1*** \* ***P2***) + ***Pn*** / (***Pn-1*** \* ***P2***)

acn = x / (***Pn-1*** \* ***P2***)

## Parameters

### Non-term parameters

***P0*** - constant

***P1*** - slope

***P2*** - concentration of the fixed component

### Term parameters

***P3 ... Pn-2*** - amplitude

***P4 ... Pn-1*** - Stoichiometry (molecules variable component / molecules fixed component)

***P5 ... Pn*** - dissociation constant (Kd)

For more general information about models, see Model Functions.
