# Supplementary material for: Structural Organization and Dynamics of Homodimeric Cytohesin Family Arf GTPase Exchange Factors in Solution and on Membranes
Source: Structure. 2019 Dec 3;27(12):1782–1797.e7. doi: 10.1016/j.str.2019.09.007 (PMC6948192; doi:10.1016/j.str.2019.09.007)
Supplement: Data S1. Software, Related to the STAR Methods [file mmc3.zip › Software/Dela App Scripts/Dela.app/Contents/Resources/Dela.help/Contents/Resources/English.lproj/pgs/scripts.html]

xml version="1.0" encoding="utf-8"?


Interpreter


Home

Contents


# Scripts

## DELA scripts

DELA scripts are complete programs consisting of source code for the Interpreter, which can be executed from the Interpret script panel.

## Script files

The interpreter recognizes standard ASCII characters extended to 8-bits using UTF-8 encoding. For this reasons, script files should be saved as plain text with UTF-8 encoding. Script file names shoud end with the extension .dela (case sensitive).

Script files can be executed or imported from any accessible path. Direct execution via a specified file path is convenient for testing, scripts that are rarely executed, or scripts requiring frequent modification. Imported scripts are copied from the orignal script to a location in the user's default directory path and made available to all documents via the Script menu in the Interpret script panel.

## Anatomy of a script program

The following simple example illustrates the basic architecture of a Dela script, which shares syntatical similarities as well as differences with the C programing language.

### Hello world script

> /\*  
> ------------------------------------------------------------------  
> DELA script to write "Hello World !!!" to the log text view (and log file if specified)  
> ------------------------------------------------------------------  
> \*/
>
> int main ( )
>
> {
>
> > // print to the default output destination, typically the log text view in the Interpret script panel and to a log file if supplied by the user
> >
> > printf ("Hello world !!!\n");
>
> }

Strictly speaking, the only essential code elements are the int main ( ) statement and the braces { and }. The int main () statement tells the interpreter where the program actually begins. The braces enclose a block of code typically containing multipe lines. Execution begins with the with opening { and ends with the closing }.

The comment lines at the top enclosed by /\* and \*/ are ignored by the interpreter, as is the comment line following //. In general /\* and \*/ can be used for single or multiline comments whereas // can only be used for a single line comment. The end of a line is defined by the presence of a new line character (\n). Return characters (\r) are ignored.

The only actual line of code in the block is the printf("Hello world !!!\n"); instruction, which tells the interpreter to write Hello world !!! follwed by a new line (\n) to the default output destination. The printf ( ) function is one of many internal functions recognized by the Interpreter.

### Hello world log

The Hello world script produces the following log

> Interpret Script Log
>
> Script File
>
> /Users/User\_home\_directory/Desktop/test.dela
>
> Started August 31, 2013 6:19:13 PM EDT
>
> Hello world !!!
>
> Finished August 31, 2013 6:19:13 PM EDT

where the text between the Started and Finished time stamps is generated by the script.

## Functions for use with scripts

The functions described below extend the basic functions of the interpreter. For several scripts functions, the return type and some of the argument types depend on a key string. Different key strings and associated arguments are essentially requests for different information. The return type and required arguments are listed for each key.

### Input and output from the default stream

*char* **getc()**

Returns a character from the default input stream

**putc()**

Sends a character to the default output stream

**puts(*string*)**

Sends a ***string*** (after appending a newline character) to the default output stream using the C function puts

**print(*expr, expr2 ...*)**

Output to the default output stream; the arguments expr, expr2 ... can be any combination of strings, numerical constants, or variables seperated by commas. The number of arguments is returned. This is the simplest way to combine string and variable output when precise formatting is not required.

**printf("...", ...)**

Output to the default output stream where the arguments "...", ... have the form of a C-like printf function

### Requesting user input

*int* **GetInt(*querry, default*)**

Get an integer value from the default input stream; arguments are a ***querry*** string or character array and a ***default*** integer value; the entered integer value, or the default if no value was entered, is returned

*double* **GetDouble(*querry, default*)**

Get a double value from the default input stream; arguments are a ***querry*** string or character array and a ***default*** double value; the double value entered, or the default if no value was entered, is returned

*int* **GetString(*querry, char\_array*)**

Get a string from the default input stream; arguments are a ***querry*** string or character array and a ***char\_array*** character array; ***char\_array*** supplies the default string and contains the entered string, or the default string if no string was entered, on return; the number of characters in the string is returned

*char* **GetOpenFilePath(*prompt\_string\_or\_char\_array*)**

Initiates an open file navigation dialog with the prompt message specified by ***prompt\_string\_or\_char\_array*** and returns the selected file path.

*char* **GetSaveFilePath(*prompt\_string\_or\_char\_array*)**

Initiates a save file navigation dialog with the prompt message specified by ***prompt\_string\_or\_char\_array*** and returns the selected file path.

**DisplayMessage(*message\_string\_or\_char\_array*)**

Displays ***message\_string\_or\_char\_array***

### File input and output

*int* **ReadArray(*path, array, ...*)**

Read columns of numbers from a file specified by the POSIX style file ***path*** into one or more numerical arrays specified by the subsequent arguments. The order and type of the arrays supplied as arguements should correspond to the expected order and type of columns of numbers in the file. The array dimensions should be large enough to hold the anticipated number of elements. The number of lines read from the file is returned. ReadArray takes care of openning and closing the file.

*int* **WriteArray(*path, nlines, array, ...*)**

Write columns of numbers to a file specified by the POSIX style file ***path*** from one or more numerical arrays specified by the subsequent arguments. The number of lines written to be written to the the file is limited by the integer argument nlines, which is also is returned. WriteArray takes care of openning and closing the file.

*int* **OpenFile(*path, access*)**

Opens the file specified by the string or character array ***path***. The character constant ***access*** must be either "r" (read only) or "w" (write only). An integer value serving as a file pointer for future reference is returned

**CloseFile(*file\_pointer*)**

Closes the file specified by the integer value ***file\_pointer*** returned by OpenFile

*int* **ReadLine(file\_pointer, char\_array)**

Reads a line from the file specified by the integer value ***file\_pointer*** returned by OpenFile into the character array ***char\_array***. The number of characters read is returned

**WriteLine(*file\_pointer, char\_array*)**

Writes a character array ***char\_array*** to the file specified by the integer value ***file\_pointer*** returned by OpenFile

**fprint(*file\_pointer, expr, expr2 ...*)**

Output to the open file specified by ***file\_pointer***; the arguments expr, expr2 ... can be any combination of strings, numerical constants, or variables seperated by commas. The number of arguments is returned.

**fprintf(*file\_pointer*, "...", ...)**

Output to the open file specified by ***file\_pointer*** where the arguments "...", ... have the form of a C-like printf function

### String functions

*int* **StringLength(*string\_or\_char\_array*)**

Returns the length of a string contined within ***string\_or\_char\_array*** terminated by the null character '\0'. Depending on the location of the null character, the length strings stored in character arrays may be less than the array size.

**TerminateString(*char\_array, index*)**

Terminates a character array with '\0' at the element of ***char\_array*** specified by ***index***

**StringCopy(*char\_array, string\_or\_char\_array*)**

Copies the string or character array ***string\_or\_char\_array*** to the character array ***char\_array***

*int* **StringCompare(*string\_or\_char\_array\_1, string\_or\_char\_array\_2*)**

Returns -1 if ***string\_or\_char\_array\_1*** < ***string\_or\_char\_array\_2***, 0 if they are identical, and 1 if ***string\_or\_char\_array\_1*** > ***string\_or\_char\_array\_2***

*char* **StringConcatenate(*string\_or\_char\_array\_1, string\_or\_char\_array\_2*)**

Returns a string formed by concatenation of ***string\_or\_char\_array\_1*** and ***string\_or\_char\_array\_2***

*char* **SubString(*string\_or\_char\_array, index\_1, index\_2*)**

Returns a sub-string consisting of the range of characters in ***string\_or\_char\_array*** begining with ***index\_1*** and including subsequent characters with indices < ***index\_2***

**StringPrint(*char\_array*, "...", ...)**

Output to ***char\_array:*** the arguments expr, expr2 ... can be any combination of strings, numerical constants, or variables seperated by commas. The number of arguments is returned.

### Array functions

*type\_of\_first\_argument* **SubArray(*char\_int\_or\_double\_array, index\_1, index\_2*)**

Returns a sub-array consisting of the range of elements in ***char\_int\_or\_double\_array*** begining with ***index\_1*** and including subsequent elements with indices < ***index\_2***

### Conversion of numeric strings

*int* **atoi(*string\_or\_char\_array*)**

Returns an integer value after conversion of ***string\_or\_char\_array***

*double* **atof(*string\_or\_char\_array*)**

Returns a double value after conversion of ***string\_or\_char\_array***

### Document functions

*type\_depends\_on\_key* **GetDocumentArray(*key*, *data\_index\_or\_label, array\_key*)**

Returns a copy of a document array

> ***key***, one of the following strings: "data"
>
> ***data\_index\_or\_label***, either an integer with the index of a data object in the document data list or a string containing the label of a data object
>
> ***array\_key***, string specifying the desired array in the data object (i.e. arg must be "x", "y", "e", or "m", for the x, y , error, or mask arrays, respectively)

**SetDocumentArray(*key*, *data\_index\_or\_label, array\_key, new\_array\_values*)**

Sets the values in a document array to the values in a supplied array

> ******key******, one of the following strings: "data" (currently the only option)
>
> ***data\_index\_or\_label***, either an integer with the index of a data object in the document data list or a string containing the label of a data object
>
> ***array\_key***, string specifying the desired array in the data object (i.e. "x", "y", "e", or "m", for the x, y , error, or mask arrays, respectively)
>
> ***new\_array\_values***, array of type int (for a mask arrays) or double (for x, y, or error arrays)

**NewDocumentData(*data\_description, array\_size, sheet\_description, #\_data\_objects*)**

Creates one or more new data objects along with a new sheet

> ***data\_description***, string describing the data object
>
> ***array\_size***, integer specifying the size of the data arrays
>
> ***sheet\_description***, string describing the sheet containing the data
>
> ***#\_data\_objects***, the number of new data object to be created

**NewDocumentSheet(*data\_indicies\_or\_initial\_index [, final\_index]*)**

Creates a new plot with one or more data objects

> ***data\_indicies\_or\_initial\_index***, integer with either an array of indices or an initial index of data objects in the document data list
>
> ***final\_index***, integer specificy the final index of a data object in the document data list

**NewDocumentPlot(*data\_indicies\_or\_initial\_index [, final\_index]*)**

Creates a new plot with one or more data objects

> ***data\_indicies\_or\_initial\_index***, integer with either an array of indices or an initial index of data objects in the document data list
>
> ***final\_index***, integer specificy the final index of a data object in the document data list

*type\_depends\_on\_key* **GetDocumentProperty(*key*, *arguments*)**

Returns the value of the document property specified by one of the following ***key*** strings and key dependent ***arguments***:

> COUNT AND SIZE
>
> *int* **GetDoumentProperty(*"data count"*)**
>
> Returns the total number of data objects
>
> *int* **GetDoumentProperty(*"sheet count"*)**
>
> Returns the total number of sheets
>
> *int* **GetDoumentProperty(*"plot count"*)**
>
> Returns the total number of plots
>
> *int* **GetDoumentProperty(*"data count in sheet", sheet\_index*)**
>
> Returns the total number of data objects in the sheet specified by ***sheet\_index***
>
> *int* **GetDoumentProperty(*"data count in plot", plot\_index*)**
>
> Returns the total number of data objects in the plot specified by ***plot\_index***
>
> *int* **GetDoumentProperty(*"label count in plot", plot\_index*)**
>
> Returns the total number of labels (not including title, axis, or tic labels) in the plot specified by ***plot\_index***
>
> *int* **GetDoumentProperty(*"data size", data\_index*)**
>
> Returns the size of the arrays in the data object specified by ***data\_index***
>
> DESCRIPTIONS, TITLES, AND LABELS
>
> *char* **GetDoumentProperty(*"data label", data\_index*)**
>
> Returns the label of the data object specified by ***data\_index***
>
> *char* **GetDoumentProperty(*"data description", data\_index*)**
>
> Returns the description of the data object specified by ***data\_index***
>
> *char* **GetDoumentProperty(*"data x description", data\_index*)**
>
> Returns the description of the x array in the data object specified by ***data\_index***
>
> *char* **GetDoumentProperty(*"data y description", data\_index*)**
>
> Returns the description of the y array in the data object specified by ***data\_index***
>
> *char* **GetDoumentProperty(*"data error description", data\_index*)**
>
> Returns the description of the error array in the data object specified by ***data\_index***
>
> *char* **GetDoumentProperty(*"sheet description", sheet\_index*)**
>
> Returns the description of the sheet specified by ***sheet\_index***
>
> *char* **GetDoumentProperty(*"plot title", plot\_index*)**
>
> Returns the title of the plot specified by ***plot\_index***
>
> INDICES
>
> *int* **GetDoumentProperty(*"indices of highlighted data"*)**
>
> Returns the indices of data objects highlighted in the document contents
>
> *int* **GetDoumentProperty(*"index of current sheet"*)**
>
> Returns the index of the most recently selected sheet
>
> *int* **GetDoumentProperty(*"indices of highlighted sheets"*)**
>
> Returns the indices of the sheets highlighted in the document contents
>
> *int* **GetDoumentProperty(*"indices of data in sheet", sheet\_index*)**
>
> Returns the indices of the data objects in the sheet specified by ***sheet\_index***
>
> *int* **GetDoumentProperty(*"index of current plot"*)**
>
> Returns the index of the most recently selected plot
>
> *int* **GetDoumentProperty(*"indices of highlighted plots"*)**
>
> Returns the indices of the plots highlighted in the document contents
>
> *int* **GetDoumentProperty(*"indices of data in plot", plot\_index*)**
>
> Returns the indices of the data objects in the plot specified by ***plot\_index***
>
> *int* **GetDoumentProperty(*"indices of checked in data list"*)**
>
> Returns the indices of data objects checked in the data list
>
> *int* **GetDoumentProperty(*"indices of highlighted in data list"*)**
>
> Returns the indices of data objects highlighted in the data list
>
> *int* **GetDoumentProperty(*"indices of all in data list"*)**
>
> Returns the indices of all data objects in the data list
>
> *int* **GetDoumentProperty(*"indices of checked in data sheet", sheet\_index*)**
>
> Returns the indices of data objects checked in the sheet specified by ***sheet\_index***
>
> *int* **GetDoumentProperty(*"indices of highlighted in data sheet", sheet\_index*)**
>
> Returns the indices of data objects highlighted in the sheet specified by ***sheet\_index***
>
> *int* **GetDoumentProperty(*"indices of all in data sheet", sheet\_index*)**
>
> Returns the indices of all data objects in the sheet specified by ***sheet\_index***
>
> *int* **GetDoumentProperty(*"indices of highlighted data in contents"*)**
>
> Returns the indices of data objects highlighted in the document contents
>
> *int* **GetDoumentProperty(*"indices of data in highlighted sheets"*)**
>
> Returns the indices of data objects in the sheets highlighted in the document contents
>
> *int* **GetDoumentProperty(*"indices of data in highlighted plots"*)**
>
> Returns the indices of data objects in the plots highlighted in the document contents
>
> *int* **GetDoumentProperty(*"indices of data in highlighted in contents"*)**
>
> Returns the indices of data objects in highlighted data, sheets, and plots in the document contents
>
> *int* **GetDoumentProperty(*"indices of selected data in current plot"*)**
>
> Returns the indices of selected data objects in the current plot
>
> *int* **GetDoumentProperty(*"indices of displayed data in current plot"*)**
>
> Returns the indices of displayed data objects in the current plot
>
> *int* **GetDoumentProperty(*"indices of edit data"*)**
>
> Returns the indices of data objects currently being edited
>
> SELECTION RECTANGLE
>
> *double* **GetDoumentProperty(*"selection rectangle x min"*)**
>
> Returns the minimum x value of the selection rectangle in the current plot
>
> *double* **GetDoumentProperty(*"selection rectangle x max"*)**
>
> Returns the maximum x value of the selection rectangle in the current plot
>
> *double* **GetDoumentProperty(*"selection rectangle y min"*)**
>
> Returns the minimum y value of the selection rectangle in the current plot
>
> *double* **GetDoumentProperty(*"selection rectangle y max"*)**
>
> Returns the maximum y value of the selection rectangle in the current plot
>
> COLOR
>
> *double* **GetDoumentProperty(*"line stroke color for data in plot"*, *data\_index, plot\_index*)**
>
> Returns an array containing the RGB components and opacity (in the order red, green, blue, alpha) of the line stroke color for the data object specified by ***data\_index*** in the plot specified by ***plot\_index***
>
> *double* **GetDoumentProperty(*"symbol stroke color for data in plot"*, *data\_index, plot\_index*)**
>
> Returns an array containing the RGB components and opacity (in the order red, green, blue, alpha) of the symbol stroke color for the data object specified by ***data\_index*** in the plot specified by ***plot\_index***
>
> *double* **GetDoumentProperty(*"symbol fill color for data in plot"*, *data\_index, plot\_index*)**
>
> Returns an array containing the RGB components and opacity (in the order red, green, blue, alpha) of the symbol fill color for the data object specified by ***data\_index*** in the plot specified by ***plot\_index***
>
> *double* **GetDoumentProperty(*"color for data in plot"*, *data\_index, plot\_index*)**
>
> Returns an array containing the RGB color components and opacity (in the order red, green, blue, alpha) of the color for the data object specified by ***data\_index*** in the plot specified by ***plot\_index***. The color components return correspond to the data line stroke if displayed, or otherwise the symbol stroke if displayed, or otherwise the symbol fill if displayed, or otherwise the data line stroke.
>
> SCRIPTS
>
> *char* **GetDoumentProperty(*"script path"*)**
>
> Returns the string value of the file path field in the document's interpret script window

**SetDocumentProperty(*key*, *arguments*)**

Sets the value of the document property specified by one of the following ***key*** strings and key dependent ***arguments***:

> DESCRIPTIONS, TITLES, AND LABELS
>
> **SetDoumentProperty(*"data description", data\_index, new\_description*)**
>
> Sets the description of the data object specified by ***data\_index***with the ***new\_description*** string
>
> **SetDoumentProperty(*"data x description", data\_index, new\_description*)**
>
> Sets the description of the x array in the data object specified by ***data\_index*** with the ***new\_description*** string
>
> **SetDoumentProperty(*"data y description", data\_index, new\_description*)**
>
> Sets the description of the y array in the data object specified by ***data\_index*** with the ***new\_description*** string
>
> **SetDoumentProperty(*"data error description", data\_index, new\_description*)**
>
> Sets the description of the error array in the data object specified by ***data\_index*** with the ***new\_description*** string
>
> **SetDoumentProperty(*"sheet description", sheet\_index, new\_description*)**
>
> Sets the description of the sheet specified by ***sheet\_index*** with the ***new\_description*** string
>
> **SetDoumentProperty(*"plot title", plot\_index, new\_title*)**
>
> Sets the title of the plot specified by ***plot\_index*** with the***new\_title*** string
>
> **SetDoumentProperty(*"plot x axis label", plot\_index, new\_axis\_label*)**
>
> Sets the x axis label of the plot specified by ***plot\_index*** with the ***new\_axis\_label*** string
>
> **SetDoumentProperty(*"plot x axis label", plot\_index, new\_axis\_label*)**
>
> Sets the y axis label of the plot specified by ***plot\_index*** with the ***new\_axis\_label*** string
>
> **SetDoumentProperty(*"plot r axis label", plot\_index, new\_axis\_label*)**
>
> Sets the residuals axis label of the plot specified by ***plot\_index*** with the ***new\_axis\_label*** string
>
> AXIS LIMITS
>
> **SetDoumentProperty(*"plot x axis minimum", plot\_index, new\_value*)**
>
> Sets the x axis minimum of the plot specified by ***plot\_index*** with the ***new\_value***
>
> **SetDoumentProperty(*"plot x axis maximum", plot\_index, new\_value*)**
>
> Sets the x axis maximum of the plot specified by ***plot\_index*** with the ***new\_value***
>
> **SetDoumentProperty(*"plot y axis minimum", plot\_index, new\_value*)**
>
> Sets the y axis minimum of the plot specified by ***plot\_index*** with the ***new\_value***
>
> **SetDoumentProperty(*"plot y axis maximum", plot\_index, new\_value*)**
>
> Sets the y axis maximum of the plot specified by ***plot\_index*** with the ***new\_value***
>
> **SetDoumentProperty(*"plot r axis minimum", plot\_index, new\_value*)**
>
> Sets the residuials axis minimum of the plot specified by ***plot\_index*** with the ***new\_value***
>
> **SetDoumentProperty(*"plot r axis maximum", plot\_index, new\_value*)**
>
> Sets the residuals axis maximum of the plot specified by ***plot\_index*** with the ***new\_value***
>
> **SetDoumentProperty(*"plot x axis limits", plot\_index, new\_minimum\_value, new\_maximum\_value*)**
>
> Sets the x axis minimum and maximum of the plot specified by ***plot\_index*** with the ***new\_minimum\_value*** and ***new\_maximum\_value***
>
> **SetDoumentProperty(*"plot y axis limits", plot\_index, new\_minimum\_value, new\_maximum\_value*)**
>
> Replaces the y axis minimum and maximum of the plot specified by ***plot\_index*** with the ***new\_minimum\_value*** and ***new\_maximum\_value***
>
> **SetDoumentProperty(*"plot r axis limits", plot\_index, new\_minimum\_value, new\_maximum\_value*)**
>
> Sets the residuals axis minimum and maximum of the plot specified by ***plot\_index*** with the ***new\_minimum\_value*** and ***new\_maximum\_value***
>
> DISPLAY
>
> **SetDoumentProperty(*"plot display model", plot\_index, display\_flag*)**
>
> Sets the display model flag in the plot specified by ***plot\_index*** with the ***new\_value*** of 0 to hide or 1 to display models
>
> COLOR
>
> **SetDoumentProperty(*"color for label in plot", plot\_index, label\_index,* *color\_components*)**
>
> Sets the color of the label specified by ***label\_index*** in the plot specified by ***plot\_index*** with the color component array (red, green, blue, alpha) specified by ***color\_components***

**DocumentAction(*key*, *arguments*)**

Sets the value of the document property specified by one of the following ***key*** strings and key dependent ***arguments***:

> SELECTION
>
> **DocumentAction(*"select data in contents", data\_index*)**
>
> Selects the data object specified by ***data\_index*** in the document contents
>
> **DocumentAction(*"select plot in contents", plot\_index*)**
>
> Selects the plot specified by***plot\_index***in the document contents
>
> **DocumentAction(*"new plot label", plot\_index, new\_label, x\_location, y\_location*)**
>
> Adds the***new\_label*** string to the plot specified by ***plot\_index*** at the location specified by ***x\_location*** and ***y\_location*** in view coordinates
>
> MODELS
>
> **DocumentAction(*"linear model for data", data\_index, slope, intercept [, x\_minimum, y\_maximum]*)**
>
> Adds a linear model to the data object specified by ***data\_index*** with the specified ***slope***, ***intercept*** values. The x range of the model is restricted by ***x\_minimum, y\_maximum*** if supplied. This function does not fit the data.
>
> **DocumentAction(*"linear model for plot", plot\_index, slope, intercept [, x\_minimum, y\_maximum]*)**
>
> Adds a linear model to the data objects in the the plot specified by ***plot\_index*** with the specified ***slope***, ***intercept*** values. The x range of the model is restricted by ***x\_minimum, y\_maximum*** if supplied. This function does not fit the data.
>
> SHEETS
>
> **DocumentAction(*"new sheet", description*)**
>
> Creates a new sheet with a ***description*** string

**UpdateDocumentViews()**

Updates document views to reflect changes

### Plot functions

*type\_depends\_on\_key* **GetPlotProperty(*key*, *arguments*)**

Returns the value of the plot property specified by one of the following ***key*** strings and key dependent ***arguments***:

> COUNT AND SIZE
>
> *int* **GetPlotProperty(*"data count", plot\_index*)**
>
> Returns the total number of data objects in the plot specified by ***plot\_index***
>
> *int* **GetPlotProperty(*"label count"*, *plot\_index*)**
>
> Returns the total number of text labels in the plot specified by ***plot\_index***
>
> INDICES
>
> *int* **GetPlotProperty(*"data indices", plot\_index*)**
>
> Returns the indicies of the data in the plot specified by ***plot\_index***
>
> *int* **GetPlotProperty(*"selected data indices", plot\_index*)**
>
> Returns the indicies of the selected data in the plot specified by ***plot\_index***
>
> *int* **GetPlotProperty(*"displayed data indices", plot\_index*)**
>
> Returns the indicies of the displayed data in the plot specified by ***plot\_index***
>
> SELECTION RECTANGLE
>
> *double* **GetPlotProperty(*"selection rectangle x min", plot\_index*)**
>
> Returns the x minimum of the selection rectangle in the plot specified by ***plot\_index***
>
> *double* **GetPlotProperty(*"selection rectangle x max", plot\_index*)**
>
> Returns the x maximum of the selection rectangle in the plot specified by ***plot\_index***
>
> *double* **GetPlotProperty(*"selection rectangle y min", plot\_index*)**
>
> Returns the y minimum of the selection rectangle in the plot specified by ***plot\_index***
>
> *double* **GetPlotProperty(*"selection rectangle y max", plot\_index*)**
>
> Returns the y maximum of the selection rectangle in the plot specified by ***plot\_index***
>
> COLOR
>
> *double* **GetPlotProperty(*"data symbol stroke color", plot\_index, data\_index*)**
>
> Returns an array containing the RGB components and opacity (in the order red, green, blue, alpha) of the symbol stroke color for the data object specified by ***data\_index*** in the plot specified by ***plot\_index***
>
> *double* **GetPlotProperty(*"data symbol fill color", plot\_index, data\_index*)**
>
> Returns an array containing the RGB components and opacity (in the order red, green, blue, alpha) of the symbol fill color for the data object specified by ***data\_index*** in the plot specified by ***plot\_index***
>
> *double* **GetPlotProperty(*"data line stroke color", plot\_index, data\_index*)**
>
> Returns an array containing the RGB components and opacity (in the order red, green, blue, alpha) of the line stroke color for the data object specified by ***data\_index*** in the plot specified by ***plot\_index***
>
> *double* **GetPlotProperty(*"data color", plot\_index, data\_index*)**
>
> Returns an array containing the RGB color components and opacity (in the order red, green, blue, alpha) of the color for the data object specified by ***data\_index*** in the plot specified by ***plot\_index***. The color components return correspond to the data line stroke if displayed, or otherwise the symbol stroke if displayed, or otherwise the symbol fill if displayed, or otherwise the data line stroke.

**SetPlotProperty(*key*, *arguments*)**

Sets the value of the document property specified by one of the following ***key*** strings and key dependent ***arguments***:

> TITLE AND LABELS
>
> **SetPlotProperty(*"title", plot\_index, new\_title*)**
>
> Sets the title of the plot specified by ***plot\_index*** with the***new\_title*** string
>
> **SetPlotProperty(*"x axis label", plot\_index, new\_label*)**
>
> Sets the x axis label of the plot specified by ***plot\_index*** with the ***new\_label*** string
>
> **SetPlotProperty(*"y axis label", plot\_index, new\_label*)**
>
> Sets the y axis label of the plot specified by ***plot\_index*** with the ***new\_label*** string
>
> **SetPlotProperty(*"r axis label", plot\_index, new\_label*)**
>
> Sets the residuals axis axis label of the plot specified by ***plot\_index*** with the ***new\_label*** string
>
> AXIS LIMITS
>
> **SetPlotProperty(*"plot x axis minimum", plot\_index, new\_value*)**
>
> Sets the x axis minimum of the plot specified by ***plot\_index*** with the ***new\_value***
>
> **SetPlotProperty(*"plot x axis maximum", plot\_index, new\_value*)**
>
> Sets the x axis maximum of the plot specified by ***plot\_index*** with the ***new\_value***
>
> **SetPlotProperty(*"plot y axis minimum", plot\_index, new\_value*)**
>
> Sets the y axis minimum of the plot specified by ***plot\_index*** with the ***new\_value***
>
> **SetPlotProperty(*"plot y axis maximum", plot\_index, new\_value*)**
>
> Sets the y axis maximum of the plot specified by ***plot\_index*** with the ***new\_value***
>
> **SetPlotProperty(*"plot r axis minimum", plot\_index, new\_value*)**
>
> Sets the residuals axis axis minimum of the plot specified by ***plot\_index*** with the ***new\_value***
>
> **SetPlotProperty(*"plot r axis maximum", plot\_index, new\_value*)**
>
> Sets the residuals axis axis maximum of the plot specified by ***plot\_index*** with the ***new\_value***
>
> **SetPlotProperty(*"x axis limits", plot\_index, new\_minimum\_value, new\_maximum\_value*)**
>
> Sets the x axis minimum and maximum of the plot specified by ***plot\_index*** with the ***new\_minimum\_value*** and ***new\_maximum\_value***
>
> **SetPlotProperty(*"y axis limits", plot\_index, new\_minimum\_value, new\_maximum\_value*)**
>
> Replaces the y axis minimum and maximum of the plot specified by ***plot\_index*** with the ***new\_minimum\_value*** and ***new\_maximum\_value***
>
> **SetPlotProperty(*"r axis limits", plot\_index, new\_minimum\_value, new\_maximum\_value*)**
>
> Sets the residuals axis minimum and maximum of the plot specified by ***plot\_index*** with the ***new\_minimum\_value*** and ***new\_maximum\_value***
>
> SELECTION RECTANGLE
>
> **SetPlotProperty(*"selection rectangle xmin", plot\_index, new\_value*)**
>
> Sets the x minimum of the selection rectangle in the plot specified by ***plot\_index*** with the ***new\_value***
>
> **SetPlotProperty(*"selection rectangle xmax", plot\_index, new\_value*)**
>
> Sets the x maximum of the selection rectangle in the plot specified by ***plot\_index*** with the ***new\_value***
>
> **SetPlotProperty(*"selection rectangle ymin", plot\_index, new\_value*)**
>
> Sets the y minimum of the selection rectangle in the plot specified by ***plot\_index*** with the ***new\_value***
>
> **SetPlotProperty(*"selection rectangle ymax", plot\_index, new\_value*)**
>
> Sets the y maximum of the selection rectangle in the plot specified by ***plot\_index*** with the ***new\_value***
>
> COLOR
>
> **SetPlotProperty(*"label color", plot\_index, label\_index, color\_components*)**
>
> Sets the color of the label specified by ***label\_index*** in the plot specified by ***plot\_index*** with the color component array (red, green, blue, alpha) specified by ***color\_components***
>
> DISPLAY
>
> **SetPlotProperty(*"display model", plot\_index, new\_value*)**
>
> Sets the display model flag in the plot specified by ***plot\_index*** with the ***new\_value*** of 0 to hide or 1 to display models

**PlotAction(*key*, *arguments*)**

Sets the value of the document property specified by one of the following ***key*** strings and key dependent ***arguments***:

> MODELS
>
> **PlotAction(*"linear model", plot\_index, slope, intercept [, x\_minimum, y\_maximum]*)**
>
> Adds a linear model to the data objects in the the plot specified by ***plot\_index*** with the specified ***slope***, ***intercept*** values. The x range of the model is restricted by ***x\_minimum, y\_maximum*** if supplied. This function does not fit the data.
>
> LABELS
>
> **PlotAction(*"new label", plot\_index, label\_string\_or\_char\_array*)**
>
> Creates a new label with ***label\_string\_or\_char\_array*** in the plot specified by ***plot\_index***

### File functions

*char* **GetOpenFilePath(*prompt\_message*)**

Returns the path of the file selected by the user after initiating an open file navigation dialog with the ***prompt\_message*** string

*char* **GetSaveFilePath(*prompt\_message*)**

Returns the path of the file selected by the user after initiating a save file navigation dialog with the ***prompt\_message*** string

### Other functions

**EndWithError(*error\_message*)**

Immediately terminates script execution and displays the ***error\_message*** string

**debug()**

Does nothing. Used for debugging.

## Fonts, charcter attributes, paragraph styles, and unicode characters

Although the interpreter does not directly recognize unicode characters or attributed strings, tags specifiying unicode charaters, fonts, character attributes, or paragraph styles can be embeded within strings. When supplied as arguments to functions that set attributed strings such as plot titles, axis labels, or other labels, the tags are processed and removed to generate attributed strings with the specified characters and attributes. The syntax for tags is resembles that used in markup languages such as HTML.

### General syntax

Tags conform to the general syntax:

> **<tag>***string***</tag>**

or

> **<tag subtag: value, value2, ..., valueN; subtag2: value, ..., valueN; ...; subtagN: value, ..., valueN>***string***</tag>**

where *string* can have any number of one byte UTF-8 extended ASCII characters. Tags and subtags are case-insensitive (i.e., the tag FONT is the same as Font or font or foNt and so on). However, string values such as font names are case sensitive. Subtags can be used individually or combined in any order. Tags can be nested or overlapping, provided the effect of the tag does not alter the characters in ***string*** such that nested or overlapping tags would not be recogized and that nested tags are not the same tag.

### Font

The FONT tag has three subtags that specifiy the font name, size, and/or color:

> <FONT NAME: *font\_name*; SIZE: *size*; COLOR: red, green, blue, alpha> ... </FONT>

where *font\_name* is a string corresponing to an available font family name including the face if desired (e.g. Arial), *size* is double value in points, and color is a list of double values (ranging from 0.0-1.0) corresponding to RBG components and opacity.

### Character attributes

The following tags specify character attributes:

> <SUB> ... </SUB>
>
> Subscript with the default size reduction and baseline offest
>
> <SUP> ... </SUP>
>
> Superscript with the default size reduction and baseline offest
>
> <P> ... </P>
>
> Regular or plain type face (i.e., non-bold, non-italic, etc).
>
> <B> ... </B>
>
> Bold type face.
>
> <I> ... </I>
>
> Italic type face.
>
> <BI> ... </BI> or <IB> ... </IB>
>
> Bold-Italic type face.
>
> <BAS *offset*> ... </BAS>
>
> Baseline offset where*offset* is a double value in points.

### Underline and strikethrough

The following tags specify underline or strikethrough and share optional subtags for line style, pattern, and color:

> <U STYLE: *style*; PATTERN: *pattern*; COLOR: *color*> ... </U>
>
> Underline
>
> <S STYLE: *style*; PATTERN: *pattern*; COLOR: *color*> ... </S>
>
> Strikethrough

where *style* is one of the following strings: None, Single, Thick, or Double; *pattern* is one of the following strings: Solid, Dot, Dash, DashDot, or DashDotDot; and *color* is a list of double values (ranging from 0.0-1.0) corresponding to RBG components and opacity.

### Paragraph style

The PSTYLE tag has two subtags for control of text alignment and line height:

> <PSTYLE ALIGN: *alignment*; LHM: *line\_height\_multiplier*> ... </PSTYLE>

where*line\_height\_multiplier* is a double value in units of the default line height (1.0 for single, 2.0 for double, etc) and *alignment* is one of the following strings (only the first letter is required): Left, Right, Center, Justified, or Natural.

### Unicode characters

The UTF8 tag specifies conversion of hexadecimal code sequences corresponding to one or more unicode characters:

> <UTF8>*hexadecimal\_code\_sequences*</UTF8>

where *hexadecimal\_code\_sequence*s is a string consisting of any number of two character hexadecimal code sequences corresponding to one or more unicode characters with UTF-8 encoding. The recognized hexadecimal code sequences for each unicode character are listed in the Unicode section of the OSX Character Viewer. Each hexadecimal code sequence should be seperated by a space, \x, or 0x.

### Examples

The following examples assume a default font of Arial, 12 pt , black, with no attributes or styles.

***Font***

> Tagged string
>
> > "<font name: Times>Hello</font> <font name: Courier; size: 18>World</font> <font color: red>!!!</font>"
>
> Attributed string
>
> > Hello World !!!

***Type face***

> Tagged string
>
> > "<b>Hello</b> <i>world</i> <bi>!!!</bi>"
>
> Attributed string
>
> > **Hello** *World* ***!!!***

***Superscript***

> Tagged string
>
> > "x<sup>2</sup> + y<sup>2</sup> = z<sup>2</sup>"
>
> Attributed string
>
> > x^2^ + y^2^ = z^2^

***Unicode***

> Tagged string
>
> > "<UTF8>CE B1 CE B2 CE B3</UFT8>"
>
> Attributed string
>
> > αβγ
>
> where the UTF-8 hexadecimal codes CE B1, CE B2, and CE B3 encode the Greek characters α, β, and γ, respectively.

## Custom functions

The interpreter also recognizes functions defined within a script. The int main() function is one such example but additional functions can be included, typically following the end of the main function but the order is not important as long as there is no overlap. All functions have the same generic form:

> *return\_type* **function\_name** (type ***argument\_name***, type ***argument\_name***, ...)
>
> {
>
> // code for function
>
> }
