# Supplementary material for: Structural Organization and Dynamics of Homodimeric Cytohesin Family Arf GTPase Exchange Factors in Solution and on Membranes
Source: Structure. 2019 Dec 3;27(12):1782–1797.e7. doi: 10.1016/j.str.2019.09.007 (PMC6948192; doi:10.1016/j.str.2019.09.007)
Supplement: Data S1. Software, Related to the STAR Methods [file mmc3.zip › Software/Dela App Scripts/Dela.app/Contents/Resources/Dela.help/Contents/Resources/English.lproj/pgs/select_model.html]

xml version="1.0" encoding="utf-8"?


Model selection


Home

Contents


# Model selection

## Selecting a model

In Dela, each data object maintains its own list of unique model objects that organize a hierarchy of related objects that collectively allow models to be calculated, displayed, and locally or globally fit. A model is created upon selection if it does not already exist in the data object's model list. Selection also designates the model as the target for subsequent actions including calculation, display and fitting.

### To select a model:

1. Create a plot of the data to be modelled or skip to the next step if the plot already exists.

2. Highlight the plot in the Document Contents.

3. Left-click ***Model*** from the document toolbar or choose ***Select...*** from the application's Model menu.

4. Make sure ***Highlighted Plots in Contents*** is displayed in the ***Data from*** popup menu.

5. Choose a model from the ***Model*** popup menu.

6. Enter an integer value equal to or greater than one in the ***Number of terms*** field.

7. Left-click the ***Select*** button.

## Data source

Although models can in principle be selected for data from any source, it is advisable in practice to restrict the data source to the data in a single plot.

## Model

Dela maintains a master list of available models, which are displayed in the model popup menu. Each of the available models is in fact a template for a class of related models formed by summation of weighted terms, where the individual terms have the same functional form. Thus, the actual model created upon selection depends on the functional form chosen from the model popup menu as well as the number of terms. Models with the same functional form but a different number of terms are distinct. For example, a single exponential function would have one term, a biexponential function would have two terms, and so on.

For more information about models, see Model Functions.
