# Supplementary material for: Structural Organization and Dynamics of Homodimeric Cytohesin Family Arf GTPase Exchange Factors in Solution and on Membranes
Source: Structure. 2019 Dec 3;27(12):1782–1797.e7. doi: 10.1016/j.str.2019.09.007 (PMC6948192; doi:10.1016/j.str.2019.09.007)
Supplement: Data S1. Software, Related to the STAR Methods [file mmc3.zip › Software/Dela App Scripts/Dela.app/Contents/Resources/Dela.help/Contents/Resources/English.lproj/pgs/set_parameters.html]

xml version="1.0" encoding="utf-8"?


Parameters panel


Home

Contents


# Set parameters panel

## Purpose and requirements

The set Parameter panel allows parameter values to be automatically set (i.e. calculated), provided there exists a mathematical expression describing the relationship between the parameter values in consective data objects. The mathematical relathionship between parameter values in consective data objects must be identical over the specified data range and must conform to the syntax of Dela's interpreter.

### To set parameters:

1. Select the parameter to set and enter a value in the parameters table for the selected parameter in the first data object in the data range

2. Select the first and last data objects in the data range of interest

3. Enter the mathematical relationship between parameter values in consecutive data objects as a function of t, where t refers to value of parameter in the previous object.

For more general information related to viewing, editing and fitting parameters, see View Parameters panel.
