# Supplementary material for: Structural Organization and Dynamics of Homodimeric Cytohesin Family Arf GTPase Exchange Factors in Solution and on Membranes
Source: Structure. 2019 Dec 3;27(12):1782–1797.e7. doi: 10.1016/j.str.2019.09.007 (PMC6948192; doi:10.1016/j.str.2019.09.007)
Supplement: Data S1. Software, Related to the STAR Methods [file mmc3.zip › Software/Dela App Scripts/Dela.app/Contents/Resources/Dela.help/Contents/Resources/English.lproj/pgs/statistics.html]

xml version="1.0" encoding="utf-8"?


Statistics


Home

Contents


# Statistics

## Calculating statistics

Common statics can be calculated for the X Y and Error arrays of any data object.

### To calculate statistics for data object(s):

1. In the document contents, select the data objects, sheets, and/or plots with the data for calculation of statistics.

2. Click ***Statistics→Statics***  from the document toolbar.

3. Change the data source if necessary, modify the options as desired, and click  ***Statistics***.

## Options

The following options are presented when ***Statistics→Statistics*** is selected from the document toolbar.

### Data from

Selects the intended data source.

## Ouput

A new data object(s) will be created with the following statistics listed in the X Y and Errors columns.

• sample size - the number of data points

• minimum - the minimum value

• maximum - the maximum value

• extent - the maximum value minus the minimum value

• sum - the sum of the array values

• mean - the average of the array values

• meadian - the meadian of the array values

• standard deviation - the small sample size estimation of the standard deviation of the array values

• standard error - the small sample size estimation of the standard error of the array values

• variance - the small sample size estimation of the variance of the array values

• kurtosis - the kurtosis of the array values

• moments - the first through fourth moments of the array values

• cumulants - the first through fourth moments of the array values

• autocorrelation - the autocorrelation of the array values
