# Supplementary material for: Structural Organization and Dynamics of Homodimeric Cytohesin Family Arf GTPase Exchange Factors in Solution and on Membranes
Source: Structure. 2019 Dec 3;27(12):1782–1797.e7. doi: 10.1016/j.str.2019.09.007 (PMC6948192; doi:10.1016/j.str.2019.09.007)
Supplement: Data S1. Software, Related to the STAR Methods [file mmc3.zip › Software/Dela App Scripts/Dela.app/Contents/Resources/Dela.help/Contents/Resources/English.lproj/pgs/string_attribute_style_tags.html]

xml version="1.0" encoding="utf-8"?


String Attribute Style Tags


Home

Contents


# String attribute and style tags

## Fonts, charcter attributes, paragraph styles, and unicode characters

Tags specifiying unicode charaters, fonts, character attributes, or paragraph styles can be embeded within strings passed as arguments to DELA python modules or DELA interpreter functions that set attributed strings such as plot titles, axis labels, or plot labels. The tags are processed and removed to generate attributed strings with the specified characters and attributes. The syntax for the tags resembles that used in markup languages.

### General syntax

Tags conform to the general syntax:

> **<tag>***string***</tag>**

or

> **<tag subtag: value, value2, ..., valueN; subtag2: value, ..., valueN; ...; subtagN: value, ..., valueN>***string***</tag>**

where *string* can have any number of one byte UTF-8 extended ASCII characters. Tags and subtage are case-insensitive (i.e., the tag FONT is the same as Font or font or foNt and so on). However, string values such as font names are case sensitive. Subtags can be used individually or combined in any order. Tags can be nested or overlapping, provided the effect of the tag does not alter the characters in ***string*** such that nested or overlapping tags would not be recogized and that nested tags are not the same tag.

### Font

The FONT tag has three subtags that specifiy the font name, size, and/or color:

> <FONT NAME: *font\_name*; SIZE: *size*; COLOR: red, green, blue, alpha> ... </FONT>

where *font\_name* is a string corresponing to an available font family name including the face if desired (e.g. Arial), *size* is double value in points, and color is a list of double values (ranging from 0.0-1.0) corresponding to RBG components and opacity.

### Character attributes

The following tags specify character attributes:

> <SUB> ... </SUB>
>
> Subscript with the default size reduction and baseline offest
>
> <SUP> ... </SUP>
>
> Superscript with the default size reduction and baseline offest
>
> <P> ... </P>
>
> Regular or plain type face (i.e., non-bold, non-italic, etc).
>
> <B> ... </B>
>
> Bold type face.
>
> <I> ... </I>
>
> Italic type face.
>
> <BI> ... </BI> or <IB> ... </IB>
>
> Bold-Italic type face.
>
> <BAS *offset*> ... </BAS>
>
> Baseline offset where*offset* is a double value in points.

### Underline and strikethrough

The following tags specify underline or strikethrough and share optional subtags for line style, pattern, and color:

> <U STYLE: *style*; PATTERN: *pattern*; COLOR: *color*> ... </U>
>
> Underline
>
> <S STYLE: *style*; PATTERN: *pattern*; COLOR: *color*> ... </S>
>
> Strikethrough

where *style* is one of the following strings: None, Single, Thick, or Double; *pattern* is one of the following strings: Solid, Dot, Dash, DashDot, or DashDotDot; and *color* is a list of double values (ranging from 0.0-1.0) corresponding to RBG components and opacity.

### Paragraph style

The PSTYLE tag has two subtags for control of text alignment and line height:

> <PSTYLE ALIGN: *alignment*; LHM: *line\_height\_multiplier*> ... </PSTYLE>

where*line\_height\_multiplier* is a double value in units of the default line height (1.0 for single, 2.0 for double, etc) and *alignment* is one of the following strings (only the first letter is required): Left, Right, Center, Justified, or Natural.

### Unicode characters

The UTF8 tag specifies conversion of hexadecimal code sequences corresponding to one or more unicode characters:

> <UTF8>*hexadecimal\_code\_sequences*</UTF8>

where *hexadecimal\_code\_sequence*s is a string consisting of any number of two character hexadecimal code sequences corresponding to one or more unicode characters with UTF-8 encoding. The recognized hexadecimal code sequences for each unicode character are listed in the Unicode section of the OSX Character Viewer. Each hexadecimal code sequence should be seperated by a space, \x, or 0x.

### Examples

The following examples assume a default font of Arial, 12 pt , black, with no attributes or styles.

***Font***

> Tagged string
>
> > "<font name: Times>Hello</font> <font name: Courier; size: 18>World</font> <font color: red>!!!</font>"
>
> Attributed string
>
> > Hello World !!!

***Type face***

> Tagged string
>
> > "<b>Hello</b> <i>world</i> <bi>!!!</bi>"
>
> Attributed string
>
> > **Hello** *World* ***!!!***

***Superscript***

> Tagged string
>
> > "x<sup>2</sup> + y<sup>2</sup> = z<sup>2</sup>"
>
> Attributed string
>
> > x^2^ + y^2^ = z^2^

***Unicode***

> Tagged string
>
> > "<UTF8>CE B1 CE B2 CE B3</UFT8>"
>
> Attributed string
>
> > αβγ
>
> where the UTF-8 hexadecimal codes CE B1, CE B2, and CE B3 encode the Greek characters α, β, and γ, respectively.
