# Supplementary material for: Structural Organization and Dynamics of Homodimeric Cytohesin Family Arf GTPase Exchange Factors in Solution and on Membranes
Source: Structure. 2019 Dec 3;27(12):1782–1797.e7. doi: 10.1016/j.str.2019.09.007 (PMC6948192; doi:10.1016/j.str.2019.09.007)
Supplement: Data S1. Software, Related to the STAR Methods [file mmc3.zip › Software/Dela App Scripts/Dela.app/Contents/Resources/Dela.help/Contents/Resources/English.lproj/pgs/symbols.html]

xml version="1.0" encoding="utf-8"?


Plot symbols panel


Home

Contents


# Plot Symbols panel

## Plots symbols

Symbols and lines for plot objects are controlled via the Plot Symbols panel.

### To access the Plot Symbols panel:

• ***double left-click on an data point*** - opens the Plot Symbols paenl to the double left-clicked data

or

• ***left-click Symbols in document tool bar*** - opens the Plot Symbols panel

or

• ***choose Symbols... from the application's Plot menu*** - opens the Plot Symbols panel

### To select a data object:

• ***select from the data menu***

or

• ***double left-click on an data point***

If Apply to all is checked, changes will be applied to all data objects in the current plot.

### To select the source:

• left-click *D* (data), *M* (current model), *R* (residuals for current model), *IR* (instrument response) or *All* in the Source selector

If All is selected, changes will be applied to all source objects associated with the selected data object.

## Metrics

Basic properties of symbols and lines can be controlled from the Metrics view of the Plot Symbols panel.

### Line Type and Width

• ***Type*** - determines the line type (none, continuous, or dashed)

• ***Width*** - the *real value* for the line width in pixels

• ***Color Well*** - sets the line color

### Symbol Type and Size

• ***Type*** - determines the symbol type (none, point, circle, square, ...)

• ***Width*** - the *real value* for the symbol size in pixels

• ***Color Well*** - sets the symbol stroke color

### Symbol Fill

• ***Type*** - determines the symbol type (none or solid)

• ***Opacity*** - the *real value* of the symbol fill opacity (i.e., the alpha component) ranging from 0 (100% transparent) to 1 (0% transparent)

• ***Color Well*** - sets the symbol fill color

## Stroke

The drawing (stroking) of a line or symbol can be controlled from the Stroke view of the Plot Symbols panel.

### Line / Symbol

• ***Type*** - determines the display type (none, continuous, or dashed)

• ***Join*** - determines how consecutive line segments are joined (miter, round, or bevel).

\* ***Cap*** - determines the shape at the end of a line segment (butt, square, or round)

• ***Color Well*** - sets the color

• ***Width*** - the *real value* of the width in pixels

• ***Miter Limit*** - the *real value* of the miter limit in pixels (limit for the length of the miter joint between line segments)

• ***Opacity*** - the *real value* of the opacity (i.e., the alpha component) ranging from 0 (100% transparent) to 1 (0% transparent)

## Shadow

The display and appearance of semi-transparent 'shadows' can be controlled from the Shadow view of the Plot Symbols panel.

### Line / Symbol Stroke / Symbol Fill

• Type - determines whether shadows are displayed

• Blur - the *real value* for the blurring factor

• Color Well - sets the shadow color

• Offset X - the *real value* of the X offset from the object casting the shadow

• Offset Y - the *real value* of the Y offset from the object casting the shadow

• Opacity - the *real value* of the opacity (i.e., the alpha component) ranging from 0 (100% transparent) to 1 (0% transparent)
